# Aquatic fauna from the Takarkori rock shelter reveals the Holocene central Saharan climate and palaeohydrography

**DOI:** 10.1371/journal.pone.0228588

**Published:** 2020-02-19

**Authors:** Wim Van Neer, Francesca Alhaique, Wim Wouters, Katrien Dierickx, Monica Gala, Quentin Goffette, Guido S. Mariani, Andrea Zerboni, Savino di Lernia

**Affiliations:** 1 Operational Direction Earth and History of Life, Royal Belgian Institute of Natural Sciences, Brussels, Belgium; 2 Laboratory of Biodiversity and Evolutionary Genomics, University of Leuven, Leuven, Belgium; 3 Bioarchaeology Service, Museo delle Civiltà, Roma, Italy; 4 Dipartimento di Scienze della Terrra “A. Desio”, Università degli Studi di Milano, Milano, Italy; 5 Dipartimento di Scienze Chimiche e Geologiche, Università degli Studi di Cagliari, Monserrato, Italy; 6 Dipartimento Scienze dell’Antichità, Sapienza Università di Roma, Roma, Italy; 7 School of Geography, Archaeology and Environmental Studies, University of the Witwatersrand, Johannesburg, South Africa; Max Planck Institute for the Science of Human History, GERMANY

## Abstract

The abundant faunal remains from the Takarkori rock shelter in the Tadrart Acacus region of southwestern Libya are described. The material that covers the period between 10,200 to 4650 years cal BP illustrates the more humid environmental conditions in the Central Sahara during early and middle Holocene times. Particular attention is focussed on the aquatic fauna that shows marked diachronic changes related to increasing aridification. This is reflected in the decreasing amount of fish remains compared to mammals and, within the fish fauna, by changes through time in the proportion of the species and by a reduction of fish size. The aquatic fauna can, in addition, be used to formulate hypotheses about the former palaeohydrographical network. This is done by considering the possible location of pre-Holocene relic populations combined with observations on the topography and palaeohydrological settings of the Central Sahara.

## Introduction

The late Quaternary palaeoecological reconstruction in desert environments is often limited by a number of factors including the lack of continuous and well-preserved archives for proxy data. For instance, the present-day, strong erosion removes sediments and destroys open air palaeontological assemblages, thus limiting the comprehension of past ecological settings. However, late Pleistocene and Holocene archaeological caves and rock shelters offer the opportunity to investigate–besides cultural aspects–well-dated archives preserving information on the composition of past faunal and plant communities. Archaeozoology of desert sheltered sites is a precious tool to reconstruct late Quaternary ecological changes especially when aquatic species are considered, thus providing information on climatic and environmental changes.

Archaeozoological studies on early Holocene sites are rare in Libya: besides the work carried out in Haua Fteah and some other late Quaternary sites excavated in Cyrenaica [1], and the analysis of surface material collected in the area between the Edeyen of Murzuq and the Kufra basin [2], faunal data are mainly available from excavations in the Tadrart Acacus massif and the Messak plateau.

The fauna discovered during the excavations in the late 1950s and early 1960s in the rock shelter at Uan Muhuggiag was first briefly reported in Pasa and Pasa-Durante [3] and Mori [4] and then restudied by Gautier [5]. Archaeozoological material was also found in three rock shelters about 50 km north of Uan Muhuggiag during excavations carried out in the wadi Ti-n-Torha [6, 7]. The fauna found at Ti-n-Torha East and Ti-n-Torha North was first analysed by Cassoli and Durante [8] and later restudied by Gautier and Van Neer [9]. In the latter publication also the fauna from the third rock shelter–Ti-n-Torha Two Caves–was described. Further data are from early and middle Holocene Acacus sites, such as Uan Afuda, Uan Tabu, Uan Telokat and again Uan Muhuggiag: together with few data from surface sites, this corpus was published by Corridi [10]. Middle Holocene faunal remains from the Messak region have been studied by Alhaique and were published more recently [11]. A short note on the molluscs was done by Girod [12].

**Fig 1:**
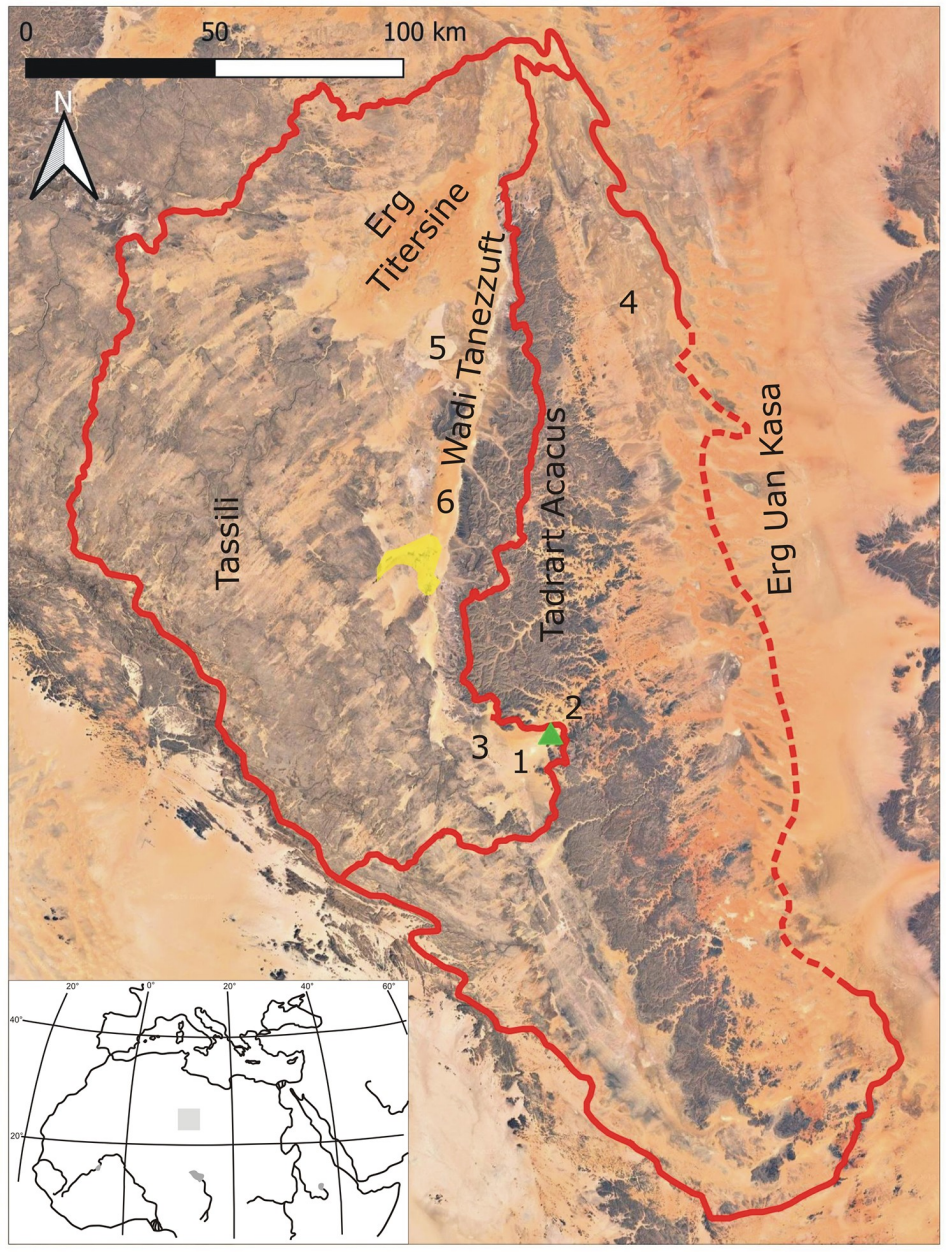
Landsat satellite imagery of the study region indicating the main localities cited in the text and reporting the limits of the two drainage basins considered (dashed line indicates an uncertain limit of the basin); the triangle indicates the position of the Takarkori rock shelter and the yellow shadow the Ghat/el Barkat/Fewet oases system. Numbers relate to former wetlands: 1) Takarkori Lake; 2) Bubu Lake; 3) Wadi Takarkori; 4) Awiss marshes; 5) Garat Ouda Lake; 6) Erg Tanezzuft.

In this paper, we present the faunal remains excavated at Takarkori (2003–2006), a rock shelter in the Tadrart Acacus, close to the border with Algeria (Fig 1). The site hosts a rich and well-preserved stratigraphic sequence, dated between 10,200 to 4650 years cal BP [13]. The size of the excavated area (ca. 145 m^2^) and the sampling strategies allowed us to collect an extensive faunal assemblage that offers a unique opportunity to reconstruct the role that animals played in the subsistence during large part of the Holocene. In addition, the material allows documenting climatic and palaeoenvironmental changes, which is the focus of this paper. Terrestrial animals will only be briefly mentioned and emphasis will be on the aquatic fauna that includes a number of taxa that can only propagate efficiently through water ways (fish, aquatic turtle, and crocodile). The material therefore has the potential of contributing also to a better understanding of the former palaeohydrographic network of the Sahara and its connection with the Nile Valley and the Sahel.

## Geomorphology and environmental conditions

### Geographic, climatic and geologic context of the Tadrart Acacus

The Tadrart Acacus is located in the Fezzan region of Saharan southwestern Libya. It is roughly included in the area between 26° and 24° latitude N (Fig 1). The mountain covers an area of ca. 5000 km^2^ with a maximum elevation of approximately 1350 m a.s.l. The rocks outcropping in the region are Lower to Middle Silurian to Lower Devonian shales and sandstones [14, 15]. The massif is a monocline gently tilted toward E-NE, and cut by a fossil drainage network [16, 17]. A sharp scarp delimits the massif towards the west, while eastwards it progressively merges below the dune fields of the Erg Uan Kasa. Late Quaternary deposits consist of fluviatile sand and silt and loamy-clay lacustrine to pond sediments, accumulated during wet phases, and sand sheets and dunes formed in arid phases thanks to enhanced wind activity [18, 17]. The present climate of the Libyan central Sahara is hyperarid and linked to low altitude pressure and winds over North Africa [19]. Scarce meteorological data are available for the Tadrart Acacus massif: the mean annual temperature is between 25° and 30°C and the mean annual rainfall is between 0 and 20 mm [20]. Several continental palaeohydrogeological archives suggest that during the Holocene environmental conditions were more humid than today. The northward expansion of the southwest African monsoon in the early to middle Holocene–the so called African Humid Period (AHP)–brought high rainfall to the central Sahara [21], thus recharging local aquifers [22, 17]. The AHP was interrupted by transitory dry spells [23, 24], and its termination occurred since ca. 5500 years cal. BP as a consequence of the progressive reduction in the intensity of the African monsoon [25]. The termination of the AHP in the Libyan central Sahara showed different timing according to specific physiographic units [25]. Landscape units connected to larger water reservoirs resisted for a longer period to aridification, whereas wetlands sustained by surface aquifers dried up quickly.

### Geomorphological outlines of the Takarkori area

**Fig 2:**
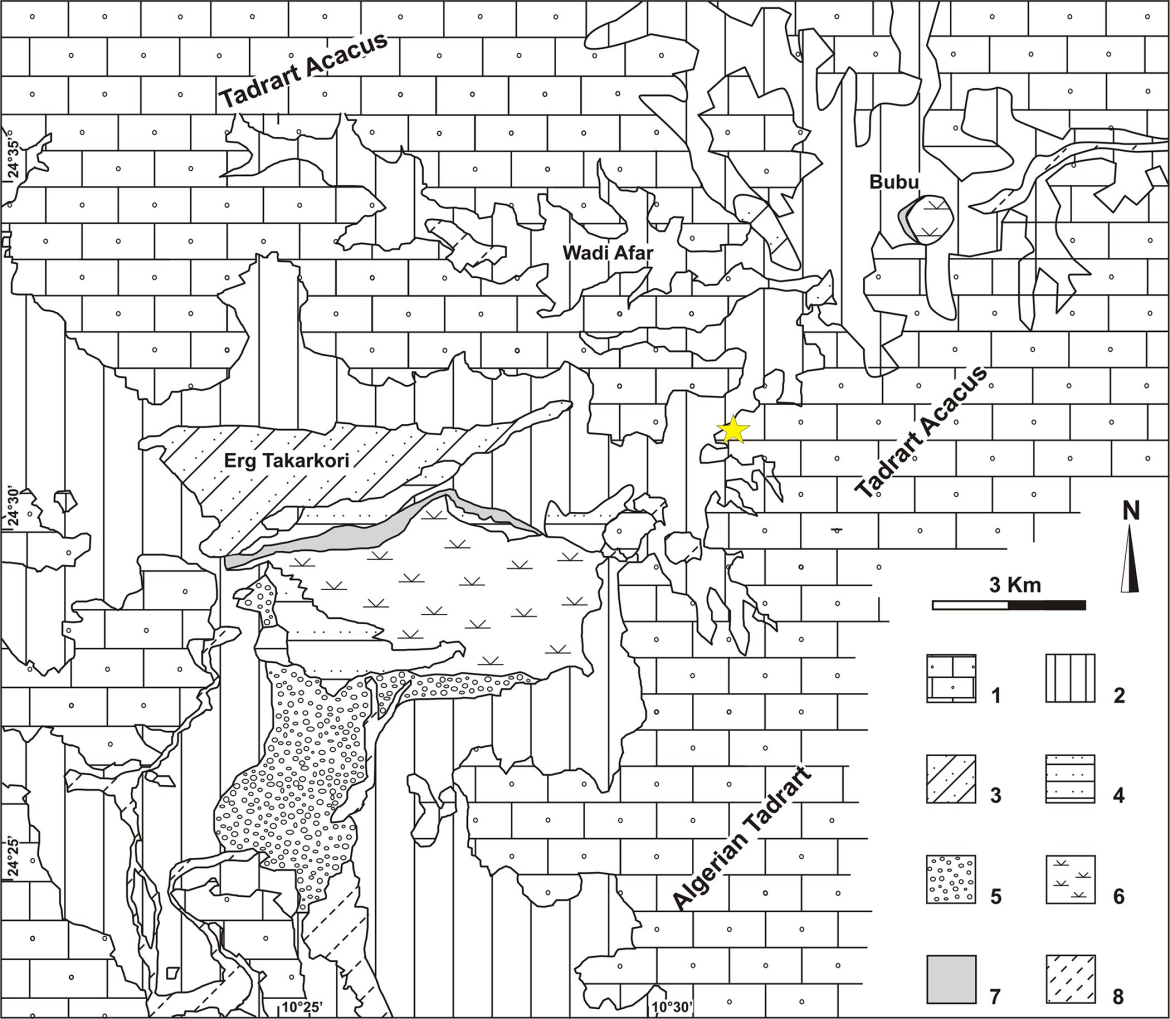
Geomorphological map of the Takarkori region (modified from Cremaschi et al., 2014); the star indicates the position of the rock shelter. Key: 1) bedrock (sandstone and siltstone, Paleozoic); 2) pediment and slope deposits (Neogene/Quaternary); 3) red dunes (Pleistocene); 4) yellow dunes (Holocene); 5) fluvial gravel (Holocene); 6) lake sediments (Early/Middle Holocene); 7) organic shore deposits (Early/Middle Holocene); 8) recent alluvium (Late Holocene).

The Takarkori rock shelter is located on Wadi Takarkori, a large valley separating the Tadrart Acacus massif in Libya from the Algerian Tadrart (Fig 1) [13]. This area also corresponds to the watershed dividing the Wadi Tanezzuft basin (to the west) from the Tadrart Acacus-Erg Uan Kasa basin (to the east). The southern side of the Tadrart Acacus massif consists of a large slope, connecting the flat area of the upper valley of the Wadi Tanezzuft to the outcrops of the Acacus sandstone. Fig 2 offers a simplified geomorphological representation of the area of Wadi Takarkori, illustrating the main units related to early Quaternary to Holocene surface processes. The bedrock consists of sandstone coated by Holocene Mn-bearing rock varnish [26]. Slope landforms include the alluvial apron at the base of the escarpment, the glacis, and deposits of heterometric angular clasts connecting the bedrock outcrops to the wadi bottoms. The Erg Takarkori is the largest dune system of the area, consisting of Pleistocene coalescent star and dome dunes [22]. Smaller alignments of coalescent barchans were accumulated after the deflation of early-mid-Holocene deposits. A deposit of bluish grey, olive, and black, loamy and clayey sand, including a rich fauna of fresh-water mollusc, outcrops in the most depressed part of the basin. This deposit formed in water environment (lake to swamp). A greyish-black, organic matter rich sand is located at the margin of swamps, corresponding to the shoreline of the former ponds. Fluvial sediments (gravel bars up to fine-textured sediments) are also present, and they are the results of early to middle Holocene sedimentation.

### Former wetlands in the vicinity of Wadi Takarkori

**Fig 3:**
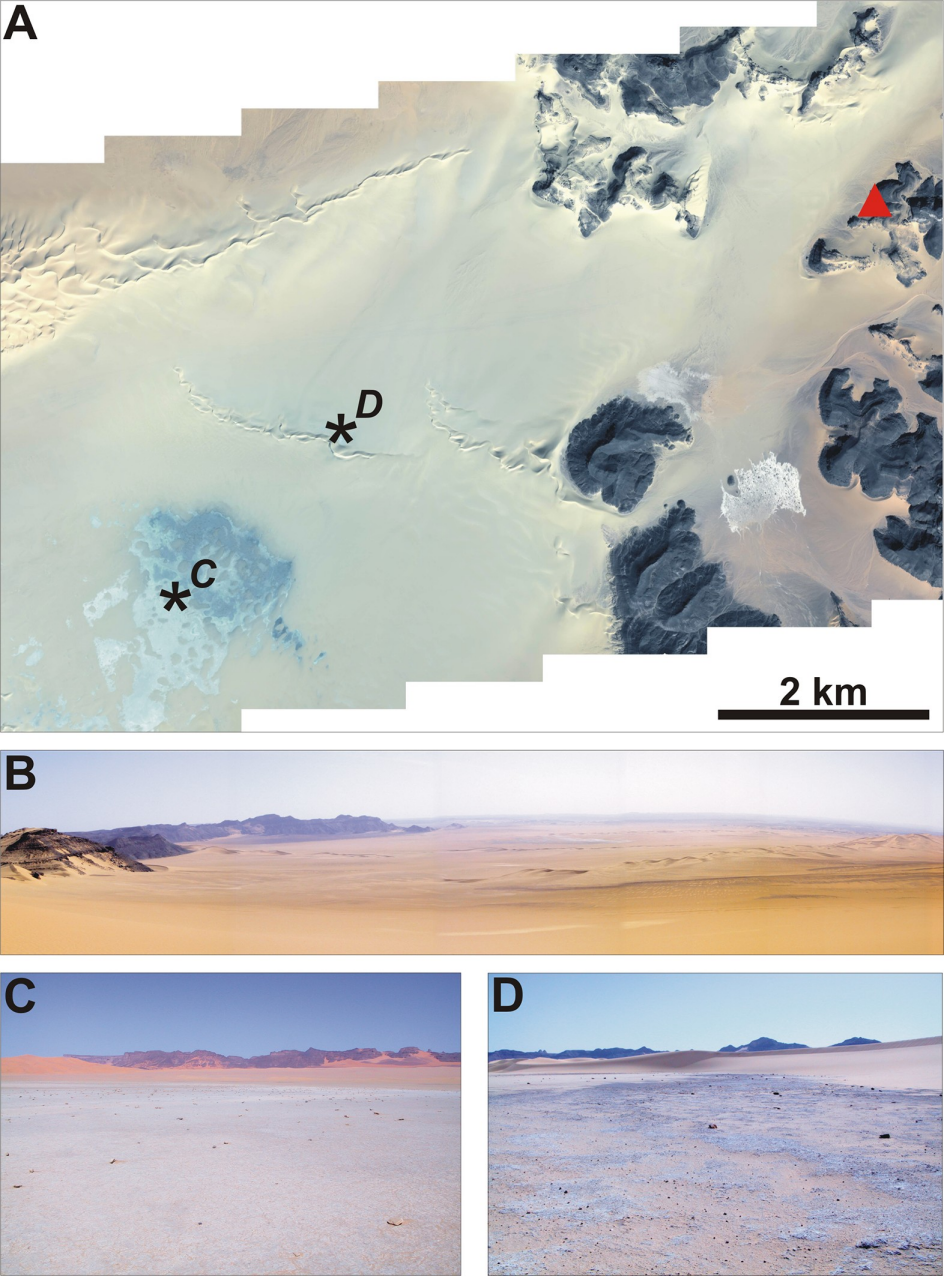
(A) Ikonos satellite imagery of the Takarkori region, the bluish to greyish surfaces are the former lake; the triangle indicates the Takarkori rock shelter; the position of field pictures C and D is also reported. (B) General view of the former Takarkori Lake from the Tadrart Acacus. (C) Field picture at the depocentre of the lake. (D) Field picture at the eastern margin of the lake.

**Fig 4:**
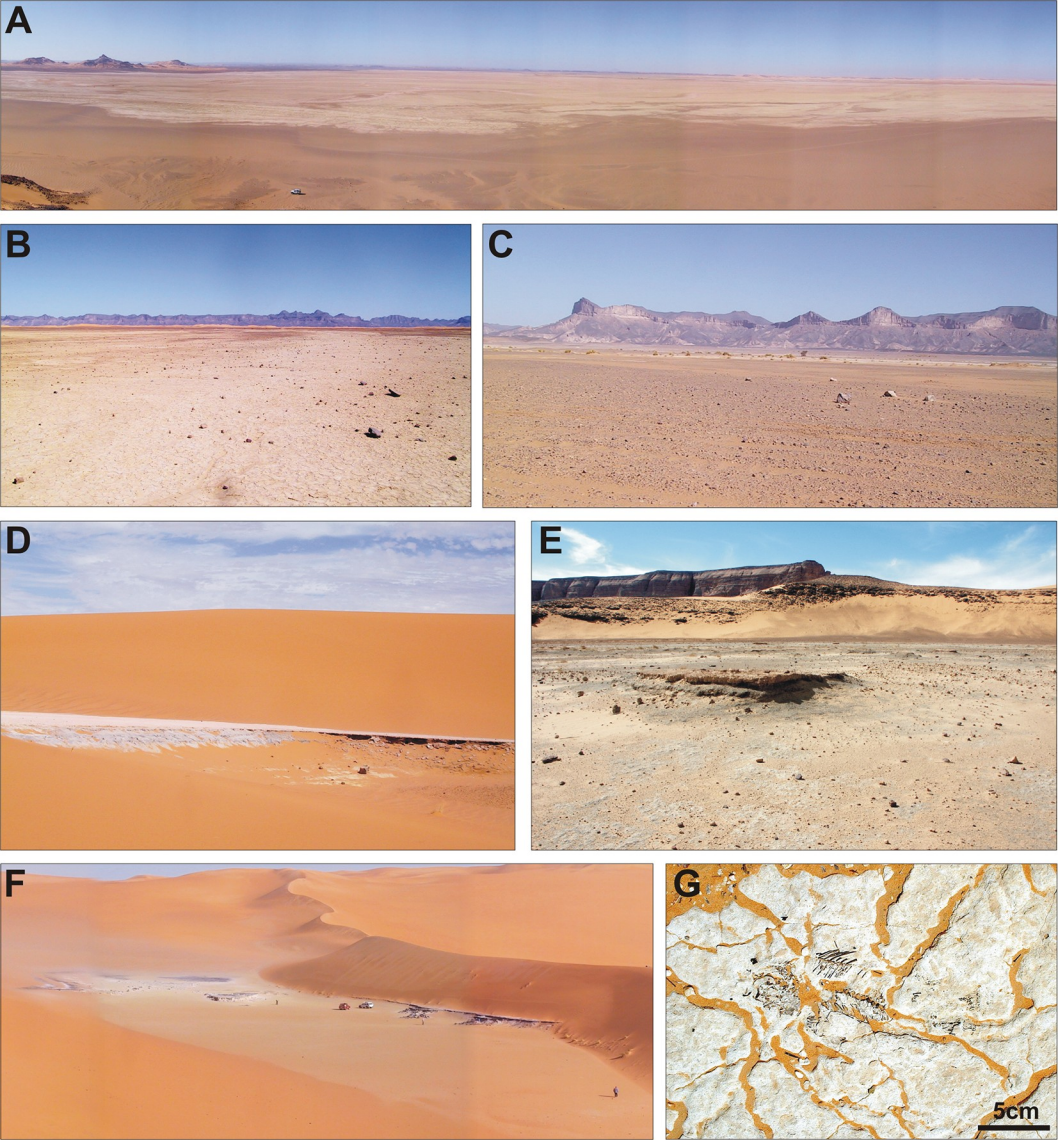
Field pictures of former wetlands in the study region. (A) General view of the Garat Ouda Lake. (B) A detail of the Garat Ouda Lake. (C) Early Holocene fluvial sediments along the Wadi Tanezzuft. (D) Carbonatic lake muds in a former basin between the dunes of the Erg Titersine. (E) General view of the Bubu basin. (F) Holocene lacustrine formation among the dunes of the Erg Uan Kasa. (G) Example of a subfossil, articulated skeleton of a tilapia inside the lake sediments of the Erg Uan Kasa.

If we consider the Tadrart Acacus massif and adjoining regions, we can identify several former wetlands that were active during the AHP, and that are today desiccated (Figs 3 and 4). The region can be divided into two major hydrographic basins, divided by a watershed roughly corresponding to the western escarpment of the Tadrart Acacus massif (Fig 1). The Tadrart Acacus-Erg Uan Kasa system, which includes the massif and its ancestral hydrographic net and the dune system of the Erg Uan Kasa [17], is one of the two drainage basins. In the erg, geomorphological and sedimentary evidence indicates the occurrence of lakes (Fig 4) between and at the margin of the dunes of the Erg Uan Kasa, radiocarbon dated between the early and middle Holocene [27, 21]. Extensive marsh sediments, mostly dating to the middle Holocene and to later phases are distributed at the northeastern margin of the Tadrart Acacus [17]. Swamp sediments similar to those found in the Wadi Takarkori region are present in the Bubu area, east of the Takarkori rock shelter. In the early and middle Holocene, wadis cutting the Tadrart Acacus massif were crossed by rainfall-sustained permanent to seasonal rivers. Extensive fluvial sediments and pollen data suggest the existence of rivers [28]. Today, wadis are only occasionally flooded favouring the formation of occasional ponds in a few, depressed areas [29, 30].

The other hydrographic basin is limited by the western escarpment of the Tadrart Acacus and encompasses the Wadi Tanezzuft valley, the Wadi Takarkori area, and large part of the Tassili plateau. During the AHP a large aquifer sustained the Tanezzuft River (Fig 4), flowing for ca. 200 km from South to North, ending in an endorheic delta north of the Tadrart Acacus massif [25]. Surface groundwater sustained several ponds active in the wet Holocene between the dunes of Erg Titersine and Erg Tanezzuft. A lateral branch of the Tanezzuft River fed for several millennia the Garat Ouda Lake [21]. The Tanezzuft River existed for several millennia and survived the termination of AHP, diminishing progressively its length and sustaining an extensive oasis. In the mid-late Holocene, the decreased discharge of the river provoked the interruption of its connection with the Garat Ouda Lake, which dried out in a few decades. The Tanezzuft River was occasionally active also in the late Holocene. Finally, a few groundwater-fed ponds were present-day inside the oases of Ghat, el Barkat and Fewet, in a few cases active up to some decades ago [31, 32].

## Chronology and archaeological context

The first inhabitants at Takarkori rock shelter were early Holocene hunter-gatherer-fishers locally called “Late Acacus” (LA1-3: ca. 10,200–8000 cal BP). Archaeological and archaeobotanical evidence indicates prolonged, albeit seasonal, residential occupation and a delayed-return system of resource exploitation [13, 33, 34, 35]. Stone structures of different size and functions (huts, windbreaks, platforms, etc.) and large fireplaces, together with large grinding stones and abundant pottery also point to semi-sedentary lifestyle, as indicated by other coeval sites in the region, such as Ti-n-Torha East, Uan Afuda and Uan Tabu [7, 36, 37]. The earliest evidence of Pastoral Neolithic herders (EP1-2) dates to ca. 8300 cal BP, mostly represented by the burial of women and children [38]. A full pastoral economy based on cattle exploitation including dairying is attested from approximately 7100 years cal BP [39], when the shelter is occupied seasonally (MP1-2), likely from the end of the rainy season and during the dry winter [13, 40]). Nomadic herders (LP1, ca. 5900–4650 years cal BP), mostly focussing on small livestock (sheep/goat) rather than cattle, briefly camped at Takarkori during the winter, using much part of the area for penning the animals, with a thick, hardened layer of ovicaprine dung closing the sequence (Table 1).

**Table 1:** 

Phase	Sub-phase	Chronology		
		Years uncal BP	Calibrated yrs BP (95% confidence)	Calibrated yrs BC (95% confidence)
Late Pastoral (LP)	LP1	5000–4000	5900–4300	3950–2350
Middle Pastoral (MP)	MP2	5500–5000	6400–5600	4450–3700
	MP1	6100–5500	7100–6200	5200–4250
Early Pastoral (EP)	EP2	6900–6400	7800–7300	5900–5300
	EP1	7400–6900	8300–7600	6400–5700
Late Acacus (LA)	LA3	7900–7400	9000–8000	7050–6100
	LA2	8500–7900	9500–8600	7600–6650
	LA1	8900–8500	10,200–9400	8250–7500

Chronology of the main chrono-cultural phases identified at Takarkori (modified, after [42]). The calibration expresses the maximum chronological range and overlaps are statistically possible. For the calibration: OxCal online version 4.3, see [109].

## Material and methods

**Fig 5:**
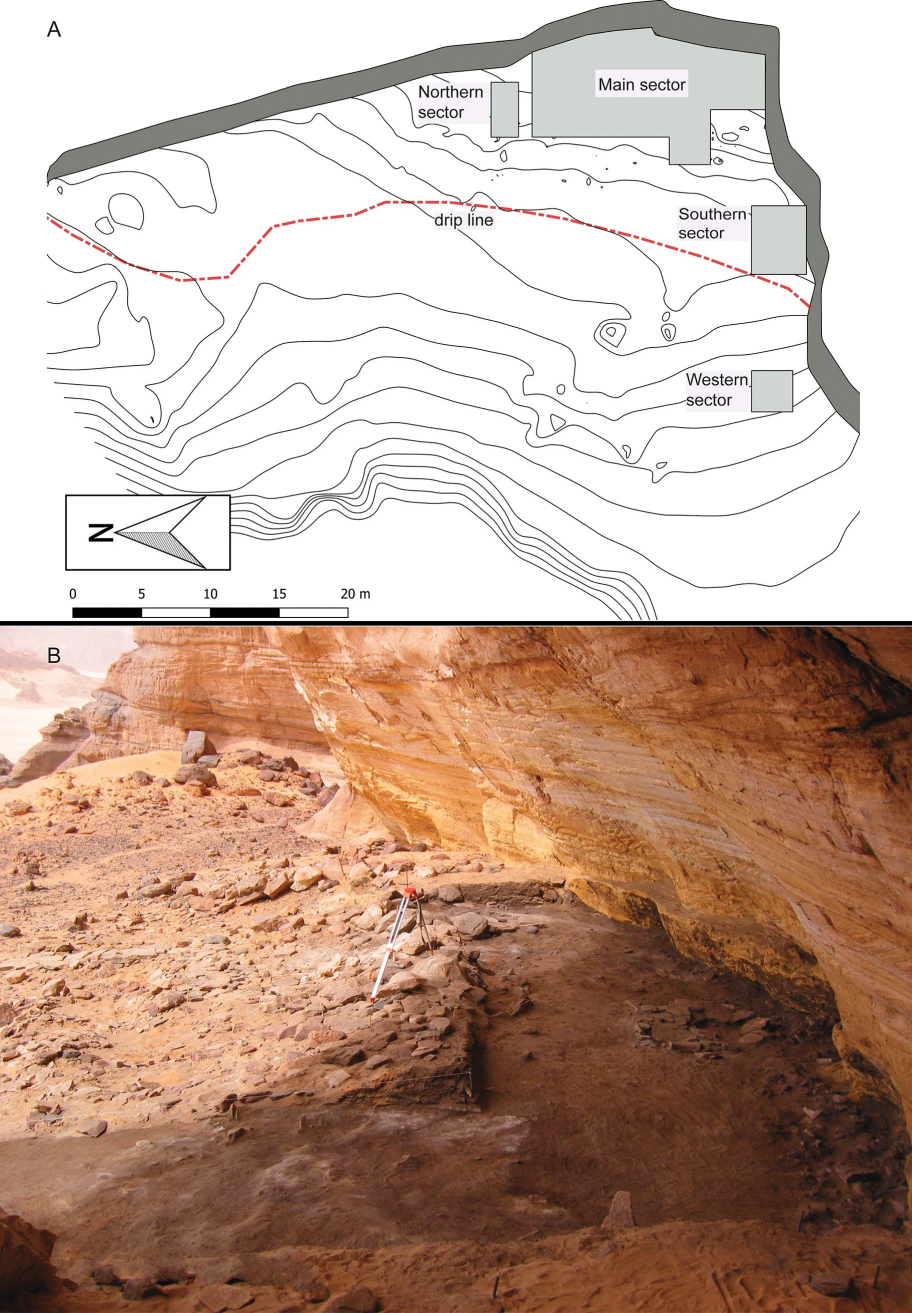
Takarkori rock shelter: (A) map of the excavated sectors and (B) view of the site from the south.

The archaeological deposit has been excavated in 4 distinct areas: Northern, Main, Western and Southern Sector (Fig 5); see [13] for details. The bedrock has been reached only in the Northern Sector, where the stratigraphy is ca. 1.6 m deep, whereas excavation in the Main Sector was blocked at the Late Acacus 2 level, in order to create an open air museum to preserve the exceptional early Holocene features–a project that was stopped by the civil war (2011). As many stratigraphic sheltered contexts in the central Sahara and in the Acacus in particular, most of the deposit at Takarkori is made of loose “organic sands” [41, 13]. Clear and sharp stratigraphic transitions are rare mostly due to the presence of fireplaces, stone structures, and hardened dung layers. The repeated use of the shelter generated complex sin- and post-depositional phenomena (e.g., human and animal trampling, moving of stone slabs, digging of burials, etc.) that strongly affected the “original” spatial configuration, both vertical and horizontal. This might explain the presence of intrusive elements in several layers, in particular in the Late Acacus 2 and 3, as well as in the early-middle Pastoral phases. Clearly recognizable items, such as decorated pottery, can be easily distinguished, whereas faunal remains, also due to their small size (and often poor state of preservation) could be more elusive. In some cases, specific elements have been directly radiocarbon dated to test their stratigraphic attribution [42].

Evidence of former wetlands in the Libyan central Sahara has been identified in remote sensing and explored in the field [17]. The reconstruction of the main Saharan and North African hydrographic networks was elaborated from existing databases and satellite image photointerpretation. The principal current water basins are derived from HydroSHEDs [43]. The main Early Holocene waterways and the past extension of Lake Chad were reconstructed by photointerpretation of satellite imagery in natural and false colours (LANDSAT and Google Satellite); the reconstruction of Lake Chad also profited of data published by Armitage and colleagues [44]. Potential past water courses were validated with the help of a global 30 arc-second DEM [45]. This permitted to identify and correct misleading interpretations of fake water courses suggested by automated algorithms, as for instance, linear interdune corridors automatically attributed to potential palaeochannels [46].

The faunal remains that are presented here were recovered during the excavations by the systematic dry sieving of all the sediment on a 4 mm mesh. All the material was shipped to Italy and then Belgium for analysis with all the necessary formal permits granted by the Libyan Department of Antiquities to the mission of Sapienza University of Rome. The material is temporarily stored in the Sapienza University, in order to be resent to the Lybian Department of Antiquities, Tripoli, Libya, as soon as safety conditions in Libya will recover. Identification of the mammal bone was carried out with the aid of the comparative collections of modern skeletons housed at the Istituto Italiano di Paleontologia Umana (IsIPU) in Rome and at the Museo di Storia Naturale La Specola in Florence. The bird bones were partly identified using the IsIPU collections and those of the Museo Preistorico Etnografico "L. Pigorini" in Rome, now part of the Museo delle Civiltà, and partly at the Royal Belgian Institute of Natural Sciences in Brussels. It is also in the latter institute that adequate collections were available to carry out the identifications of the molluscs, fish, amphibians, and reptiles.

Identifications were based on both morphological and metrical criteria and included the recording of the skeletal element and the taxon. In the case of fish also a reconstruction of the body length was carried out. For well-preserved bones the corresponding fish lengths could be calculated using published regression formulae [47]. In case the bones were too broken to allow taking measurements, fish length–in 10 cm length classes–was estimated by direct comparison with modern specimens of known size. All the fauna was quantified using number of identified specimens (NISP). The numerous unidentified specimens were counted as well in the case of the mammals and birds. Among the fish remains there were hardly any unidentified bones, which may seem remarkable at first sight. However, this is due to the fact that the only two taxa that occur–tilapia and clariid catfish–are so different in morphology and texture of the bones that they can be identified easily even in case where the skeletal element is no longer recognisable.

Modifications that could be observed on the bone, due to either natural or anthropogenic factors, were noted as well. These include observations on the weathering of the bones, gnaw marks caused by rodents and carnivores, cut marks and traces of burning. These data, combined with the skeletal element representation of each of the taxa, and in some cases also the behaviour of the species, are used to reconstruct the taphonomic history of the finds and to assign the remains as much as possible to so-called taphonomic groups [48].

## Results

### The species spectrum and main taphonomic groups

An overview of all the fauna recorded from Takarkori is provided in Table 2. It is clear that fish remains are the major find category (see S1 Table for details on the skeletal elements by which the different taxa are represented, and for fish length reconstructions). Even when taking into account the large amount of unidentified mammal and bird bones, the fish represent 79.5% of the entire find collection that consists of 17,551 remains. The mammal and bird bones (including the unidentified ones) represent 19.1% and 1.1% of the total collection. The contribution of molluscs (0.03%), amphibians (0.02%) and reptiles (0.21%) is negligible. In the paragraphs below we describe the species that were found with a special focus on the ones that are of relevance for the reconstruction of the aquatic environment and the palaeohydrography during the early and middle Holocene. All of the fish and most of the mammals, bird and reptile remains are considered as human consumption refuse, sensu Gautier [48]. The small molluscs are all intrusive animals that were penecontemporanous to the human habitation, and the same is probably the case for the anurans (toad or frog), the agama and small snake. As will be discussed further, it is in some cases not clear if the animals were deposited as a result of natural or anthropogenic factors. Whereas this can hamper the palaeo-economical interpretation, it is not a problem for the palaeo-ecological analysis that is undertaken here. There is no evidence at the site for recent intrusives, i.e. modern animals that would have burried into the archaeological layers, and there is no evidence either for older, so-called geological intrusives. All the taxa that we have at our disposal are contemporaneous to the human occupation and can thus safely be used for the environmental reconstruction at the time Takarkori was inhabited by humans.

**Table 2:** 

CHRONOLOGICAL PHASE	LA1	LA2	LA3	EP1	EP2	MP1	MP2	LP	?	Total
**MOLLUSCA**										
*Corbicula consobrina*	-	-	-	-	-	-	1	-	-	1
*Melanoides tuberculata*	-	-	2	-	-	-	-	-	1	3
*Zootecus insularis*	-	-	-	1	-	-	-	-	-	1
unidentified terrestrial gastropod	-	-	1	-	-	-	-	-	1	2
**PISCES**										
*Clarias gariepinus* (catfish)	227	2401	3025	1220	769	294	1095	236	NC*	9267
*Oreochromis niloticus* (Nile tilapia)	9	98	37	20	27	9	24	4	NC	228
*Coptodon zillii* (redbelly tilapia)	1	5	3	3	9	-	8	-	NC	29
Haplotilapiini indet. (tilapia)	186	1575	961	536	503	95	519	54	NC	4429
**AMPHIBIA**										
*Bufo* sp. (toad)	-	1	1	-	-	-	-	-	-	2
Anura indet. (toad or frog)	1	-	-	-	-	-	-	-	-	1
**REPTILIA**										
cf. *Agama* (agama)	-	1	-	-	-	-	-	-	-	1
*Crocodylus niloticus* (crocodile)	-	-	-	6	-	-	2	1	5	14
*Pelusios adansoni* (Adanson’s mud turtle)	-	-	-	1	3	-	4	-	-	8
unidentified snake	-	2	3	2	2	-	3	1	-	13
**AVES**										
*Aythya* cf. *fuligula* (cf. tufted duck)	-	1	-	-	-	-	-	-	-	1
*Podiceps cristatus* (great crested grebe)	-	4	1	-	-	-	-	-	-	5
*Pelecanus rufescens* (pink-backed pelican)	-	-	-	1	-	1	-	-	-	2
*Pelecanus onocrotalus* (great white pelican)	-	-	1	-	-	-	-	-	1	2
*Pelecanus* sp. (pelican)	1	1	1	3	2	1	-	-	1	10
*Microcarbo africanus* (long-tailed cormorant)	-	2	1	-	-	-	-	-	-	3
*Phalacrocorax* cf. *carbo* (cf. great cormorant)	-	1	-	-	-	-	-	-	-	1
*Phalacrocorax* sp. (cormorant)	-	-	-	1	-	-	-	-	-	1
*Plegadis falcinellus* (glossy ibis)	-	-	-	1	-	-	-	-	-	1
*Plectropterus gambensis* (spur-winged goose)	-	-	-	-	1	1	-	-	-	2
*Pernis apivorus* (European honey-buzzard)	-	-	1	-	-	-	-	-	-	1
*Buteo* sp. (buzzard)	-	-	-	-	-	-	-	-	1	1
*Circaetus* sp. (snake-eagle)	-	7	2	-	-	-	-	-	-	9
Accipitridae size *Milvus* sp. (cf. kite)	-	-	2	1	1	-	-	-	-	4
Accipitridae size *Circus aeruginosus* (cf. western marsh-harrier)	-	1	-	-	-	-	-	-	-	1
Accipitridae size *Circaetus* sp. (cf. snake-eagle)	1	5	8	2	-	-	-	-	-	16
Accipitridae indet. (raptors)	-	6	6	2	-	-	2	-	3	19
*Falco* cf. *subbuteo* (cf. Eurasian hobby)	-	1	-	-	-	-	-	-	-	1
*Fulica atra* (common coot)	2	1	1	1	-	-	1	-	1	7
*Gallinula chloropus* (common moorhen)	-	-	1	-	-	-	-	-	-	1
Otididae size *Ardeotis kori* (large bustard)	-	-	2	-	2	-	-	-	-	4
Columbidae (pigeon or dove)	-	2	-	-	-	-	-	1	-	3
*Corvus* cf. *albus* (cf. pied crow)	-	-	-	-	-	-	-	-	1	1
cf. *Hirundo rustica* (cf. barn swallow)	-	-	-	-	-	-	-	1	-	1
Unidentifiable bird	4	22	18	25	8	1	12	4	4	98
**MAMMALIA**										
*Hystrix cristata* (crested porcupine)	-	-	1	-	-	-	-	-	-	1
*Thryonomys* cf. *swinderianus* (cane rat)	-	1	-	-	-	-	-	-	-	1
*Massoutiera mzabi* (Mzab gundi)	-	-	2	-	3	-	4	-	3	12
cf. *Xerus erythropus* (cf. striped ground squirrel)	-	-	-	1	-	-	-	-	-	1
*Gerbillus* sp. (gerbil)	-	-	2	1	-	-	-	-	-	3
Rodentia indet. (small rodent)	-	2	2	4	5	-	6	1	2	22
*Procavia capensis* (rock dassie)	-	11	12	3	6	2	13	2	5	54
*Lepus capensis* (hare)	4	1	4	-	1	-	2	1	1	14
*Papio anubis* (olive baboon)	-	1	-	1	-	-	-	1	-	3
*Herpestes ichneumon* (Egyptian mongoose)	-	-	-	1	-	-	4	-	-	5
*Ictonyx libyca* (Libyan striped weasel)	-	-	-	-	-	-	-	-	1	1
*Felis margarita* (sand cat)	-	-	-	1	-	-	-	-	-	1
*Felis silvestris*/*margarita* (wild cat/sand cat)	-	1	-	-	-	-	-	-	-	1
*Caracal caracal* (caracal)	-	-	-	-	1	-	1	-	-	2
*Vulpes zerda* (fennec fox)	-	-	-	-	-	-	1	-	-	1
*Canis aureus* (golden jackal)	-	1	-	-	1	-	-	-	-	2
Rhinocerotidae indet. (rhinoceros)	-	1	-	-	-	-	-	-	-	1
cf. *Phacochoerus aethiopicus* (cf. warthog)	-	-	1	-	-	-	-	1	-	2
*Ammotragus lervia* (Barbary sheep)	1	21	30	13	6	-	5	-	7	83
*Gazella dorcas* (dorcas gazelle)	2	12	23	8	1	2	9	6	7	70
cf. *Nanger dama* (cf. dama gazelle)	-	2	-	1	-	-	1	-	-	4
*Ovis*/*Capra* (sheep/goat)	-	3**	2**	5	2	2	17	7	9	47
*Bos taurus* (cattle)	-	-	-	1	6	1	12	3	1	24
Large carnivore	-	-	1	-	-	-	-	-	-	1
Small bovid	4	28	62	60	40	19	85	30	47	375
Large bovid	-	9	10	22	20	6	79	17	26	189
Medium mammal	5	45	95	75	56	10	128	46	38	498
Small mammal	-	7	22	10	7	1	18	2	5	72
Unidentifiable mammal	8	85	272	237	237	52	652	189	135	1867

Overview of the faunal remains from Takarkori. Figures correspond to NISP’s (Number of identified specimens). See Table 1 for chronological phases.

* NC = not counted.

** intrusive, due to post-depositional disturbance

#### Molluscs

The mollusc fauna is limited both in number of specimens and taxa. Of the three terrestrial gastropods that were found only the one in an EP1 context was sufficiently preserved to be identifiable as *Zootecus insularis*. This is a small species that is typically found in arid and semi-arid environments of the entire Saharo-Indian region [49]. Its presence at Takarkori had already been reported by Girod [12], who also mentioned Holocene finds from other localities namely, the high Hoggar, the Tifedest region and Tin Tahart west of the Tassili N'Ajjer. In a context dating to MP2, a single shell was found of *Corbicula consobrina*. This small aquatic bivalve is widespread in the present-day Sahel and in some relic areas of the Sahara and used to occur here more frequently during the early Holocene [50]. *Corbicula consobrina* has been reported in Libya from localities near Brak [51] and Tejerhi [52]. This freshwater species can support relatively high salinity levels and thanks to this tolerance it is also found in rather brackish waters [53]. The same is true for the gastropod *Melanoides tuberculata* of which two individuals were found in LA3. It is a panafrican species of which living relic populations can be found on many localities in the Sahara, including in Libya at Ghat [50]. In Libya, Holocene fossil finds have been reported from Tejerhi in Fezzan [52], Wadi Shati [54] and in the Kufra oasis [55]. In our study area, accumulations of *Melanoides* and *Corbicula* shells have been recurrently found in lake and fluvial sediments [27, 25]. For instance, they are abundant in the lacustrine sequences of the Erg Uan Kasa and in the area of Garat Ouda [52, 56].

#### Fish

**Fig 6:**
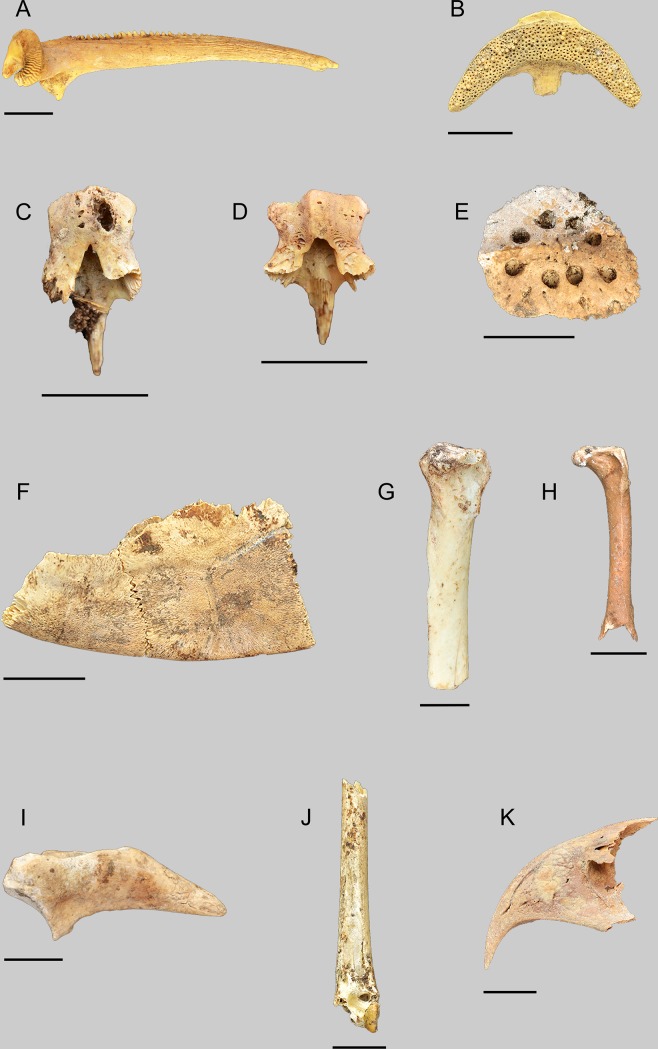
A selection of aquatic and terrestrial taxa found at Takarkori. Each scale bar is 1 cm. (A) *Clarias* sp.: dorsal view of right pectoral spine. (B) *Clarias gariepinus*: ventral view of vomerine toothplate. (C) *Tilapia zillii*: dorsal view of prevomer. (D) *Oreochromis niloticus*: dorsal view of prevomer. (E) *Crocodylus niloticus*: dorsal view of dermal scute. (F) *Pelusios adansonii*: dorsal view of carapace fragment (peripheral 7 and 8). (G) *Pelecanus rufescens*: medial view of right proximal radius. (H) *Microcarbo africanus*: cranial view of left femur. (I) *Plectropterus gambensis*: ventral view of left os carpi radiale. (J) *Plegadis falcinellus*: cranial view of left distal tibiotarsus. (K) *Circaetus* sp.: left lateral view of premaxilla.

**Fig 7:**
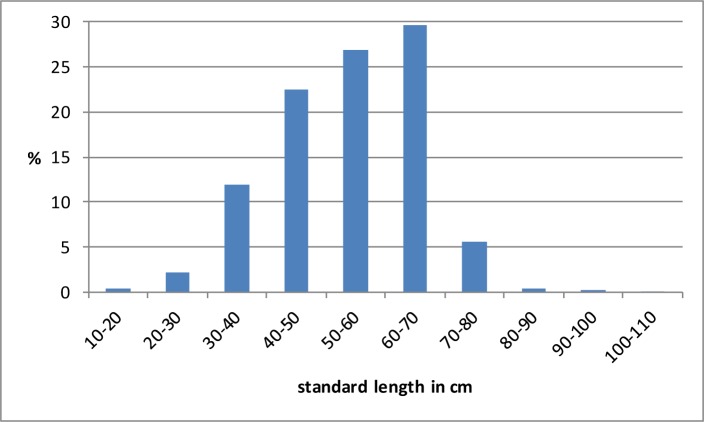
The reconstructed fish lengths of the *Clarias* catfish, presented in 10 cm size classes. Total sample size was 4505 specimens.

The fish fauna identified from Takarkori consists for two thirds (66.4%) of Clariidae catfish and the remaining third (33.6%) are tilapia (Haplotilapiini).The family of Clariidae is represented by two genera in the Nilo-Sudan ichthyofaunal province, namely *Heterobranchus* and *Clarias* [57]. Although in the present-day relic populations of fish in the Sahara only the genus *Clarias* occurs [58, 59], it cannot be excluded a priori that also the other genus may have occurred further north in the early and middle Holocene. However, it appears that none of the diagnostic bones that allow distinguishing between both genera [60, 61] are from *Heterobranchus*. All pectoral spines clearly belong to the genus *Clarias* (Fig 6A). The two Nilo-Sudanic species of *Clarias*, namely *Clarias gariepinus* and *Clarias anguillaris* occur nowadays as relic populations in the Sahara. *Clarias anguillaris* is found in the Adrar and Tagant regions of Mauritania, and in the Tassili n'Ajjer of Algeria [58]. The other species is living nowadays in various localities in Algeria (including the Tassili n'Ajjer), in Chad at the guelta of Tottous in the Tibesti, and in Libya, where it has been reported from the Lake of El Hombra, and from the Fezzan region near Ghat [31, 62]. Both *Clarias* species are difficult to distinguish on isolated bones, but the few diagnostic pieces that we had at Takarkori (vomerine toothplates; cf. Fig 6.2 in [63]) indicate the sole presence of *Clarias gariepinus* (Fig 6B). The distribution of the reconstructed fish sizes is given in Fig 7. The smaller size classes (10–20 and 20–30 cm SL) are poorly represented. Most of the catfish are in the length classes 40–50 to 60–70 cm SL. A few large specimens larger than 1 metre SL were observed.

**Fig 8:**
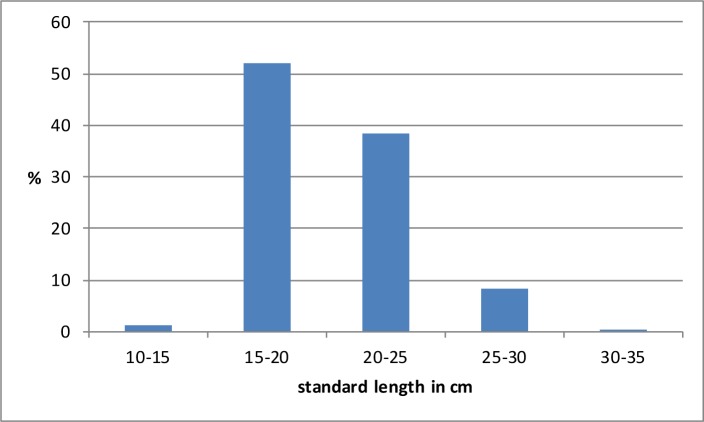
The reconstructed fish lengths of the tilapia, presented in 5 cm size classes. Total sample size was 2541 specimens.

There are 3 species of tilapia in the Nilo-Sudan ichthyofaunal province, namely *Oreochromis niloticus*, *Coptodon zillii* and *Sarotherodon galilaeus* [57]. Although at first sight they seem very similar from an osteomorphological point of view, it appears that several skeletal elements have diagnostic criteria permitting identification at species level [64]. A little more than 5% of the tilapia bones recovered from Takarkori were identifiable to species: 29 bones belong to *Coptodon zillii* (Fig 6C) and 228 bones are from *Oreochromis niloticus* (Fig 6D). Not a single bone of *Sarotherodon galilaeus* was found. These findings are somewhat surprising, as the most commonly identified species (*O*. *niloticus*) does not occur in the present-day relic waters of the Sahara [62, 58, 59, 65]. *Coptodon zillii* is the species that nowadays is found in multiple places in the Sahara, i.e. in the region of Biskra, Touggourt, Arak, Tassili n'Ajjer, Borkou, Tibesti and Ennedi, yet at Takarkori it is less frequently represented than *O*. *niloticus*. *Sarotherodon galilaeus*, of which not a single bone was found, occurs nowadays in the Adrar region of Mauritania. *Sarotherodon borkuanus*, now considered a subspecies of *S*. *galilaeus* [59] was found in the Tibesti, Ennedi and Borkou regions [66]. The length distribution of the tilapia from Takarkori is shown in Fig 8. Tilapia smaller than 15 cm SL are rare, most of the fish were 15–20 or 20–25 cm long. The tilapia larger than 25 cm SL are likely to be from *O*. *niloticus* that reaches larger sizes than *C*.*zillii*. Holocene finds of tilapia and clariid catfish have been reported regularly from Saharan sites [58]. In Libya, they were found thus far during the excavations at Ti-n-Torha [9] and they were also reported as surface finds, together with another catfish *Synodontis*, from the region of the eastern foreland of the Tadrart Acacus massif and the Edeyen of Murzuq, and from the Kufra basin [63, 2].

Ethnohistorical information, mostly based on reports from Italian expeditions in Libya in the early 1930s, provides a local background on the fish species still living in our study region. As mentioned above [31] *Clarias lazera* (synonym of *Clarias gariepinus*; called Asulmai in Tuaregh) was widespread in the Fezzan. From the oasis of el-Barkat, a few kilometers south of Ghat, the presence was reported of *Barbus deserti* (now called *Enteromius deserti*) and of *Hemichromis bimaculatus* (called Ilim sciàia in Tuaregh). No archaeozoological evidence for the presence of the latter two species was found. *Enteromius deserti* is a small cyprinid, the maximum total length of which is 9 cm, whereas *Hemichromis bimaculatus* is a cichlid with a maximum reported total length of 14 cm (common length 7.5 cm) [67]. It is unclear if their absence from the faunal assemblage of Takarkori is due to the small size, and hence to the limited food they represent, or if their bones went unnoticed because of their small size and the mesh width (4 mm) of the sieves used during the excavations. Also worth underlining is that no tilapia species were observed in the region.

#### Amphibians and reptiles

Three bones of anurans (toad or frog) were found, two of which could be identified as toad (*Bufo* sp.). Toads are terrestrial but they need water for reproduction. Amphibians are historically documented [31] in the Ghat area only and not widespread elsewhere. Besides two toad species, *Bufo regularis* and *Bufo viridis*, also two frogs *Rana zavattarii* now known as *Pelophylax saharicus* and *Rana occipitalis* now synonymized with *Hoplobatrachus occipitalis* have been mentioned [31]. Among the reptile remains, a lower jaw that might belong to an agama was found. Another terrestrial taxon is the unidentified snake that is represented by vertebrae. Two aquatic taxa are found as well among the reptiles, namely the crocodile (*Crocodylus niloticus*) and Adanson’s mud turtle (*Pelusios adansonii*). The crocodile remains are mainly dermal scutes (Fig 6E), but the few cranial remains that were identified allow estimating the total length of the corresponding individuals. It appears that these lengths vary between about 70–80 and 100–120 centimetres. This is in agreement with the general observation that crocodiles from the Sahara do not grow larger than 2 metres [62]. A recent detailed compilation of data on the modern, sub-recent and ancient distribution of the crocodile [68] shows that the species must have widespread in northern Africa during humid periods of the Holocene. The same study reports that crocodiles are still surviving today in southern Mauritania, the Ennedi, and possibly also in the Tibesti region and that fossil finds attesting the wider former distribution are known from a large number of localities, including the Hoggar and Tassili n'Ajjer in southern Algeria and in Libya, where they have been reported from Djebel Akhdar, far north in present-day Cyrenaica, and In Habeter in the neighbouring Messak plateau.

All eight turtle remains that were found are carapace fragments, i.e. three peripherals and five pleurals. They all have a very smooth surface and are relatively thin, thus excluding that they are from tortoises (*Testud o*/*Geochelone*). One of the remains, consisting of two articulating peripherals (7 and 8), fits perfectly the *Pelusios adansonii* from the modern reference collection (Fig 6F). Among the other specimens are at least two pleurals that are narrow and that seem to fit the same species. The remaining specimens are rather fragmented and do not allow a precise identification, but they were also attributed to *P*. *adansonii*. This species was also reported from Holocene deposits in the Taoudenni area of Mali [69], and from contexts dating to the last Pleistocene interglacial at Bir Tarfawi, Egypt [70]. Nowadays *P*. *adansonii* is found in most major river basins of the Sahelo-Sudanese belt from Senegal to Sudan [71]. Of all *Pelusios* species, it is the best adapted to the desertification of the Sahara [69].

#### Birds

The bird fauna identified at Takarkori consists of 21 taxa, 10 of which are typically associated with aquatic environments. The corvid species, possibly the pied crow (*Corvus* cf. *albus*) and the buzzard (*Buteo* sp.) may have been attracted by the food waste left by the human occupants but it is unclear if they died naturally or if they were killed on purpose and eventually eaten. Birds that must have been typically attracted by the area of Takarkori are the piscivorous taxa. These include two species of pelican identified based on size [72], the pink-backed pelican (*Pelecanus rufescens*) (Fig 6G) and the great white pelican (*Pelecanus onocrotalus*) and at least two cormorant species: a larger species, probably the great cormorant (*Phalacrocorax carbo*), and the long-tailed cormorant (*Microcarbo africanus*) (Fig 6H), a smaller species that nowadays has a wide distribution south of the Sahara [73, 74]. It is unlikely that the smaller cormorant remains are from the pygmy cormorant (*Microcarbo pygmaeus*), a species that in recent times occurred very sporadically in Africa, but that nested in Algeria during the 19th century [75]. Although the latter species is close in size to *M*. *africanus*, a femur from Takarkori fits well the measurements of the modern *M*. *africanus* and of the Middle Palaeolithic specimens of Bir Tarfawi (Egypt), attributed to that species [76]. The pink-backed pelican also has a wide subsaharan distribution and occurs as well in the south-western part of the Arabian Peninsula, it is entirely piscivorous [77]. In relatively small waterbodies the long-tailed cormorant can take frogs, crustaceans and aquatic insects, but usually its diet is dominated by fish of up to 20 cm long, especially tilapia and other cichlids [73, 78]. The great cormorant also has a diverse diet with again a preference for fish up to 15 cm [78] while the great crested grebe has a diet that consists mainly of large fish up to 20 cm length [78]. Besides, there are still a number of birds with a varied diet that can take fish to some extent, these include the coot (*Fulica atra*), the common moorhen (*Gallinula chloropus*), the spur-winged goose (*Plectropterus gambensis*) (Fig 6I), the tufted duck (*Aythya fuligula*) and the glossy ibis (*Plegadis falcinellus*) (Fig 6J) [79, 78, 80, 81, 82]. The remains of snake-eagle (*Circaetus* sp.) (Fig 6K) come from an unidentified species. No snake-eagle is known to nest in the region today but the Beaudouin’s snake-eagle (*C*. *beaudouini*), the brown snake-eagle (*C*. *cinereus*) and the Western banded snake-eagle (*C*. *cinerascens*) breed directly south to the Sahara, frequently near water, while the short-toed snake eagle (*C*. *gallicus*) occurs as a migrant. All of them are specialized in catching reptiles but they can also eat rodents, amphibia and sometimes fish (*C*. *cinerascens*). Other birds of prey that were identified feed on flying insects and sometimes birds (hobby *Falco* cf. *subbuteo*) or on invertebrates or small vertebrates (European honey-buzzard *Pernis apivorus*). Although these species did not feed on the abundant fish or other aquatic animals living in the area, it is likely that they were attracted by the relatively abundant terrestrial prey animals that were present near the site. Also the bustards, species that prefer open dry grassland, may have found a favourable environment near Takarkori.

It is unclear if the bones of the birds of prey should be seen as parts of carcasses of animals that died naturally on the spot or near the place where the bones were found. Some of the species may have nested on the rocky outcrops of the site, like the buzzard or on trees nearby such as the snake-eagle. Alternatively, these and other raptors may have been killed intentionally by humans either because they were seen as a nuisance, or because they were used for their feathers (as indicated by rock art in the Acacus region; see [83]) and/or their meat. The abundance of remains of raptors (Accipitridae), most of which do not nest on cliffs, is an indication of a specific hunt by humans. In terms of meat yield, several of the other bird species may have been attractive such as the bustards, the pelicans and the spur-winged goose. The grebes, duck, coot and the cormorants were somewhat smaller but may also have served as food.

Some of the bird species encountered at Takarkori have been attested previously in Saharan sites. The great crested grebe (*Podiceps cristatus*) was reported from surface material collected in the Edeyen of Murzuq and dated to the first half of the Holocene [2], and the spur-winged goose (*Plectropterus gambensis*) has been found in a mid-Holocene site in the Abu Tabari region, lower Wadi Howar [84]. At the last interglacial site of Bir Tarfawi [76] the presence was attested of glossy ibis (*Plegadis falcinellus*), of corvids (*Corvus albus* and an unidentified large *Corvus* sp.) and of the long-tailed cormorant (*Microcarbo africanus*), which was the most abundant species. The brown snake-eagle (*Circaetus cinereus*), the long-legged buzzard (*Buteo rufinus*), the rock dove (*Columba livia*) and the brown-necked raven (*Corvus ruficollis*) were identified at Ti-N-Torha East, Tadrart Acacus and dated to the early Holocene [63].

Birds are represented both by aquatic taxa (n = 10) and land birds (n = 11), the latter being the most numerous in terms of number of bones. Among the land birds, raptors are dominant with 52 out of 61 remains. This overrepresentation likely originates from human activities, as the taxa identified with the possible exception of the buzzards, are no rock dwellers and are hence unlikely to accumulate in the rock shelter naturally. The land bird taxa, in particular the snake-eagles and the bustards are typical of open woodlands or savannah.

The aquatic bird taxa identified do not reveal much about the water system as most of them occur in very variable habitats, although the long-tailed cormorant prefers waterbodies with riparian vegetation [78]. Pelicans nest on trees, generally on the water shore and can be indifferent to human presence [77]. Among the pelican remains is a bone from an immature bird indicating local breeding in the region. The long-tailed cormorant [74] and the spur-winged goose [85] use to perch in scattered trees. Together with the pink-backed pelican [77] and the coot [82], they are frequently found in seasonal wetlands. The spur-winged goose prefers water bodies surrounded by open landscapes, such as grassland [77]. Overall, the avifauna indicates an environment with large waterbodies, marshes or ponds, even temporary, bordered by some riverine vegetation, such as reed beds and scattered trees, surrounded by savannah and perhaps grasslands.

From a zoogeographical point of view, the presence in the Sahara of afro-tropical or Sahelian bird species occurring nowadays in sub-Saharan regions was highlighted at several locations such as Ti-n-Torha [9], Edeyen of Murzuq [2] or further to the east, at Bir Tarfawi [76] and in the Jebel Tageru/Meidob hills [2]. At Takarkori, those species are the pink-backed pelican, the long-tailed cormorant and the spur-winged goose. They are species typically making seasonal movements mainly in response to the local water conditions, sometimes over very long distances [73, 78, 86, 87, 88]. The remains of snake-eagle identified at Takarkori represent another potential sub-Saharan species as it is likely either the Beaudouin’s snake-eagle (*C*. *beaudouini*), which breeds occasionally in northern Niger and north-eastern Mali [89] or the brown snake-eagle (*C*. *cinereus*), which nests nowadays in the south of Mauritania and eastern Senegambia.

#### Mammals

The mammalian fauna does not contribute much to the reconstruction of the aquatic environment, however its inclusion in this paper is useful because it allows to evidence the changes through time in the proportions of fish versus mammals, besides providing further, although general, information about the climatic conditions around the site. Several wild mammal species encountered at Takarkori still survive in the central Sahara and some of them still occur in the Acacus itself such as the Barbary sheep (*Ammotragus lervia*), dorcas gazelle (*Gazella dorcas*), and rock dassie (*Procavia capensis*). Others live nowadays south (and sometimes also north) of the Sahara and their presence at Takarkori can be explained by their over-land migration to the area when the environmental conditions became more favourable at the beginning of the Holocene. The remains of most of the mammalian taxa identified at Takarkori can be seen as a result of human activity (hunting, snaring, and stock keeping). The remains of small, unidentified rodents and of the gerbils, however, may result from natural accumulation, e.g., denning animals, predation by mammalian carnivores or raptors. Although gerbils are routinely eaten when other food resources are scarce, they often dig into archaeological layers and die there. In that case they should be seen as penecontemporaneous intrusives (cf. taphonomic groups in [48]). The somewhat larger ground squirrel (cf. *Xerus erythropus*) and the gundi (*Massoutiera mzabi*) do not dig burrows and they should probably be seen as human food animals or the result of predator activity. The occurrence of carnivore modifications on dassies indicates that sometimes even larger species were predated and accumulated at the site. Porcupine (*Hystrix cristata*) and cane rat (*Thryonomys* cf. *swinderianus*) have been more likely exploited by humans as suggested, at least in the case the first taxon, by the presence of cut marks. The species spectrum is typical of an open environment that was much lusher than today as also indicated by the pollen data [28, 40]. Some of the taxa occur nowadays much farther south, e.g. the cane rat, rhinoceros and baboon. The majority of the other species are rather tolerant to dry conditions although not all of them can be found in the Acacus region today. The wild terrestrial fauna comprises large amounts of gazelles (both the dorcas gazelle and the somewhat larger dama gazelle *Nanger dama*), Barbary sheep, rock dassie and cape hare (*Lepus capensis*) that are nowadays inhabitants of arid and semi-arid environments. Also the carnivores are capable of surviving in such relatively dry conditions. That is certainly the case for the Libyan striped weasel (*Ictonyx libyca*), the sand cat (*Felis margarita*) and the fennec fox (*Vulpes zerda*), but also for the golden jackal (*Canis aureus*), the caracal (*Caracal caracal*) and the Egyptian mongoose (*Herpestes ichneumon*) (cf. ecological data mentioned in [90]). Most of these carnivores have a varied diet and may have been consuming fish more or less frequently. This may have occurred through active capture of fish in shallow water or by scavenging of individuals that died naturally or that were discarded by humans. The golden jackal, for instance, is known to be a carrion eater [90].

## Discussion

### Diachronic changes in the fauna of Takarkori

**Fig 9:**
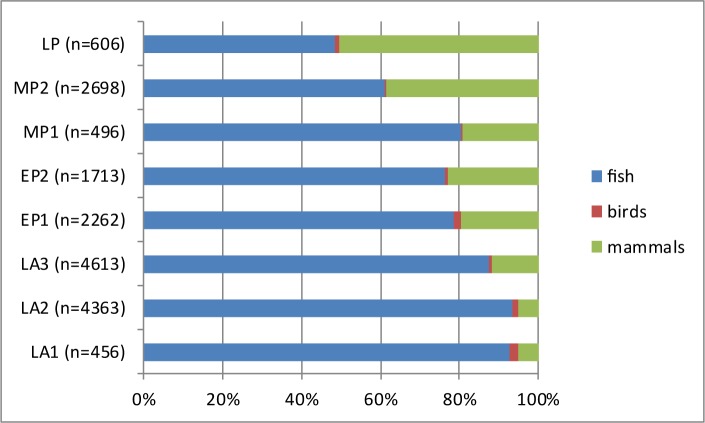
Proportions of the fish, birds and mammals in the various chronological phases. For each period the number of remains (n) is indicated on which the percentages are calculated. The fish bones are all identified specimens, in the case of mammals and birds both identified and unidentified remains are taken into account.

**Fig 10:**
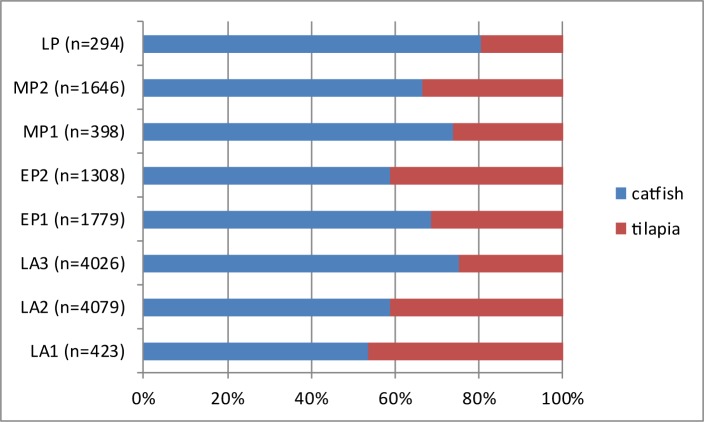
Proportions of the catfish and tilapia in the various chronological phases. For each period the number of remains (n) is indicated on which the percentages are calculated.

**Fig 11:**
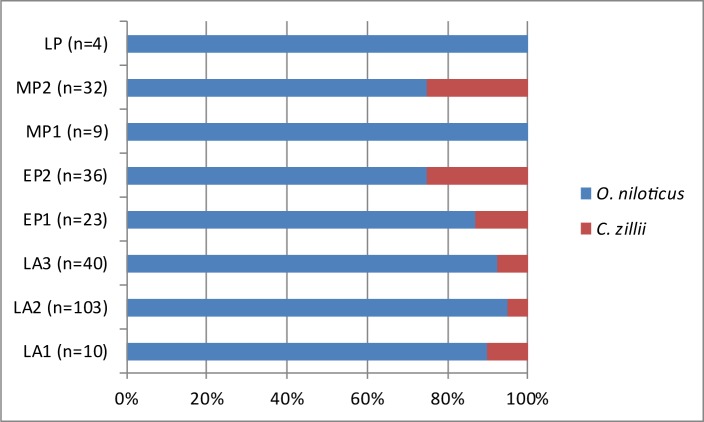
Proportions of the two tilapia species (*Oreochromis niloticus* and *Coptodon zillii*) in the various chronological phases. For each period the number of remains (n) is indicated on which the percentages are calculated.

**Fig 12:**
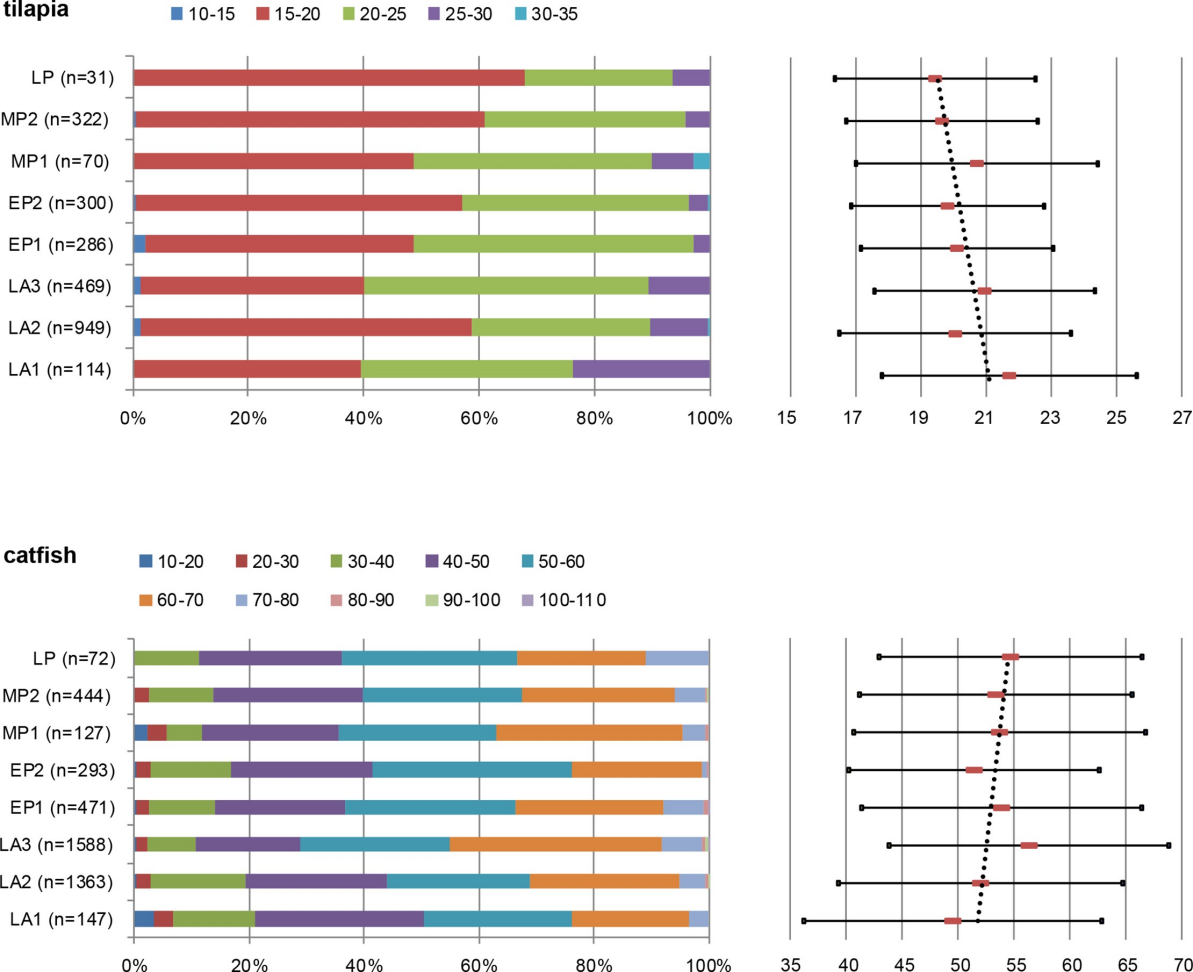
Diachronic changes in the size of the tilapia and catfish. The left pane indicates the % contribution of each of the length classes and in the right pane, the mean length and the ± standard deviations are indicated.

As already mentioned above, the major animal groups represented at Takarkori are fish (ca. 80%) followed by mammals (ca. 19%) and birds (ca. 1%). It appears however, that there is a diachronic change in the relative importance of these major animal taxa when the proportions are plotted by phase (Fig 9). The amount of fish is decreasing through time and the contribution of mammals increases, showing that people at Takarkori focussed gradually more on hunting and livestock keeping. It is unclear if this was an intentional process or if this shift could be related to increasing aridity, which made the environment less favourable for fishes. In Fig 10 the proportions of tilapia versus catfish are plotted through time. The graph shows that there is some variation in the proportions, but it appears that generally speaking the contribution of tilapia is decreasing through time. This could also be a result of the shift to drier conditions as clariid catfish have accessory breathing organs that allow them to use oxygen from the air [91]. They are therefore better adapted than tilapia to survive in shallow water bodies with high temperatures and low oxygen levels. Fig 11 shows the proportions of *Coptodon zillii* and *Oreochromis niloticus* through time. The amount of specifically identified tilapia is low in each phase and in some cases (for LP, MP1 and LA1) the percentages are probably not meaningful for that reason. If we only consider the phases that have 20 or more identified tilapia, there seems to be an increase in *Coptodon zillii* through time: the contribution of that species increases in EP1 and this continues into EP2 and remains at the same level in MP2. *Coptodon zillii* is better adapted than *O*. *niloticus* to survival in extreme conditions, i.e. low oxygen levels, high temperatures and in particular elevated salinity [92]. These data seem to suggest an increasing aridity from EP1 onwards, which is earlier than suggested by the catfish/tilapia proportions. The proportions through time of the different size classes of the catfish and tilapia, given in Fig 12, were calculated with the aim of observing possible trends that could be related to either fishing pressure or climate related growth changes. We added to each of these histograms a graph showing the means and ± standard deviations, and we provide the results of the T-tests (Table 3), comparing the means between periods. In the tilapia, the length class 25–30 cm SL decreases through time whereas the proportion of fish of the 15–20 cm SL length class increases towards the younger levels. The smaller average size may be due to changing environmental conditions. Unlike catfish, tilapia often become stunted when they live in adverse conditions in pools, gueltas or other small waterbodies [93]. In the catfish, there is an opposite trend, with fishes that become gradually larger towards the younger levels. A possible explanation for the size increase may be a reduction of the fishing pressure, suggested also by the aforementioned decrease of fishing activities compared to exploitation of birds and mammals.

**Table 3:** 

catfish	LA1	LA2	LA3	EP1	EP2	MP1	MP2	LP1
LA1	//							
LA2	*	//						
LA3	**	**	//					
EP1	**	**	**	//				
EP2	-	-	**	**	//			
MP1	**	-	*	-	-	//		
MP2	**	*	**	-	*	-	//	
LP1	**	-	-	-	*	-	-	//
								
tilapia								
LA1	//							
LA2	**	//						
LA3	*	**	//					
EP1	**	-	**	//				
EP2	**	-	**	-	//			
MP1	-	-	-	-	*	//		
MP2	**	-	**	-	-	**	//	
LP1	**	-	*	-	-	-	-	//

Comparison of the means of the catfish and the tilapia between periods using T-test (PAST, version 3.26b): no difference: -; significant different: p<0.05 *, highly significant different p<0.01 **

The two aquatic reptile species, the crocodile and the Adanson’s mud turtle, occur sporadically at the site. The crocodile is attested since the EP1 and occurs until the LP, whereas the mud turtle is found from the EP1 to MP2 phase. As it seems unlikely that they would have colonised the area later than the fish, their absence in the LA phases might be due either to their overall rarity and chance fluctuations or, alternatively, it could be postulated that people started exploiting these aquatic reptiles only from the EP1 phase onwards. This could then be due to more arid conditions, resulting in less extensive waters with crocodiles and mud turtles that became more vulnerable because the water levels had dropped. If fish became less plentiful, people may also have started exploiting this alternative food source. Hunting crocodiles may also have been a way of reducing numbers of a species that was competing for the same major food resource that fish represented.

In the case of mammals, the only trend seen is the adoption of livestock keeping since the early pastoral phase; this is a general local and regional trend, documented at other sites (e.g., [94]) as much as at Takarkori [13].

### Palaeoenvironmental conditions and changes in fish fauna

From the paragraphs above, it is clear that the major faunal trends through time are all observed within the fish, besides their changing proportion versus the mammals. It appears that these diachronic trends in exploitation of aquatic resources, and especially fish, can be explained to a large extent by climatic changes in early and middle Holocene times. The closest water environments to Takarkori rock shelter (Fig 3) was the lake about 6.5 kilometres as the crow flies west of the shelter. Analogous basins slightly farther away to the east are in the inner part of the Tadrart Acacus massif and the upper course of the Wadi Tanezzuft. Much farther from the site several lakes were located, including the Garat Ouda basin and the piezometric lakes among the dunes of the Erg Uan Kasa, Erg Tanezzuft, and Erg Titersine. All these aquatic environments were active in the early and middle Holocene, suffered periodic level fluctuations, and underwent rapid or progressive level drops or desiccation since the mid-Holocene transition [25].

The shallow aquifer feeding basins among dunes were recharged since the beginning of the Holocene and at ca. 10,000 years BP permanent lakes were active. According to sedimentological data [27, 56], many of the piezometric lakes among dunes suffered a strong drop in lake level and increasing desiccation at the end of the 9th millennium BP, as potential consequence of the cold/dry phase dated at 8.2 ka BP that triggered the interruption of the monsoon system [24]. After this disruption, the lake level rose again in the middle Holocene, but interdune lakes suffered strong seasonal level changes and at ca. 5500 years BP they disappeared. Hundreds of archaeological sites dot the shorelines of ancient interdune lakes, and in many cases fish bones were retrieved, suggesting the exploitation of aquatic resources.

Compared to the relatively smaller interdune lakes that may have disappeared for a few centuries around 8000 cal BP, the situation must have been less extreme for the Garat Ouda and Takarkori Lakes, which are geomorphologically very different. The Garat Ouda basin survived throughout the wet Holocene–including the 8.2 ka BP–, sustained by the Wadi Tanezzuft, up to its desiccation at ca. 5000 years BP. At Garat Ouda in the middle Holocene the landscape shifted from a lake basin to a delta-lake/playa system. In this phase, we have abundant evidence of fish exploitation in the area; in fact, many middens of–thus far unstudied–fish bones were mapped [21]. The history of the Wadi Tanezzuft is well known and its activity is reconstructed for most of the Holocene [25], showing that the river was active since the beginning of the Holocene and survived the aridity in the middle Holocene. The river existed–even with a shorter course—up to two millennia ago. Near-surface aquifers and the surface drainage network located on the eastern Tassili plateau sustained the small lake west of Takarkori rock shelter. Likely, also the Takarkori Lake had the same trajectory of the Garat Ouda Lake. Field evidence suggests that the lake was permanent in the early and middle Holocene and progressively the level dropped in the middle Holocene; likely, in the latter period it was more sensitive to seasonal fluctuations.

The palaeohydrological data help interpreting trends in fish exploitation at Takarkori represented in Figs 9–11. The largest fish exploitation dates to the early Holocene, when plenty of permanent wet environments (lakes and a large river) were able to support aquatic life. In the course of the middle Holocene, on the contrary, water bodies become more prone to seasonal fluctuations. This, from one side, led to a general reduction of the availability of fish and its consumption from the Middle Pastoral 2 onwards (Fig 9), up to the Late Pastoral 1, when fish bones in the archaeological record halved. The observed trends in the ichthyofauna, i.e. the increase in catfish when compared to the tilapia and the growing amount of *Coptodon zillii* among the tilapia in the LA3 phase, can be explained by increased aridity. This must have made the waters less suitable for tilapia and in particular *Oreochromis niloticus*. Clariid catfish, thanks to their accessory breathing organs and greater tolerance to high salinity levels, could thrive much better. Also the increased seasonality in the fluctuation of lake levels may explain the greater consumption of catfish in the middle Holocene and especially in the LP phase, as this species better survives drops in the water level.

### Implications of the aquatic fauna at Takarkori for the palaeohydrography of Sahara

Nowadays, the area of Takarkori does not harbour any aquatic species anymore and the spectrum of terrestrial species is limited compared to the one attested at the archaeological site. The present-day environment may to a large extent be comparable to the one before the onset of the wetter conditions at the beginning of the Holocene. Because the fish species that we described above are unescapably confined to their water basins, they are good indicators for former hydrological connections between areas where relic populations survived during arid periods and areas that were newly colonised after wetter conditions set in.

In order to define possible waterways through which fish, and probably also crocodile and aquatic turtle, colonised the area of Takarkori at the beginning of the Holocene, it is necessary to first consider where relic populations of these taxa may have been present in the arid late Pleistocene. Starting from this distribution it is possible to try defining waterways through which the animals from particular refuge areas reached the region of Takarkori.

This kind of approach has been used in northwestern Sudan, where Holocene sites with numerous fish bones occur in the now dry Middle Wadi Howar region. The wide species spectrum included many fish taxa not known from refuge areas in the Sahara, and therefore testified a connection with the Nile through the still poorly documented Lower Wadi Howar, during the peak of the Holocene wet phase [58, 95]. But if in the case of the Wadi Howar we have topographic evidence of a connection between its upper reaches and the Nile River, the case of the central Sahara is more difficult. In fact, in the latter region there is no direct waterway connecting the central Sahara with the main hydrographic networks of the Nile River basin and the Sahelian river/lake basins. Notwithstanding this, we might propose some hypotheses.

The number of aquatic taxa at Takarkori is however limited, the *Clarias* and tilapia, as well as the crocodile and aquatic turtle that are found there may have come in theory from any of the large rivers or basins further south and east (Nile, Senegal, Niger rivers or Lake Chad), and some of them are also known from refuge areas in the Sahara itself. The location of these modern or sub-recent populations in the desert have been mentioned in the species descriptions above and the most relevant ones for us include the Ennedi and Tibesti for *Clarias*, *Coptodon zillii* and the crocodile. Both *Clarias* and *Coptodon zillii* are known nowadays from guelta Efeni (23°49’N, 5°47’E) in the Hoggar area. *Coptodon zillii* has also been reported from the Tassili. However, connections need to be supposed with more distant areas as shown by the presence at Takarkori of *Oreochromis niloticus*, the other tilapia species identified at the site that is only found today in the Chad, Nile and other large rivers of the Nilo-Sudan ichthyofaunal province. The same is true for the aquatic turtle *Pelusios adansonii* that is not found in any of the Saharan waterbodies. Crocodiles, finally, live or lived until rather recent times in the Ennedi, Tibesti and Hoggar.

Taking into account all the information above, it is clear that the climatic changes at the beginning of the Holocene must have induced changes in the drainage networks–namely their reactivation–that allowed aquatic turtles and *Oreochromis niloticus* to colonise the Takarkori area. And if this water-connection existed, it is clear that in theory also *Clarias* catfish, *Coptodon zillii* and crocodiles may have taken the same route. Of course, it is not excluded that Saharan relic populations of these taxa may have contributed to the fauna of Takarkori. What still seems strange is why *Sarotherodon galilaeus*, the third tilapia species known from the Nilo-Sudan ichthyofaunal province is absent at Takarkori despite the fact that it has the same preservation chances as the two other species.

**Fig 13:**
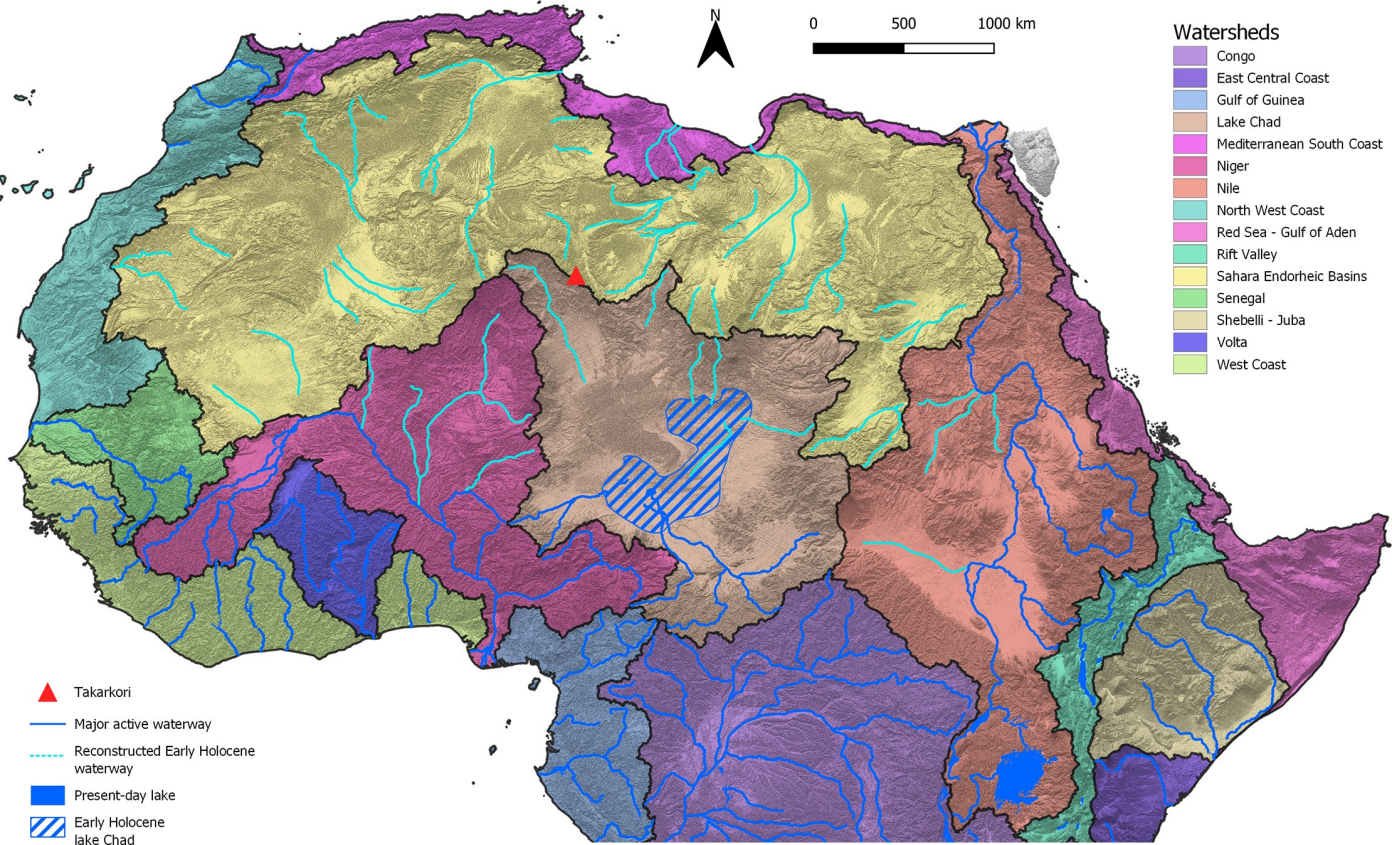
Reconstruction of the main active and fossil North African hydrographic basins elaborated from HydroSHEDS (www.hydrosheds.org).

**Fig 14:**
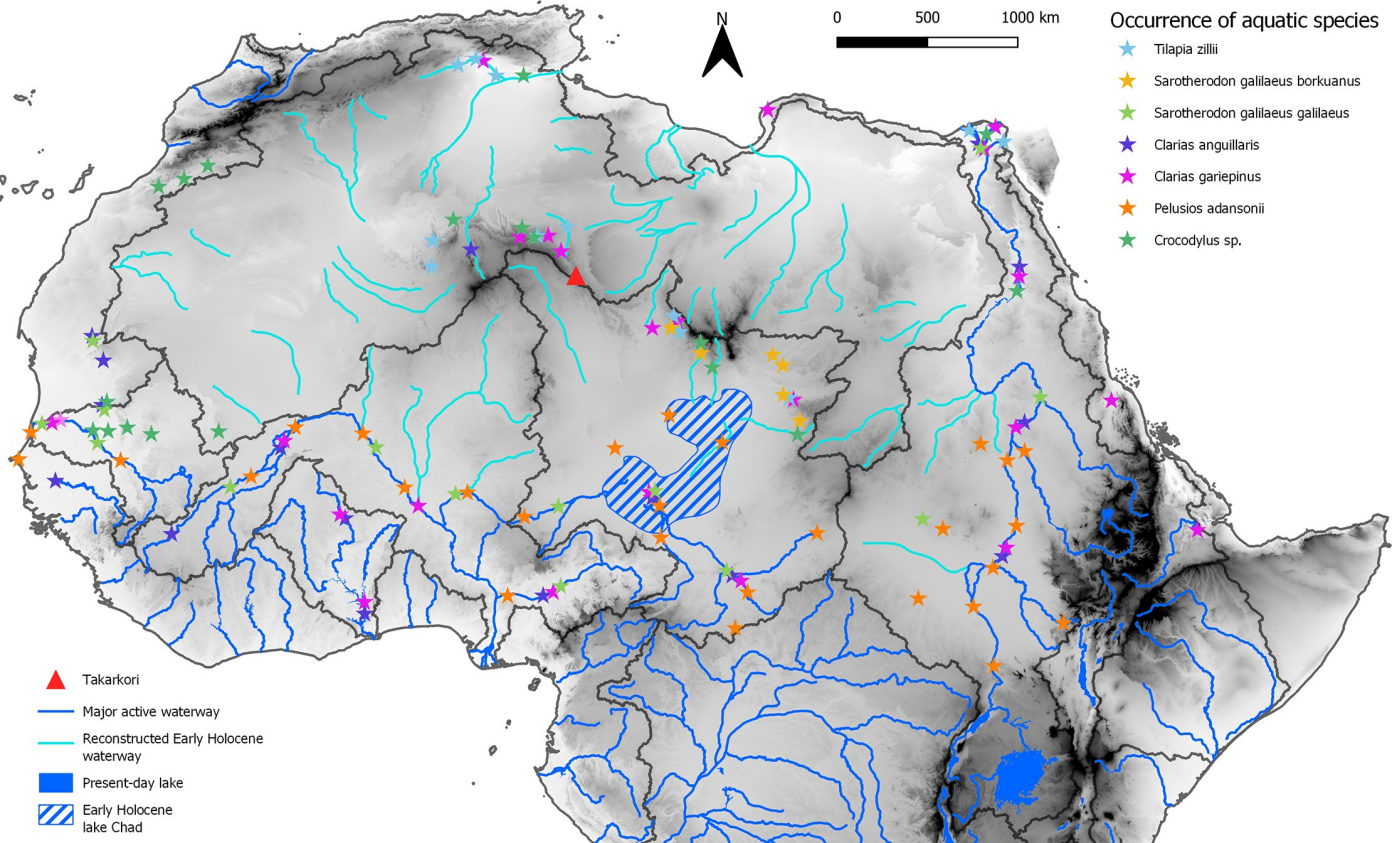
Extant occurrence of selected aquatic species (fish, crocodile, and turtle) in North Africa waterways (according to: Scortecci, 1937; Gautier and Van Neer, 1982; Lévêque, 1990; Bour, 2008; Brito et al., 2011). *Crocodylus* distribution has to be considered continuous in the present waterways south of the Sahara desert and along the Nile River south of Aswan.

**Fig 15:**
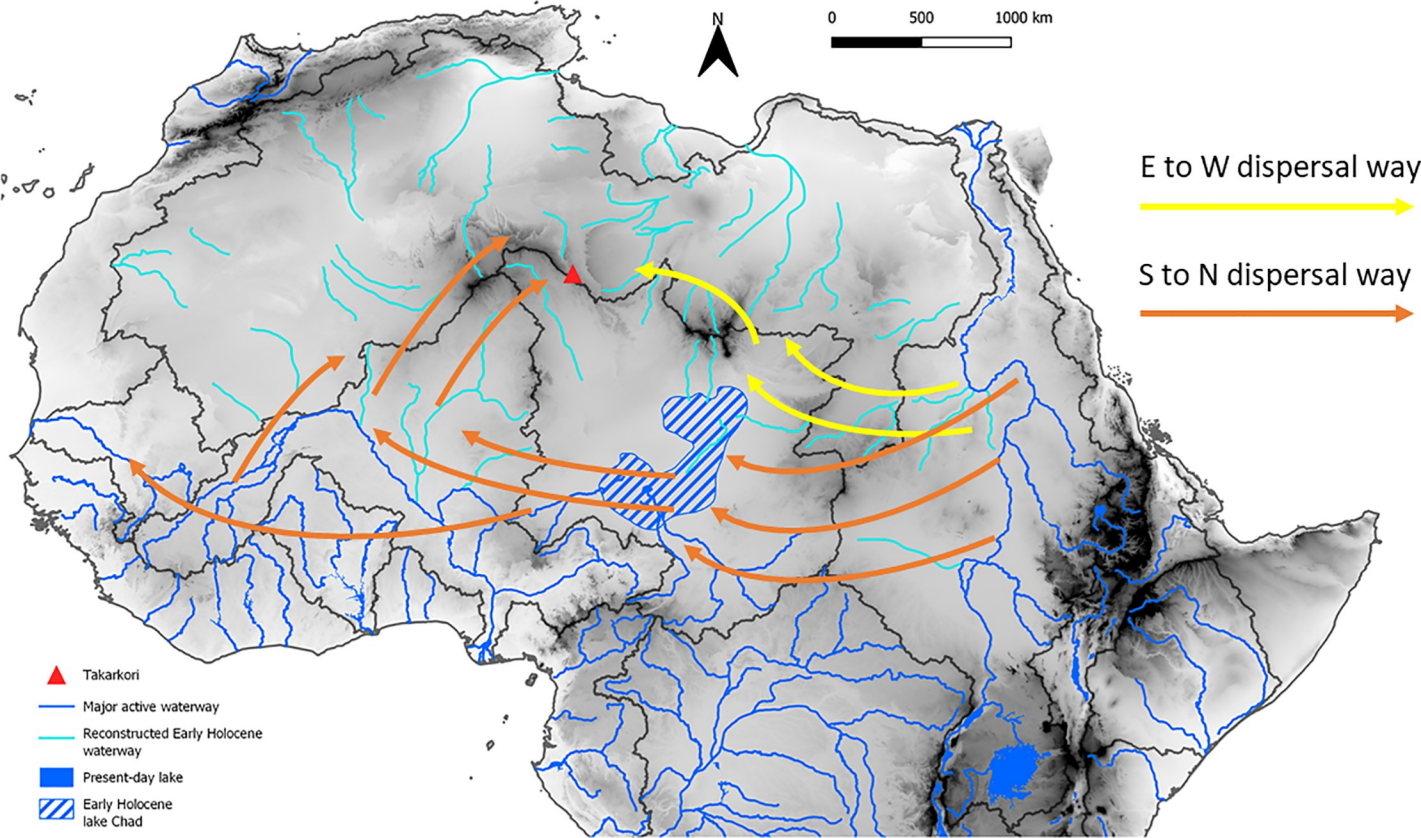
Possible colonization pathways of aquatic species to the central Sahara; detailed discussion is in the text.

Emerging evidence suggests that along the Quaternary, Saharan river systems underwent repeated reactivation in correspondence of interglacial periods (e.g., [96, 97, 98, 99]). The last of these events was triggered by increased precipitation in the early and middle Holocene (e.g., [100])–the present interglacial. In this last period, hydrological changes also triggered the expansion of the Lake Chad [44]–forming a proper megalake–and the formation of isolated wetlands and small lakes in most of the Sahara north of the present-day Sahelian belt [101] (Fig 13). The latter basins were active inside interdune basins and along major depressions, as common in the Libyan central Sahara [17]. The general reactivation of surface hydrology made possible the migration of species along the catchment basis. For instance, Hugueny and Lévêque [102] suggest that the distribution of fishes in North and West Africa is the result of past climatic and geological events, and especially they emphasise the role of alternating wet and dry periods, mountains as dispersal barriers, and links via lagoons to explain the present-day biogeographical setting. Yet, Drake and colleagues [97] suggest that palaeoriver networks created several very large alluvial fans with many distributary channels in the inland area; fans were located on the boundary between two catchment areas and their channels may have temporarily linked adjacent river systems permitting species to migrate from one basin to the next. Notwithstanding this, to establish the proper waterway followed by aquatic species in the colonization of the central Sahara is a difficult task. If we consider the abovementioned extant distribution of relic species (Fig 14) we may consider two possible colonization patterns (Fig 15): (i) an East to West way, and (ii) a South to North way. The first scenario implies the migration of species from the eastern sector of the Sahara and the Nile Valley, moving towards the west along the hydrographic nets of the Ennedi and Tibesti mountains and then northwards via Lake Mega-Chad. In the second hypothesis, the main migration patterns would have started from the Sahelian rivers and the Lake Mega-Chad moving northward up, reaching the Hoggar-Tassili hydrographic network and then reaching the Takarkori region through the palaeo-tributary of the Niger River described in [100]. Possibly, the two hypotheses on the migration waterways are not mutually exclusive, and aquatic species completed the colonization of the Sahara exploiting any possible way. What is evident is the role played by Lake Mega-Chad. In both hypotheses, the exceptional early Holocene expansion of Lake Chad [103] likely played a pivotal role in the colonization process, especially if we consider fishes. In fact, such a huge wetland offered an extensive humid to riparian environment representing a main hub for the mobility of fishes. If we look at the topography of the Sahara and superimposed reconstructed catchments (see for instance the one in [68], we note a difficult communication between the Tibesti massif and the Murzuq-Messak system. The Tibesti looks like a natural barrage to colonization and the Holocene palaeohydrology of the area seems relatively poorly developed [18]. On the contrary, the early and middle Holocene Sahelian catchments–Lake Mega-Chad, Niger River, Senegal River—were tightly interconnected with the hydrographic net of the Hoggar massif [100], thus suggesting a more likely colonization pathway for aquatic species.

The nature of the connection should probably not be seen as real waterways running over long distances and crossing the whole Sahara; but rather as a series of catchments that were active and communicating with each other when conditions were periodically or even seasonally more humid. Certainly for the catfish and tilapia, which have limited possibilities of moving over land, it can be hypothesized that they were able to propagate during flood events, when water, with the fish, was decanted from one catchment to the other. Anyway, Saharan watersheds are in many cases difficult to connect even if we consider the occurrence of flooding events, mega-fan divagation, and close basins overflow [97].

We believe it is unlikely that mechanisms other than the aforementioned migration of fauna during events of exceptional rainfall have played a role in the mobilization of aquatic fauna from a basin to the next one. It has been suggested that in the Holocene birds acted as a mechanism to spread mollusc species between lake and river systems of the Ounianga region as much as in most of the hydrologically isolated sites of the Sahara [104]. Among the species they mention is *Melanoides tuberculata* that we also find at Takarkori, but not *Corbicula consobrina*. However, the relatively high temperatures and low air humidity must have made it extremely unlikely that the less resistant fish eggs or larvae were spread by aquatic birds. During exceptional storms fish can be lifted up [105] and this phenomenon has been supposed to be sometimes responsible for the spread of fish over shorter distances. For instance, in a phylogeographic study of cichlid fish from several adjacent Nicaraguan crater lakes it has been hypothesized that the colonization occurred as a consequence of intense storms mobilizing fishes from adjoining basins, rather than by human transport [106]. As we have argued before [107], these ‘natural’ mechanisms are highly unlikely to be effective in our study region as is illustrated by the fact that there are numerous waterbodies in the Sahara that remain devoid of fish despite the fact that they are on the route of migratory birds. Also the high rate of speciation of killifish (Cyprinodontiformes) in western Africa is difficult to understand. Adjacent basins have different species although the eggs of these fish are the most drought resistant of all African fish and thus ideal candidates to be transported by birds. Jubb [108] also underlines that there is no evidence for the dispersal of fish by birds or other animals. As an example, he mentions the ichthyofaunas above and below a waterfall on the river Lundi in Zimbabwe that remain different despite the fact that numerous bird taxa occur that could theoretically spread fish from one part of the river to the other.

## Conclusions

The remains of both terrestrial and aquatic animals retrieved during the excavation of the Takarkori rock shelter illustrate the more humid climatic conditions in Saharan southwestern Libya during early and middle Holocene times. As the material is so abundant–consisting of 17,551 identifiable remains–and covering a long period–between 10,200 and 4650 years cal BP–it was possible to search for diachronic trends in the faunal spectrum of this unique assemblage. No significant changes in the species composition of the mammals, birds, reptiles or fish are noted except for the appearance of domestic animals (sheep/goat and cattle) from the Early Pastoral phase 1 (8300–7600 cal BP) onwards. However, clear diachronic trends are noted in the proportions of various taxa. In the Late Acacus period (10,200 to 8000 cal BP) fish remains represent around 90% of all the remains. Interestingly, this proportion remains very robust (80%) until the Middle Pastoral 1 (6400 to 5600 cal BP) and it then drops in the Late Pastoral phase (5900–4650 cal BP) when fish contribute only about 48% of the remains. This shift is in line with the palaeohydrological and geomorphological data that could be gathered for the region, indicating the presence of extensive permanent wet environments during the early Holocene, followed by more unstable conditions during the middle Holocene, when the number and extent of waterbodies decreased, and their seasonality increased, resulting in major level fluctuation. The aridification and the increase in seasonality explain the decrease in relative importance of fish compared to the terrestrial fauna as well as the observed changes in the fish fauna itself. Clariid catfish, that can better withstand adverse environmental conditions (low oxygen content, high temperatures and salinity) become more frequent through time compared to the tilapia. Among the tilapia, an increase is observed in the proportion of the species *Coptodon zillii* that is better adapted to arid conditions than *Oreochromis niloticus* and that is still the most common cichlid in the present-day Sahara. Moreover, it appears that the tilapia become smaller in the middle Holocene, possibly as a result of stunting which is known to occur in these fish when waterbodies are small.

The presence of the fish, of the aquatic turtle *Pelusios adansonii* and the crocodile that are nowadays absent from the region, indicates that connections must have existed in the early Holocene allowing these aquatic animals to colonise the area, starting from places where populations were present in late Pleistocene times. The modern distribution of the aquatic species, which must be comparable to that during the late Pleistocene, was combined with geomorphological and hydrological data of northern Africa to define two possible routes for the colonisation. The species may have come from the Nile, migrating westwards through the eastern Sahara along the hydrographic networks of the Ennedi and Tibesti and the Lake Mega-Chad. Alternatively, the aquatic species may have come from rivers in the Sahel and the Lake Mega-Chad, propagating northwards towards the Hoggar-Tassili hydrographic network and from there towards the Tadrart Acacus area through the palaeotributary of the Niger River. Although it cannot be ascertained which of the postulated routes were actually responsible for the colonisation of the Takarkori area, it is clear that in both scenarios Lake Mega-Chad played an important role in the propagation of aquatic species. It should be underlined as well that these reconstructed palaeohydrographic networks should not be seen as permanently flowing, long waterways. Colonisation may have been a gradual process whereby a series of catchments were active that may have communicated with each other when conditions were periodically, or even seasonally, more humid allowing fish to be ‘decanted’ from one catchment to the next.

## Supporting information

S1 Table Fish identifications. List of all the identified fish bone by the chrono-cultural phase. Indicated are the taxon, the skeletal element, the number of these skeletal elements found (NISP), fish length reconstruction expressed in cm standard length (SL), and the number of specimens that could be sized in each case.
(PDF)
