# Supplementary material for: Aquatic fauna from the Takarkori rock shelter reveals the Holocene central Saharan climate and palaeohydrography
Source: PLoS One. 2020 Feb 19;15(2):e0228588. doi: 10.1371/journal.pone.0228588 (PMC7029841; doi:10.1371/journal.pone.0228588)
Supplement: S1 Table — List of all the identified fish bone by the chrono-cultural phase. Indicated are the taxon, the skeletal element, the number of these skeletal elements found (NISP), fish length reconstruction expressed in cm standard length (SL), and the number of specimens that could be sized in each case. (PDF) [file pone.0228588.s001.pdf]

cultural phase	taxon	skeletal element	NISP	SL in cm	number
LA1	O. niloticus	1st precaudal vertebra	1	20-25	1
LA1	O. niloticus	1st precaudal vertebra	1	25-30	1
LA1	Haplotilapiini indet.	1st precaudal vertebra	1	15-20	1
LA1	Clarias sp.	cranial roof fragment	1	60-70	1
LA1	Haplotilapiini indet.	basipterygium	1	15-20	1
LA1	Haplotilapiini indet.	caudal vertebra	1	15-20	1
LA1	Haplotilapiini indet.	caudal vertebra	1	20-25	1
LA1	Haplotilapiini indet.	skull roof fragment	1		
LA1	Haplotilapiini indet.	fin spine	1		
LA1	Haplotilapiini indet.	precaudal vertebra	2	15-20	2
LA1	Clarias sp.	caudal vertebra	1	60-70	1
LA1	Clarias sp.	cranial roof fragment	1	60-70	1
LA1	Clarias sp.	urohyale	1	50-60	1
LA1	Clarias sp.	cranial roof fragment	2	40-50	2
LA1	Clarias sp.	cranial roof fragment	2		
LA1	Haplotilapiini indet.	caudal vertebra	1	15-20	1
LA1	Haplotilapiini indet.	cleithrum	1		
LA1	Haplotilapiini indet.	precaudal vertebra	1	15-20	1
LA1	Haplotilapiini indet.	dorsal or anal pterygophore	1	15-20	1
LA1	Haplotilapiini indet.	neurocranium fragment	2		
LA1	Haplotilapiini indet.	lepidotrich	2		
LA1	Clarias sp.	caudal vertebra	1		
LA1	Clarias sp.	cleithrum	1		
LA1	Clarias sp.	cranial roof fragment	1	50-60	1
LA1	Clarias sp.	cranial roof fragment	1		
LA1	Clarias sp.	pectoral spine	1	50-60	1
LA1	C. zillii	articular	1	20-25	1
LA1	Haplotilapiini indet.	basioccipital	1	20-25	1
LA1	Haplotilapiini indet.	cleithrum	1		
LA1	Haplotilapiini indet.	skull roof fragment	1		
LA1	Haplotilapiini indet.	mesethmoid	1	25-30	1
LA1	Haplotilapiini indet.	opercular	1	15-20	1
LA1	Haplotilapiini indet.	precaudal vertebra	1	20-25	1
LA1	Haplotilapiini indet.	dorsal or anal pterygophore	1	15-20	1
LA1	Haplotilapiini indet.	lepidotrich	2		
LA1	Clarias sp.	articular	1	60-70	1
LA1	Clarias sp.	basioccipital	1	60-70	1
LA1	Clarias sp.	caudal vertebra	1	40-50	1
LA1	Clarias sp.	cleithrum	1	40-50	1
LA1	Clarias sp.	cranial roof fragment	1	30-40	1
LA1	Clarias sp.	cranial roof fragment	1		
LA1	Clarias sp.	hyomandibula	1	30-40	1
LA1	Clarias sp.	opercular	1		
LA1	Clarias sp.	precaudal vertebra	1	30-40	1
LA1	Clarias sp.	urohyale	1	40-50	1
LA1	Haplotilapiini indet.	articular	1	25-30	1
LA1	Haplotilapiini indet.	skull roof fragment	1		
LA1	O. niloticus	hyomandibula	1	15-20	1
LA1	Haplotilapiini indet.	precaudal vertebra	1	15-20	1
LA1	Haplotilapiini indet.	3rd precaudal vertebra	1	20-25	1
LA1	Haplotilapiini indet.	cleithrum	2		
LA1	Haplotilapiini indet.	lepidotrich	2		
LA1	Clarias sp.	articular	1	30-40	1
LA1	Clarias sp.	ceratohyale	1	50-60	1
LA1	Clarias sp.	cleithrum	1	40-50	1
LA1	Clarias sp.	articular	1	50-60	1
LA1	Clarias sp.	cleithrum	1		
LA1	Clarias sp.	premaxilla	1	40-50	1

cultural phase	taxon	skeletal element	NISP	SL in cm	number
LA1	Haplotilapiini indet.	basipterygium	1	15-20	1
LA1	Haplotilapiini indet.	fin spine	1		
LA1	Haplotilapiini indet.	precaudal vertebra	1	15-20	1
LA1	Clarias sp.	basipterygium	1	15-20	1
LA1	Clarias sp.	caudal vertebra	1	15-20	2
LA1	Clarias sp.	caudal vertebra	1	25-30	1
LA1	Clarias sp.	cranial roof fragment	1	40-50	1
LA1	Clarias sp.	hyomandibula	1	50-60	1
LA1	Clarias sp.	opercular	1	20-30	1
LA1	Clarias sp.	pterygophore	1		
LA1	Clarias sp.	quadrate	1	40-50	1
LA1	Clarias sp.	cranial roof fragment	2		
LA1	Clarias sp.	precaudal vertebra	2	15-20	2
LA1	Haplotilapiini indet.	cleithrum	1		
LA1	Clarias sp.	cranial roof fragment	1	30-40	1
LA1	Haplotilapiini indet.	basipterygium	1	20-25	1
LA1	Haplotilapiini indet.	opercular	1	20-25	1
LA1	Haplotilapiini indet.	opercular	1	25-30	1
LA1	Haplotilapiini indet.	precaudal vertebra	1	15-20	1
LA1	Haplotilapiini indet.	precaudal vertebra	1	20-25	1
LA1	Clarias sp.	basioccipital	1	30-40	1
LA1	Clarias sp.	caudal vertebra	1	40-50	1
LA1	Clarias sp.	caudal vertebra	1	60-70	1
LA1	Clarias sp.	cleithrum	1	40-50	1
LA1	Clarias sp.	cleithrum	1	30-40	1
LA1	Clarias sp.	cleithrum	1		
LA1	Clarias sp.	cranial roof fragment	1	60-70	1
LA1	Clarias sp.	pectoral spine	1	30-40	1
LA1	Clarias sp.	cranial roof fragment	2	40-50	2
LA1	Clarias sp.	cranial roof fragment	4		
LA1	Haplotilapiini indet.	basioccipital	1	15-20	1
LA1	Haplotilapiini indet.	basipterygium	1	15-20	1
LA1	Haplotilapiini indet.	ceratohyale	1	25-30	1
LA1	Haplotilapiini indet.	cleithrum	1	25-30	1
LA1	Haplotilapiini indet.	cleithrum	1		
LA1	O. niloticus	hyomandibula	1	25-30	1
LA1	Haplotilapiini indet.	opercular	1	20-25	1
LA1	Haplotilapiini indet.	opercular	1		
LA1	Haplotilapiini indet.	precaudal vertebra	1	15-20	2
LA1	Haplotilapiini indet.	postcleithrum	1	20-25	1
LA1	Haplotilapiini indet.	dorsal or anal pterygophore	1	20-25	1
LA1	Haplotilapiini indet.	neurocranium fragment	2	20-25	2
LA1	Haplotilapiini indet.	skull roof fragment	4		
LA1	Haplotilapiini indet.	cleithrum	1	25-30	1
LA1	Haplotilapiini indet.	neurocranium fragment	2		
LA1	Clarias sp.	caudal vertebra	1	60-70	1
LA1	Clarias sp.	quadrate	1	60-70	1
LA1	Clarias sp.	cranial roof fragment	3		
LA1	Haplotilapiini indet.	skull roof fragment	1	25-30	1
LA1	Haplotilapiini indet.	fin spine	1		
LA1	Haplotilapiini indet.	precaudal vertebra	1	25-30	1
LA1	Haplotilapiini indet.	2nd precaudal vertebra	1	25-30	1
LA1	Haplotilapiini indet.	cleithrum	2		
LA1	Clarias sp.	articular	1	60-70	1
LA1	Clarias sp.	basioccipital	1	40-50	1
LA1	Clarias sp.	cleithrum	1	50-60	1
LA1	Clarias sp.	cleithrum	1	30-40	1
LA1	Clarias sp.	coracoid	1	60-70	1

cultural phase	taxon	skeletal element	NISP	SL in cm	number
LA1	Clarias sp.	cranial roof fragment	1	20-30	1
LA1	Clarias sp.	hyomandibula	1		
LA1	Clarias sp.	mesethmoid	1	40-50	1
LA1	Clarias sp.	opercular	1	40-50	1
LA1	Clarias sp.	urohyale	1	60-70	1
LA1	Clarias sp.	urohyale	1	40-50	1
LA1	Clarias sp.	cranial roof fragment	10		
LA1	Clarias sp.	caudal vertebra	2	60-70	2
LA1	Clarias sp.	coracoid	2	50-60	2
LA1	Clarias sp.	cranial roof fragment	2	50-60	2
LA1	Clarias sp.	cranial roof fragment	2	60-70	2
LA1	Clarias sp.	precaudal vertebra	3	60-70	3
LA1	Clarias sp.	cranial roof fragment	5	40-50	5
LA1	Haplotilapiini indet.	articular	1	20-25	1
LA1	Haplotilapiini indet.	dorsal or anal pterygophore	1	20-25	1
LA1	Haplotilapiini indet.	dorsal or anal pterygophore	1	20-25	1
LA1	Haplotilapiini indet.	cleithrum	2		
LA1	Haplotilapiini indet.	skull roof fragment	4		
LA1	Clarias sp.	cranial roof fragment	1	40-50	1
LA1	Clarias sp.	cranial roof fragment	1		
LA1	Haplotilapiini indet.	cleithrum	1		
LA1	Haplotilapiini indet.	epihyale	1		
LA1	Haplotilapiini indet.	postcleithrum	1	25-30	1
LA1	Haplotilapiini indet.	dorsal or anal pterygophore	1	20-25	1
LA1	Clarias sp.	cranial roof fragment	1	50-60	1
LA1	Haplotilapiini indet.	basipterygium	1	15-20	1
LA1	Haplotilapiini indet.	cleithrum	1	15-20	1
LA1	O. niloticus	hyomandibula	1	20-25	1
LA1	Haplotilapiini indet.	fin spine	1		
LA1	Haplotilapiini indet.	preopercular	1		
LA1	Haplotilapiini indet.	postcleithrum	1	15-20	1
LA1	Haplotilapiini indet.	caudal vertebra	2	15-20	1
LA1	Clarias sp.	caudal vertebra	1	30-40	1
LA1	Clarias sp.	caudal vertebra	1	60-70	1
LA1	Clarias sp.	cranial roof fragment	1		
LA1	Clarias sp.	opercular	1	40-50	1
LA1	Clarias sp.	quadrate	1	20-30	1
LA1	Haplotilapiini indet.	caudal vertebra	1	25-30	1
LA1	Haplotilapiini indet.	cleithrum	1		
LA1	Haplotilapiini indet.	skull roof fragment	1		
LA1	Haplotilapiini indet.	fin spine	1		
LA1	Haplotilapiini indet.	precaudal vertebra	1	25-30	1
LA1	Clarias sp.	caudal vertebra	1	50-60	1
LA1	Clarias sp.	ceratohyale	1	30-40	1
LA1	Clarias sp.	cranial roof fragment	1	50-60	1
LA1	Clarias sp.	mesethmoid	1	40-50	1
LA1	Clarias sp.	opercular	1	40-50	1
LA1	Clarias sp.	quadrate	1	20-30	1
LA1	Clarias sp.	caudal vertebra	2	60-70	2
LA1	Clarias sp.	cranial roof fragment	2	60-70	2
LA1	Clarias sp.	cranial roof fragment	2		
LA1	Haplotilapiini indet.	ceratohyale	1	15-20	1
LA1	Haplotilapiini indet.	cleithrum	1		
LA1	Haplotilapiini indet.	hyomandibula	1	15-20	1
LA1	Haplotilapiini indet.	hyomandibula	1	20-25	1
LA1	Haplotilapiini indet.	hyomandibula	1	20-25	1
LA1	Haplotilapiini indet.	opercular	1	20-25	1
LA1	Haplotilapiini indet.	opercular	1	25-30	1

cultural phase	taxon	skeletal element	NISP	SL in cm	number
LA1	Haplotilapiini indet.	precaudal vertebra	1	15-20	1
LA1	Haplotilapiini indet.	precaudal vertebra	1	25-30	1
LA1	Haplotilapiini indet.	dorsal or anal pterygophore	1	25-30	1
LA1	Haplotilapiini indet.	caudal vertebra	2	15-20	2
LA1	Haplotilapiini indet.	caudal vertebra	2	25-30	2
LA1	Haplotilapiini indet.	postcleithrum	2	20-25	2
LA1	Haplotilapiini indet.	skull roof fragment	4		
LA1	Clarias sp.	articular	1	50-60	1
LA1	Clarias sp.	palatinum	1		
LA1	Haplotilapiini indet.	cleithrum	1		
LA1	Haplotilapiini indet.	precaudal vertebra	1	25-30	1
LA1	Clarias sp.	cleithrum	1	30-40	1
LA1	Clarias sp.	cleithrum	1		
LA1	Clarias sp.	coracoid	1		
LA1	Clarias sp.	cranial roof fragment	1	40-50	1
LA1	Haplotilapiini indet.	caudal vertebra	1	20-25	1
LA1	Haplotilapiini indet.	cleithrum	1		
LA1	Haplotilapiini indet.	skull roof fragment	1		
LA1	Haplotilapiini indet.	fin spine	1		
LA1	Haplotilapiini indet.	opercular	1	20-25	1
LA1	Haplotilapiini indet.	skull roof fragment	1		
LA1	Clarias sp.	ceratohyale	1	60-70	1
LA1	Clarias sp.	cleithrum	1	50-60	1
LA1	Clarias sp.	cleithrum	1	40-50	1
LA1	Clarias sp.	coracoid	1	50-60	1
LA1	Clarias sp.	coracoid	1	50-60	1
LA1	Clarias sp.	dentary	1	40-50	1
LA1	Clarias sp.	cranial roof fragment	1		
LA1	Clarias sp.	cranial roof fragment	1	30-40	1
LA1	Clarias sp.	cranial roof fragment	1	60-70	1
LA1	Clarias sp.	hyomandibula	1	30-40	1
LA1	Clarias sp.	hypohyale	1	40-50	1
LA1	Clarias sp.	branchial element	1		
LA1	Clarias sp.	opercular	1	50-60	1
LA1	Clarias sp.	opercular	1	40-50	1
LA1	Clarias sp.	opercular	1	30-40	1
LA1	Clarias sp.	precaudal vertebra	1	50-60	1
LA1	Clarias sp.	caudal vertebra	2	50-60	2
LA1	Clarias sp.	cleithrum	2	40-50	2
LA1	Clarias sp.	precaudal vertebra	2	40-50	2
LA1	Clarias sp.	cranial roof fragment	9		
LA1	Haplotilapiini indet.	articular	1	20-25	1
LA1	Haplotilapiini indet.	maxilla	1	20-25	1
LA1	Haplotilapiini indet.	palatinum	1	25-30	1
LA1	Haplotilapiini indet.	preopercular	1		
LA1	Haplotilapiini indet.	dorsal or anal pterygophore	1	20-25	1
LA1	Haplotilapiini indet.	quadrate	1	25-30	1
LA1	Haplotilapiini indet.	urohyale	1		
LA1	Haplotilapiini indet.	caudal vertebra	2	15-20	2
LA1	Haplotilapiini indet.	lepidotrich	2		
LA1	Haplotilapiini indet.	skull roof fragment	3		
LA1	Haplotilapiini indet.	cleithrum	4		
LA1	Clarias sp.	cranial roof fragment	1	50-60	1
LA1	Clarias sp.	hypohyale	1	50-60	1
LA1	Clarias sp.	opercular	1	30-40	1
LA1	Clarias sp.	cranial roof fragment	3		
LA1	Haplotilapiini indet.	skull roof fragment	1		
LA1	Haplotilapiini indet.	opercular	1	25-30	1

cultural phase	taxon	skeletal element	NISP	SL in cm	number
LA1	Haplotilapiini indet.	opercular	1		
LA1	Haplotilapiini indet.	precaudal vertebra	1	15-20	1
LA1	Haplotilapiini indet.	dorsal or anal pterygophore	1	20-25	1
LA1	Clarias sp.	ceratohyale	1	40-50	1
LA1	Clarias sp.	cleithrum	1	50-60	1
LA1	Clarias sp.	coracoid	1	50-60	1
LA1	Clarias sp.	opercular	1	40-50	1
LA1	Clarias sp.	cranial roof fragment	2		
LA1	Clarias sp.	cranial roof fragment	3		
LA1	Haplotilapiini indet.	skull roof fragment	1		
LA1	Haplotilapiini indet.	basipterygium	1	15-20	1
LA1	Haplotilapiini indet.	caudal vertebra	1	25-30	1
LA1	Haplotilapiini indet.	opercular	1	15-20	1
LA1	Haplotilapiini indet.	dorsal or anal pterygophore	1	20-25	1
LA1	Haplotilapiini indet.	dorsal or anal pterygophore	1		
LA1	Haplotilapiini indet.	cleithrum	2		
LA1	Clarias sp.	cranial roof fragment	1	40-50	1
LA1	Clarias sp.	cranial roof fragment	1	70-80	1
LA1	Haplotilapiini indet.	precaudal vertebra	2	25-30	2
LA1	Clarias sp.	articular	1	30-40	1
LA1	Clarias sp.	cranial roof fragment	1	50-60	1
LA1	Clarias sp.	cranial roof fragment	1	70-80	1
LA1	Clarias sp.	precaudal vertebra	1		
LA1	Clarias sp.	pectoral spine	1	50-60	1
LA1	Clarias sp.	pectoral spine	1	60-70	1
LA1	Clarias sp.	quadrate	1	70-80	1
LA1	Clarias sp.	urohyale	1	50-60	1
LA1	Clarias sp.	cranial roof fragment	7		
LA1	Haplotilapiini indet.	basipterygium	1	15-20	1
LA1	Haplotilapiini indet.	caudal vertebra	1	20-25	1
LA1	Haplotilapiini indet.	ceratohyale	1	25-30	1
LA1	Haplotilapiini indet.	skull roof fragment	1		
LA1	Haplotilapiini indet.	precaudal vertebra	1	15-20	1
LA1	Haplotilapiini indet.	dorsal or anal pterygophore	1	20-25	1
LA1	Haplotilapiini indet.	dorsal or anal pterygophore	1	20-25	1
LA1	Haplotilapiini indet.	dorsal or anal pterygophore	1	20-25	1
LA1	Haplotilapiini indet.	lepidotrich	2		
LA1	Clarias sp.	cranial roof fragment	1	50-60	1
LA1	Haplotilapiini indet.	basioccipital	1	20-25	1
LA1	Haplotilapiini indet.	cleithrum	1		
LA1	Haplotilapiini indet.	opercular	1	15-20	1
LA1	Haplotilapiini indet.	precaudal vertebra	1	15-20	1
LA1	Haplotilapiini indet.	dorsal or anal pterygophore	1	20-25	1
LA1	Clarias sp.	pectoral spine	1	70-80	1
LA1	Haplotilapiini indet.	cleithrum	1		
LA1	Haplotilapiini indet.	dorsal or anal pterygophore	1	15-20	1
LA1	Clarias sp.	ceratohyale	1	30-40	1
LA1	Clarias sp.	coracoid	1	40-50	1
LA1	Clarias sp.	dentary	1	40-50	1
LA1	Clarias sp.	pectoral spine	1	30-40	1
LA1	Clarias sp.	cranial roof fragment	4		
LA1	Haplotilapiini indet.	caudal vertebra	1	15-20	1
LA1	Haplotilapiini indet.	coracoid	1		
LA1	O. niloticus	hyomandibula	1	20-25	1
LA1	Haplotilapiini indet.	fin spine	1		
LA1	Haplotilapiini indet.	precaudal vertebra	1	15-20	1
LA1	Haplotilapiini indet.	neurocranium fragment	2		
LA1	Clarias sp.	articular	1	40-50	1

cultural phase	taxon	skeletal element	NISP	SL in cm	number
LA1	Clarias sp.	cleithrum	1	50-60	1
LA1	Clarias sp.	cleithrum	1		
LA1	Clarias sp.	cranial roof fragment	1	50-60	1
LA1	Clarias sp.	cranial roof fragment	5		
LA1	Haplotilapiini indet.	dorsal or anal pterygophore	1	15-20	1
LA1	Clarias sp.	cranial roof fragment	1		
LA1	Clarias sp.	mesethmoid	1	40-50	1
LA1	Clarias sp.	pectoral spine	1	60-70	1
LA1	Clarias sp.	cranial roof fragment	2		
LA1	Haplotilapiini indet.	cleithrum	1		
LA1	Haplotilapiini indet.	opercular	1	20-25	1
LA1	Clarias sp.	coracoid	1		
LA1	Clarias sp.	cranial roof fragment	1		
LA1	Clarias sp.	opercular	1	30-40	1
LA1	Clarias sp.	cranial roof fragment	2	50-60	1
LA1	Clarias sp.	caudal vertebra	1	60-70	1
LA1	Clarias sp.	precaudal vertebra	1	40-50	1
LA1	Haplotilapiini indet.	precaudal vertebra	1	20-25	1
LA1	Haplotilapiini indet.	dorsal or anal pterygophore	1	20-25	1
LA1	Clarias sp.	caudal vertebra	1	50-60	1
LA1	Clarias sp.	cranial roof fragment	1	50-60	1
LA1	Haplotilapiini indet.	precaudal vertebra	1	15-20	1
LA1	Haplotilapiini indet.	caudal vertebra	2	15-20	1
LA1	Clarias sp.	caudal vertebra	1	50-60	1
LA1	Haplotilapiini indet.	caudal vertebra	1	15-20	1
LA1	Haplotilapiini indet.	precaudal vertebra	1	15-20	1
LA1	Clarias sp.	urohyale	1	30-40	1
LA1	Clarias sp.	cranial roof fragment	1	60-70	1
LA1	Clarias sp.	cranial roof fragment	1	70-80	1
LA1	Clarias sp.	hyomandibula	1		
LA1	Clarias sp.	caudal vertebra	2	50-60	2
LA1	Haplotilapiini indet.	skull roof fragment	1		
LA1	Haplotilapiini indet.	fin spine	1		
LA1	O. niloticus	mesethmoid	1	20-25	1
LA2	Haplotilapiini indet.	1st precaudal vertebra	1	30-35	1
LA2	O. niloticus	1st precaudal vertebra	1		
LA2	Haplotilapiini indet.	1st precaudal vertebra	1	20-25	1
LA2	Haplotilapiini indet.	1st precaudal vertebra	1	20-25	1
LA2	O. niloticus	1st precaudal vertebra	1	20-25	1
LA2	Clarias sp.	cranial roof fragment	1	60-70	1
LA2	Clarias sp.	opercular	1	30-40	1
LA2	Clarias sp.	caudal vertebra	1	40-50	1
LA2	Clarias sp.	caudal vertebra	1	50-60	1
LA2	Clarias sp.	ceratohyale	1	30-40	1
LA2	Clarias sp.	vomerine toothplate	1	50-60	1
LA2	Clarias sp.	cranial roof fragment	2	30-40	2
LA2	Clarias sp.	cranial roof fragment	2	60-70	2
LA2	Clarias sp.	cranial roof fragment	2		
LA2	Clarias sp.	caudal vertebra	1	60-70	1
LA2	Clarias sp.	cranial roof fragment	1	60-70	1
LA2	Clarias sp.	precaudal vertebra	1	60-70	1
LA2	Clarias sp.	premaxilla	1	70-80	1
LA2	Clarias sp.	quadrate	1	40-50	1
LA2	Clarias sp.	caudal vertebra	2	50-60	2
LA2	Clarias sp.	cranial roof fragment	2		
LA2	Haplotilapiini indet.	skull roof fragment	1	20-25	1
LA2	Haplotilapiini indet.	skull roof fragment	1		
LA2	Haplotilapiini indet.	fin spine	1		

cultural phase	taxon	skeletal element	NISP	SL in cm	number
LA2	Haplotilapiini indet.	cleithrum	2	25-30	2
LA2	Clarias sp.	caudal vertebra	1	30-40	1
LA2	Clarias sp.	cranial roof fragment	1	40-50	1
LA2	Clarias sp.	cranial roof fragment	1	70-80	1
LA2	Clarias sp.	cranial roof fragment	1		
LA2	Clarias sp.	quadrate	1	60-70	1
LA2	Clarias sp.	cranial roof fragment	2	30-40	2
LA2	Haplotilapiini indet.	skull roof fragment	1		
LA2	Haplotilapiini indet.	cleithrum	2		
LA2	Clarias sp.	coracoid	1		
LA2	Clarias sp.	palatinum	1	90-100	1
LA2	Clarias sp.	cranial roof fragment	2		
LA2	Haplotilapiini indet.	basioccipital	1	20-25	1
LA2	O. niloticus	hyomandibula	1	20-25	1
LA2	O. niloticus	mesethmoid	1	25-30	1
LA2	Haplotilapiini indet.	precaudal vertebra	1	15-20	1
LA2	Haplotilapiini indet.	supracleithrum	1	25-30	1
LA2	Haplotilapiini indet.	neurocranium fragment	2		
LA2	Haplotilapiini indet.	caudal vertebra	3	15-20	3
LA2	Haplotilapiini indet.	cleithrum	4		
LA2	Clarias sp.	cleithrum	1		
LA2	Clarias sp.	coracoid	1		
LA2	Clarias sp.	pectoral spine	1	40-50	1
LA2	Clarias sp.	cranial roof fragment	3		
LA2	Haplotilapiini indet.	cleithrum	1		
LA2	Haplotilapiini indet.	cleithrum	1		
LA2	Haplotilapiini indet.	fin spine	1		
LA2	O. niloticus	mesethmoid	1	15-20	1
LA2	Haplotilapiini indet.	precaudal vertebra	1	15-20	1
LA2	Haplotilapiini indet.	preopercular	1		
LA2	Haplotilapiini indet.	3rd precaudal vertebra	1	20-25	1
LA2	Haplotilapiini indet.	dorsal or anal pterygophore	1	20-25	1
LA2	Haplotilapiini indet.	neurocranium fragment	2		
LA2	Clarias sp.	cleithrum	1	60-70	1
LA2	Clarias sp.	cleithrum	1		
LA2	Clarias sp.	cranial roof fragment	1	40-50	1
LA2	Clarias sp.	cranial roof fragment	1		
LA2	Clarias sp.	coracoid	2		
LA2	Haplotilapiini indet.	caudal vertebra	1	15-20	1
LA2	Haplotilapiini indet.	cleithrum	1		
LA2	Haplotilapiini indet.	skull roof fragment	1		
LA2	Haplotilapiini indet.	precaudal vertebra	1	15-20	1
LA2	Haplotilapiini indet.	2nd precaudal vertebra	1	25-30	1
LA2	Haplotilapiini indet.	dorsal or anal pterygophore	1		
LA2	Haplotilapiini indet.	supracleithrum	1	20-25	1
LA2	Clarias sp.	articular	1	40-50	1
LA2	Clarias sp.	cleithrum	1	40-50	1
LA2	Clarias sp.	cleithrum	1		
LA2	Clarias sp.	cranial roof fragment	1	30-40	1
LA2	Clarias sp.	cranial roof fragment	1	60-70	1
LA2	Clarias sp.	cranial roof fragment	1		
LA2	Clarias sp.	precaudal vertebra	1	50-60	1
LA2	Clarias sp.	quadrate	1	50-60	1
LA2	Clarias sp.	caudal vertebra	2	50-60	1
LA2	Haplotilapiini indet.	caudal vertebra	1	20-25	1
LA2	Haplotilapiini indet.	caudal vertebra	1	25-30	1
LA2	Haplotilapiini indet.	ceratohyale	1	20-25	1
LA2	Haplotilapiini indet.	cleithrum	1		

cultural phase	taxon	skeletal element	NISP	SL in cm	number
LA2	Haplotilapiini indet.	opercular	1		
LA2	Haplotilapiini indet.	precaudal vertebra	1	20-25	1
LA2	Haplotilapiini indet.	precaudal vertebra	1	25-30	1
LA2	Haplotilapiini indet.	dorsal or anal pterygophore	1	10-15	1
LA2	Haplotilapiini indet.	dorsal or anal pterygophore	1		
LA2	Clarias sp.	basioccipital	1	50-60	1
LA2	Clarias sp.	basioccipital	1	20-30	1
LA2	Clarias sp.	caudal vertebra	1	50-60	1
LA2	Clarias sp.	caudal vertebra	1	60-70	1
LA2	Clarias sp.	cleithrum	1		
LA2	Clarias sp.	cleithrum	1		
LA2	Clarias sp.	dentary	1	50-60	1
LA2	Clarias sp.	cranial roof fragment	1	30-40	1
LA2	Clarias sp.	cranial roof fragment	1		
LA2	Clarias sp.	mesethmoid	1	40-50	1
LA2	Clarias sp.	cranial roof fragment	2	60-70	2
LA2	Clarias sp.	cranial roof fragment	3		
LA2	Haplotilapiini indet.	cleithrum	1		
LA2	Haplotilapiini indet.	opercular	1	20-25	1
LA2	Haplotilapiini indet.	opercular	1	20-25	1
LA2	Haplotilapiini indet.	preopercular	1		
LA2	Haplotilapiini indet.	dorsal or anal pterygophore	1	20-25	1
LA2	Clarias sp.	hyomandibula	1		
LA2	Clarias sp.	palatinum	1	50-60	1
LA2	Clarias sp.	vomerine toothplate	1	40-50	1
LA2	Clarias sp.	cranial roof fragment	2	50-60	2
LA2	Clarias sp.	cranial roof fragment	5		
LA2	Haplotilapiini indet.	basipterygium	1	25-30	1
LA2	Haplotilapiini indet.	caudal vertebra	1	15-20	1
LA2	Haplotilapiini indet.	cleithrum	1		
LA2	Haplotilapiini indet.	dorsal or anal pterygophore	1	20-25	1
LA2	Haplotilapiini indet.	neurocranium fragment	2		
LA2	Haplotilapiini indet.	lepidotrich	2		
LA2	Clarias sp.	cranial roof fragment	1	40-50	1
LA2	Clarias sp.	precaudal vertebra	1	50-60	1
LA2	Clarias sp.	cranial roof fragment	2	30-40	2
LA2	Clarias sp.	cranial roof fragment	2		
LA2	Haplotilapiini indet.	basioccipital	1	25-30	1
LA2	Haplotilapiini indet.	basipterygium	1	15-20	1
LA2	Haplotilapiini indet.	basipterygium	1	25-30	1
LA2	Haplotilapiini indet.	fin spine	1		
LA2	Haplotilapiini indet.	precaudal vertebra	1	15-20	1
LA2	Haplotilapiini indet.	3rd precaudal vertebra	1	20-25	1
LA2	Haplotilapiini indet.	dorsal or anal pterygophore	1	20-25	1
LA2	Haplotilapiini indet.	dorsal or anal pterygophore	1		
LA2	Haplotilapiini indet.	skull roof fragment	4		
LA2	Clarias sp.	quadrate	1	40-50	1
LA2	Clarias sp.	unidentified	2		
LA2	Haplotilapiini indet.	fin spine	1		
LA2	O. niloticus	opercular	1	20-25	1
LA2	Haplotilapiini indet.	opercular	1	20-25	1
LA2	Haplotilapiini indet.	cleithrum	2	15-20	2
LA2	Clarias sp.	coracoid	1		
LA2	Clarias sp.	cranial roof fragment	1	30-40	1
LA2	Clarias sp.	precaudal vertebra	2	30-40	2
LA2	Clarias sp.	cranial roof fragment	3		
LA2	Haplotilapiini indet.	basipterygium	1	20-25	1
LA2	O. niloticus	dentary	1	20-25	1

cultural phase	taxon	skeletal element	NISP	SL in cm	number
LA2	O. niloticus	hyomandibula	1	15-20	1
LA2	Haplotilapiini indet.	fin spine	1		
LA2	Haplotilapiini indet.	mesethmoid	1	20-25	1
LA2	Haplotilapiini indet.	mesethmoid	1	20-25	1
LA2	Haplotilapiini indet.	opercular	1	20-25	1
LA2	Haplotilapiini indet.	opercular	1	20-25	1
LA2	Haplotilapiini indet.	precaudal vertebra	1	20-25	1
LA2	O. niloticus	premaxilla	1	20-25	1
LA2	Haplotilapiini indet.	preopercular	1		
LA2	Haplotilapiini indet.	3rd precaudal vertebra	1	20-25	1
LA2	Haplotilapiini indet.	dorsal or anal pterygophore	1		
LA2	Haplotilapiini indet.	caudal vertebra	2	15-20	2
LA2	Haplotilapiini indet.	postcleithrum	3	20-25	3
LA2	Haplotilapiini indet.	skull roof fragment	5		
LA2	Clarias sp.	caudal vertebra	1	50-60	1
LA2	Clarias sp.	mesethmoid	1	50-60	1
LA2	Clarias sp.	pectoral spine	1	70-80	1
LA2	Haplotilapiini indet.	cleithrum	1		
LA2	Clarias sp.	pectoral spine	1		
LA2	Clarias sp.	cranial roof fragment	2	40-50	2
LA2	Clarias sp.	cranial roof fragment	2		
LA2	Clarias sp.	cranial roof fragment	1		
LA2	Clarias sp.	caudal vertebra	2	60-70	2
LA2	Clarias sp.	cranial roof fragment	2	50-60	2
LA2	Haplotilapiini indet.	basioccipital	1	20-25	1
LA2	Haplotilapiini indet.	basipterygium	1	15-20	1
LA2	Haplotilapiini indet.	cleithrum	1		
LA2	Haplotilapiini indet.	skull roof fragment	1		
LA2	Clarias sp.	caudal vertebra	1	60-70	1
LA2	Clarias sp.	cleithrum	1		
LA2	Clarias sp.	cranial roof fragment	1	40-50	1
LA2	Clarias sp.	cranial roof fragment	1	60-70	1
LA2	Haplotilapiini indet.	skull roof fragment	1		
LA2	Haplotilapiini indet.	opercular	1	15-20	1
LA2	Haplotilapiini indet.	precaudal vertebra	1	15-20	1
LA2	Haplotilapiini indet.	dorsal or anal pterygophore	1	15-20	1
LA2	Haplotilapiini indet.	lepidotrich	2		
LA2	Clarias sp.	cranial roof fragment	1		
LA2	Clarias sp.	pectoral spine	1	50-60	1
LA2	Haplotilapiini indet.	cleithrum	1		
LA2	Haplotilapiini indet.	skull roof fragment	1		
LA2	Clarias sp.	articular	1	20-30	1
LA2	Clarias sp.	caudal vertebra	1	50-60	1
LA2	Clarias sp.	cranial roof fragment	3		
LA2	Haplotilapiini indet.	fin spine	1		
LA2	Haplotilapiini indet.	opercular	1	20-25	1
LA2	Haplotilapiini indet.	precaudal vertebra	1	15-20	1
LA2	Clarias sp.	basioccipital	1	70-80	1
LA2	Clarias sp.	ceratohyale	1	50-60	1
LA2	Clarias sp.	ceratohyale	1	60-70	1
LA2	Clarias sp.	cranial roof fragment	1	60-70	1
LA2	Clarias sp.	cranial roof fragment	2		
LA2	Haplotilapiini indet.	caudal vertebra	1	15-20	1
LA2	Haplotilapiini indet.	ceratohyale	1	15-20	1
LA2	Haplotilapiini indet.	cleithrum	1		
LA2	O. niloticus	hyomandibula	1	25-30	1
LA2	Haplotilapiini indet.	fin spine	1		
LA2	Haplotilapiini indet.	opercular	1	15-20	1

cultural phase	taxon	skeletal element	NISP	SL in cm	number
LA2	Haplotilapiini indet.	precaudal vertebra	1	15-20	1
LA2	Haplotilapiini indet.	precaudal vertebra	1	15-20	1
LA2	Haplotilapiini indet.	posttemporal	1	20-25	1
LA2	Haplotilapiini indet.	dorsal or anal pterygophore	1		
LA2	Clarias sp.	articular	1	50-60	1
LA2	Clarias sp.	articular	1		
LA2	Clarias sp.	basioccipital	1	10-20	1
LA2	Clarias sp.	caudal vertebra	1	50-60	1
LA2	Clarias sp.	caudal vertebra	1	60-70	1
LA2	Clarias sp.	ceratohyale	1	70-80	1
LA2	Clarias sp.	cleithrum	1		
LA2	Clarias sp.	cranial roof fragment	1		
LA2	Clarias sp.	cranial roof fragment	1		
LA2	Clarias sp.	fin ray	1		
LA2	Clarias sp.	mesethmoid	1	50-60	1
LA2	Clarias sp.	opercular	1	30-40	1
LA2	Clarias sp.	opercular	1	50-60	1
LA2	Clarias sp.	precaudal vertebra	1	50-60	1
LA2	Clarias sp.	precaudal vertebra	1	20-25	1
LA2	Clarias sp.	pterygophore	1		
LA2	Clarias sp.	urohyale	1	60-70	1
LA2	Clarias sp.	cranial roof fragment	4		
LA2	Haplotilapiini indet.	cleithrum	1		
LA2	Haplotilapiini indet.	opercular	1		
LA2	Clarias sp.	articular	1		
LA2	Clarias sp.	cranial roof fragment	1		
LA2	Clarias sp.	cleithrum	1		
LA2	Clarias sp.	coracoid	1		
LA2	Clarias sp.	cranial roof fragment	1	40-50	1
LA2	Haplotilapiini indet.	basipterygium	1	20-25	1
LA2	Haplotilapiini indet.	opercular	1		
LA2	Haplotilapiini indet.	precaudal vertebra	1	15-20	1
LA2	Clarias sp.	caudal vertebra	1	50-60	1
LA2	Clarias sp.	coracoid	1		
LA2	Clarias sp.	epihyale	1	40-50	1
LA2	Clarias sp.	epihyale	1	40-50	1
LA2	Clarias sp.	cranial roof fragment	1	40-50	1
LA2	Clarias sp.	cranial roof fragment	1	50-60	1
LA2	Clarias sp.	hyomandibula	1	60-70	1
LA2	Clarias sp.	hypohyale	1	40-50	1
LA2	Clarias sp.	opercular	1	30-40	1
LA2	Clarias sp.	vomerine toothplate	1	40-50	1
LA2	Clarias sp.	precaudal vertebra	2	50-60	2
LA2	Clarias sp.	caudal vertebra	3	60-70	3
LA2	Haplotilapiini indet.	caudal vertebra	1	15-20	1
LA2	Haplotilapiini indet.	2nd precaudal vertebra	1	20-25	1
LA2	Clarias sp.	caudal vertebra	1	30-40	1
LA2	Clarias sp.	caudal vertebra	1	30-40	1
LA2	Clarias sp.	caudal vertebra	1	40-50	1
LA2	Clarias sp.	precaudal vertebra	1	60-70	1
LA2	Clarias sp.	quadrate	1	20-30	1
LA2	Clarias sp.	urohyale	1	30-40	1
LA2	Clarias sp.	cranial roof fragment	2		
LA2	Clarias sp.	cranial roof fragment	2	40-50	2
LA2	Haplotilapiini indet.	caudal vertebra	1		
LA2	Haplotilapiini indet.	hyomandibula	1	25-30	1
LA2	Haplotilapiini indet.	opercular	1		
LA2	Haplotilapiini indet.	precaudal vertebra	1	15-20	1

cultural phase	taxon	skeletal element	NISP	SL in cm	number
LA2	Haplotilapiini indet.	dorsal or anal pterygophore	1		
LA2	Haplotilapiini indet.	dorsal or anal pterygophore	1	20-25	1
LA2	Haplotilapiini indet.	neurocranium fragment	2		
LA2	Haplotilapiini indet.	precaudal vertebra	2	15-20	2
LA2	Clarias sp.	articular	1	40-50	1
LA2	Clarias sp.	caudal vertebra	1	50-60	1
LA2	Clarias sp.	caudal vertebra	1	60-70	1
LA2	Clarias sp.	cleithrum	1	40-50	1
LA2	Clarias sp.	cleithrum	1		
LA2	Clarias sp.	coracoid	1		
LA2	Clarias sp.	epihyale	1	40-50	1
LA2	Clarias sp.	cranial roof fragment	1	40-50	1
LA2	Clarias sp.	cranial roof fragment	1	60-70	1
LA2	Clarias sp.	cranial roof fragment	1		
LA2	Clarias sp.	precaudal vertebra	1	50-60	1
LA2	Clarias sp.	quadrate	1	30-40	1
LA2	Clarias sp.	quadrate	1	30-40	1
LA2	Clarias sp.	caudal vertebra	2	60-70	2
LA2	Clarias sp.	cranial roof fragment	2		
LA2	Haplotilapiini indet.	basipterygium	1	20-25	1
LA2	Clarias sp.	articular	1	40-50	1
LA2	Clarias sp.	articular	1	40-50	1
LA2	Clarias sp.	articular	1	50-60	1
LA2	Clarias sp.	caudal vertebra	1	30-40	1
LA2	Clarias sp.	caudal vertebra	1	80-90	1
LA2	Clarias sp.	caudal vertebra	1	50-60	1
LA2	Clarias sp.	ceratohyale	1	40-50	1
LA2	Clarias sp.	ceratohyale	1	40-50	1
LA2	Clarias sp.	ceratohyale	1	40-50	1
LA2	Clarias sp.	cleithrum	1	40-50	1
LA2	Clarias sp.	cleithrum	1	50-60	1
LA2	Clarias sp.	cleithrum	1		
LA2	Clarias sp.	cleithrum	1	40-50	1
LA2	Clarias sp.	cleithrum	1		
LA2	Clarias sp.	coracoid	1	60-70	1
LA2	Clarias sp.	coracoid	1		
LA2	Clarias sp.	cranial roof fragment	1	30-40	1
LA2	Clarias sp.	cranial roof fragment	1		
LA2	Clarias sp.	cranial roof fragment	1		
LA2	Clarias sp.	cranial roof fragment	1	30-40	1
LA2	Clarias sp.	mesethmoid	1	40-50	1
LA2	Clarias sp.	precaudal vertebra	1	30-40	1
LA2	Clarias sp.	premaxilla	1	40-50	1
LA2	Clarias sp.	quadrate	1	40-50	1
LA2	Clarias sp.	quadrate	1	30-40	1
LA2	Clarias sp.	vomerine toothplate	1		
LA2	Clarias sp.	cranial roof fragment	2	30-40	2
LA2	Clarias sp.	cranial roof fragment	2	40-50	2
LA2	Clarias sp.	precaudal vertebra	2	60-70	2
LA2	Clarias sp.	cranial roof fragment	3		
LA2	Clarias sp.	cranial roof fragment	6		
LA2	Haplotilapiini indet.	basipterygium	1	15-20	1
LA2	Haplotilapiini indet.	caudal vertebra	1	20-25	1
LA2	Haplotilapiini indet.	caudal vertebra	1	25-30	1
LA2	Haplotilapiini indet.	ceratohyale	1	15-20	1
LA2	Haplotilapiini indet.	cleithrum	1		
LA2	Haplotilapiini indet.	cleithrum	1		
LA2	Haplotilapiini indet.	cleithrum	1		

cultural phase	taxon	skeletal element	NISP	SL in cm	number
LA2	Haplotilapiini indet.	skull roof fragment	1	15-20	1
LA2	Haplotilapiini indet.	skull roof fragment	1		
LA2	Haplotilapiini indet.	skull roof fragment	1		
LA2	Haplotilapiini indet.	skull roof fragment	1	15-20	1
LA2	Haplotilapiini indet.	skull roof fragment	1		
LA2	Haplotilapiini indet.	hyomandibula	1	15-20	1
LA2	Haplotilapiini indet.	hyomandibula	1		
LA2	Haplotilapiini indet.	fin spine	1		
LA2	O. niloticus	mesethmoid	1	25-30	1
LA2	O. niloticus	opercular	1	15-20	1
LA2	Haplotilapiini indet.	opercular	1	25-30	1
LA2	Haplotilapiini indet.	opercular	1		
LA2	Haplotilapiini indet.	opercular	1	20-25	1
LA2	Haplotilapiini indet.	precaudal vertebra	1	15-20	1
LA2	Haplotilapiini indet.	precaudal vertebra	1	20-25	1
LA2	Haplotilapiini indet.	precaudal vertebra	1	15-20	1
LA2	Haplotilapiini indet.	premaxilla	1	20-25	1
LA2	Haplotilapiini indet.	preopercular	1		
LA2	Haplotilapiini indet.	postcleithrum	1	15-20	1
LA2	O. niloticus	2nd precaudal vertebra	1	20-25	1
LA2	Haplotilapiini indet.	2nd precaudal vertebra	1	15-20	1
LA2	O. niloticus	3rd precaudal vertebra	1	15-20	1
LA2	Haplotilapiini indet.	dorsal or anal pterygophore	1	15-20	1
LA2	Haplotilapiini indet.	dorsal or anal pterygophore	1	15-20	1
LA2	Haplotilapiini indet.	caudal vertebra	2	15-20	2
LA2	Haplotilapiini indet.	cleithrum	2		
LA2	Haplotilapiini indet.	cleithrum	2		
LA2	Haplotilapiini indet.	neurocranium fragment	2		
LA2	Haplotilapiini indet.	neurocranium fragment	2		
LA2	Haplotilapiini indet.	neurocranium fragment	2	25-30	2
LA2	Clarias sp.	articular	1	40-50	1
LA2	Clarias sp.	cranial roof fragment	1		
LA2	Clarias sp.	hyomandibula	1	40-50	1
LA2	Clarias sp.	unidentified	1		
LA2	Clarias sp.	opercular	1		
LA2	Clarias sp.	pectoral spine	1	50-60	1
LA2	Haplotilapiini indet.	basioccipital	1	20-25	1
LA2	Haplotilapiini indet.	fin spine	1		
LA2	Haplotilapiini indet.	precaudal vertebra	1	20-25	1
LA2	Haplotilapiini indet.	2nd precaudal vertebra	1	20-25	1
LA2	Clarias sp.	cranial roof fragment	1	30-40	1
LA2	Clarias sp.	pectoral spine	1	40-50	1
LA2	Clarias sp.	caudal vertebra	1	30-40	1
LA2	Clarias sp.	caudal vertebra	1	60-70	1
LA2	Clarias sp.	cleithrum	1		
LA2	Clarias sp.	cranial roof fragment	1		
LA2	Clarias sp.	opercular	1	40-50	1
LA2	Clarias sp.	pectoral spine	1	50-60	1
LA2	Clarias sp.	cranial roof fragment	3		
LA2	Clarias sp.	cranial roof fragment	3		
LA2	Haplotilapiini indet.	skull roof fragment	1		
LA2	Haplotilapiini indet.	skull roof fragment	1	20-25	1
LA2	Haplotilapiini indet.	3rd precaudal vertebra	1	15-20	1
LA2	Haplotilapiini indet.	dorsal or anal pterygophore	1	15-20	1
LA2	Clarias sp.	caudal vertebra	1	10-20	1
LA2	Clarias sp.	caudal vertebra	1	40-50	1
LA2	Clarias sp.	caudal vertebra	1	20-30	1
LA2	Clarias sp.	caudal vertebra	1	50-60	1

cultural phase	taxon	skeletal element	NISP	SL in cm	number
LA2	Clarias sp.	ceratohyale	1	60-70	1
LA2	Clarias sp.	ceratohyale	1		
LA2	Clarias sp.	ceratohyale	1		
LA2	Clarias sp.	cleithrum	1	20-30	1
LA2	Clarias sp.	cleithrum	1	40-50	1
LA2	Clarias sp.	cleithrum	1		
LA2	Clarias sp.	cleithrum	1		
LA2	Clarias sp.	cleithrum	1	50-60	1
LA2	Clarias sp.	coracoid	1		
LA2	Clarias sp.	coracoid	1	50-60	1
LA2	Clarias sp.	cranial roof fragment	1		
LA2	Clarias sp.	cranial roof fragment	1		
LA2	Clarias sp.	cranial roof fragment	1	20-30	1
LA2	Clarias sp.	cranial roof fragment	1	50-60	1
LA2	Clarias sp.	cranial roof fragment	1	60-70	1
LA2	Clarias sp.	hyomandibula	1	30-40	1
LA2	Clarias sp.	opercular	1	40-50	1
LA2	Clarias sp.	pectoral spine	1	40-50	1
LA2	Clarias sp.	quadrate	1	20-30	1
LA2	Clarias sp.	vomerine toothplate	1	50-60	1
LA2	Clarias sp.	cranial roof fragment	2		
LA2	Clarias sp.	cranial roof fragment	9		
LA2	Haplotilapiini indet.	caudal vertebra	1	15-20	1
LA2	Haplotilapiini indet.	caudal vertebra	1	25-30	1
LA2	Haplotilapiini indet.	cleithrum	1		
LA2	Haplotilapiini indet.	skull roof fragment	1	15-20	1
LA2	Haplotilapiini indet.	skull roof fragment	1		
LA2	Haplotilapiini indet.	skull roof fragment	1		
LA2	Haplotilapiini indet.	fin spine	1		
LA2	Haplotilapiini indet.	fin spine	1		
LA2	O. niloticus	mesethmoid	1	15-20	1
LA2	Haplotilapiini indet.	opercular	1		
LA2	Haplotilapiini indet.	precaudal vertebra	2	15-20	2
LA2	Haplotilapiini indet.	precaudal vertebra	3	15-20	3
LA2	Clarias sp.	caudal vertebra	1	50-60	1
LA2	Clarias sp.	cranial roof fragment	1		
LA2	Clarias sp.	cranial roof fragment	1		
LA2	Clarias sp.	pectoral spine	1	50-60	1
LA2	Haplotilapiini indet.	basipterygium	1	15-20	1
LA2	Haplotilapiini indet.	caudal vertebra	1	15-20	1
LA2	Haplotilapiini indet.	cleithrum	1		
LA2	Haplotilapiini indet.	precaudal vertebra	1	15-20	1
LA2	Clarias sp.	vomerine toothplate	1		
LA2	Clarias sp.	cranial roof fragment	3		
LA2	Haplotilapiini indet.	skull roof fragment	1	20-25	1
LA2	Clarias sp.	caudal vertebra	1	40-50	1
LA2	Clarias sp.	cleithrum	1		
LA2	Clarias sp.	coracoid	1	50-60	1
LA2	Clarias sp.	coracoid	1		
LA2	Clarias sp.	cranial roof fragment	1		
LA2	Haplotilapiini indet.	basipterygium	1	15-20	1
LA2	Haplotilapiini indet.	caudal vertebra	1	15-20	1
LA2	Haplotilapiini indet.	skull roof fragment	1		
LA2	Haplotilapiini indet.	fin spine	1		
LA2	Haplotilapiini indet.	dorsal or anal pterygophore	1	15-20	1
LA2	Haplotilapiini indet.	cleithrum	2		
LA2	Clarias sp.	cranial roof fragment	1		
LA2	Haplotilapiini indet.	cleithrum	1		

cultural phase	taxon	skeletal element	NISP	SL in cm	number
LA2	Haplotilapiini indet.	hyomandibula	1	15-20	1
LA2	Haplotilapiini indet.	fin spine	1		
LA2	Haplotilapiini indet.	dorsal or anal pterygophore	1	15-20	1
LA2	Clarias sp.	cranial roof fragment	1	50-60	1
LA2	Clarias sp.	precaudal vertebra	1	50-60	1
LA2	Clarias sp.	pectoral spine	1	40-50	1
LA2	Clarias sp.	cranial roof fragment	3	30-40	3
LA2	Clarias sp.	cranial roof fragment	8		
LA2	Haplotilapiini indet.	articular	1	20-25	1
LA2	Haplotilapiini indet.	cleithrum	1		
LA2	Haplotilapiini indet.	skull roof fragment	1		
LA2	Haplotilapiini indet.	precaudal vertebra	1	15-20	1
LA2	Clarias sp.	articular	1	40-50	1
LA2	Clarias sp.	ceratohyale	1	30-40	1
LA2	Clarias sp.	cleithrum	1	40-50	1
LA2	Clarias sp.	cranial roof fragment	4		
LA2	Haplotilapiini indet.	basipterygium	1	20-25	1
LA2	Haplotilapiini indet.	fin spine	1		
LA2	Haplotilapiini indet.	dorsal or anal pterygophore	1		
LA2	Haplotilapiini indet.	neurocranium fragment	2		
LA2	Haplotilapiini indet.	cleithrum	4		
LA2	Clarias sp.	articular	1	90-100	1
LA2	Clarias sp.	caudal vertebra	1	40-50	1
LA2	Clarias sp.	ceratohyale	1	50-60	1
LA2	Clarias sp.	cleithrum	1		
LA2	Clarias sp.	coracoid	1		
LA2	Clarias sp.	cranial roof fragment	1	50-60	1
LA2	Clarias sp.	cranial roof fragment	1	40-50	1
LA2	Clarias sp.	cranial roof fragment	1		
LA2	Clarias sp.	mesethmoid	1	30-40	1
LA2	Clarias sp.	opercular	1	30-40	1
LA2	Clarias sp.	palatinum	1	50-60	1
LA2	Clarias sp.	pectoral spine	1	60-70	1
LA2	Clarias sp.	premaxilla	1	70-80	1
LA2	Clarias sp.	urohyale	1	40-50	1
LA2	Clarias sp.	caudal vertebra	2	40-50	2
LA2	Clarias sp.	cranial roof fragment	2	30-40	2
LA2	Clarias sp.	precaudal vertebra	2	50-60	2
LA2	Haplotilapiini indet.	caudal vertebra	1	15-20	1
LA2	Haplotilapiini indet.	caudal vertebra	1	20-25	1
LA2	Haplotilapiini indet.	caudal vertebra	1	25-30	1
LA2	Haplotilapiini indet.	ceratohyale	1	15-20	1
LA2	O. niloticus	hyomandibula	1	15-20	1
LA2	O. niloticus	hyomandibula	1	15-20	1
LA2	O. niloticus	hyomandibula	1	15-20	1
LA2	O. niloticus	mesethmoid	1	20-25	1
LA2	O. niloticus	mesethmoid	1	20-25	1
LA2	Haplotilapiini indet.	palatinum	1	25-30	1
LA2	Haplotilapiini indet.	precaudal vertebra	1	20-25	1
LA2	Haplotilapiini indet.	preopercular	1		
LA2	Haplotilapiini indet.	3rd precaudal vertebra	1	15-20	1
LA2	Haplotilapiini indet.	dorsal or anal pterygophore	1	15-20	1
LA2	Haplotilapiini indet.	dorsal or anal pterygophore	1	20-25	1
LA2	Haplotilapiini indet.	dorsal or anal pterygophore	1	20-25	1
LA2	Haplotilapiini indet.	dorsal or anal pterygophore	1	15-20	1
LA2	Haplotilapiini indet.	dorsal or anal pterygophore	1	15-20	1
LA2	Haplotilapiini indet.	dorsal or anal pterygophore	1		
LA2	Haplotilapiini indet.	supracleithrum	1	20-25	1

cultural phase	taxon	skeletal element	NISP	SL in cm	number
LA2	Haplotilapiini indet.	basipterygium	2	15-20	2
LA2	Haplotilapiini indet.	cleithrum	2		
LA2	Haplotilapiini indet.	neurocranium fragment	2		
LA2	Haplotilapiini indet.	lepidotrich	3		
LA2	Clarias sp.	articular	1	60-70	1
LA2	Clarias sp.	caudal vertebra	1	30-40	1
LA2	Clarias sp.	caudal vertebra	1	40-50	1
LA2	Clarias sp.	caudal vertebra	1	60-70	1
LA2	Clarias sp.	cleithrum	1	40-50	1
LA2	Clarias sp.	cleithrum	1	30-40	1
LA2	Clarias sp.	cleithrum	1		
LA2	Clarias sp.	coracoid	1		
LA2	Clarias sp.	epihyale	1	60-70	1
LA2	Clarias sp.	cranial roof fragment	1	30-40	1
LA2	Clarias sp.	cranial roof fragment	1	60-70	1
LA2	Clarias sp.	hyomandibula	1	30-40	1
LA2	Clarias sp.	precaudal vertebra	1	40-50	1
LA2	Clarias sp.	precaudal vertebra	1	50-60	1
LA2	Clarias sp.	pectoral spine	1	30-40	1
LA2	Clarias sp.	pectoral spine	1	40-50	1
LA2	Clarias sp.	cranial roof fragment	2	20-30	2
LA2	Clarias sp.	cranial roof fragment	9		
LA2	Haplotilapiini indet.	basipterygium	1	15-20	1
LA2	Haplotilapiini indet.	opercular	1	20-25	1
LA2	Haplotilapiini indet.	opercular	1	15-20	1
LA2	Haplotilapiini indet.	precaudal vertebra	1	25-30	1
LA2	Haplotilapiini indet.	preopercular	1		
LA2	Haplotilapiini indet.	3rd precaudal vertebra	1	25-30	1
LA2	Haplotilapiini indet.	dorsal or anal pterygophore	1		
LA2	Haplotilapiini indet.	supracleithrum	1	20-25	1
LA2	Haplotilapiini indet.	cleithrum	3		
LA2	Haplotilapiini indet.	precaudal vertebra	3	15-20	3
LA2	Haplotilapiini indet.	lepidotrich	4		
LA2	Haplotilapiini indet.	skull roof fragment	5		
LA2	Haplotilapiini indet.	cleithrum	1		
LA2	Haplotilapiini indet.	precaudal vertebra	1	20-25	1
LA2	Haplotilapiini indet.	dorsal or anal pterygophore	1	20-25	1
LA2	Clarias sp.	cranial roof fragment	1		
LA2	Haplotilapiini indet.	cleithrum	1		
LA2	Haplotilapiini indet.	dorsal or anal pterygophore	1	20-25	1
LA2	Clarias sp.	caudal vertebra	1	40-50	1
LA2	Clarias sp.	caudal vertebra	1	60-70	1
LA2	Clarias sp.	ceratohyale	1	60-70	1
LA2	Clarias sp.	cleithrum	1		
LA2	Clarias sp.	coracoid	1		
LA2	Clarias sp.	cranial roof fragment	1		
LA2	Clarias sp.	hyomandibula	1	40-50	1
LA2	Clarias sp.	opercular	1	30-40	1
LA2	Clarias sp.	precaudal vertebra	1	60-70	1
LA2	Clarias sp.	pectoral spine	1	50-60	1
LA2	Clarias sp.	quadrate	1	40-50	1
LA2	Clarias sp.	urohyale	1	40-50	1
LA2	Clarias sp.	vomerine toothplate	1	50-60	1
LA2	Clarias sp.	cranial roof fragment	2	40-50	2
LA2	Clarias sp.	cranial roof fragment	2		
LA2	Haplotilapiini indet.	basipterygium	1	20-25	1
LA2	Haplotilapiini indet.	cleithrum	1		
LA2	Haplotilapiini indet.	skull roof fragment	1		

cultural phase	taxon	skeletal element	NISP	SL in cm	number
LA2	Haplotilapiini indet.	fin spine	1		
LA2	Haplotilapiini indet.	opercular	1	15-20	1
LA2	Haplotilapiini indet.	precaudal vertebra	1	15-20	1
LA2	Haplotilapiini indet.	precaudal vertebra	1	20-25	1
LA2	Haplotilapiini indet.	2nd precaudal vertebra	1	20-25	1
LA2	Haplotilapiini indet.	supracleithrum	1	20-25	1
LA2	Haplotilapiini indet.	cleithrum	2		
LA2	Haplotilapiini indet.	neurocranium fragment	2		
LA2	Clarias sp.	articular	1	30-40	1
LA2	Clarias sp.	caudal vertebra	1	30-40	1
LA2	Clarias sp.	caudal vertebra	1	40-50	1
LA2	Clarias sp.	ceratohyale	1	40-50	1
LA2	Clarias sp.	cranial roof fragment	1	50-60	1
LA2	Clarias sp.	hypohyale	1	40-50	1
LA2	Clarias sp.	branchial element	1		
LA2	Clarias sp.	precaudal vertebra	1	40-50	1
LA2	Clarias sp.	urohyale	1	40-50	1
LA2	Clarias sp.	caudal vertebra	2	60-70	2
LA2	Clarias sp.	cranial roof fragment	2	40-50	2
LA2	Clarias sp.	cranial roof fragment	4		
LA2	Haplotilapiini indet.	articular	1	20-25	1
LA2	Haplotilapiini indet.	basioccipital	1	20-25	1
LA2	Haplotilapiini indet.	basipterygium	1	15-20	1
LA2	Haplotilapiini indet.	basipterygium	1	20-25	1
LA2	Haplotilapiini indet.	caudal vertebra	1	20-25	1
LA2	Haplotilapiini indet.	cleithrum	1	20-25	1
LA2	Haplotilapiini indet.	skull roof fragment	1	15-20	1
LA2	O. niloticus	hyomandibula	1	20-25	1
LA2	O. niloticus	mesethmoid	1	15-20	1
LA2	Haplotilapiini indet.	opercular	1	10-15	1
LA2	Haplotilapiini indet.	opercular	1	20-25	1
LA2	Haplotilapiini indet.	precaudal vertebra	1	15-20	1
LA2	Haplotilapiini indet.	precaudal vertebra	1	25-30	1
LA2	Haplotilapiini indet.	postcleithrum	1	20-25	1
LA2	Haplotilapiini indet.	postcleithrum	1	25-30	1
LA2	Haplotilapiini indet.	2nd precaudal vertebra	1	20-25	1
LA2	Haplotilapiini indet.	cleithrum	2		
LA2	Haplotilapiini indet.	preopercular	2		
LA2	Haplotilapiini indet.	supracleithrum	2	20-25	2
LA2	Haplotilapiini indet.	skull roof fragment	4		
LA2	Haplotilapiini indet.	lepidotrich	5		
LA2	Clarias sp.	coracoid	1	50-60	1
LA2	Clarias sp.	cranial roof fragment	1	60-70	1
LA2	Clarias sp.	cranial roof fragment	1	40-50	1
LA2	Clarias sp.	cranial roof fragment	1	60-70	1
LA2	Clarias sp.	pectoral spine	1	50-60	1
LA2	Clarias sp.	vomerine toothplate	1	60-70	1
LA2	Clarias sp.	cranial roof fragment	2		
LA2	Clarias sp.	cranial roof fragment	3		
LA2	Clarias sp.	caudal vertebra	4	60-70	4
LA2	Haplotilapiini indet.	basipterygium	1	15-20	1
LA2	Haplotilapiini indet.	basipterygium	1	15-20	1
LA2	Haplotilapiini indet.	basipterygium	1	20-25	1
LA2	O. niloticus	hyomandibula	1	15-20	1
LA2	Haplotilapiini indet.	opercular	1	20-25	1
LA2	Haplotilapiini indet.	precaudal vertebra	1	25-30	1
LA2	Haplotilapiini indet.	cleithrum	2		
LA2	Haplotilapiini indet.	lepidotrich	4		

cultural phase	taxon	skeletal element	NISP	SL in cm	number
LA2	Haplotilapiini indet.	caudal vertebra	5	20-25	5
LA2	Haplotilapiini indet.	precaudal vertebra	7	20-25	7
LA2	Haplotilapiini indet.	dorsal or anal pterygophore	8		
LA2	Clarias sp.	cleithrum	1	30-40	1
LA2	Clarias sp.	cleithrum	1		
LA2	Clarias sp.	unidentified	1		
LA2	Clarias sp.	branchial element	1		
LA2	Clarias sp.	opercular	1	30-40	1
LA2	Clarias sp.	pectoral spine	1	60-70	1
LA2	Clarias sp.	pectoral spine	1	40-50	1
LA2	Clarias sp.	quadrate	1	30-40	1
LA2	Clarias sp.	quadrate	1	50-60	1
LA2	Clarias sp.	cranial roof fragment	2	40-50	2
LA2	Clarias sp.	cranial roof fragment	2		
LA2	Clarias sp.	cranial roof fragment	2	40-50	2
LA2	Clarias sp.	cranial roof fragment	3		
LA2	Clarias sp.	caudal vertebra	4	60-70	4
LA2	Haplotilapiini indet.	basipterygium	1	20-25	1
LA2	Haplotilapiini indet.	skull roof fragment	1	20-25	1
LA2	Haplotilapiini indet.	hyomandibula	1	20-25	1
LA2	Haplotilapiini indet.	fin spine	1		
LA2	Haplotilapiini indet.	preopercular	1		
LA2	O. niloticus	3rd precaudal vertebra	1	20-25	1
LA2	Haplotilapiini indet.	3rd precaudal vertebra	1	20-25	1
LA2	Haplotilapiini indet.	basipterygium	2	15-20	2
LA2	Haplotilapiini indet.	precaudal vertebra	3	10-15	1
LA2	Haplotilapiini indet.	skull roof fragment	4		
LA2	Haplotilapiini indet.	cleithrum	5		
LA2	Clarias sp.	coracoid	1	50-60	1
LA2	Clarias sp.	cranial roof fragment	1		
LA2	Clarias sp.	quadrate	1	50-60	1
LA2	Haplotilapiini indet.	cleithrum	1		
LA2	Haplotilapiini indet.	skull roof fragment	1	15-20	1
LA2	Haplotilapiini indet.	skull roof fragment	1		
LA2	O. niloticus	hyomandibula	1	15-20	1
LA2	Haplotilapiini indet.	opercular	1	15-20	1
LA2	Clarias sp.	articular	1	40-50	1
LA2	Clarias sp.	opercular	1	40-50	1
LA2	Clarias sp.	cranial roof fragment	3		
LA2	Haplotilapiini indet.	basipterygium	1	15-20	1
LA2	Haplotilapiini indet.	caudal vertebra	1	15-20	1
LA2	O. niloticus	hyomandibula	1	25-30	1
LA2	O. niloticus	hyomandibula	1	15-20	1
LA2	Haplotilapiini indet.	opercular	1	10-15	1
LA2	Haplotilapiini indet.	opercular	1	20-25	1
LA2	Haplotilapiini indet.	opercular	1	20-25	1
LA2	Haplotilapiini indet.	opercular	1	15-20	1
LA2	Haplotilapiini indet.	precaudal vertebra	1	15-20	1
LA2	Haplotilapiini indet.	precaudal vertebra	1	25-30	1
LA2	Haplotilapiini indet.	3rd precaudal vertebra	1	15-20	1
LA2	Haplotilapiini indet.	dorsal or anal pterygophore	1	20-25	1
LA2	Haplotilapiini indet.	dorsal or anal pterygophore	1	15-20	1
LA2	Haplotilapiini indet.	dorsal or anal pterygophore	1	15-20	1
LA2	Haplotilapiini indet.	subopercular	1	20-25	1
LA2	Haplotilapiini indet.	supracleithrum	1	25-30	1
LA2	Haplotilapiini indet.	skull roof fragment	12		
LA2	Haplotilapiini indet.	basipterygium	2	20-25	2
LA2	Haplotilapiini indet.	postcleithrum	2	20-25	2

cultural phase	taxon	skeletal element	NISP	SL in cm	number
LA2	Haplotilapiini indet.	dorsal or anal pterygophore	2		
LA2	Haplotilapiini indet.	supracleithrum	2	20-25	2
LA2	Haplotilapiini indet.	lepidotrich	3		
LA2	Haplotilapiini indet.	cleithrum	5		
LA2	Clarias sp.	caudal vertebra	1	50-60	1
LA2	Clarias sp.	cleithrum	1	40-50	1
LA2	Clarias sp.	cleithrum	1	30-40	1
LA2	Clarias sp.	cleithrum	1		
LA2	Clarias sp.	cranial roof fragment	1		
LA2	Haplotilapiini indet.	basioccipital	1	20-25	1
LA2	Haplotilapiini indet.	basipterygium	1	15-20	1
LA2	Haplotilapiini indet.	ceratohyale	1	15-20	1
LA2	Haplotilapiini indet.	cleithrum	1		
LA2	Haplotilapiini indet.	dentary	1	15-20	1
LA2	O. niloticus	hyomandibula	1	20-25	1
LA2	Haplotilapiini indet.	hyomandibula	1		
LA2	O. niloticus	mesethmoid	1	15-20	1
LA2	Haplotilapiini indet.	opercular	1	15-20	1
LA2	Haplotilapiini indet.	opercular	1	20-25	1
LA2	Haplotilapiini indet.	opercular	1	10-15	1
LA2	Haplotilapiini indet.	opercular	1	25-30	1
LA2	Haplotilapiini indet.	precaudal vertebra	1	20-25	1
LA2	Haplotilapiini indet.	postcleithrum	1	25-30	1
LA2	Haplotilapiini indet.	dorsal or anal pterygophore	1	15-20	1
LA2	Haplotilapiini indet.	caudal vertebra	3	15-20	3
LA2	Haplotilapiini indet.	preopercular	3		
LA2	Haplotilapiini indet.	precaudal vertebra	4	15-20	4
LA2	Haplotilapiini indet.	skull roof fragment	8		
LA2	Clarias sp.	articular	1	30-40	1
LA2	Clarias sp.	articular	1	30-40	1
LA2	Clarias sp.	basioccipital	1	40-50	1
LA2	Clarias sp.	caudal vertebra	1	50-60	1
LA2	Clarias sp.	cleithrum	1	20-30	1
LA2	Clarias sp.	cleithrum	1	30-40	1
LA2	Clarias sp.	cranial roof fragment	1	30-40	1
LA2	Clarias sp.	cranial roof fragment	1	40-50	1
LA2	Clarias sp.	cranial roof fragment	1	60-70	1
LA2	Clarias sp.	mesethmoid	1	40-50	1
LA2	Clarias sp.	caudal vertebra	3	60-70	3
LA2	Clarias sp.	cranial roof fragment	4		
LA2	Haplotilapiini indet.	articular	1	20-25	1
LA2	Haplotilapiini indet.	articular	1	25-30	1
LA2	Haplotilapiini indet.	caudal vertebra	1	15-20	1
LA2	Haplotilapiini indet.	cleithrum	1		
LA2	Haplotilapiini indet.	dentary	1	25-30	1
LA2	Haplotilapiini indet.	opercular	1	15-20	1
LA2	Haplotilapiini indet.	opercular	1	15-20	1
LA2	Haplotilapiini indet.	opercular	1		
LA2	Haplotilapiini indet.	preopercular	1		
LA2	Haplotilapiini indet.	3rd precaudal vertebra	1	20-25	1
LA2	Haplotilapiini indet.	3rd precaudal vertebra	1	20-25	1
LA2	Haplotilapiini indet.	dorsal or anal pterygophore	1	15-20	1
LA2	Haplotilapiini indet.	dorsal or anal pterygophore	1		
LA2	Haplotilapiini indet.	scapula	1	15-20	1
LA2	Haplotilapiini indet.	cleithrum	2	15-20	2
LA2	Haplotilapiini indet.	cleithrum	2	20-25	2
LA2	Haplotilapiini indet.	lepidotrich	2		
LA2	Haplotilapiini indet.	precaudal vertebra	5	15-20	5

cultural phase	taxon	skeletal element	NISP	SL in cm	number
LA2	Haplotilapiini indet.	skull roof fragment	6		
LA2	Clarias sp.	caudal vertebra	1	50-60	1
LA2	Clarias sp.	ceratohyale	1	50-60	1
LA2	Clarias sp.	coracoid	1	40-50	1
LA2	Clarias sp.	cranial roof fragment	1		
LA2	Haplotilapiini indet.	caudal vertebra	1	15-20	1
LA2	Haplotilapiini indet.	postcleithrum	1	15-20	1
LA2	Haplotilapiini indet.	2nd precaudal vertebra	1	15-20	1
LA2	Haplotilapiini indet.	dorsal or anal pterygophore	1	15-20	1
LA2	Haplotilapiini indet.	cleithrum	2		
LA2	Clarias sp.	cleithrum	1	30-40	1
LA2	Clarias sp.	cleithrum	1	50-60	1
LA2	Clarias sp.	hyomandibula	1	50-60	1
LA2	Clarias sp.	caudal vertebra	2	60-70	2
LA2	Clarias sp.	coracoid	2		
LA2	Clarias sp.	cranial roof fragment	2		
LA2	Clarias sp.	cranial roof fragment	2	40-50	2
LA2	Haplotilapiini indet.	caudal vertebra	1	15-20	1
LA2	Haplotilapiini indet.	cleithrum	1		
LA2	Haplotilapiini indet.	opercular	1	15-20	1
LA2	Haplotilapiini indet.	opercular	1		
LA2	Haplotilapiini indet.	dorsal or anal pterygophore	1	20-25	1
LA2	Haplotilapiini indet.	dorsal or anal pterygophore	1	20-25	1
LA2	Haplotilapiini indet.	skull roof fragment	3		
LA2	Clarias sp.	articular	1	50-60	1
LA2	Clarias sp.	basioccipital	1	40-50	1
LA2	Clarias sp.	caudal vertebra	1	50-60	1
LA2	Clarias sp.	cranial roof fragment	1	40-50	1
LA2	Clarias sp.	cranial roof fragment	1	50-60	1
LA2	Clarias sp.	cranial roof fragment	10		
LA2	Clarias sp.	premaxilla	2	50-60	2
LA2	Haplotilapiini indet.	basipterygium	1	20-25	1
LA2	Haplotilapiini indet.	fin spine	1		
LA2	Haplotilapiini indet.	precaudal vertebra	1	15-20	1
LA2	Haplotilapiini indet.	preopercular	1		
LA2	Haplotilapiini indet.	dorsal or anal pterygophore	1	20-25	1
LA2	Haplotilapiini indet.	dorsal or anal pterygophore	1	15-20	1
LA2	Haplotilapiini indet.	dorsal or anal pterygophore	1	15-20	1
LA2	Haplotilapiini indet.	supracleithrum	1	15-20	1
LA2	Haplotilapiini indet.	supracleithrum	1	20-25	1
LA2	Haplotilapiini indet.	cleithrum	3		
LA2	Haplotilapiini indet.	skull roof fragment	6		
LA2	Clarias sp.	basioccipital	1	40-50	1
LA2	Clarias sp.	cranial roof fragment	1	30-40	1
LA2	Clarias sp.	cranial roof fragment	1		
LA2	O. niloticus	cleithrum	1		
LA2	Haplotilapiini indet.	postcleithrum	1	15-20	1
LA2	Clarias sp.	basioccipital	1	50-60	1
LA2	Clarias sp.	basioccipital	1	20-30	1
LA2	Clarias sp.	caudal vertebra	1	40-50	1
LA2	Clarias sp.	caudal vertebra	1	50-60	1
LA2	Clarias sp.	caudal vertebra	1	60-70	1
LA2	Clarias sp.	caudal vertebra	1		
LA2	Clarias sp.	ceratohyale	1	60-70	1
LA2	Clarias sp.	ceratohyale	1	40-50	1
LA2	Clarias sp.	ceratohyale	1	60-70	1
LA2	Clarias sp.	cleithrum	1	30-40	1
LA2	Clarias sp.	cleithrum	1		

cultural phase	taxon	skeletal element	NISP	SL in cm	number
LA2	Clarias sp.	hyomandibula	1	50-60	1
LA2	Clarias sp.	hypohyale	1	30-40	1
LA2	Clarias sp.	mesethmoid	1	50-60	1
LA2	Clarias sp.	precaudal vertebra	1	40-50	1
LA2	Clarias sp.	pectoral spine	1	40-50	1
LA2	Clarias sp.	pectoral spine	1	60-70	1
LA2	Clarias sp.	cranial roof fragment	16		
LA2	Clarias sp.	cranial roof fragment	3	50-60	3
LA2	Haplotilapiini indet.	mesethmoid	1	15-20	1
LA2	Haplotilapiini indet.	opercular	1	15-20	1
LA2	Haplotilapiini indet.	opercular	1		
LA2	Haplotilapiini indet.	precaudal vertebra	1	20-25	1
LA2	Haplotilapiini indet.	preopercular	1		
LA2	Haplotilapiini indet.	3rd precaudal vertebra	1	25-30	1
LA2	Haplotilapiini indet.	dorsal or anal pterygophore	1	15-20	1
LA2	O. niloticus	quadrate	1	20-25	1
LA2	Haplotilapiini indet.	cleithrum	2		
LA2	Haplotilapiini indet.	opercular	2		
LA2	Haplotilapiini indet.	precaudal vertebra	2	15-20	2
LA2	Haplotilapiini indet.	supracleithrum	2	15-20	2
LA2	Haplotilapiini indet.	supracleithrum	2	25-30	2
LA2	Haplotilapiini indet.	caudal vertebra	4	15-20	4
LA2	Haplotilapiini indet.	skull roof fragment	4		
LA2	Clarias sp.	coracoid	1	40-50	1
LA2	Clarias sp.	cranial roof fragment	1	20-30	1
LA2	Clarias sp.	cranial roof fragment	1	30-40	1
LA2	Clarias sp.	cranial roof fragment	1	40-50	1
LA2	Clarias sp.	branchial element	1		
LA2	Haplotilapiini indet.	basipterygium	1	15-20	1
LA2	Haplotilapiini indet.	caudal vertebra	1	15-20	1
LA2	Haplotilapiini indet.	cleithrum	1		
LA2	O. niloticus	hyomandibula	1	20-25	1
LA2	Haplotilapiini indet.	maxilla	1	25-30	1
LA2	O. niloticus	mesethmoid	1	25-30	1
LA2	Haplotilapiini indet.	palatinum	1	15-20	1
LA2	Haplotilapiini indet.	dorsal or anal pterygophore	1	10-15	1
LA2	Haplotilapiini indet.	supracleithrum	1	20-25	1
LA2	Haplotilapiini indet.	skull roof fragment	3		
LA2	Haplotilapiini indet.	precaudal vertebra	3	15-20	3
LA2	Clarias sp.	caudal vertebra	1	50-60	1
LA2	Clarias sp.	hyomandibula	1	30-40	1
LA2	Haplotilapiini indet.	cleithrum	1		
LA2	Haplotilapiini indet.	precaudal vertebra	1	15-20	1
LA2	Clarias sp.	caudal vertebra	1	50-60	1
LA2	Haplotilapiini indet.	fin spine	1		
LA2	Haplotilapiini indet.	dorsal or anal pterygophore	1		
LA2	Haplotilapiini indet.	cleithrum	2		
LA2	Clarias sp.	ceratohyale	1	40-50	1
LA2	Clarias sp.	vomerine toothplate	1	30-40	1
LA2	Clarias sp.	caudal vertebra	2	50-60	2
LA2	Clarias sp.	cranial roof fragment	2	30-40	2
LA2	Clarias sp.	cranial roof fragment	3		
LA2	Haplotilapiini indet.	cleithrum	1		
LA2	Haplotilapiini indet.	fin spine	1		
LA2	Haplotilapiini indet.	dorsal or anal pterygophore	1	25-30	1
LA2	Haplotilapiini indet.	supracleithrum	1	15-20	1
LA2	Haplotilapiini indet.	supracleithrum	1	20-25	1
LA2	Clarias sp.	cranial roof fragment	1		

cultural phase	taxon	skeletal element	NISP	SL in cm	number
LA2	Clarias sp.	hypohyale	1	60-70	1
LA2	Haplotilapiini indet.	caudal vertebra	1	20-25	1
LA2	Haplotilapiini indet.	skull roof fragment	1		
LA2	Clarias sp.	caudal vertebra	1	60-70	1
LA2	Clarias sp.	caudal vertebra	1	60-70	1
LA2	Clarias sp.	coracoid	1		
LA2	Clarias sp.	coracoid	1		
LA2	Clarias sp.	cranial roof fragment	1	40-50	1
LA2	Clarias sp.	cranial roof fragment	1	60-70	1
LA2	Clarias sp.	cranial roof fragment	1	40-50	1
LA2	Clarias sp.	cranial roof fragment	1	50-60	1
LA2	Clarias sp.	cranial roof fragment	1		
LA2	Clarias sp.	mesethmoid	1	40-50	1
LA2	Clarias sp.	mesethmoid	1	40-50	1
LA2	Clarias sp.	pectoral spine	1	40-50	1
LA2	Clarias sp.	pectoral spine	1	50-60	1
LA2	Clarias sp.	pectoral spine	1	60-70	1
LA2	Clarias sp.	pectoral spine	1	60-70	1
LA2	Clarias sp.	pectoral spine	1	40-50	1
LA2	Clarias sp.	pectoral spine	1	50-60	1
LA2	Haplotilapiini indet.	cleithrum	1		
LA2	Haplotilapiini indet.	fin spine	1		
LA2	Haplotilapiini indet.	fin spine	1		
LA2	Haplotilapiini indet.	opercular	1	20-25	1
LA2	Haplotilapiini indet.	opercular	1		
LA2	Haplotilapiini indet.	opercular	1	20-25	1
LA2	Haplotilapiini indet.	opercular	1		
LA2	Haplotilapiini indet.	precaudal vertebra	1	15-20	1
LA2	Haplotilapiini indet.	precaudal vertebra	1	15-20	1
LA2	Haplotilapiini indet.	dorsal or anal pterygophore	1	20-25	1
LA2	Haplotilapiini indet.	dorsal or anal pterygophore	1	15-20	1
LA2	Haplotilapiini indet.	dorsal or anal pterygophore	1	15-20	1
LA2	Haplotilapiini indet.	dorsal or anal pterygophore	1	15-20	1
LA2	Haplotilapiini indet.	dorsal or anal pterygophore	1	20-25	1
LA2	Haplotilapiini indet.	dorsal or anal pterygophore	1	15-20	1
LA2	Haplotilapiini indet.	quadrate	1	20-25	1
LA2	Haplotilapiini indet.	quadrate	1	20-25	1
LA2	Haplotilapiini indet.	supracleithrum	1	15-20	1
LA2	Haplotilapiini indet.	supracleithrum	1	15-20	1
LA2	Haplotilapiini indet.	dorsal or anal pterygophore	2		
LA2	Haplotilapiini indet.	skull roof fragment	3		
LA2	Haplotilapiini indet.	skull roof fragment	3		
LA2	Clarias sp.	cleithrum	1		
LA2	Clarias sp.	coracoid	1	50-60	1
LA2	Clarias sp.	epihyale	1	40-50	1
LA2	Clarias sp.	cranial roof fragment	1	40-50	1
LA2	Clarias sp.	hyomandibula	1		
LA2	Clarias sp.	caudal vertebra	3	60-70	3
LA2	Haplotilapiini indet.	neurocranium fragment	2		
LA2	Haplotilapiini indet.	cleithrum	3		
LA2	Clarias sp.	cranial roof fragment	2		
LA2	Clarias sp.	caudal vertebra	1		
LA2	Clarias sp.	cranial roof fragment	1		
LA2	Haplotilapiini indet.	skull roof fragment	1		
LA2	Haplotilapiini indet.	precaudal vertebra	1	20-25	1
LA2	Haplotilapiini indet.	cleithrum	2		
LA2	Clarias sp.	caudal vertebra	1		
LA2	Clarias sp.	caudal vertebra	1	20-25	1

cultural phase	taxon	skeletal element	NISP	SL in cm	number
LA2	Clarias sp.	pectoral spine	1	60-70	1
LA2	Clarias sp.	precaudal vertebra	2	20-25	1
LA2	Haplotilapiini indet.	precaudal vertebra	1	25-30	1
LA2	Clarias sp.	coracoid	1	60-70	1
LA2	Clarias sp.	pectoral spine	1	60-70	1
LA2	Clarias sp.	cranial roof fragment	2		
LA2	Clarias sp.	caudal vertebra	1	60-70	1
LA2	Clarias sp.	cleithrum	1	40-50	1
LA2	Clarias sp.	coracoid	1	50-60	1
LA2	Clarias sp.	dentary	1	60-70	1
LA2	Clarias sp.	cranial roof fragment	1	40-50	1
LA2	Clarias sp.	cranial roof fragment	1	60-70	1
LA2	Clarias sp.	cranial roof fragment	1	70-80	1
LA2	Clarias sp.	precaudal vertebra	1	30-40	1
LA2	Clarias sp.	pectoral spine	1	70-80	1
LA2	Clarias sp.	quadrate	1	30-40	1
LA2	Clarias sp.	cranial roof fragment	7		
LA2	Haplotilapiini indet.	cleithrum	1		
LA2	Haplotilapiini indet.	fin spine	1		
LA2	Haplotilapiini indet.	precaudal vertebra	1	20-25	1
LA2	Clarias sp.	articular	1	40-50	1
LA2	Clarias sp.	caudal vertebra	1	60-70	1
LA2	Clarias sp.	caudal vertebra	1	40-50	1
LA2	Clarias sp.	caudal vertebra	1	50-60	1
LA2	Clarias sp.	cleithrum	1		
LA2	Clarias sp.	cleithrum	1		
LA2	Clarias sp.	coracoid	1		
LA2	Clarias sp.	epihyale	1	70-80	1
LA2	Clarias sp.	epi- & ceratohyale	1	40-50	1
LA2	Clarias sp.	opercular	1	50-60	1
LA2	Clarias sp.	precaudal vertebra	1	40-50	1
LA2	Clarias sp.	precaudal vertebra	1		
LA2	Clarias sp.	caudal vertebra	2		
LA2	Clarias sp.	caudal vertebra	2	60-70	1
LA2	Clarias sp.	cranial roof fragment	2		
LA2	Clarias sp.	cranial roof fragment	6		
LA2	Clarias sp.	cranial roof fragment	8		
LA2	Haplotilapiini indet.	caudal vertebra	1		
LA2	Haplotilapiini indet.	caudal vertebra	1		
LA2	C. gariepinus	vomerine toothplate	1		
LA2	Clarias sp.	articular	1	60-70	1
LA2	Clarias sp.	articular & hyomandibular	1	30-40	1
LA2	Clarias sp.	ceratohyale	1	50-60	1
LA2	Clarias sp.	cleithrum	1	40-50	1
LA2	Clarias sp.	coracoid	1	30-40	1
LA2	Clarias sp.	dentary	1	30-40	1
LA2	Clarias sp.	epihyale	1	50-60	1
LA2	Clarias sp.	hyomandibula	1	50-60	1
LA2	Clarias sp.	mesethmoid	1	70-80	1
LA2	Clarias sp.	mesethmoid	1	70-80	1
LA2	Clarias sp.	cranial roof fragment	1	60-70	1
LA2	Clarias sp.	cranial roof fragment	1		
LA2	Clarias sp.	precaudal vertebra	1	40-50	1
LA2	Clarias sp.	precaudal vertebra	1	60-70	1
LA2	Clarias sp.	precaudal vertebra	1	70-80	1
LA2	Clarias sp.	pectoral spine	1	40-50	1
LA2	Clarias sp.	pectoral spine	1	40-50	1
LA2	Clarias sp.	pectoral spine	1	50-60	1

cultural phase	taxon	skeletal element	NISP	SL in cm	number
LA2	Clarias sp.	pectoral spine	1	60-70	1
LA2	Clarias sp.	pectoral spine	1	70-80	1
LA2	Clarias sp.	quadrate	1	40-50	1
LA2	Clarias sp.	urohyale	1	40-50	1
LA2	Clarias sp.	urohyale	1	40-50	1
LA2	Clarias sp.	cranial roof fragment	16		
LA2	Clarias sp.	coracoid	2	40-50	1
LA2	Clarias sp.	coracoid	2	50-60	1
LA2	Clarias sp.	cranial roof fragment	2		
LA2	Clarias sp.	caudal vertebra	3	60-70	1
LA2	Clarias sp.	branchial element	3		
LA2	Clarias sp.	caudal vertebra	1		
LA2	Clarias sp.	cleithrum	1	30-40	1
LA2	Clarias sp.	precaudal vertebra	1	50-60	1
LA2	Haplotilapiini indet.	anal pterygophore	1	15-20	1
LA2	Haplotilapiini indet.	cleithrum	1		
LA2	Haplotilapiini indet.	unidentified	1		
LA2	O. niloticus	cleithrum	1	20-25	1
LA2	Haplotilapiini indet.	dorsal or anal pterygophore	1		
LA2	C. gariepinus	vomerine toothplate	1	50-60	1
LA2	Clarias sp.	caudal vertebra	1		
LA2	Clarias sp.	caudal vertebra	1	50-60	1
LA2	Clarias sp.	caudal vertebra	1	60-70	1
LA2	Clarias sp.	cranial roof fragment	1		
LA2	Clarias sp.	cranial roof fragment	1		
LA2	Clarias sp.	cleithrum	2	40-50	1
LA2	Haplotilapiini indet.	cleithrum	1	20-25	1
LA2	Clarias sp.	coracoid	1	30-40	1
LA2	Clarias sp.	coracoid	1		
LA2	Clarias sp.	cranial roof fragment	1	70-80	1
LA2	Haplotilapiini indet.	caudal vertebra	1	20-25	1
LA2	Haplotilapiini indet.	cleithrum	1	15-20	1
LA2	O. niloticus	cleithrum	1	20-25	1
LA2	Haplotilapiini indet.	opercular	1	15-20	1
LA2	Haplotilapiini indet.	opercular	1	20-25	1
LA2	Haplotilapiini indet.	preopercular	1	20-25	1
LA2	Haplotilapiini indet.	postcleithrum	1	20-25	1
LA2	Haplotilapiini indet.	1st precaudal vertebra	1	20-25	1
LA2	O. niloticus	1st precaudal vertebra	1	15-20	1
LA2	Haplotilapiini indet.	1st precaudal vertebra	1	30-40	1
LA2	C. zillii	1st precaudal vertebra	1		
LA2	O. niloticus	1st precaudal vertebra	1	20-25	1
LA2	C. zillii	1st precaudal vertebra	1	20-25	1
LA2	Haplotilapiini indet.	1st precaudal vertebra	1	20-25	1
LA2	Haplotilapiini indet.	1st precaudal vertebra	1	25-30	1
LA2	Clarias sp.	precaudal vertebra	1	30-40	1
LA2	Clarias sp.	quadrate	1	40-50	1
LA2	Clarias sp.	cleithrum	1	40-50	1
LA2	Clarias sp.	dentary	1	50-60	1
LA2	Clarias sp.	cranial roof fragment	1		
LA2	Clarias sp.	pectoral spine	1	40-50	1
LA2	Clarias sp.	cranial roof fragment	3	30-40	1
LA2	Clarias sp.	precaudal vertebra	1	40-50	1
LA2	Clarias sp.	caudal vertebra	2	40-50	2
LA2	Clarias sp.	cleithrum	2		
LA2	Clarias sp.	caudal vertebra	2	60-70	2
LA2	Clarias sp.	ceratohyale	1	30-40	1
LA2	Clarias sp.	cleithrum	1	30-40	

cultural phase	taxon	skeletal element	NISP	SL in cm	number
LA2	Clarias sp.	coracoid	1	30-40	1
LA2	Clarias sp.	caudal vertebra	1	30-40	1
LA2	Clarias sp.	hyomandibula	1	40-50	1
LA2	Clarias sp.	cranial roof fragment	1	30-40	1
LA2	Clarias sp.	cranial roof fragment	2		
LA2	Clarias sp.	caudal vertebra	1	70-80	1
LA2	Clarias sp.	ceratohyale	1	70-80	1
LA2	Clarias sp.	cranial roof fragment	1	70-80	1
LA2	Clarias sp.	precaudal vertebra	1	70-80	1
LA2	Clarias sp.	caudal vertebra	1	30-40	1
LA2	Clarias sp.	cleithrum	1		
LA2	Clarias sp.	cranial roof fragment	1		
LA2	Clarias sp.	ceratohyale	1	40-50	1
LA2	Clarias sp.	mesethmoid	1	60-70	1
LA2	Clarias sp.	caudal vertebra	3	50-60	3
LA2	Clarias sp.	precaudal vertebra	1	60-70	1
LA2	Clarias sp.	pectoral spine	1	40-50	1
LA2	Clarias sp.	cranial roof fragment	4	20-30	1
LA2	Clarias sp.	caudal vertebra	1	15-20	1
LA2	Clarias sp.	cleithrum	1	50-60	1
LA2	Clarias sp.	cleithrum	1		
LA2	Clarias sp.	dentary	1		
LA2	Clarias sp.	branchial element	1	20-25	1
LA2	Clarias sp.	pectoral spine	1	40-50	1
LA2	Clarias sp.	cranial roof fragment	8		
LA2	Clarias sp.	cranial roof fragment	2	60-70	1
LA2	Clarias sp.	basioccipital	1	40-50	1
LA2	Clarias sp.	ceratohyale	1		
LA2	Clarias sp.	quadrate	1	30-40	1
LA2	Clarias sp.	caudal vertebra	2	60-70	2
LA2	Clarias sp.	cranial roof fragment	2		
LA2	Clarias sp.	cranial roof fragment	4	20-30	1
LA2	Clarias sp.	cranial roof fragment	2	50-60	1
LA2	Clarias sp.	cranial roof fragment	2		
LA2	Clarias sp.	articular	1	40-50	1
LA2	Clarias sp.	cranial roof fragment	1		
LA2	Clarias sp.	vertebra	3	40-50	3
LA2	Clarias sp.	caudal vertebra	2	30-40	2
LA2	Clarias sp.	cranial roof fragment	3	30-40	1
LA2	Clarias sp.	cleithrum	1	50-60	1
LA2	Clarias sp.	cranial roof fragment	3		
LA2	Clarias sp.	caudal vertebra	1	50-60	1
LA2	Clarias sp.	cleithrum	1		
LA2	Clarias sp.	caudal vertebra	1	40-50	1
LA2	Clarias sp.	pectoral spine	1	40-50	1
LA2	Clarias sp.	cranial roof fragment	2		
LA2	Haplotilapiini indet.	basioccipital	1	25-30	1
LA2	Haplotilapiini indet.	caudal vertebra	1	15-20	1
LA2	Haplotilapiini indet.	skull roof fragment	2		
LA2	Haplotilapiini indet.	caudal vertebra	1	25-30	1
LA2	Haplotilapiini indet.	opercular	1		
LA2	Haplotilapiini indet.	caudal vertebra	2	15-20	2
LA2	Haplotilapiini indet.	cleithrum	1		
LA2	O. niloticus	mesethmoid	1	20-25	1
LA2	Haplotilapiini indet.	precaudal vertebra	1	25-30	1
LA2	Haplotilapiini indet.	dorsal or anal pterygophore	1	15-20	1
LA2	Haplotilapiini indet.	skull roof fragment	1		
LA2	Haplotilapiini indet.	preopercular	1	25-30	1

cultural phase	taxon	skeletal element	NISP	SL in cm	number
LA2	Haplotilapiini indet.	skull roof fragment	1		
LA2	Haplotilapiini indet.	precaudal vertebra	1	15-20	1
LA2	Haplotilapiini indet.	basipterygium	1	25-30	1
LA2	Haplotilapiini indet.	cleithrum	1		
LA2	Haplotilapiini indet.	precaudal vertebra	1	20-25	1
LA2	Haplotilapiini indet.	basioccipital	1	20-25	1
LA2	Haplotilapiini indet.	caudal vertebra	1	15-20	1
LA2	Haplotilapiini indet.	caudal vertebra	1	20-25	1
LA2	O. niloticus	mesethmoid	1	15-20	1
LA2	Haplotilapiini indet.	opercular	1	20-25	1
LA2	Haplotilapiini indet.	2nd precaudal vertebra	1	15-20	1
LA2	Haplotilapiini indet.	postcleithrum	1		
LA2	Haplotilapiini indet.	lepidotrich	2		
LA2	Haplotilapiini indet.	cleithrum	5		
LA2	Haplotilapiini indet.	precaudal vertebra	5	15-20	5
LA2	Haplotilapiini indet.	cleithrum	1	15-20	1
LA2	O. niloticus	hyomandibula	1	20-25	1
LA2	O. niloticus	hyomandibula	1	20-25	1
LA2	Haplotilapiini indet.	skull roof fragment	1		
LA2	Haplotilapiini indet.	basipterygium	1	20-25	1
LA2	Haplotilapiini indet.	opercular	1	15-20	1
LA2	Haplotilapiini indet.	precaudal vertebra	1	15-20	1
LA2	Haplotilapiini indet.	preopercular	1		
LA2	Haplotilapiini indet.	dorsal or anal pterygophore	1	15-20	1
LA2	Haplotilapiini indet.	dorsal or anal pterygophore	1	15-20	1
LA2	Haplotilapiini indet.	dorsal or anal pterygophore	1	15-20	1
LA2	Haplotilapiini indet.	lepidotrich	2		
LA2	Haplotilapiini indet.	skull roof fragment	2		
LA2	Haplotilapiini indet.	dorsal or anal pterygophore	2		
LA2	Haplotilapiini indet.	cleithrum	3		
LA2	Haplotilapiini indet.	caudal vertebra	4	15-20	4
LA2	O. niloticus	1st precaudal vertebra	1	30-40	1
LA2	O. niloticus	1st precaudal vertebra	1	15-20	1
LA2	O. niloticus	1st precaudal vertebra	1	15-20	1
LA2	Clarias sp.	basioccipital	1	20-30	1
LA2	Clarias sp.	basioccipital	1	20-30	1
LA2	Clarias sp.	cranial roof fragment	2		
LA2	Clarias sp.	cranial roof fragment	4	40-50	1
LA2	Clarias sp.	cleithrum	1		
LA2	Clarias sp.	coracoid	1		
LA2	Clarias sp.	dentary	1	30-40	1
LA2	Clarias sp.	hyomandibula	1	20-30	1
LA2	Clarias sp.	opercular	1	20-25	1
LA2	Clarias sp.	pectoral spine	1	30-40	1
LA2	Clarias sp.	cranial roof fragment	4	40-50	1
LA2	Haplotilapiini indet.	cleithrum	1		
LA2	Haplotilapiini indet.	opercular	1		
LA2	Haplotilapiini indet.	basioccipital	1	20-25	1
LA2	Haplotilapiini indet.	basipterygium	1	15-20	1
LA2	Haplotilapiini indet.	hyomandibula	1	15-20	1
LA2	Haplotilapiini indet.	unidentified	1		
LA2	Haplotilapiini indet.	fin spine	1		
LA2	Haplotilapiini indet.	opercular	1	15-20	1
LA2	Haplotilapiini indet.	1st precaudal vertebra	1	15-20	1
LA2	Haplotilapiini indet.	3rd precaudal vertebra	1	20-25	1
LA2	Haplotilapiini indet.	postcleithrum	1	15-20	1
LA2	Haplotilapiini indet.	dorsal or anal pterygophore	1	15-20	1
LA2	Haplotilapiini indet.	dorsal or anal pterygophore	1	15-20	1

cultural phase	taxon	skeletal element	NISP	SL in cm	number
LA2	Haplotilapiini indet.	precaudal vertebra	3	15-20	3
LA2	Haplotilapiini indet.	skull roof fragment	4		
LA2	Haplotilapiini indet.	cleithrum	5	15-20	1
LA2	Clarias sp.	coracoid	1		
LA2	Clarias sp.	caudal vertebra	2	40-50	2
LA2	Clarias sp.	cranial roof fragment	3		
LA2	Clarias sp.	articular	1	40-50	1
LA2	Clarias sp.	cranial roof fragment	1		
LA2	Clarias sp.	precaudal vertebra	2	50-60	2
LA2	Clarias sp.	articular	1	40-50	1
LA2	Clarias sp.	caudal vertebra	1	30-40	1
LA2	Clarias sp.	caudal vertebra	1	60-70	1
LA2	Clarias sp.	ceratohyale	1	70-80	1
LA2	Clarias sp.	cleithrum	1		
LA2	Clarias sp.	cranial roof fragment	1	40-50	1
LA2	Clarias sp.	cranial roof fragment	1	50-60	1
LA2	Clarias sp.	pectoral spine	1	50-60	1
LA2	Clarias sp.	caudal vertebra	1	50-60	1
LA2	Clarias sp.	cranial roof fragment	1	30-40	1
LA2	Clarias sp.	precaudal vertebra	1	50-60	1
LA2	Clarias sp.	pectoral spine	1	30-40	1
LA2	Clarias sp.	caudal vertebra	4	30-40	4
LA2	Clarias sp.	cleithrum	1		
LA2	Clarias sp.	mesethmoid	1	20-30	1
LA2	Clarias sp.	cranial roof fragment	1	40-50	1
LA2	Clarias sp.	pectoral spine	1	40-50	1
LA2	Clarias sp.	cranial roof fragment	2		
LA2	Clarias sp.	cranial roof fragment	3	30-40	3
LA2	Clarias sp.	basioccipital	1	20-30	1
LA2	Clarias sp.	caudal vertebra	1	40-50	1
LA2	Clarias sp.	cleithrum	1	30-40	1
LA2	Clarias sp.	hyomandibula	1	30-40	1
LA2	Clarias sp.	precaudal vertebra	1	30-40	1
LA2	Clarias sp.	premaxilla	1		
LA2	Clarias sp.	cranial roof fragment	2	50-60	2
LA2	Clarias sp.	caudal vertebra	4	30-40	4
LA2	Clarias sp.	cranial roof fragment	5		
LA2	Clarias sp.	articular	1	40-50	1
LA2	Clarias sp.	caudal vertebra	1	50-60	1
LA2	Clarias sp.	epihyale	1		
LA2	Clarias sp.	cranial roof fragment	3		
LA2	Clarias sp.	cranial roof fragment	2	30-40	1
LA2	Clarias sp.	mesethmoid	1	40-50	1
LA2	Clarias sp.	cranial roof fragment	5		
LA2	Haplotilapiini indet.	fin spine	1		
LA2	Haplotilapiini indet.	opercular	1		
LA2	Haplotilapiini indet.	dorsal or anal pterygophore	1	15-20	1
LA2	Haplotilapiini indet.	basioccipital	1	15-20	1
LA2	Haplotilapiini indet.	basipterygium	1		
LA2	Haplotilapiini indet.	caudal vertebra	1	20-25	1
LA2	O. niloticus	hyomandibula	1	15-20	1
LA2	O. niloticus	hyomandibula	1	15-20	1
LA2	O. niloticus	mesethmoid	1	20-25	1
LA2	Haplotilapiini indet.	opercular	1	15-20	1
LA2	Haplotilapiini indet.	opercular	1		
LA2	Haplotilapiini indet.	2nd precaudal vertebra	1	20-25	1
LA2	Haplotilapiini indet.	3rd precaudal vertebra	1	15-20	1
LA2	Haplotilapiini indet.	3rd precaudal vertebra	1	20-25	1

cultural phase	taxon	skeletal element	NISP	SL in cm	number
LA2	Haplotilapiini indet.	precaudal vertebra	1	20-25	1
LA2	Haplotilapiini indet.	postcleithrum	1		
LA2	Haplotilapiini indet.	dorsal or anal pterygophore	1	20-25	1
LA2	Haplotilapiini indet.	cleithrum	2		
LA2	Haplotilapiini indet.	caudal vertebra	5	15-20	1
LA2	Haplotilapiini indet.	skull roof fragment	5		
LA2	Haplotilapiini indet.	articular	1	15-20	1
LA2	Haplotilapiini indet.	articular	1	20-25	1
LA2	O. niloticus	hyomandibula	1	15-20	1
LA2	O. niloticus	hyomandibula	1	15-20	1
LA2	O. niloticus	hyomandibula	1	15-20	1
LA2	O. niloticus	mesethmoid	1	20-25	1
LA2	Haplotilapiini indet.	opercular	1	20-25	1
LA2	Haplotilapiini indet.	opercular	1	25-30	1
LA2	Haplotilapiini indet.	opercular	1		
LA2	Haplotilapiini indet.	palatinum	1	20-25	1
LA2	Haplotilapiini indet.	dorsal or anal pterygophore	1	15-20	1
LA2	Haplotilapiini indet.	dorsal or anal pterygophore	1	15-20	1
LA2	Haplotilapiini indet.	dorsal or anal pterygophore	1	15-20	1
LA2	Haplotilapiini indet.	scapula	1	15-20	1
LA2	Haplotilapiini indet.	precaudal vertebra	22	15-20	22
LA2	Haplotilapiini indet.	basipterygium	9	15-20	9
LA2	Haplotilapiini indet.	basioccipital	1	20-25	1
LA2	Haplotilapiini indet.	basioccipital	1	20-25	1
LA2	O. niloticus	hyomandibula	1	25-30	1
LA2	O. niloticus	hyomandibula	1	20-25	1
LA2	O. niloticus	hyomandibula	1	15-20	1
LA2	O. niloticus	hyomandibula	1	15-20	1
LA2	O. niloticus	hyomandibula	1	10-15	1
LA2	Haplotilapiini indet.	hyomandibula	1		
LA2	O. niloticus	mesethmoid	1	20-25	1
LA2	Haplotilapiini indet.	skull roof fragment	1	20-25	1
LA2	Haplotilapiini indet.	1st precaudal vertebra	1	20-25	1
LA2	Haplotilapiini indet.	2nd precaudal vertebra	1	15-20	1
LA2	Haplotilapiini indet.	2nd precaudal vertebra	1	20-25	1
LA2	Haplotilapiini indet.	2nd precaudal vertebra	1	20-25	1
LA2	Haplotilapiini indet.	3rd precaudal vertebra	1	15-20	1
LA2	O. niloticus	premaxilla	1	20-25	1
LA2	Haplotilapiini indet.	postcleithrum	1	20-25	1
LA2	Haplotilapiini indet.	posttemporal	1	20-25	1
LA2	Haplotilapiini indet.	dorsal or anal pterygophore	1	10-15	1
LA2	Haplotilapiini indet.	dorsal or anal pterygophore	1	15-20	1
LA2	Haplotilapiini indet.	dorsal or anal pterygophore	1	15-20	1
LA2	Haplotilapiini indet.	quadrate	1	15-20	1
LA2	Haplotilapiini indet.	supracleithrum	1	20-25	1
LA2	Haplotilapiini indet.	urohyale	1	15-20	1
LA2	Haplotilapiini indet.	lepidotrich	13		
LA2	Haplotilapiini indet.	caudal vertebra	2	20-25	2
LA2	Haplotilapiini indet.	precaudal vertebra	2	20-25	2
LA2	Haplotilapiini indet.	preopercular	2		
LA2	Haplotilapiini indet.	caudal vertebra	20	15-20	20
LA2	Haplotilapiini indet.	skull roof fragment	3	15-20	3
LA2	Haplotilapiini indet.	postcleithrum	3	15-20	3
LA2	Haplotilapiini indet.	dorsal or anal pterygophore	3		
LA2	Haplotilapiini indet.	supracleithrum	3	15-20	3
LA2	Haplotilapiini indet.	skull roof fragment	8		
LA2	Haplotilapiini indet.	cleithrum	9		
LA2	Haplotilapiini indet.	fin spine	1		

cultural phase	taxon	skeletal element	NISP	SL in cm	number
LA2	Haplotilapiini indet.	opercular	1		
LA2	Haplotilapiini indet.	1st precaudal vertebra	1	20-25	1
LA2	Haplotilapiini indet.	1st precaudal vertebra	1	15-20	1
LA2	Haplotilapiini indet.	2nd precaudal vertebra	1	15-20	1
LA2	Haplotilapiini indet.	supracleithrum	1	15-20	1
LA2	Haplotilapiini indet.	cleithrum	3		
LA2	Haplotilapiini indet.	skull roof fragment	3	15-20	1
LA2	Haplotilapiini indet.	precaudal vertebra	6	15-20	6
LA2	Haplotilapiini indet.	caudal vertebra	7	15-20	7
LA2	Haplotilapiini indet.	basipterygium	1	15-20	1
LA2	Haplotilapiini indet.	basipterygium	1	20-25	1
LA2	Haplotilapiini indet.	caudal vertebra	1	20-25	1
LA2	O. niloticus	hyomandibula	1	20-25	1
LA2	Haplotilapiini indet.	mesethmoid	1	20-25	1
LA2	Haplotilapiini indet.	opercular	1		
LA2	O. niloticus	1st precaudal vertebra	1	25-30	1
LA2	Haplotilapiini indet.	postcleithrum	1	15-20	1
LA2	Haplotilapiini indet.	postcleithrum	1	20-25	1
LA2	Haplotilapiini indet.	dorsal or anal pterygophore	1	10-15	1
LA2	Haplotilapiini indet.	urohyale	1		
LA2	Haplotilapiini indet.	cleithrum	2		
LA2	Haplotilapiini indet.	lepidotrich	2		
LA2	Haplotilapiini indet.	precaudal vertebra	2	20-25	1
LA2	Haplotilapiini indet.	skull roof fragment	3		
LA2	Haplotilapiini indet.	precaudal vertebra	4	15-20	4
LA2	Haplotilapiini indet.	caudal vertebra	6	15-20	6
LA2	O. niloticus	mesethmoid	1	20-25	1
LA2	Haplotilapiini indet.	cleithrum	2	15-20	2
LA2	Haplotilapiini indet.	precaudal vertebra	2	15-20	2
LA2	Clarias sp.	cranial roof fragment	2	40-50	1
LA2	Clarias sp.	cleithrum	1	30-40	1
LA2	Clarias sp.	cranial roof fragment	1		
LA2	Clarias sp.	cleithrum	1	50-60	1
LA2	Clarias sp.	caudal vertebra	1	40-50	1
LA2	Clarias sp.	pectoral spine	1	50-60	1
LA2	Clarias sp.	cranial roof fragment	1		
LA2	Clarias sp.	pectoral spine	1	40-50	1
LA2	Clarias sp.	quadrate	1	20-30	1
LA2	Clarias sp.	caudal vertebra	1	60-70	1
LA2	Clarias sp.	precaudal vertebra	1	50-60	1
LA2	Clarias sp.	vomerine toothplate	1	30-40	1
LA2	Clarias sp.	caudal vertebra	2	50-60	2
LA2	Clarias sp.	caudal vertebra	2	50-60	2
LA2	Clarias sp.	caudal vertebra	1	50-60	1
LA2	Clarias sp.	cleithrum	1		
LA2	Clarias sp.	cranial roof fragment	4		
LA2	Clarias sp.	cranial roof fragment	3		
LA2	Clarias sp.	cleithrum	1	30-40	1
LA2	Clarias sp.	coracoid	1	40-50	1
LA2	Clarias sp.	caudal vertebra	2	50-60	2
LA2	Clarias sp.	caudal vertebra	4	40-50	4
LA2	Clarias sp.	cranial roof fragment	1	50-60	1
LA2	Clarias sp.	articular	1		
LA2	Clarias sp.	opercular	1	50-60	1
LA2	Clarias sp.	pectoral spine	1	70-80	1
LA2	Clarias sp.	cranial roof fragment	5	40-50	1
LA2	Haplotilapiini indet.	cleithrum	1		
LA2	Haplotilapiini indet.	skull roof fragment	1		

cultural phase	taxon	skeletal element	NISP	SL in cm	number
LA2	Haplotilapiini indet.	3rd precaudal vertebra	1	20-25	1
LA2	Haplotilapiini indet.	urohyale	1	15-20	1
LA2	Haplotilapiini indet.	precaudal vertebra	2	15-20	2
LA2	Haplotilapiini indet.	dorsal or anal pterygophore	2		
LA2	Haplotilapiini indet.	caudal vertebra	3	15-20	3
LA2	Haplotilapiini indet.	cleithrum	1		
LA2	Haplotilapiini indet.	fin spine	1		
LA2	Haplotilapiini indet.	1st precaudal vertebra	1	20-25	1
LA2	Haplotilapiini indet.	caudal vertebra	2	15-20	2
LA2	Haplotilapiini indet.	lepidotrich	2		
LA2	Haplotilapiini indet.	cleithrum	1		
LA2	Haplotilapiini indet.	dentary	1	20-25	1
LA2	Haplotilapiini indet.	dorsal or anal pterygophore	1		
LA2	Haplotilapiini indet.	skull roof fragment	1		
LA2	Haplotilapiini indet.	basipterygium	1	20-25	1
LA2	Haplotilapiini indet.	cleithrum	1		
LA2	Haplotilapiini indet.	cleithrum	1		
LA2	Haplotilapiini indet.	fin spine	1		
LA2	Haplotilapiini indet.	mesethmoid	1	15-20	1
LA2	Haplotilapiini indet.	opercular	1	20-25	1
LA2	Haplotilapiini indet.	postcleithrum	1	20-25	1
LA2	Haplotilapiini indet.	dorsal or anal pterygophore	1		
LA2	Haplotilapiini indet.	caudal vertebra	2	15-20	2
LA2	Haplotilapiini indet.	skull roof fragment	2		
LA2	Haplotilapiini indet.	precaudal vertebra	3	15-20	3
LA2	Haplotilapiini indet.	caudal vertebra	1	15-20	1
LA2	Haplotilapiini indet.	cleithrum	1		
LA2	Haplotilapiini indet.	skull roof fragment	1		
LA2	Haplotilapiini indet.	palatinum	1	15-20	1
LA2	Haplotilapiini indet.	palatinum	1	20-25	1
LA2	Haplotilapiini indet.	caudal vertebra	1	15-20	1
LA2	Haplotilapiini indet.	cleithrum	1		
LA2	Haplotilapiini indet.	skull roof fragment	1		
LA2	Clarias sp.	articular	1	50-60	1
LA2	Clarias sp.	caudal vertebra	1	70-80	1
LA2	Clarias sp.	ceratohyale	1		
LA2	Clarias sp.	cleithrum	1		
LA2	Clarias sp.	coracoid	1		
LA2	Clarias sp.	pectoral spine	1	60-70	1
LA2	Clarias sp.	pectoral spine	1	60-70	1
LA2	Clarias sp.	pectoral spine	1	60-70	1
LA2	Clarias sp.	vomerine toothplate	1		
LA2	Clarias sp.	caudal vertebra	4	60-70	4
LA2	Clarias sp.	cranial roof fragment	6		
LA2	Haplotilapiini indet.	caudal vertebra	1	25-30	1
LA2	Haplotilapiini indet.	precaudal vertebra	1		
LA2	Haplotilapiini indet.	dorsal or anal pterygophore	1		
LA2	Haplotilapiini indet.	skull roof fragment	2		
LA2	Clarias sp.	dentary	1	30-40	1
LA2	Clarias sp.	hyomandibula	1	60-70	1
LA2	Clarias sp.	branchial element	1		
LA2	Clarias sp.	pectoral spine	1	50-60	1
LA2	Clarias sp.	cleithrum	2		
LA2	Clarias sp.	cranial roof fragment	2		
LA2	Haplotilapiini indet.	fin spine	1		
LA2	Haplotilapiini indet.	basipterygium	1	15-20	1
LA2	O. niloticus	mesethmoid	1	20-25	1
LA2	Haplotilapiini indet.	opercular	1		

cultural phase	taxon	skeletal element	NISP	SL in cm	number
LA2	Haplotilapiini indet.	precaudal vertebra	1	20-25	1
LA2	Haplotilapiini indet.	quadrate	1		
LA2	Haplotilapiini indet.	cleithrum	2		
LA2	Clarias sp.	caudal vertebra	1	40-50	1
LA2	Clarias sp.	ceratohyale	1	40-50	1
LA2	Clarias sp.	precaudal vertebra	1	40-50	1
LA2	Clarias sp.	cranial roof fragment	2		
LA2	Haplotilapiini indet.	unidentified	1		
LA2	Haplotilapiini indet.	precaudal vertebra	1	15-20	1
LA2	Haplotilapiini indet.	precaudal vertebra	1	20-25	1
LA2	Clarias sp.	cranial roof fragment	1	40-50	1
LA2	Clarias sp.	caudal vertebra	1	50-60	1
LA2	Clarias sp.	ceratohyale	1	50-60	1
LA2	Clarias sp.	cleithrum	1		
LA2	Clarias sp.	quadrate	1	60-70	1
LA2	Clarias sp.	cranial roof fragment	2		
LA2	Clarias sp.	cleithrum	1	30-40	1
LA2	Clarias sp.	cleithrum	1		
LA2	Clarias sp.	dentary	1		
LA2	Clarias sp.	pectoral spine	1	50-60	1
LA2	Clarias sp.	coracoid	2		
LA2	Clarias sp.	cranial roof fragment	3		
LA2	Clarias sp.	cleithrum	1	40-50	1
LA2	Clarias sp.	cleithrum	1	60-70	1
LA2	Clarias sp.	cleithrum	1	70-80	1
LA2	Clarias sp.	cranial roof fragment	4	30-40	1
LA2	Clarias sp.	caudal vertebra	1	60-70	1
LA2	Clarias sp.	ceratohyale	1	70-80	1
LA2	Clarias sp.	cleithrum	1	30-40	1
LA2	Clarias sp.	cleithrum	1		
LA2	Clarias sp.	costa	1		
LA2	Clarias sp.	hyomandibula	1	50-60	1
LA2	Clarias sp.	vomerine toothplate	1	50-60	1
LA2	Clarias sp.	cranial roof fragment	4	50-60	4
LA2	Clarias sp.	cranial roof fragment	6	60-70	6
LA2	Clarias sp.	cranial roof fragment	6		
LA2	Clarias sp.	basioccipital	1	60-70	1
LA2	Clarias sp.	caudal vertebra	1	30-40	1
LA2	Clarias sp.	cleithrum	1		
LA2	Clarias sp.	dentary	1	30-40	1
LA2	Clarias sp.	epihyale	1	60-70	1
LA2	Clarias sp.	cranial roof fragment	1	30-40	1
LA2	Clarias sp.	opercular	1	40-50	1
LA2	Clarias sp.	opercular	1	50-60	1
LA2	Clarias sp.	precaudal vertebra	1	30-40	1
LA2	Clarias sp.	quadrate	1	50-60	1
LA2	Clarias sp.	cranial roof fragment	15		
LA2	Clarias sp.	coracoid	2	50-60	1
LA2	Clarias sp.	cranial roof fragment	2	60-70	2
LA2	Clarias sp.	precaudal vertebra	2	60-70	2
LA2	Clarias sp.	caudal vertebra	3	50-60	3
LA2	Clarias sp.	ceratohyale	1	70-80	1
LA2	Clarias sp.	ceratohyale	1	70-80	1
LA2	Clarias sp.	cleithrum	1	50-60	1
LA2	Clarias sp.	cranial roof fragment	3		
LA2	Clarias sp.	articular	1	30-40	1
LA2	Clarias sp.	basioccipital	1	30-40	1
LA2	Clarias sp.	cleithrum	1	80-90	1

cultural phase	taxon	skeletal element	NISP	SL in cm	number
LA2	Clarias sp.	unidentified	1		
LA2	Clarias sp.	precaudal vertebra	1	70-80	1
LA2	Clarias sp.	precaudal vertebra	1	50-60	1
LA2	Clarias sp.	caudal vertebra	2	30-40	2
LA2	Clarias sp.	cranial roof fragment	3	30-40	1
LA2	Clarias sp.	ceratohyale	1	40-50	1
LA2	Clarias sp.	ceratohyale	1	40-50	1
LA2	Clarias sp.	hyomandibula	1	40-50	1
LA2	Clarias sp.	cranial roof fragment	1	30-40	1
LA2	Clarias sp.	cranial roof fragment	1	40-50	1
LA2	Clarias sp.	cranial roof fragment	1	50-60	1
LA2	Clarias sp.	opercular	1	60-70	1
LA2	Clarias sp.	pectoral spine	1	30-40	1
LA2	Clarias sp.	quadrate	1	40-50	1
LA2	Clarias sp.	caudal vertebra	11	50-60	1
LA2	Clarias sp.	cranial roof fragment	5		
LA2	Clarias sp.	basioccipital	1	80-90	1
LA2	Clarias sp.	basioccipital	1	60-70	1
LA2	Clarias sp.	basioccipital	1	40-50	1
LA2	Clarias sp.	ceratohyale	1	60-70	1
LA2	Clarias sp.	cleithrum	1	70-80	1
LA2	Clarias sp.	dentary	1	40-50	1
LA2	Clarias sp.	dentary	1	50-60	1
LA2	Clarias sp.	dentary	1		
LA2	Clarias sp.	hyomandibula	1	50-60	1
LA2	Clarias sp.	cranial roof fragment	1	50-60	
LA2	Clarias sp.	pectoral spine	1	40-50	1
LA2	Clarias sp.	pectoral spine	1	50-60	1
LA2	Clarias sp.	quadrate	1	60-70	1
LA2	Clarias sp.	quadrate	1	50-60	1
LA2	Clarias sp.	quadrate	1	50-60	1
LA2	Clarias sp.	vomerine toothplate	1	70-80	1
LA2	Clarias sp.	cranial roof fragment	13		
LA2	Clarias sp.	caudal vertebra	2	50-60	2
LA2	Clarias sp.	cranial roof fragment	2	20-30	2
LA2	Clarias sp.	caudal vertebra	3	60-70	3
LA2	Clarias sp.	cranial roof fragment	4	40-50	4
LA2	Clarias sp.	precaudal vertebra	5	60-70	5
LA2	Clarias sp.	cranial roof fragment	7	60-70	7
LA2	Clarias sp.	caudal vertebra	1	30-40	1
LA2	Clarias sp.	caudal vertebra	1	50-60	1
LA2	Clarias sp.	caudal vertebra	1	60-70	1
LA2	Clarias sp.	coracoid	1	60-70	1
LA2	Clarias sp.	cranial roof fragment	1	50-60	1
LA2	Clarias sp.	opercular	1	40-50	1
LA2	Clarias sp.	pectoral spine	1	60-70	1
LA2	Clarias sp.	caudal vertebra	2	40-50	2
LA2	Clarias sp.	precaudal vertebra	3	60-70	3
LA2	Clarias sp.	cranial roof fragment	8	60-70	8
LA2	Clarias sp.	cranial roof fragment	8		
LA2	Clarias sp.	basioccipital	1	10-15	1
LA2	Clarias sp.	caudal vertebra	1	40-50	1
LA2	Clarias sp.	precaudal vertebra	1	40-50	1
LA2	Clarias sp.	precaudal vertebra	1	60-70	1
LA2	Clarias sp.	pectoral spine	1	50-60	1
LA2	Clarias sp.	caudal vertebra	2	60-70	2
LA2	Clarias sp.	cranial roof fragment	2		
LA2	Clarias sp.	articular	1	60-70	1

cultural phase	taxon	skeletal element	NISP	SL in cm	number
LA2	Clarias sp.	basioccipital	1	30-40	1
LA2	Clarias sp.	cleithrum	1		
LA2	Clarias sp.	coracoid	1		
LA2	Clarias sp.	cranial roof fragment	1	20-30	1
LA2	Clarias sp.	cranial roof fragment	1	60-70	1
LA2	Clarias sp.	precaudal vertebra	1	40-50	1
LA2	Clarias sp.	precaudal vertebra	1	70-80	1
LA2	Clarias sp.	vomerine toothplate	1	50-60	1
LA2	Clarias sp.	cranial roof fragment	15		
LA2	Clarias sp.	caudal vertebra	2	50-60	2
LA2	Clarias sp.	caudal vertebra	2	60-70	2
LA2	Clarias sp.	precaudal vertebra	2	40-50	2
LA2	Clarias sp.	cranial roof fragment	4	40-50	4
LA2	Clarias sp.	caudal vertebra	1	40-50	1
LA2	Clarias sp.	cleithrum	2	30-40	1
LA2	Clarias sp.	cranial roof fragment	3	60-70	2
LA2	Clarias sp.	dentary	1	60-70	1
LA2	Clarias sp.	cranial roof fragment	3	40-50	1
LA2	Clarias sp.	caudal vertebra	1	50-60	1
LA2	Clarias sp.	hyomandibula	1	60-70	1
LA2	Clarias sp.	hyomandibula	1	30-40	1
LA2	Clarias sp.	cranial roof fragment	1	50-60	1
LA2	Clarias sp.	opercular	1	60-70	1
LA2	Clarias sp.	pectoral spine	1	40-50	1
LA2	Clarias sp.	cranial roof fragment	14		
LA2	Clarias sp.	caudal vertebra	2	60-70	2
LA2	Clarias sp.	ceratohyale	2	60-70	2
LA2	Clarias sp.	cranial roof fragment	2	60-70	2
LA2	Clarias sp.	precaudal vertebra	2	50-60	2
LA2	Clarias sp.	cranial roof fragment	3	40-50	3
LA2	Clarias sp.	precaudal vertebra	3	60-70	3
LA2	Clarias sp.	cleithrum	4		
LA2	Clarias sp.	coracoid	4		
LA2	Clarias sp.	basioccipital	1	30-40	1
LA2	Clarias sp.	ceratohyale	1	50-60	1
LA2	Clarias sp.	ceratohyale	1	50-60	1
LA2	Clarias sp.	precaudal vertebra	1	60-70	1
LA2	Clarias sp.	caudal vertebra	2	40-50	2
LA2	Clarias sp.	caudal vertebra	5	60-70	5
LA2	Clarias sp.	cranial roof fragment	7	30-40	1
LA2	Clarias sp.	caudal vertebra	1	60-70	1
LA2	Clarias sp.	ceratohyale	1	70-80	1
LA2	Clarias sp.	dentary	1	50-60	1
LA2	Clarias sp.	hyomandibula	1	30-40	1
LA2	Clarias sp.	cranial roof fragment	2	60-70	1
LA2	Clarias sp.	cleithrum	1		
LA2	Clarias sp.	cleithrum	1		
LA2	Clarias sp.	articular	1	60-70	1
LA2	Clarias sp.	caudal vertebra	1	70-80	1
LA2	Clarias sp.	cranial roof fragment	1	60-70	1
LA2	Clarias sp.	cleithrum	1	30-40	1
LA2	Clarias sp.	hyomandibula	1	30-40	1
LA2	Clarias sp.	cranial roof fragment	1	40-50	1
LA2	Clarias sp.	precaudal vertebra	1	50-60	1
LA2	Clarias sp.	cleithrum	2		
LA2	Clarias sp.	cranial roof fragment	2	50-60	2
LA2	Clarias sp.	caudal vertebra	3	60-70	3
LA2	Clarias sp.	cranial roof fragment	3		

cultural phase	taxon	skeletal element	NISP	SL in cm	number
LA2	Clarias sp.	cranial roof fragment	4	60-70	4
LA2	Clarias sp.	basioccipital	1	50-60	1
LA2	Clarias sp.	caudal vertebra	1	40-50	1
LA2	Clarias sp.	ceratohyale	1	70-80	1
LA2	Clarias sp.	cleithrum	1	40-50	1
LA2	Clarias sp.	dentary	1	30-40	1
LA2	Clarias sp.	epihyale	1	50-60	1
LA2	Clarias sp.	hyomandibula	1	50-60	1
LA2	Clarias sp.	hypohyale	1		
LA2	Clarias sp.	cranial roof fragment	1	30-40	1
LA2	Clarias sp.	palatinum	1	60-70	1
LA2	Clarias sp.	precaudal vertebra	1	40-50	1
LA2	Clarias sp.	cleithrum	2		
LA2	Clarias sp.	coracoid	2		
LA2	Clarias sp.	cranial roof fragment	22		
LA2	Clarias sp.	caudal vertebra	3	50-60	3
LA2	Clarias sp.	cranial roof fragment	3	50-60	3
LA2	Clarias sp.	caudal vertebra	6	60-70	6
LA2	Clarias sp.	articular	1	40-50	1
LA2	Clarias sp.	articular	1	50-60	1
LA2	Clarias sp.	caudal vertebra	1	60-70	1
LA2	Clarias sp.	ceratohyale	1	30-40	1
LA2	Clarias sp.	cleithrum	1	50-60	1
LA2	Clarias sp.	cleithrum	1	40-50	1
LA2	Clarias sp.	dentary	1	50-60	1
LA2	Clarias sp.	cranial roof fragment	2	50-60	2
LA2	Clarias sp.	cranial roof fragment	2	60-70	2
LA2	Clarias sp.	precaudal vertebra	2	40-50	2
LA2	Clarias sp.	caudal vertebra	3	40-50	3
LA2	Clarias sp.	cranial roof fragment	3	30-40	3
LA2	Clarias sp.	cranial roof fragment	3	40-50	3
LA2	Clarias sp.	cranial roof fragment	5		
LA2	Clarias sp.	basioccipital	1	50-60	1
LA2	Clarias sp.	basioccipital	1	60-70	1
LA2	Clarias sp.	pectoral spine	1	70-80	1
LA2	Clarias sp.	cranial roof fragment	5	60-70	1
LA2	Clarias sp.	articular	1	60-70	1
LA2	Clarias sp.	precaudal vertebra	1	60-70	1
LA2	Clarias sp.	vomerine toothplate	1	60-70	1
LA2	Clarias sp.	basioccipital	1	40-50	1
LA2	Clarias sp.	ceratohyale	1	30-40	1
LA2	Clarias sp.	ceratohyale	1	40-50	1
LA2	Clarias sp.	coracoid	1		
LA2	Clarias sp.	dentary	1	50-60	1
LA2	Clarias sp.	mesethmoid	1	60-70	1
LA2	Clarias sp.	cranial roof fragment	2		
LA2	Clarias sp.	precaudal vertebra	2	50-60	2
LA2	Clarias sp.	caudal vertebra	3	50-60	3
LA2	Haplotilapiini indet.	3rd precaudal vertebra	1	25-30	1
LA2	Haplotilapiini indet.	dorsal or anal pterygophore	1	15-20	1
LA2	Haplotilapiini indet.	dorsal or anal pterygophore	1		
LA2	Haplotilapiini indet.	lepidotrich	2		
LA2	Haplotilapiini indet.	cleithrum	3	20-25	2
LA2	Haplotilapiini indet.	skull roof fragment	3		
LA2	Haplotilapiini indet.	caudal vertebra	1	20-25	1
LA2	Haplotilapiini indet.	fin spine	1		
LA2	Haplotilapiini indet.	dorsal or anal pterygophore	1	25-30	1
LA2	Haplotilapiini indet.	caudal vertebra	2	15-20	2

cultural phase	taxon	skeletal element	NISP	SL in cm	number
LA2	Haplotilapiini indet.	skull roof fragment	2		
LA2	Haplotilapiini indet.	2nd precaudal vertebra	1	20-25	1
LA2	Haplotilapiini indet.	cleithrum	2	20-25	1
LA2	Haplotilapiini indet.	preopercular	2		
LA2	Haplotilapiini indet.	skull roof fragment	1		
LA2	Haplotilapiini indet.	opercular	1		
LA2	Haplotilapiini indet.	cleithrum	3		
LA2	Haplotilapiini indet.	caudal vertebra	1	20-25	1
LA2	Haplotilapiini indet.	caudal vertebra	1	25-30	1
LA2	Haplotilapiini indet.	skull roof fragment	1	25-30	1
LA2	Haplotilapiini indet.	2nd precaudal vertebra	1	20-25	1
LA2	Haplotilapiini indet.	precaudal vertebra	1	25-30	1
LA2	Haplotilapiini indet.	dorsal or anal pterygophore	1	15-20	1
LA2	Haplotilapiini indet.	dorsal or anal pterygophore	1		
LA2	Haplotilapiini indet.	precaudal vertebra	3	20-25	3
LA2	Haplotilapiini indet.	caudal vertebra	1	15-20	1
LA2	Haplotilapiini indet.	caudal vertebra	1	15-20	1
LA2	O. niloticus	hyomandibula	1	25-30	1
LA2	Haplotilapiini indet.	opercular	1	25-30	1
LA2	Haplotilapiini indet.	palatinum	1	25-30	1
LA2	Haplotilapiini indet.	preopercular	1		
LA2	Haplotilapiini indet.	postcleithrum	1	20-25	1
LA2	Haplotilapiini indet.	dorsal or anal pterygophore	1	15-20	1
LA2	Haplotilapiini indet.	dorsal or anal pterygophore	1	20-25	1
LA2	Haplotilapiini indet.	dorsal or anal pterygophore	1	15-20	1
LA2	Haplotilapiini indet.	supracleithrum	1		
LA2	Haplotilapiini indet.	caudal vertebra	2	20-25	2
LA2	Haplotilapiini indet.	cleithrum	3		
LA2	Haplotilapiini indet.	precaudal vertebra	3	20-25	3
LA2	Haplotilapiini indet.	precaudal vertebra	5	15-20	5
LA2	Haplotilapiini indet.	skull roof fragment	1		
LA2	Haplotilapiini indet.	basioccipital	1	20-25	1
LA2	Haplotilapiini indet.	basioccipital	1	25-30	1
LA2	Haplotilapiini indet.	basipterygium	1	15-20	1
LA2	Haplotilapiini indet.	hyomandibula	1	25-30	1
LA2	Haplotilapiini indet.	interopercular	1	20-25	1
LA2	Haplotilapiini indet.	opercular	1	15-20	1
LA2	Haplotilapiini indet.	opercular	1	15-20	1
LA2	Haplotilapiini indet.	opercular	1	20-25	1
LA2	Haplotilapiini indet.	opercular	1	25-30	1
LA2	Haplotilapiini indet.	3rd precaudal vertebra	1	25-30	1
LA2	Haplotilapiini indet.	preopercular	1		
LA2	Haplotilapiini indet.	dorsal or anal pterygophore	1	15-20	1
LA2	Haplotilapiini indet.	dorsal or anal pterygophore	1	15-20	1
LA2	Haplotilapiini indet.	dorsal or anal pterygophore	1	20-25	1
LA2	Haplotilapiini indet.	dorsal or anal pterygophore	1	20-25	1
LA2	Haplotilapiini indet.	urohyale	1		
LA2	Haplotilapiini indet.	caudal vertebra	2	15-20	2
LA2	Haplotilapiini indet.	precaudal vertebra	2	15-20	2
LA2	Haplotilapiini indet.	precaudal vertebra	2	20-25	2
LA2	Haplotilapiini indet.	caudal vertebra	3	20-25	3
LA2	Haplotilapiini indet.	lepidotrich	3		
LA2	Haplotilapiini indet.	skull roof fragment	5		
LA2	Haplotilapiini indet.	cleithrum	7		
LA2	Haplotilapiini indet.	cleithrum	1	20-25	1
LA2	Haplotilapiini indet.	fin spine	1		
LA2	Haplotilapiini indet.	opercular	1	15-20	1
LA2	Haplotilapiini indet.	3rd precaudal vertebra	1	20-25	1

cultural phase	taxon	skeletal element	NISP	SL in cm	number
LA2	Haplotilapiini indet.	precaudal vertebra	1		
LA2	Haplotilapiini indet.	basipterygium	1	20-25	1
LA2	Haplotilapiini indet.	skull roof fragment	1		
LA2	Haplotilapiini indet.	articular	1	20-25	1
LA2	Haplotilapiini indet.	cleithrum	1		
LA2	Haplotilapiini indet.	3rd precaudal vertebra	1	25-30	1
LA2	Haplotilapiini indet.	precaudal vertebra	1	20-25	1
LA2	Haplotilapiini indet.	dorsal or anal pterygophore	1	20-25	1
LA2	Haplotilapiini indet.	skull roof fragment	2	20-25	1
LA2	Haplotilapiini indet.	precaudal vertebra	2	15-20	2
LA2	O. niloticus	dentary	1	25-30	1
LA2	O. niloticus	hyomandibula	1	25-30	1
LA2	Haplotilapiini indet.	precaudal vertebra	1	25-30	1
LA2	Haplotilapiini indet.	premaxilla	1	25-30	1
LA2	Haplotilapiini indet.	preopercular	1		
LA2	Haplotilapiini indet.	dorsal or anal pterygophore	1	15-20	1
LA2	Haplotilapiini indet.	dorsal or anal pterygophore	1	15-20	1
LA2	Haplotilapiini indet.	dorsal or anal pterygophore	1	15-20	1
LA2	O. niloticus	urohyale	1	25-30	1
LA2	Haplotilapiini indet.	cleithrum	2		
LA2	Haplotilapiini indet.	skull roof fragment	2		
LA2	Haplotilapiini indet.	precaudal vertebra	2	20-25	2
LA2	Haplotilapiini indet.	lepidotrich	3		
LA2	Haplotilapiini indet.	basipterygium	1	20-25	1
LA2	Haplotilapiini indet.	cleithrum	1		
LA2	Haplotilapiini indet.	fin spine	1		
LA2	Haplotilapiini indet.	skull roof fragment	1		
LA2	Haplotilapiini indet.	dentary	1	25-30	1
LA2	Haplotilapiini indet.	skull roof fragment	1		
LA2	Haplotilapiini indet.	basipterygium	1	15-20	1
LA2	Haplotilapiini indet.	caudal vertebra	1	15-20	1
LA2	Haplotilapiini indet.	cleithrum	1		
LA2	Haplotilapiini indet.	skull roof fragment	1		
LA2	Haplotilapiini indet.	precaudal vertebra	1	15-20	1
LA2	Clarias sp.	caudal vertebra	1	60-70	1
LA2	Clarias sp.	ceratohyale	1	40-50	1
LA2	Clarias sp.	cleithrum	1	30-40	1
LA2	Clarias sp.	cranial roof fragment	4		
LA2	Clarias sp.	cranial roof fragment	2		
LA2	Haplotilapiini indet.	basipterygium	1	15-20	1
LA2	Haplotilapiini indet.	cleithrum	2	15-20	2
LA2	Clarias sp.	caudal vertebra	1	15-20	1
LA2	Clarias sp.	caudal vertebra	1	50-60	1
LA2	Clarias sp.	ceratohyale	1	50-60	1
LA2	Clarias sp.	cleithrum	1	30-40	1
LA2	Clarias sp.	cleithrum	1		
LA2	Clarias sp.	coracoid	1	60-70	1
LA2	Clarias sp.	dentary	1	50-60	1
LA2	Clarias sp.	palatinum	1	30-40	1
LA2	Clarias sp.	precaudal vertebra	1	15-20	1
LA2	Clarias sp.	precaudal vertebra	1	40-50	1
LA2	Clarias sp.	pectoral spine	1	40-50	1
LA2	Clarias sp.	pectoral spine	1		
LA2	Clarias sp.	cranial roof fragment	3	30-40	1
LA2	Clarias sp.	basioccipital	1	40-50	1
LA2	Clarias sp.	basioccipital	1	50-60	1
LA2	Clarias sp.	basioccipital	1	40-50	1
LA2	Clarias sp.	ceratohyale	1	70-80	1

cultural phase	taxon	skeletal element	NISP	SL in cm	number
LA2	Clarias sp.	cleithrum	1	40-50	1
LA2	Clarias sp.	cranial roof fragment	4		
LA2	Clarias sp.	caudal vertebra	1	60-70	1
LA2	Clarias sp.	cranial roof fragment	1		
LA2	Clarias sp.	caudal vertebra	5	30-40	5
LA2	Clarias sp.	caudal vertebra	1	60-70	1
LA2	Clarias sp.	epihyale	1	60-70	1
LA2	Clarias sp.	cranial roof fragment	1		
LA2	Clarias sp.	basipterygium	1		
LA2	Clarias sp.	ceratohyale	1	40-50	1
LA2	Clarias sp.	dentary	1	30-40	1
LA2	Clarias sp.	epihyale	1	50-60	1
LA2	Clarias sp.	epihyale	1	60-70	1
LA2	Clarias sp.	cranial roof fragment	1	40-50	1
LA2	Clarias sp.	cranial roof fragment	1	60-70	1
LA2	Clarias sp.	precaudal vertebra	1	50-60	1
LA2	Clarias sp.	caudal vertebra	3	40-50	3
LA2	Clarias sp.	caudal vertebra	5	50-60	5
LA2	Clarias sp.	cranial roof fragment	9		
LA2	Clarias sp.	caudal vertebra	1	30-40	1
LA2	Clarias sp.	caudal vertebra	2	50-60	2
LA2	Clarias sp.	cranial roof fragment	9	50-60	1
LA2	Clarias sp.	caudal vertebra	1	30-40	1
LA2	Clarias sp.	cleithrum	1	30-40	1
LA2	Clarias sp.	dentary	1	50-60	1
LA2	Clarias sp.	mesethmoid	1	30-40	1
LA2	Clarias sp.	cranial roof fragment	1	30-40	1
LA2	Clarias sp.	premaxilla	1	30-40	1
LA2	Clarias sp.	premaxilla	1	50-60	1
LA2	Clarias sp.	caudal vertebra	2	50-60	2
LA2	Clarias sp.	cranial roof fragment	4		
LA2	Clarias sp.	precaudal vertebra	1	60-70	1
LA2	Clarias sp.	urohyale	1	40-50	1
LA2	Clarias sp.	cranial roof fragment	3		1
LA2	Clarias sp.	precaudal vertebra	5	30-40	5
LA2	Clarias sp.	caudal vertebra	9	30-40	9
LA2	Clarias sp.	articular	1	50-60	1
LA2	Clarias sp.	articular	1	60-70	1
LA2	Clarias sp.	ceratohyale	1		
LA2	Clarias sp.	cleithrum	1	60-70	1
LA2	Clarias sp.	dentary	1	50-60	1
LA2	Clarias sp.	pectoral spine	1		
LA2	Clarias sp.	caudal vertebra	2	50-60	1
LA2	Clarias sp.	coracoid	2		
LA2	Clarias sp.	cranial roof fragment	7		
LA2	Clarias sp.	cranial roof fragment	2		
LA2	Clarias sp.	cranial roof fragment	1	30-40	1
LA2	Clarias sp.	cranial roof fragment	1	40-50	1
LA2	Clarias sp.	palatinum	1	50-60	1
LA2	Clarias sp.	precaudal vertebra	1	30-40	1
LA2	Clarias sp.	caudal vertebra	2	30-40	2
LA2	Clarias sp.	caudal vertebra	3	40-50	3
LA2	Clarias sp.	cranial roof fragment	3		
LA2	Clarias sp.	ceratohyale	1		
LA2	Clarias sp.	cranial roof fragment	1		
LA2	Clarias sp.	vomerine toothplate	1	60-70	1
LA2	Clarias sp.	caudal vertebra	2	60-70	2
LA2	Clarias sp.	precaudal vertebra	1	60-70	1

cultural phase	taxon	skeletal element	NISP	SL in cm	number
LA2	O. niloticus	hyomandibula	1	20-25	1
LA2	O. niloticus	3rd precaudal vertebra	1	25-30	1
LA2	Haplotilapiini indet.	basipterygium	1		
LA2	O. niloticus	hyomandibula	1	15-20	1
LA2	Haplotilapiini indet.	caudal vertebra	2	15-20	2
LA2	Haplotilapiini indet.	precaudal vertebra	4	15-20	4
LA2	Haplotilapiini indet.	precaudal vertebra	1	15-20	1
LA2	Haplotilapiini indet.	articular	1	25-30	1
LA2	Haplotilapiini indet.	ceratohyale	1	25-30	1
LA2	Haplotilapiini indet.	precaudal vertebra	1	15-20	1
LA2	Haplotilapiini indet.	caudal vertebra	2	15-20	2
LA2	Haplotilapiini indet.	caudal vertebra	2	20-25	2
LA2	Haplotilapiini indet.	cleithrum	2		
LA2	Haplotilapiini indet.	precaudal vertebra	2	20-25	2
LA2	Haplotilapiini indet.	2nd precaudal vertebra	1	20-25	1
LA2	Haplotilapiini indet.	articular	1	15-20	1
LA2	Haplotilapiini indet.	articular	1	15-20	1
LA2	O. niloticus	dentary	1	20-25	1
LA2	O. niloticus	hyomandibula	1	15-20	1
LA2	O. niloticus	hyomandibula	1	10-15	1
LA2	O. niloticus	mesethmoid	1	20-25	1
LA2	Haplotilapiini indet.	2nd precaudal vertebra	1	15-20	1
LA2	Haplotilapiini indet.	precaudal vertebra	1	20-25	1
LA2	Haplotilapiini indet.	premaxilla	1	25-30	1
LA2	Haplotilapiini indet.	dorsal or anal pterygophore	1	15-20	1
LA2	Haplotilapiini indet.	dorsal or anal pterygophore	1	15-20	1
LA2	Haplotilapiini indet.	dorsal or anal pterygophore	1	15-20	1
LA2	Haplotilapiini indet.	supracleithrum	1	20-25	1
LA2	Haplotilapiini indet.	precaudal vertebra	16	15-20	16
LA2	Haplotilapiini indet.	dorsal or anal pterygophore	2		
LA2	Haplotilapiini indet.	basipterygium	3	15-20	3
LA2	Haplotilapiini indet.	cleithrum	3		
LA2	Haplotilapiini indet.	caudal vertebra	5	15-20	5
LA2	Haplotilapiini indet.	skull roof fragment	6		
LA2	Haplotilapiini indet.	basioccipital	1	20-25	1
LA2	Haplotilapiini indet.	basipterygium	1	20-25	1
LA2	Haplotilapiini indet.	dentary	1	15-20	1
LA2	O. niloticus	hyomandibula	1	15-20	1
LA2	Haplotilapiini indet.	opercular	1		
LA2	C. zillii	2nd precaudal vertebra	1	20-25	1
LA2	Haplotilapiini indet.	preopercular	1	15-20	1
LA2	Haplotilapiini indet.	postcleithrum	1		
LA2	Haplotilapiini indet.	quadrate	1	15-20	1
LA2	Haplotilapiini indet.	precaudal vertebra	10	15-20	1
LA2	Haplotilapiini indet.	precaudal vertebra	2	20-25	2
LA2	Haplotilapiini indet.	lepidotrich	4		
LA2	Haplotilapiini indet.	dorsal or anal pterygophore	4		
LA2	Haplotilapiini indet.	basipterygium	5	15-20	5
LA2	Haplotilapiini indet.	skull roof fragment	6	15-20	1
LA2	Haplotilapiini indet.	caudal vertebra	9	15-20	9
LA2	O. niloticus	cleithrum	9	15-20	1
LA2	Haplotilapiini indet.	basioccipital	1	25-30	1
LA2	Haplotilapiini indet.	cleithrum	1		
LA2	Haplotilapiini indet.	2nd precaudal vertebra	1	30-40	1
LA2	Haplotilapiini indet.	3rd precaudal vertebra	1	25-30	1
LA2	Haplotilapiini indet.	supracleithrum	1		
LA2	C. zillii	hyomandibula	1	15-20	1
LA2	Haplotilapiini indet.	hyomandibula	1		

cultural phase	taxon	skeletal element	NISP	SL in cm	number
LA2	Haplotilapiini indet.	interopercular	1	15-20	1
LA2	O. niloticus	mesethmoid	1	15-20	1
LA2	O. niloticus	opercular	1	15-20	1
LA2	Haplotilapiini indet.	opercular	1	15-20	1
LA2	Haplotilapiini indet.	3rd precaudal vertebra	1	20-25	1
LA2	Haplotilapiini indet.	precaudal vertebra	1	20-25	1
LA2	Haplotilapiini indet.	preopercular	1	20-25	1
LA2	Haplotilapiini indet.	postcleithrum	1	20-25	1
LA2	Haplotilapiini indet.	dorsal or anal pterygophore	1	15-20	1
LA2	Haplotilapiini indet.	dorsal or anal pterygophore	1	15-20	1
LA2	Haplotilapiini indet.	dorsal or anal pterygophore	1	15-20	1
LA2	Haplotilapiini indet.	dorsal or anal pterygophore	1	15-20	1
LA2	Haplotilapiini indet.	dorsal or anal pterygophore	1	15-20	1
LA2	Haplotilapiini indet.	dorsal or anal pterygophore	1	15-20	1
LA2	Haplotilapiini indet.	dorsal or anal pterygophore	1	15-20	1
LA2	Haplotilapiini indet.	dorsal or anal pterygophore	1	10-15	1
LA2	Haplotilapiini indet.	supracleithrum	1	15-20	1
LA2	Haplotilapiini indet.	precaudal vertebra	10	15-20	10
LA2	Haplotilapiini indet.	lepidotrich	17		
LA2	Haplotilapiini indet.	ceratohyale	2		
LA2	Haplotilapiini indet.	skull roof fragment	2	15-20	2
LA2	Haplotilapiini indet.	preopercular	2	15-20	2
LA2	Haplotilapiini indet.	opercular	3		
LA2	Haplotilapiini indet.	basipterygium	5	15-20	5
LA2	Haplotilapiini indet.	caudal vertebra	5	15-20	5
LA2	Haplotilapiini indet.	dorsal or anal pterygophore	5		
LA2	Haplotilapiini indet.	cleithrum	7		
LA2	Haplotilapiini indet.	skull roof fragment	7		
LA2	Haplotilapiini indet.	dorsal or anal pterygophore	1	15-20	1
LA2	Haplotilapiini indet.	cleithrum	2		
LA2	Clarias sp.	articular	1	50-60	1
LA2	Clarias sp.	caudal vertebra	1	40-50	1
LA2	Clarias sp.	cleithrum	1		
LA2	Clarias sp.	precaudal vertebra	1	40-50	1
LA2	Clarias sp.	cranial roof fragment	2		
LA2	Haplotilapiini indet.	articular	1	15-20	1
LA2	Haplotilapiini indet.	basioccipital	1	20-25	1
LA2	O. niloticus	hyomandibula	1	20-25	1
LA2	O. niloticus	hyomandibula	1	15-20	1
LA2	O. niloticus	hyomandibula	1	15-20	1
LA2	O. niloticus	hyomandibula	1	15-20	1
LA2	O. niloticus	hyomandibula	1	10-15	1
LA2	Haplotilapiini indet.	hyomandibula	1	10-15	1
LA2	Haplotilapiini indet.	hyomandibula	1		
LA2	Haplotilapiini indet.	fin spine	1		
LA2	O. niloticus	mesethmoid	1	20-25	1
LA2	Haplotilapiini indet.	mesethmoid	1		
LA2	Haplotilapiini indet.	opercular	1	15-20	1
LA2	Haplotilapiini indet.	2nd precaudal vertebra	1	15-20	1
LA2	Haplotilapiini indet.	precaudal vertebra	1	20-25	1
LA2	Haplotilapiini indet.	dorsal or anal pterygophore	1	15-20	1
LA2	Haplotilapiini indet.	dorsal or anal pterygophore	1	15-20	1
LA2	Haplotilapiini indet.	dorsal or anal pterygophore	1	15-20	1
LA2	Haplotilapiini indet.	dorsal or anal pterygophore	1	15-20	1
LA2	Haplotilapiini indet.	dorsal or anal pterygophore	1		
LA2	Haplotilapiini indet.	supracleithrum	1	15-20	1
LA2	Haplotilapiini indet.	supracleithrum	1	20-25	1
LA2	Haplotilapiini indet.	caudal vertebra	11	15-20	1

cultural phase	taxon	skeletal element	NISP	SL in cm	number
LA2	Haplotilapiini indet.	precaudal vertebra	11	15-20	1
LA2	Haplotilapiini indet.	basipterygium	3	15-20	3
LA2	Haplotilapiini indet.	cleithrum	7		
LA2	Haplotilapiini indet.	skull roof fragment	7		
LA2	Clarias sp.	cranial roof fragment	1		
LA2	Haplotilapiini indet.	fin spine	1		
LA2	Haplotilapiini indet.	precaudal vertebra	3	15-20	3
LA2	Clarias sp.	cleithrum	1	30-40	1
LA2	Clarias sp.	hyomandibula	1		
LA2	Clarias sp.	pectoral spine	1	40-50	1
LA2	Clarias sp.	quadrate	1	40-50	1
LA2	Clarias sp.	cranial roof fragment	3		
LA2	Clarias sp.	caudal vertebra	4	50-60	4
LA2	Haplotilapiini indet.	basipterygium	1		
LA2	Haplotilapiini indet.	cleithrum	1		
LA2	Haplotilapiini indet.	dentary	1		
LA2	Haplotilapiini indet.	precaudal vertebra	1	20-25	1
LA2	Haplotilapiini indet.	precaudal vertebra	1	20-25	1
LA2	Haplotilapiini indet.	postcleithrum	1		
LA2	Haplotilapiini indet.	lepidotrich	2		
LA2	Haplotilapiini indet.	precaudal vertebra	3	15-20	3
LA2	Clarias sp.	basioccipital	1	30-40	1
LA2	Clarias sp.	caudal vertebra	1	60-70	1
LA2	Clarias sp.	ceratohyale	1	30-40	1
LA2	Clarias sp.	ceratohyale	1	40-50	1
LA2	Clarias sp.	cleithrum	1	30-40	1
LA2	Clarias sp.	opercular	1	30-40	1
LA2	Clarias sp.	cranial roof fragment	4		
LA2	Haplotilapiini indet.	postcleithrum	1	15-20	1
LA2	Clarias sp.	caudal vertebra	1	50-60	1
LA2	Clarias sp.	caudal vertebra	1	60-70	1
LA2	Clarias sp.	ceratohyale	1	50-60	1
LA2	Clarias sp.	ceratohyale	1	40-50	1
LA2	Clarias sp.	ceratohyale	1	50-60	1
LA2	Clarias sp.	cleithrum	1	30-40	1
LA2	Clarias sp.	cleithrum	1	20-30	1
LA2	Clarias sp.	cleithrum	1		
LA2	Clarias sp.	coracoid	1	30-40	1
LA2	Clarias sp.	coracoid	1	50-60	1
LA2	Clarias sp.	dentary	1	50-60	1
LA2	Clarias sp.	mesethmoid	1	30-40	1
LA2	Clarias sp.	mesethmoid	1	40-50	1
LA2	Clarias sp.	cranial roof fragment	1	30-40	1
LA2	Clarias sp.	cranial roof fragment	1	50-60	1
LA2	Clarias sp.	cranial roof fragment	1	60-70	1
LA2	Clarias sp.	opercular	1	50-60	1
LA2	Clarias sp.	pectoral spine	1	60-70	1
LA2	Clarias sp.	pectoral spine	1	30-40	1
LA2	Clarias sp.	pectoral spine	1	60-70	1
LA2	Clarias sp.	quadrate	1	40-50	1
LA2	Clarias sp.	caudal vertebra	2	40-50	2
LA2	Clarias sp.	precaudal vertebra	2	40-50	2
LA2	Clarias sp.	cranial roof fragment	6	40-50	6
LA2	Clarias sp.	cranial roof fragment	6		
LA2	Haplotilapiini indet.	basipterygium	1	20-25	1
LA2	Haplotilapiini indet.	hyomandibula	1	15-20	1
LA2	Haplotilapiini indet.	hyomandibula	1	25-30	1
LA2	Haplotilapiini indet.	skull roof fragment	1	15-20	1

cultural phase	taxon	skeletal element	NISP	SL in cm	number
LA2	Haplotilapiini indet.	skull roof fragment	1	20-25	1
LA2	Haplotilapiini indet.	opercular	1	15-20	1
LA2	Haplotilapiini indet.	opercular	1	15-20	1
LA2	Haplotilapiini indet.	opercular	1		
LA2	Haplotilapiini indet.	3rd precaudal vertebra	1	20-25	1
LA2	Haplotilapiini indet.	precaudal vertebra	1	20-25	1
LA2	Haplotilapiini indet.	postcleithrum	1	20-25	1
LA2	Haplotilapiini indet.	dorsal or anal pterygophore	1	15-20	1
LA2	Haplotilapiini indet.	dorsal or anal pterygophore	1	15-20	1
LA2	Haplotilapiini indet.	dorsal or anal pterygophore	1	20-25	1
LA2	Haplotilapiini indet.	skull roof fragment	11		
LA2	Haplotilapiini indet.	precaudal vertebra	2	15-20	2
LA2	Haplotilapiini indet.	precaudal vertebra	2	25-30	2
LA2	Haplotilapiini indet.	preopercular	2		
LA2	Haplotilapiini indet.	cleithrum	8		
LA2	Clarias sp.	cranial roof fragment	2	30-40	1
LA2	Clarias sp.	cranial roof fragment	1	50-60	1
LA2	Haplotilapiini indet.	fin spine	1		
LA2	Haplotilapiini indet.	skull roof fragment	1		
LA2	Haplotilapiini indet.	preopercular	1		
LA2	Haplotilapiini indet.	cleithrum	2		
LA2	Clarias sp.	articular	1	50-60	1
LA2	Clarias sp.	ceratohyale	1	30-40	1
LA2	Clarias sp.	cranial roof fragment	2		
LA2	Clarias sp.	cleithrum	2		
LA2	Clarias sp.	cranial roof fragment	8		
LA2	Clarias sp.	ceratohyale	1	30-40	1
LA2	Clarias sp.	ceratohyale	1	50-60	1
LA2	Clarias sp.	coracoid	1		
LA2	Clarias sp.	mesethmoid	1	70-80	1
LA2	Clarias sp.	cleithrum	1	40-50	1
LA2	Clarias sp.	cleithrum	1	50-60	1
LA2	Clarias sp.	ceratohyale	1	40-50	1
LA2	Clarias sp.	cleithrum	1		
LA2	Clarias sp.	palatinum	1	40-50	1
LA2	Clarias sp.	cranial roof fragment	3		
LA2	Clarias sp.	ceratohyale	1	40-50	1
LA2	Clarias sp.	cleithrum	1	40-50	1
LA2	Clarias sp.	cleithrum	1	60-70	1
LA2	Clarias sp.	cleithrum	1		
LA2	Clarias sp.	cleithrum	1		
LA2	Clarias sp.	coracoid	1	40-50	1
LA2	Clarias sp.	dentary	1	40-50	1
LA2	Clarias sp.	fin ray	1		
LA2	Clarias sp.	cranial roof fragment	1	50-60	1
LA2	Clarias sp.	pectoral spine	1	50-60	1
LA2	Clarias sp.	quadrate	1	70-80	1
LA2	Clarias sp.	coracoid	2	50-60	1
LA2	Clarias sp.	cranial roof fragment	2	20-30	2
LA2	Clarias sp.	cranial roof fragment	2		
LA2	Clarias sp.	cranial roof fragment	8		
LA2	Clarias sp.	caudal vertebra	1	60-70	1
LA2	Clarias sp.	cleithrum	1		
LA2	Clarias sp.	pectoral spine	1	50-60	1
LA2	Clarias sp.	cranial roof fragment	5		
LA2	Clarias sp.	caudal vertebra	1	60-70	1
LA2	Clarias sp.	cleithrum	1		
LA2	Clarias sp.	epihyale	1	30-40	1

cultural phase	taxon	skeletal element	NISP	SL in cm	number
LA2	Clarias sp.	pectoral spine	1	40-50	1
LA2	Clarias sp.	caudal vertebra	2	40-50	2
LA2	Clarias sp.	precaudal vertebra	2	40-50	2
LA2	Clarias sp.	cranial roof fragment	8	50-60	1
LA2	Clarias sp.	caudal vertebra	1	40-50	1
LA2	Clarias sp.	pectoral spine	1	40-50	1
LA2	Haplotilapiini indet.	precaudal vertebra	1	20-25	1
LA2	Haplotilapiini indet.	cleithrum	1		
LA2	Haplotilapiini indet.	opercular	1	20-25	1
LA2	Haplotilapiini indet.	basipterygium	1	15-20	1
LA2	Haplotilapiini indet.	caudal vertebra	1	15-20	1
LA2	Haplotilapiini indet.	cleithrum	1		
LA2	Haplotilapiini indet.	cleithrum	1		
LA2	Haplotilapiini indet.	skull roof fragment	1		
LA2	Haplotilapiini indet.	3rd precaudal vertebra	1	20-25	1
LA2	Haplotilapiini indet.	3rd precaudal vertebra	1	20-25	1
LA2	Haplotilapiini indet.	cleithrum	1		
LA2	Haplotilapiini indet.	skull roof fragment	1	20-25	1
LA2	Haplotilapiini indet.	precaudal vertebra	1	15-20	1
LA2	Haplotilapiini indet.	dorsal or anal pterygophore	1		
LA2	Haplotilapiini indet.	scapula	1		
LA2	Haplotilapiini indet.	supracleithrum	1		
LA2	Haplotilapiini indet.	basipterygium	2	20-25	2
LA2	Haplotilapiini indet.	caudal vertebra	2	15-20	2
LA2	Haplotilapiini indet.	skull roof fragment	2		
LA2	Haplotilapiini indet.	cleithrum	3		
LA2	Haplotilapiini indet.	lepidotrich	4		
LA2	Haplotilapiini indet.	dorsal or anal pterygophore	1	20-25	1
LA2	Clarias sp.	epihyale	1	80-90	1
LA2	Clarias sp.	caudal vertebra	1	60-70	1
LA2	Clarias sp.	ceratohyale	1	50-60	1
LA2	Clarias sp.	ceratohyale	1	60-70	1
LA2	Clarias sp.	mesethmoid	1	70-80	1
LA2	Clarias sp.	cranial roof fragment	5		
LA2	Clarias sp.	articular	1	80-90	1
LA2	Clarias sp.	caudal vertebra	1	60-70	1
LA2	Clarias sp.	dentary	1	30-40	1
LA2	Clarias sp.	cranial roof fragment	1	60-70	1
LA2	Clarias sp.	pectoral spine	1	40-50	1
LA2	Clarias sp.	pectoral spine	1	70-80	1
LA2	Clarias sp.	quadrate	1	40-50	1
LA2	Clarias sp.	cranial roof fragment	14		
LA2	Clarias sp.	cranial roof fragment	2	30-40	2
LA2	Clarias sp.	articular	1	50-60	1
LA2	Clarias sp.	caudal vertebra	1	70-80	1
LA2	Clarias sp.	ceratohyale	1		
LA2	Clarias sp.	epihyale	1		
LA2	Clarias sp.	cranial roof fragment	1	20-30	1
LA2	Clarias sp.	cranial roof fragment	1	30-40	1
LA2	Clarias sp.	opercular	1	50-60	1
LA2	Clarias sp.	precaudal vertebra	1	50-60	1
LA2	Clarias sp.	vomerine toothplate	1	60-70	1
LA2	Clarias sp.	cranial roof fragment	16		
LA2	Clarias sp.	coracoid	2		
LA2	Clarias sp.	cleithrum	3		
LA2	Clarias sp.	caudal vertebra	1	70-80	1
LA2	Clarias sp.	cleithrum	1	40-50	1
LA2	Clarias sp.	coracoid	1		

cultural phase	taxon	skeletal element	NISP	SL in cm	number
LA2	Clarias sp.	pectoral spine	1	50-60	1
LA2	Clarias sp.	articular	1	60-70	1
LA2	Clarias sp.	caudal vertebra	1	40-50	1
LA2	Clarias sp.	ceratohyale	1	60-70	1
LA2	Clarias sp.	ceratohyale	1	50-60	1
LA2	Clarias sp.	ceratohyale	1	50-60	1
LA2	Clarias sp.	ceratohyale	1		
LA2	Clarias sp.	cleithrum	1	60-70	1
LA2	Clarias sp.	cleithrum	1	70-80	1
LA2	Clarias sp.	coracoid	1	50-60	1
LA2	Clarias sp.	costa	1		
LA2	Clarias sp.	hyomandibula	1	50-60	1
LA2	Clarias sp.	hypohyale	1	70-80	1
LA2	Clarias sp.	cranial roof fragment	1	20-30	1
LA2	Clarias sp.	cranial roof fragment	1	50-60	1
LA2	Clarias sp.	opercular	1		
LA2	Clarias sp.	palatinum	1	30-40	1
LA2	Clarias sp.	precaudal vertebra	1	40-50	1
LA2	Clarias sp.	pectoral spine	1	40-50	1
LA2	Clarias sp.	pectoral spine	1		
LA2	Clarias sp.	quadrate	1	40-50	1
LA2	Clarias sp.	quadrate	1	60-70	1
LA2	Clarias sp.	urohyale	1	50-60	1
LA2	Clarias sp.	cleithrum	2		
LA2	Clarias sp.	cranial roof fragment	2		
LA2	Clarias sp.	cranial roof fragment	4	40-50	4
LA2	Clarias sp.	articular	1	60-70	1
LA2	Clarias sp.	coracoid	1		
LA2	Clarias sp.	hyomandibula	1		
LA2	Clarias sp.	mesethmoid	1	50-60	1
LA2	Clarias sp.	cranial roof fragment	1	60-70	1
LA2	Clarias sp.	pectoral spine	1	70-80	1
LA2	Clarias sp.	premaxilla	1	60-70	1
LA2	Clarias sp.	quadrate	1	30-40	1
LA2	Clarias sp.	caudal vertebra	2	50-60	2
LA2	Clarias sp.	cranial roof fragment	2	40-50	2
LA2	Clarias sp.	cranial roof fragment	3	50-60	3
LA2	Clarias sp.	cranial roof fragment	4		
LA2	Clarias sp.	coracoid	1	40-50	1
LA2	Clarias sp.	cranial roof fragment	2		
LA2	Clarias sp.	caudal vertebra	1	50-60	1
LA2	Clarias sp.	caudal vertebra	1	60-70	1
LA2	Clarias sp.	cleithrum	1		
LA2	Clarias sp.	cranial roof fragment	1		
LA2	Clarias sp.	opercular	1	50-60	1
LA2	Clarias sp.	palatinum	1	70-80	1
LA2	Clarias sp.	pectoral spine	1	60-70	1
LA2	Clarias sp.	cranial roof fragment	2		
LA2	Clarias sp.	articular	1	50-60	1
LA2	Clarias sp.	articular	1	50-60	1
LA2	Clarias sp.	articular	1	60-70	1
LA2	Clarias sp.	caudal vertebra	1	40-50	1
LA2	Clarias sp.	caudal vertebra	1	60-70	1
LA2	Clarias sp.	ceratohyale	1	40-50	1
LA2	Clarias sp.	ceratohyale	1	50-60	1
LA2	Clarias sp.	cleithrum	1	30-40	1
LA2	Clarias sp.	cleithrum	1	30-40	1
LA2	Clarias sp.	cleithrum	1	60-70	1

cultural phase	taxon	skeletal element	NISP	SL in cm	number
LA2	Clarias sp.	cleithrum	1	40-50	1
LA2	Clarias sp.	dentary	1	30-40	1
LA2	Clarias sp.	hyomandibula	1	30-40	1
LA2	Clarias sp.	mesethmoid	1	40-50	1
LA2	Clarias sp.	cranial roof fragment	1	40-50	1
LA2	Clarias sp.	opercular	1	40-50	1
LA2	Clarias sp.	pectoral spine	1	50-60	1
LA2	Clarias sp.	quadrate	1	30-40	1
LA2	Clarias sp.	cranial roof fragment	15		
LA2	Clarias sp.	cranial roof fragment	2	50-60	2
LA2	Clarias sp.	coracoid	3	60-70	2
LA2	Clarias sp.	cranial roof fragment	3	60-70	3
LA2	Clarias sp.	articular	1	40-50	1
LA2	Clarias sp.	articular	1	60-70	1
LA2	Clarias sp.	articular	1	60-70	1
LA2	Clarias sp.	articular	1	30-40	1
LA2	Clarias sp.	articular	1	40-50	1
LA2	Clarias sp.	basioccipital	1	50-60	1
LA2	Clarias sp.	coracoid	1		
LA2	Clarias sp.	hyomandibula	1	50-60	1
LA2	Clarias sp.	unidentified	1		
LA2	Clarias sp.	mesethmoid	1	40-50	1
LA2	Clarias sp.	opercular	1	60-70	1
LA2	Clarias sp.	precaudal vertebra	1	50-60	1
LA2	Clarias sp.	precaudal vertebra	1	60-70	1
LA2	Clarias sp.	pectoral spine	1	60-70	1
LA2	Clarias sp.	pectoral spine	1	60-70	1
LA2	Clarias sp.	pectoral spine	1	50-60	1
LA2	Clarias sp.	pectoral spine	1	80-90	1
LA2	Clarias sp.	pectoral spine	1		
LA2	Clarias sp.	quadrate	1	40-50	1
LA2	Clarias sp.	quadrate	1	60-70	1
LA2	Clarias sp.	quadrate	1	30-40	1
LA2	Clarias sp.	cranial roof fragment	17		
LA2	Clarias sp.	caudal vertebra	2	50-60	2
LA2	Clarias sp.	ceratohyale	2	60-70	2
LA2	Clarias sp.	caudal vertebra	3	40-50	3
LA2	Clarias sp.	cranial roof fragment	3	30-40	3
LA2	Clarias sp.	cranial roof fragment	3	40-50	3
LA2	Clarias sp.	cranial roof fragment	3	50-60	3
LA2	Clarias sp.	caudal vertebra	4	60-70	4
LA2	Clarias sp.	cranial roof fragment	5	60-70	5
LA2	Clarias sp.	cleithrum	6		
LA2	Clarias sp.	articular	1		
LA2	Clarias sp.	basioccipital	1	50-60	1
LA2	Clarias sp.	caudal vertebra	1	60-70	1
LA2	Clarias sp.	cleithrum	1	50-60	1
LA2	Clarias sp.	cleithrum	1	40-50	1
LA2	Clarias sp.	cleithrum	1		
LA2	Clarias sp.	coracoid	1	60-70	1
LA2	Clarias sp.	hyomandibula	1	50-60	1
LA2	Clarias sp.	mesethmoid	1	50-60	1
LA2	Clarias sp.	cranial roof fragment	1	50-60	1
LA2	Clarias sp.	opercular	1	50-60	1
LA2	Clarias sp.	opercular	1	60-70	1
LA2	Clarias sp.	opercular	1		
LA2	Clarias sp.	precaudal vertebra	1	40-50	1
LA2	Clarias sp.	pectoral spine	1	40-50	1

cultural phase	taxon	skeletal element	NISP	SL in cm	number
LA2	Clarias sp.	pectoral spine	1	50-60	1
LA2	Clarias sp.	quadrate	1	60-70	1
LA2	Clarias sp.	cranial roof fragment	10		
LA2	Clarias sp.	cranial roof fragment	4	70-80	4
LA2	Haplotilapiini indet.	opercular	1	20-25	1
LA2	Haplotilapiini indet.	opercular	1		
LA2	Haplotilapiini indet.	cleithrum	1		
LA2	Haplotilapiini indet.	fin spine	1		
LA2	Haplotilapiini indet.	precaudal vertebra	1	15-20	1
LA2	O. niloticus	hyomandibula	1	20-25	1
LA2	Haplotilapiini indet.	basipterygium	1	15-20	1
LA2	Haplotilapiini indet.	skull roof fragment	1		
LA2	Haplotilapiini indet.	preopercular	1		
LA2	Haplotilapiini indet.	cleithrum	2		
LA2	Haplotilapiini indet.	basipterygium	1	20-25	1
LA2	Haplotilapiini indet.	maxilla	1	20-25	1
LA2	Haplotilapiini indet.	opercular	1		
LA2	Haplotilapiini indet.	dorsal or anal pterygophore	1	20-25	1
LA2	Haplotilapiini indet.	supracleithrum	1	20-25	1
LA2	Clarias sp.	caudal vertebra	1	60-70	1
LA2	Clarias sp.	cleithrum	1	40-50	1
LA2	Clarias sp.	urohyale	1	60-70	1
LA2	Clarias sp.	cranial roof fragment	4		
LA2	Clarias sp.	ceratohyale	1	50-60	1
LA2	Clarias sp.	cleithrum	1		
LA2	Clarias sp.	cranial roof fragment	1		
LA2	Haplotilapiini indet.	preopercular	1	25-30	1
LA2	Clarias sp.	cranial roof fragment	1		
LA2	Clarias sp.	pectoral spine	1	70-80	1
LA2	Clarias sp.	cleithrum	1		
LA2	Clarias sp.	quadrate	1	30-40	1
LA2	Clarias sp.	cranial roof fragment	4	60-70	2
LA2	Clarias sp.	articular	1	50-60	1
LA2	Clarias sp.	caudal vertebra	1	60-70	1
LA2	Clarias sp.	cranial roof fragment	4	30-40	1
LA2	Clarias sp.	coracoid	1	60-70	1
LA2	Clarias sp.	dentary	1	50-60	1
LA2	Clarias sp.	cranial roof fragment	1	40-50	1
LA2	Clarias sp.	cranial roof fragment	1	50-60	1
LA2	Clarias sp.	cranial roof fragment	1	60-70	1
LA2	Clarias sp.	caudal vertebra	2	60-70	2
LA2	Clarias sp.	cranial roof fragment	7		
LA2	Haplotilapiini indet.	hyomandibula	1	20-25	1
LA2	Haplotilapiini indet.	cleithrum	1		
LA2	Haplotilapiini indet.	dorsal or anal pterygophore	1	15-20	1
LA2	Haplotilapiini indet.	dorsal or anal pterygophore	1	25-30	1
LA2	Clarias sp.	cleithrum	1	50-60	1
LA2	Clarias sp.	coracoid	1	60-70	1
LA2	Clarias sp.	cranial roof fragment	7	60-70	1
LA2	Clarias sp.	articular	1	30-40	1
LA2	Clarias sp.	cranial roof fragment	1	30-40	1
LA2	Clarias sp.	precaudal vertebra	1	60-70	1
LA2	Clarias sp.	pectoral spine	1		
LA2	Clarias sp.	cleithrum	2		
LA2	Clarias sp.	cranial roof fragment	3	60-70	3
LA2	Clarias sp.	unidentified	4		
LA2	Clarias sp.	caudal vertebra	6	60-70	6
LA2	Clarias sp.	cranial roof fragment	6		

cultural phase	taxon	skeletal element	NISP	SL in cm	number
LA2	Clarias sp.	articular	1	40-50	1
LA2	Clarias sp.	dentary	1	40-50	1
LA2	Clarias sp.	cranial roof fragment	1	30-40	1
LA2	Clarias sp.	cranial roof fragment	1	60-70	1
LA2	Clarias sp.	precaudal vertebra	1	60-70	1
LA2	Clarias sp.	vomerine toothplate	1		
LA2	Clarias sp.	cleithrum	2	40-50	1
LA2	Clarias sp.	caudal vertebra	4	60-70	4
LA2	Haplotilapiini indet.	3rd precaudal vertebra	1	20-25	1
LA2	Haplotilapiini indet.	fin spine	1		
LA2	Clarias sp.	opercular	1	50-60	1
LA2	Clarias sp.	cranial roof fragment	2	60-70	2
LA2	Clarias sp.	cranial roof fragment	1		
LA2	Haplotilapiini indet.	cleithrum	1	15-20	1
LA2	Clarias sp.	coracoid	1	50-60	1
LA2	Clarias sp.	cranial roof fragment	1	50-60	1
LA2	Clarias sp.	cleithrum	1	40-50	1
LA2	Clarias sp.	cleithrum	1		
LA2	Clarias sp.	mesethmoid	1	40-50	1
LA2	Clarias sp.	mesethmoid	1	40-50	1
LA2	Clarias sp.	cranial roof fragment	1	50-60	1
LA2	Clarias sp.	cranial roof fragment	1	50-60	1
LA2	Clarias sp.	cranial roof fragment	1	60-70	1
LA2	Clarias sp.	cranial roof fragment	1	60-70	1
LA2	Clarias sp.	parasphenoid	1	40-50	1
LA2	Clarias sp.	precaudal vertebra	1	40-50	1
LA2	Clarias sp.	precaudal vertebra	1	50-60	1
LA2	Clarias sp.	caudal vertebra	2	50-60	2
LA2	Clarias sp.	caudal vertebra	2	60-70	2
LA2	Clarias sp.	unidentified	2		
LA2	Clarias sp.	cranial roof fragment	2	30-40	2
LA2	Clarias sp.	cranial roof fragment	3	40-50	3
LA2	Clarias sp.	caudal vertebra	4	60-70	4
LA2	Clarias sp.	cranial roof fragment	9		
LA2	Clarias sp.	caudal vertebra	1	40-50	1
LA2	Clarias sp.	caudal vertebra	1	50-60	1
LA2	Clarias sp.	ceratohyale	1	50-60	1
LA2	Clarias sp.	cleithrum	1	40-50	1
LA2	Clarias sp.	mesethmoid	1	50-60	1
LA2	Clarias sp.	precaudal vertebra	1	30-40	1
LA2	Clarias sp.	quadrate	1	60-70	1
LA2	Clarias sp.	vomerine toothplate	1	50-60	1
LA2	Clarias sp.	caudal vertebra	3	60-70	3
LA2	Clarias sp.	cranial roof fragment	5		
LA2	Clarias sp.	articular	1	50-60	1
LA2	Clarias sp.	ceratohyale	1	30-40	1
LA2	Clarias sp.	cleithrum	1	50-60	1
LA2	Clarias sp.	costa	1		
LA2	Clarias sp.	cranial roof fragment	1	70-80	1
LA2	Clarias sp.	opercular	1	50-60	1
LA2	Clarias sp.	cranial roof fragment	2	60-70	2
LA2	Clarias sp.	caudal vertebra	3	60-70	3
LA2	Clarias sp.	cranial roof fragment	6		
LA2	Haplotilapiini indet.	caudal vertebra	1	15-20	1
LA2	Haplotilapiini indet.	cleithrum	1		
LA2	Haplotilapiini indet.	fin spine	1		
LA2	Haplotilapiini indet.	skull roof fragment	1	15-20	1
LA2	Haplotilapiini indet.	skull roof fragment	1	20-25	1

cultural phase	taxon	skeletal element	NISP	SL in cm	number
LA2	Haplotilapiini indet.	2nd precaudal vertebra	1	25-30	1
LA2	Haplotilapiini indet.	precaudal vertebra	1	15-20	1
LA2	Haplotilapiini indet.	precaudal vertebra	1	20-25	1
LA2	Haplotilapiini indet.	postcleithrum	1	15-20	1
LA2	Haplotilapiini indet.	dorsal or anal pterygophore	1	15-20	1
LA2	Haplotilapiini indet.	dorsal or anal pterygophore	1	15-20	1
LA2	Haplotilapiini indet.	dorsal or anal pterygophore	1	20-25	1
LA2	Haplotilapiini indet.	dorsal or anal pterygophore	1	25-30	1
LA2	Haplotilapiini indet.	cleithrum	2	20-25	1
LA2	Haplotilapiini indet.	caudal vertebra	1	25-30	1
LA2	Haplotilapiini indet.	cleithrum	1	25-30	1
LA2	Haplotilapiini indet.	skull roof fragment	1	25-30	1
LA2	Haplotilapiini indet.	opercular	1	15-20	1
LA2	Haplotilapiini indet.	preopercular	1	15-20	1
LA2	Haplotilapiini indet.	postcleithrum	1		
LA2	Haplotilapiini indet.	dorsal or anal pterygophore	1		
LA2	Haplotilapiini indet.	caudal vertebra	2	15-20	2
LA2	Haplotilapiini indet.	skull roof fragment	2		
LA2	Haplotilapiini indet.	cleithrum	3		
LA2	Haplotilapiini indet.	articular	1	25-30	1
LA2	Haplotilapiini indet.	caudal vertebra	1	15-20	1
LA2	Haplotilapiini indet.	caudal vertebra	1	25-30	1
LA2	Haplotilapiini indet.	skull roof fragment	1		
LA2	O. niloticus	premaxilla	1	25-30	1
LA2	Haplotilapiini indet.	dorsal or anal pterygophore	1	15-20	1
LA2	Haplotilapiini indet.	dorsal or anal pterygophore	1		
LA2	Haplotilapiini indet.	lepidotrich	2		
LA2	Haplotilapiini indet.	cleithrum	4		
LA2	Clarias sp.	cranial roof fragment	1		
LA2	Clarias sp.	cleithrum	1		
LA2	Clarias sp.	cranial roof fragment	1		
LA2	Clarias sp.	articular	1	40-50	1
LA2	Clarias sp.	coracoid	1	60-70	1
LA2	Clarias sp.	pectoral spine	1	70-80	1
LA2	Clarias sp.	cleithrum	2	50-60	1
LA2	Clarias sp.	cranial roof fragment	3		
LA2	Clarias sp.	articular	1	50-60	1
LA2	Clarias sp.	caudal vertebra	1	50-60	1
LA2	Clarias sp.	cranial roof fragment	3		
LA2	Clarias sp.	articular	1	30-40	1
LA2	Clarias sp.	cleithrum	1	50-60	1
LA2	Clarias sp.	cleithrum	1	30-40	1
LA2	Clarias sp.	coracoid	1		
LA2	Clarias sp.	coracoid	1		
LA2	Clarias sp.	dentary	1	30-40	1
LA2	Clarias sp.	epihyale	1	50-60	1
LA2	Clarias sp.	cranial roof fragment	1	50-60	1
LA2	Clarias sp.	cranial roof fragment	1	70-80	1
LA2	Clarias sp.	cranial roof fragment	1	80-90	1
LA2	Clarias sp.	quadrate	1	30-40	1
LA2	Clarias sp.	quadrate	1	50-60	1
LA2	Clarias sp.	caudal vertebra	2	40-50	2
LA2	Clarias sp.	caudal vertebra	2	50-60	2
LA2	Clarias sp.	caudal vertebra	2	60-70	2
LA2	Clarias sp.	cranial roof fragment	2	40-50	2
LA2	Clarias sp.	cranial roof fragment	4		
LA2	Clarias sp.	cranial roof fragment	5		
LA2	Clarias sp.	cleithrum	1		

cultural phase	taxon	skeletal element	NISP	SL in cm	number
LA2	Clarias sp.	vomerine toothplate	1	50-60	1
LA2	Clarias sp.	caudal vertebra	2	50-60	2
LA2	Clarias sp.	coracoid	2		
LA2	Clarias sp.	cranial roof fragment	2		
LA2	Haplotilapiini indet.	skull roof fragment	1	15-20	1
LA2	Haplotilapiini indet.	opercular	1	15-20	1
LA2	Haplotilapiini indet.	palatinum	1	25-30	1
LA2	Haplotilapiini indet.	skull roof fragment	2	20-25	1
LA2	Haplotilapiini indet.	caudal vertebra	1	25-30	1
LA2	Haplotilapiini indet.	cleithrum	1		
LA2	Haplotilapiini indet.	precaudal vertebra	1	20-25	1
LA2	Haplotilapiini indet.	precaudal vertebra	1	25-30	1
LA2	Haplotilapiini indet.	cleithrum	2	25-30	2
LA2	Haplotilapiini indet.	skull roof fragment	2		
LA2	Haplotilapiini indet.	cleithrum	1		
LA2	Clarias sp.	pectoral spine	1	70-80	1
LA2	Clarias sp.	quadrate	1	40-50	1
LA2	Clarias sp.	coracoid	1		
LA2	Clarias sp.	cranial roof fragment	1		
LA2	Clarias sp.	cranial roof fragment	1		
LA2	Clarias sp.	cleithrum	2	30-40	1
LA2	Clarias sp.	ceratohyale	1		
LA2	Haplotilapiini indet.	precaudal vertebra	1	15-20	1
LA2	Clarias sp.	caudal vertebra	1	60-70	1
LA2	Clarias sp.	dentary	1	30-40	1
LA2	Clarias sp.	opercular	1	50-60	1
LA2	Clarias sp.	pectoral spine	1	70-80	1
LA2	Clarias sp.	articular	1	40-50	1
LA2	Clarias sp.	articular	1	30-40	1
LA2	Clarias sp.	articular	1	40-50	1
LA2	Clarias sp.	articular	1	50-60	1
LA2	Clarias sp.	articular	1	50-60	1
LA2	Clarias sp.	basioccipital	1	60-70	1
LA2	Clarias sp.	coracoid	1	40-50	1
LA2	Clarias sp.	coracoid	1	70-80	1
LA2	Clarias sp.	dentary	1	50-60	1
LA2	Clarias sp.	epihyale	1	60-70	1
LA2	Clarias sp.	epihyale	1	70-80	1
LA2	Clarias sp.	hypohyale	1	60-70	1
LA2	Clarias sp.	mesethmoid	1	60-70	1
LA2	Clarias sp.	mesethmoid	1	50-60	1
LA2	Clarias sp.	mesethmoid	1	50-60	1
LA2	Clarias sp.	mesethmoid	1	40-50	1
LA2	Clarias sp.	mesethmoid	1		
LA2	Clarias sp.	cranial roof fragment	1	30-40	1
LA2	Clarias sp.	cranial roof fragment	1	70-80	1
LA2	Clarias sp.	precaudal vertebra	1	40-50	1
LA2	Clarias sp.	pectoral spine	1	60-70	1
LA2	Clarias sp.	pectoral spine	1	70-80	1
LA2	Clarias sp.	pectoral spine	1	30-40	1
LA2	Clarias sp.	quadrate	1	30-40	1
LA2	Clarias sp.	quadrate	1	40-50	1
LA2	Clarias sp.	quadrate	1	50-60	1
LA2	Clarias sp.	urohyale	1	50-60	1
LA2	Clarias sp.	vomerine toothplate	1	60-70	1
LA2	Clarias sp.	caudal vertebra	2	40-50	2
LA2	Clarias sp.	ceratohyale	2		
LA2	Clarias sp.	dentary	2	60-70	2

cultural phase	taxon	skeletal element	NISP	SL in cm	number
LA2	Clarias sp.	cranial roof fragment	2	40-50	2
LA2	Clarias sp.	precaudal vertebra	2	60-70	2
LA2	Clarias sp.	vomerine toothplate	2	70-80	2
LA2	Clarias sp.	coracoid	3		
LA2	Clarias sp.	caudal vertebra	5	50-60	5
LA2	Clarias sp.	cranial roof fragment	59		
LA2	Clarias sp.	cranial roof fragment	6	50-60	6
LA2	Clarias sp.	cranial roof fragment	7	60-70	7
LA2	Clarias sp.	caudal vertebra	8	60-70	8
LA2	Clarias sp.	cleithrum	9		
LA2	Clarias sp.	ceratohyale	1	40-50	1
LA2	Clarias sp.	hypohyale	1	50-60	1
LA2	Clarias sp.	cleithrum	2		
LA2	Clarias sp.	cranial roof fragment	3		
LA2	Haplotilapiini indet.	fin spine	1		
LA2	Haplotilapiini indet.	opercular	1		
LA2	Haplotilapiini indet.	supracleithrum	1	25-30	1
LA2	Haplotilapiini indet.	caudal vertebra	2	15-20	2
LA2	Haplotilapiini indet.	cleithrum	2	25-30	2
LA2	Haplotilapiini indet.	precaudal vertebra	2	15-20	2
LA2	Clarias sp.	basioccipital	1	50-60	1
LA2	Clarias sp.	cleithrum	1	40-50	1
LA2	Clarias sp.	unidentified	1		
LA2	Clarias sp.	vomerine toothplate	1		
LA2	Clarias sp.	parasphenoid	2	40-50	2
LA2	Clarias sp.	caudal vertebra	5	50-60	5
LA2	Clarias sp.	cranial roof fragment	9		
LA2	Clarias sp.	caudal vertebra	1	60-70	1
LA2	Clarias sp.	cranial roof fragment	1	30-40	1
LA2	Clarias sp.	cranial roof fragment	1	60-70	1
LA2	Clarias sp.	cranial roof fragment	1		
LA2	Clarias sp.	caudal vertebra	2	50-60	2
LA2	Clarias sp.	cranial roof fragment	2	40-50	2
LA2	Haplotilapiini indet.	caudal vertebra	1	15-20	1
LA2	Haplotilapiini indet.	cleithrum	1		
LA2	Haplotilapiini indet.	2nd precaudal vertebra	1	25-30	1
LA2	Haplotilapiini indet.	precaudal vertebra	1	15-20	1
LA2	Haplotilapiini indet.	dorsal or anal pterygophore	1		
LA2	Haplotilapiini indet.	cleithrum	1		
LA2	Clarias sp.	articular	1	50-60	1
LA2	Clarias sp.	basioccipital	1	50-60	1
LA2	Clarias sp.	ceratohyale	1	30-40	1
LA2	Clarias sp.	hyomandibula	1	30-40	1
LA2	Clarias sp.	pectoral spine	1	40-50	1
LA2	Clarias sp.	vomerine toothplate	1	50-60	1
LA2	Clarias sp.	cranial roof fragment	3		
LA2	Haplotilapiini indet.	basipterygium	1	15-20	1
LA2	Haplotilapiini indet.	cleithrum	1		
LA2	Haplotilapiini indet.	2nd precaudal vertebra	1	20-25	1
LA2	Haplotilapiini indet.	precaudal vertebra	1	15-20	1
LA2	Haplotilapiini indet.	dorsal or anal pterygophore	1	15-20	1
LA2	Clarias sp.	caudal vertebra	1	40-50	1
LA2	Clarias sp.	ceratohyale	1	40-50	1
LA2	Clarias sp.	vomerine toothplate	1		
LA2	Clarias sp.	cleithrum	3		
LA2	Clarias sp.	cranial roof fragment	4		
LA2	Haplotilapiini indet.	parasphenoid	1		
LA2	Haplotilapiini indet.	cleithrum	3		

cultural phase	taxon	skeletal element	NISP	SL in cm	number
LA2	Clarias sp.	precaudal vertebra	1	50-60	1
LA2	Clarias sp.	pectoral spine	1	40-50	1
LA2	Clarias sp.	caudal vertebra	2	60-70	2
LA2	Clarias sp.	cranial roof fragment	5		
LA2	Clarias sp.	cleithrum	1	30-40	1
LA2	Clarias sp.	cranial roof fragment	2	40-50	1
LA2	Haplotilapiini indet.	caudal vertebra	1	15-20	1
LA2	O. niloticus	hyomandibula	1	20-25	1
LA2	Haplotilapiini indet.	dorsal or anal pterygophore	1	15-20	1
LA2	Haplotilapiini indet.	dorsal or anal pterygophore	1		
LA2	Haplotilapiini indet.	subopercular	1		
LA2	Haplotilapiini indet.	supracleithrum	1	15-20	1
LA2	Haplotilapiini indet.	skull roof fragment	3		
LA3	Clarias sp.	articular	1	60-70	1
LA3	Clarias sp.	articular	1	50-60	1
LA3	Clarias sp.	articular	1	50-60	1
LA3	Clarias sp.	articular	1	60-70	1
LA3	Clarias sp.	articular	1	60-70	1
LA3	Clarias sp.	basioccipital	1	70-80	1
LA3	Clarias sp.	ceratohyale	1	70-80	1
LA3	Clarias sp.	cleithrum	1	60-70	1
LA3	Clarias sp.	cleithrum	1	50-60	1
LA3	Clarias sp.	cleithrum	1	50-60	1
LA3	Clarias sp.	coracoid	1		
LA3	Clarias sp.	dentary	1	50-60	1
LA3	Clarias sp.	dentary	1	50-60	1
LA3	Clarias sp.	hypohyale	1	70-80	1
LA3	Clarias sp.	mesethmoid	1	60-70	1
LA3	Clarias sp.	opercular	1	40-50	1
LA3	Clarias sp.	opercular	1	40-50	1
LA3	Clarias sp.	opercular	1	50-60	1
LA3	Clarias sp.	pectoral spine	1	70-80	1
LA3	Clarias sp.	pectoral spine	1	70-80	1
LA3	Clarias sp.	pectoral spine	1	60-70	1
LA3	Clarias sp.	pectoral spine	1	40-50	1
LA3	Clarias sp.	pectoral spine	1	70-80	1
LA3	Clarias sp.	pectoral spine	1	60-70	1
LA3	Clarias sp.	pectoral spine	1		
LA3	Clarias sp.	quadrate	1	50-60	1
LA3	Clarias sp.	quadrate	1	60-70	1
LA3	Clarias sp.	urohyale	1	50-60	1
LA3	Clarias sp.	urohyale	1	50-60	1
LA3	Clarias sp.	vomerine toothplate	1	50-60	1
LA3	Clarias sp.	vomerine toothplate	1	60-70	1
LA3	Clarias sp.	cranial roof fragment	12	60-70	12
LA3	Clarias sp.	caudal vertebra	16	60-70	16
LA3	Clarias sp.	coracoid	2	60-70	2
LA3	Clarias sp.	cranial roof fragment	2	50-60	2
LA3	Clarias sp.	precaudal vertebra	2	50-60	2
LA3	Clarias sp.	premaxilla	2	50-60	2
LA3	Clarias sp.	cranial roof fragment	20		
LA3	Clarias sp.	caudal vertebra	4	50-60	4
LA3	Clarias sp.	cranial roof fragment	5	70-80	5
LA3	Clarias sp.	precaudal vertebra	5	60-70	5
LA3	Clarias sp.	articular	1	40-50	1
LA3	Clarias sp.	articular	1	40-50	1
LA3	Clarias sp.	basioccipital	1	50-60	1
LA3	Clarias sp.	caudal vertebra	1	30-40	1

cultural phase	taxon	skeletal element	NISP	SL in cm	number
LA3	Clarias sp.	ceratohyale	1	70-80	1
LA3	Clarias sp.	cleithrum	1	40-50	1
LA3	Clarias sp.	dentary	1	30-40	1
LA3	Clarias sp.	dentary	1	50-60	1
LA3	Clarias sp.	cranial roof fragment	1	40-50	1
LA3	Clarias sp.	cranial roof fragment	1	50-60	1
LA3	Clarias sp.	hyomandibula	1	50-60	1
LA3	Clarias sp.	hyomandibula	1	40-50	1
LA3	Clarias sp.	hypohyale	1	70-80	1
LA3	Clarias sp.	opercular	1	30-40	1
LA3	Clarias sp.	opercular	1		
LA3	Clarias sp.	palatinum	1	60-70	1
LA3	Clarias sp.	palatinum	1	70-80	1
LA3	Clarias sp.	palatinum	1	40-50	1
LA3	Clarias sp.	pectoral spine	1	50-60	1
LA3	Clarias sp.	pectoral spine	1	60-70	1
LA3	Clarias sp.	quadrate	1	50-60	1
LA3	Clarias sp.	costa	1		
LA3	Clarias sp.	urohyale	1	60-70	1
LA3	Clarias sp.	cranial roof fragment	14		
LA3	Clarias sp.	caudal vertebra	2	50-60	2
LA3	Clarias sp.	cranial roof fragment	2	70-80	2
LA3	Clarias sp.	hypohyale	2	60-70	1
LA3	Clarias sp.	caudal vertebra	4	60-70	4
LA3	Clarias sp.	precaudal vertebra	7	60-70	7
LA3	Haplotilapiini indet.	basioccipital	1	25-30	1
LA3	Haplotilapiini indet.	skull roof fragment	1	25-30	1
LA3	Haplotilapiini indet.	hyomandibula	1	20-25	1
LA3	Haplotilapiini indet.	fin spine	1		
LA3	Haplotilapiini indet.	opercular	1		
LA3	Haplotilapiini indet.	palatinum	1	15-20	1
LA3	O. niloticus	premaxilla	1	25-30	1
LA3	Haplotilapiini indet.	preopercular	1		
LA3	Haplotilapiini indet.	dorsal or anal pterygophore	1		
LA3	Haplotilapiini indet.	supracleithrum	1	25-30	1
LA3	Haplotilapiini indet.	neurocranium fragment	2		
LA3	Haplotilapiini indet.	precaudal vertebra	2	15-20	1
LA3	Clarias sp.	articular	1	50-60	1
LA3	Clarias sp.	basioccipital	1	40-50	1
LA3	Clarias sp.	caudal vertebra	1	50-60	1
LA3	Clarias sp.	dentary	1	50-60	1
LA3	Clarias sp.	cranial roof fragment	1	60-70	1
LA3	Clarias sp.	opercular	1	30-40	1
LA3	Clarias sp.	opercular	1	40-50	1
LA3	Clarias sp.	precaudal vertebra	1	60-70	1
LA3	Clarias sp.	pectoral spine	1	60-70	1
LA3	Clarias sp.	caudal vertebra	3	60-70	3
LA3	Clarias sp.	cranial roof fragment	3		
LA3	Haplotilapiini indet.	caudal vertebra	1	20-25	1
LA3	Haplotilapiini indet.	opercular	1	20-25	1
LA3	Haplotilapiini indet.	dorsal or anal pterygophore	1	20-25	1
LA3	Haplotilapiini indet.	cleithrum	2		
LA3	Haplotilapiini indet.	lepidotrich	2		
LA3	Clarias sp.	articular	1	50-60	1
LA3	Clarias sp.	articular	1	50-60	1
LA3	Clarias sp.	caudal vertebra	1	50-60	1
LA3	Clarias sp.	ceratohyale	1	70-80	1
LA3	Clarias sp.	coracoid	1	60-70	1

cultural phase	taxon	skeletal element	NISP	SL in cm	number
LA3	Clarias sp.	coracoid	1		
LA3	Clarias sp.	dentary	1	50-60	1
LA3	Clarias sp.	dentary	1	60-70	1
LA3	Clarias sp.	hyomandibula	1	60-70	1
LA3	Clarias sp.	mesethmoid	1	40-50	1
LA3	Clarias sp.	mesethmoid	1	60-70	1
LA3	Clarias sp.	precaudal vertebra	1	70-80	1
LA3	Clarias sp.	quadrate	1	60-70	1
LA3	Clarias sp.	vomerine toothplate	1	50-60	1
LA3	Clarias sp.	vomerine toothplate	1	60-70	1
LA3	Clarias sp.	cranial roof fragment	16		
LA3	Clarias sp.	cranial roof fragment	5	40-50	5
LA3	Clarias sp.	precaudal vertebra	5	60-70	5
LA3	Clarias sp.	caudal vertebra	6	60-70	6
LA3	Clarias sp.	cranial roof fragment	6	60-70	6
LA3	Haplotilapiini indet.	precaudal vertebra	1	20-25	1
LA3	Haplotilapiini indet.	dorsal or anal pterygophore	1		
LA3	Clarias sp.	articular	1	50-60	1
LA3	Clarias sp.	articular	1	50-60	1
LA3	Clarias sp.	articular	1	60-70	1
LA3	Clarias sp.	articular	1	40-50	1
LA3	Clarias sp.	articular	1	60-70	1
LA3	Clarias sp.	basioccipital	1	60-70	1
LA3	Clarias sp.	ceratohyale	1	50-60	1
LA3	Clarias sp.	ceratohyale	1	50-60	1
LA3	Clarias sp.	ceratohyale	1	60-70	1
LA3	Clarias sp.	cleithrum	1	60-70	1
LA3	Clarias sp.	cleithrum	1	40-50	1
LA3	Clarias sp.	cleithrum	1	60-70	1
LA3	Clarias sp.	cleithrum	1	60-70	1
LA3	Clarias sp.	cleithrum	1	60-70	1
LA3	Clarias sp.	coracoid	1	50-60	1
LA3	Clarias sp.	coracoid	1	60-70	1
LA3	Clarias sp.	coracoid	1		
LA3	Clarias sp.	epihyale	1	90-100	1
LA3	Clarias sp.	cranial roof fragment	1	30-40	1
LA3	Clarias sp.	cranial roof fragment	1	70-80	1
LA3	Clarias sp.	cranial roof fragment	1	50-60	1
LA3	Clarias sp.	hyomandibula	1	40-50	1
LA3	Clarias sp.	hyomandibula	1	50-60	1
LA3	Clarias sp.	mesethmoid	1	50-60	1
LA3	Clarias sp.	opercular	1	40-50	1
LA3	Clarias sp.	opercular	1	40-50	1
LA3	Clarias sp.	opercular	1	40-50	1
LA3	Clarias sp.	pectoral spine	1	60-70	1
LA3	Clarias sp.	pectoral spine	1	60-70	1
LA3	Clarias sp.	quadrate	1	50-60	1
LA3	Clarias sp.	quadrate	1	40-50	1
LA3	Clarias sp.	urohyale	1	60-70	1
LA3	Clarias sp.	vomerine toothplate	1	60-70	1
LA3	Clarias sp.	caudal vertebra	17	60-70	17
LA3	Clarias sp.	cleithrum	2		
LA3	Clarias sp.	palatinum	2	50-60	2
LA3	Clarias sp.	premaxilla	2	60-70	2
LA3	Clarias sp.	caudal vertebra	3	50-60	3
LA3	Clarias sp.	cranial roof fragment	3	50-60	3
LA3	Clarias sp.	precaudal vertebra	3	50-60	3
LA3	Clarias sp.	precaudal vertebra	3	70-80	3

cultural phase	taxon	skeletal element	NISP	SL in cm	number
LA3	Clarias sp.	palatinum	4	60-70	4
LA3	Clarias sp.	precaudal vertebra	5	60-70	5
LA3	Clarias sp.	cranial roof fragment	6	60-70	6
LA3	Clarias sp.	caudal vertebra	8	70-80	8
LA3	Clarias sp.	cranial roof fragment	9		
LA3	Haplotilapiini indet.	opercular	1	15-20	1
LA3	Haplotilapiini indet.	subopercular	1	20-25	1
LA3	Clarias sp.	articular	1	40-50	1
LA3	Clarias sp.	articular	1	60-70	1
LA3	Clarias sp.	articular	1	50-60	1
LA3	Clarias sp.	caudal vertebra	1	50-60	1
LA3	Clarias sp.	ceratohyale	1	60-70	1
LA3	Clarias sp.	ceratohyale	1	60-70	1
LA3	Clarias sp.	cleithrum	1	70-80	1
LA3	Clarias sp.	epihyale	1	50-60	1
LA3	Clarias sp.	mesethmoid	1	50-60	1
LA3	Clarias sp.	mesethmoid	1	60-70	1
LA3	Clarias sp.	opercular	1	50-60	1
LA3	Clarias sp.	opercular	1	40-50	1
LA3	Clarias sp.	precaudal vertebra	1	50-60	1
LA3	Clarias sp.	precaudal vertebra	1	60-70	1
LA3	Clarias sp.	ceratohyale	2	60-70	2
LA3	Clarias sp.	cranial roof fragment	2	50-60	2
LA3	Clarias sp.	cranial roof fragment	2		
LA3	Clarias sp.	cranial roof fragment	3	60-70	3
LA3	Clarias sp.	caudal vertebra	8	60-70	8
LA3	Clarias sp.	articular	1	60-70	1
LA3	Clarias sp.	ceratohyale	1	40-50	1
LA3	Clarias sp.	cleithrum	1	30-40	1
LA3	Clarias sp.	cleithrum	1		
LA3	Clarias sp.	cleithrum	1		
LA3	Clarias sp.	coracoid	1	40-50	1
LA3	Clarias sp.	coracoid	1	50-60	1
LA3	Clarias sp.	dentary	1	50-60	1
LA3	Clarias sp.	dentary	1	50-60	1
LA3	Clarias sp.	epihyale	1	70-80	1
LA3	Clarias sp.	cranial roof fragment	1	30-40	1
LA3	Clarias sp.	hyomandibula	1		
LA3	Clarias sp.	hyomandibula	1	60-70	1
LA3	Clarias sp.	hyomandibula	1	50-60	1
LA3	Clarias sp.	opercular	1		
LA3	Clarias sp.	palatinum	1	60-70	1
LA3	Clarias sp.	pectoral spine	1	70-80	1
LA3	Clarias sp.	premaxilla	1	70-80	1
LA3	Clarias sp.	quadrate	1	40-50	1
LA3	Clarias sp.	quadrate	1	50-60	1
LA3	Clarias sp.	urohyale	1	70-80	1
LA3	Clarias sp.	precaudal vertebra	2	60-70	2
LA3	Clarias sp.	precaudal vertebra	2	60-70	2
LA3	Clarias sp.	caudal vertebra	3	50-60	3
LA3	Clarias sp.	coracoid	3	60-70	3
LA3	Clarias sp.	cranial roof fragment	3	50-60	3
LA3	Clarias sp.	cranial roof fragment	4	50-60	4
LA3	Clarias sp.	caudal vertebra	5	60-70	5
LA3	Clarias sp.	cranial roof fragment	5		
LA3	Clarias sp.	cranial roof fragment	5	60-70	5
LA3	Clarias sp.	cranial roof fragment	6		
LA3	Clarias sp.	caudal vertebra	7	60-70	7

cultural phase	taxon	skeletal element	NISP	SL in cm	number
LA3	Haplotilapiini indet.	skull roof fragment	1		
LA3	O. niloticus	opercular	1	20-25	1
LA3	Haplotilapiini indet.	preopercular	1	25-30	1
LA3	Clarias sp.	articular	1	60-70	1
LA3	Clarias sp.	articular	1	60-70	1
LA3	Clarias sp.	articular	1	50-60	1
LA3	Clarias sp.	caudal vertebra	1	50-60	1
LA3	Clarias sp.	ceratohyale	1	60-70	1
LA3	Clarias sp.	ceratohyale	1	40-50	1
LA3	Clarias sp.	ceratohyale	1	70-80	1
LA3	Clarias sp.	cleithrum	1	50-60	1
LA3	Clarias sp.	cleithrum	1	50-60	1
LA3	Clarias sp.	cleithrum	1	40-50	1
LA3	Clarias sp.	coracoid	1	30-40	1
LA3	Clarias sp.	costa	1		
LA3	Clarias sp.	dentary	1	50-60	1
LA3	Clarias sp.	epihyale	1	70-80	1
LA3	Clarias sp.	cranial roof fragment	1	40-50	1
LA3	Clarias sp.	cranial roof fragment	1	50-60	1
LA3	Clarias sp.	mesethmoid	1	60-70	1
LA3	Clarias sp.	opercular	1	60-70	1
LA3	Clarias sp.	opercular	1	50-60	1
LA3	Clarias sp.	precaudal vertebra	1	60-70	1
LA3	Clarias sp.	pectoral spine	1	70-80	1
LA3	Clarias sp.	quadrate	1	50-60	1
LA3	Clarias sp.	quadrate	1	30-40	1
LA3	Clarias sp.	quadrate	1	50-60	1
LA3	Clarias sp.	quadrate	1	50-60	1
LA3	Clarias sp.	cleithrum	2		
LA3	Clarias sp.	caudal vertebra	3	60-70	3
LA3	Clarias sp.	cranial roof fragment	3	60-70	3
LA3	Clarias sp.	precaudal vertebra	4	60-70	4
LA3	Clarias sp.	caudal vertebra	5	60-70	5
LA3	Clarias sp.	cranial roof fragment	5		
LA3	Clarias sp.	cranial roof fragment	7		
LA3	Haplotilapiini indet.	skull roof fragment	1		
LA3	Haplotilapiini indet.	skull roof fragment	1		
LA3	O. niloticus	hyomandibula	1	20-25	1
LA3	Haplotilapiini indet.	opercular	1		
LA3	Haplotilapiini indet.	preopercular	1	20-25	1
LA3	Haplotilapiini indet.	urohyale	1	20-25	1
LA3	Haplotilapiini indet.	lepidotrich	2		
LA3	Clarias sp.	articular	1	50-60	1
LA3	Clarias sp.	articular	1	50-60	1
LA3	Clarias sp.	articular	1	50-60	1
LA3	Clarias sp.	articular	1	80-90	1
LA3	Clarias sp.	basioccipital	1	50-60	1
LA3	Clarias sp.	basioccipital	1	80-90	1
LA3	Clarias sp.	caudal vertebra	1	50-60	1
LA3	Clarias sp.	ceratohyale	1	70-80	1
LA3	Clarias sp.	cleithrum	1		
LA3	Clarias sp.	coracoid	1	60-70	1
LA3	Clarias sp.	coracoid	1		
LA3	Clarias sp.	cranial roof fragment	1	30-40	1
LA3	Clarias sp.	cranial roof fragment	1	60-70	1
LA3	Clarias sp.	cranial roof fragment	1	30-40	1
LA3	Clarias sp.	cranial roof fragment	1	40-50	1
LA3	Clarias sp.	cranial roof fragment	1	70-80	1

cultural phase	taxon	skeletal element	NISP	SL in cm	number
LA3	Clarias sp.	hyomandibula	1	40-50	1
LA3	Clarias sp.	hyomandibula	1	40-50	1
LA3	Clarias sp.	opercular	1	30-40	1
LA3	Clarias sp.	opercular	1	60-70	1
LA3	Clarias sp.	precaudal vertebra	1	60-70	1
LA3	Clarias sp.	pectoral spine	1	30-40	1
LA3	Clarias sp.	basipterygium	1	60-70	1
LA3	Clarias sp.	premaxilla	1	60-70	1
LA3	Clarias sp.	premaxilla	1	100-110	1
LA3	Clarias sp.	urohyale	1	50-60	1
LA3	Clarias sp.	vomerine toothplate	1	60-70	1
LA3	Clarias sp.	cranial roof fragment	15		
LA3	Clarias sp.	caudal vertebra	2	60-70	2
LA3	Clarias sp.	cranial roof fragment	2	50-60	2
LA3	Clarias sp.	precaudal vertebra	3	60-70	3
LA3	Clarias sp.	cranial roof fragment	6	60-70	6
LA3	Clarias sp.	cranial roof fragment	7		
LA3	Haplotilapiini indet.	cleithrum	1	25-30	1
LA3	Haplotilapiini indet.	skull roof fragment	1	25-30	1
LA3	Haplotilapiini indet.	skull roof fragment	1		
LA3	Haplotilapiini indet.	skull roof fragment	1		
LA3	Haplotilapiini indet.	opercular	1		
LA3	Clarias sp.	articular	1	60-70	1
LA3	Clarias sp.	articular	1	50-60	1
LA3	Clarias sp.	caudal vertebra	1	50-60	1
LA3	Clarias sp.	ceratohyale	1	60-70	1
LA3	Clarias sp.	cleithrum	1	60-70	1
LA3	Clarias sp.	cleithrum	1		
LA3	Clarias sp.	cleithrum	1	50-60	1
LA3	Clarias sp.	cleithrum	1	50-60	1
LA3	Clarias sp.	cleithrum	1		
LA3	Clarias sp.	coracoid	1		
LA3	Clarias sp.	dentary	1	40-50	1
LA3	Clarias sp.	dentary	1	50-60	1
LA3	Clarias sp.	epihyale	1	50-60	1
LA3	Clarias sp.	cranial roof fragment	1	60-70	1
LA3	Clarias sp.	hyomandibula	1		
LA3	Clarias sp.	hyomandibula	1	50-60	1
LA3	Clarias sp.	opercular	1	50-60	1
LA3	Clarias sp.	opercular	1	40-50	1
LA3	Clarias sp.	premaxilla	1	50-60	1
LA3	Clarias sp.	quadrate	1	50-60	1
LA3	Clarias sp.	quadrate	1	40-50	1
LA3	Clarias sp.	cranial roof fragment	11		
LA3	Clarias sp.	caudal vertebra	2	60-70	2
LA3	Clarias sp.	unidentified	2		
LA3	Clarias sp.	precaudal vertebra	2	50-60	2
LA3	Clarias sp.	caudal vertebra	3	50-60	3
LA3	Clarias sp.	cranial roof fragment	3	40-50	3
LA3	Clarias sp.	cranial roof fragment	3	50-60	3
LA3	Clarias sp.	precaudal vertebra	3	60-70	3
LA3	Clarias sp.	caudal vertebra	4	60-70	4
LA3	Clarias sp.	precaudal vertebra	4	60-70	4
LA3	Clarias sp.	cranial roof fragment	7		
LA3	Haplotilapiini indet.	basipterygium	1	15-20	1
LA3	Haplotilapiini indet.	mesethmoid	1	25-30	1
LA3	Haplotilapiini indet.	precaudal vertebra	1	20-25	1
LA3	Haplotilapiini indet.	cleithrum	3		

cultural phase	taxon	skeletal element	NISP	SL in cm	number
LA3	Clarias sp.	hyomandibula	1	50-60	1
LA3	Clarias sp.	pectoral spine	1	60-70	1
LA3	Clarias sp.	cranial roof fragment	4		
LA3	Clarias sp.	caudal vertebra	1	60-70	1
LA3	Clarias sp.	cleithrum	1	40-50	1
LA3	Clarias sp.	cranial roof fragment	1	50-60	1
LA3	Clarias sp.	cranial roof fragment	1	60-70	1
LA3	Clarias sp.	cranial roof fragment	7		
LA3	Clarias sp.	basioccipital	1	60-70	1
LA3	Clarias sp.	cranial roof fragment	1		
LA3	Clarias sp.	cranial roof fragment	2	70-80	2
LA3	Clarias sp.	cranial roof fragment	1		
LA3	Clarias sp.	caudal vertebra	1	50-60	1
LA3	Clarias sp.	cleithrum	1	40-50	1
LA3	Clarias sp.	cleithrum	1		
LA3	Clarias sp.	cranial roof fragment	1	60-70	1
LA3	Clarias sp.	hyomandibula	1	50-60	1
LA3	Clarias sp.	caudal vertebra	2	60-70	2
LA3	Clarias sp.	cranial roof fragment	3		
LA3	Clarias sp.	ceratohyale	1	60-70	1
LA3	Clarias sp.	dentary	1	50-60	1
LA3	Clarias sp.	dentary	1	50-60	1
LA3	Clarias sp.	cranial roof fragment	1		
LA3	Clarias sp.	precaudal vertebra	1	50-60	1
LA3	Clarias sp.	quadrate	1	30-40	1
LA3	Clarias sp.	cranial roof fragment	2	40-50	2
LA3	Clarias sp.	cranial roof fragment	2	30-40	2
LA3	Haplotilapiini indet.	cleithrum	1		
LA3	Clarias sp.	pectoral spine	1	50-60	1
LA3	Clarias sp.	premaxilla	1	60-70	1
LA3	Clarias sp.	cranial roof fragment	3	60-70	3
LA3	Haplotilapiini indet.	dentary	1	25-30	1
LA3	Clarias sp.	articular	1	40-50	1
LA3	Clarias sp.	caudal vertebra	1	60-70	1
LA3	Clarias sp.	cranial roof fragment	1	30-40	1
LA3	Clarias sp.	precaudal vertebra	1	60-70	1
LA3	Clarias sp.	cranial roof fragment	3		
LA3	Clarias sp.	caudal vertebra	1		
LA3	Clarias sp.	ceratohyale	1	70-80	1
LA3	Clarias sp.	coracoid	1		
LA3	Clarias sp.	mesethmoid	1	60-70	1
LA3	Clarias sp.	pectoral spine	1		
LA3	Clarias sp.	quadrate	1	60-70	1
LA3	Clarias sp.	cranial roof fragment	16		
LA3	Clarias sp.	articular	1	50-60	1
LA3	Clarias sp.	cranial roof fragment	1	60-70	1
LA3	Clarias sp.	pectoral spine	1	60-70	1
LA3	Clarias sp.	cranial roof fragment	5		
LA3	Clarias sp.	cleithrum	1		
LA3	Clarias sp.	cranial roof fragment	1	60-70	1
LA3	Clarias sp.	cranial roof fragment	9		
LA3	Clarias sp.	articular	1	60-70	1
LA3	Clarias sp.	caudal vertebra	1	50-60	1
LA3	Clarias sp.	cleithrum	1	70-80	1
LA3	Clarias sp.	coracoid	1		
LA3	Clarias sp.	dentary	1		
LA3	Clarias sp.	opercular	1	50-60	1
LA3	Clarias sp.	opercular	1	40-50	1

cultural phase	taxon	skeletal element	NISP	SL in cm	number
LA3	Clarias sp.	cranial roof fragment	9		
LA3	Haplotilapiini indet.	caudal vertebra	1	20-25	1
LA3	Haplotilapiini indet.	fin spine	1		
LA3	Haplotilapiini indet.	precaudal vertebra	1	20-25	1
LA3	Clarias sp.	caudal vertebra	1	60-70	1
LA3	Clarias sp.	ceratohyale	1	50-60	1
LA3	Clarias sp.	cranial roof fragment	1	70-80	1
LA3	Clarias sp.	branchial element	1		
LA3	Clarias sp.	precaudal vertebra	1	60-70	1
LA3	Clarias sp.	pectoral spine	1	40-50	1
LA3	Clarias sp.	cleithrum	2		
LA3	Clarias sp.	cranial roof fragment	9		
LA3	Haplotilapiini indet.	cleithrum	1		
LA3	Haplotilapiini indet.	skull roof fragment	1		
LA3	Haplotilapiini indet.	fin spine	1		
LA3	Haplotilapiini indet.	precaudal vertebra	1	15-20	1
LA3	Haplotilapiini indet.	dorsal or anal pterygophore	1	15-20	1
LA3	Haplotilapiini indet.	dorsal or anal pterygophore	1	25-30	1
LA3	Haplotilapiini indet.	dorsal or anal pterygophore	1	20-25	1
LA3	Clarias sp.	caudal vertebra	1	40-50	1
LA3	Clarias sp.	caudal vertebra	1	50-60	1
LA3	Clarias sp.	cleithrum	1	40-50	1
LA3	Clarias sp.	cleithrum	1	50-60	1
LA3	Clarias sp.	cranial roof fragment	1	60-70	1
LA3	Clarias sp.	quadrate	1	70-80	1
LA3	Clarias sp.	urophore	1		
LA3	Clarias sp.	cranial roof fragment	2	40-50	2
LA3	Clarias sp.	cranial roof fragment	5		
LA3	Haplotilapiini indet.	cleithrum	1		
LA3	Haplotilapiini indet.	opercular	1	20-25	1
LA3	Haplotilapiini indet.	supracleithrum	1	20-25	1
LA3	Haplotilapiini indet.	lepidotrich	2		
LA3	Clarias sp.	caudal vertebra	1	50-60	1
LA3	Clarias sp.	caudal vertebra	1	60-70	1
LA3	Clarias sp.	cranial roof fragment	1	50-60	1
LA3	Clarias sp.	cranial roof fragment	1	60-70	1
LA3	Clarias sp.	cranial roof fragment	4		
LA3	Haplotilapiini indet.	cleithrum	1		
LA3	Clarias sp.	cleithrum	1		
LA3	Clarias sp.	cranial roof fragment	1		
LA3	Clarias sp.	cranial roof fragment	2	50-60	2
LA3	Clarias sp.	cranial roof fragment	3		
LA3	Haplotilapiini indet.	suborbital	1	20-25	1
LA3	Clarias sp.	parasphenoid	1	100-110	1
LA3	Clarias sp.	epihyale	1	70-80	1
LA3	Clarias sp.	pectoral spine	1	40-50	1
LA3	Clarias sp.	quadrate	1	70-80	1
LA3	Clarias sp.	cranial roof fragment	2	70-80	1
LA3	Clarias sp.	cranial roof fragment	4		
LA3	Haplotilapiini indet.	opercular	1	20-25	1
LA3	Clarias sp.	cleithrum	1	40-50	1
LA3	Clarias sp.	cleithrum	1	50-60	1
LA3	Clarias sp.	cranial roof fragment	1	70-80	1
LA3	Clarias sp.	quadrate	1	30-40	1
LA3	Clarias sp.	cranial roof fragment	2		
LA3	Clarias sp.	articular	1	50-60	1
LA3	Clarias sp.	hyomandibula	1		
LA3	Clarias sp.	precaudal vertebra	1		

cultural phase	taxon	skeletal element	NISP	SL in cm	number
LA3	Clarias sp.	cleithrum	1		
LA3	Clarias sp.	dentary	1	50-60	1
LA3	Clarias sp.	cranial roof fragment	1	40-50	1
LA3	Clarias sp.	cranial roof fragment	1		
LA3	Clarias sp.	cranial roof fragment	1		
LA3	Clarias sp.	quadrate	1	50-60	1
LA3	Haplotilapiini indet.	unidentified	1		
LA3	Haplotilapiini indet.	skull roof fragment	1		
LA3	Haplotilapiini indet.	cleithrum	2		
LA3	Clarias sp.	coracoid	1		
LA3	Clarias sp.	cranial roof fragment	1	70-80	1
LA3	Clarias sp.	opercular	1	70-80	1
LA3	Clarias sp.	pectoral spine	1	40-50	1
LA3	Clarias sp.	cranial roof fragment	5		
LA3	Haplotilapiini indet.	caudal vertebra	1	15-20	1
LA3	Haplotilapiini indet.	caudal vertebra	1	20-25	1
LA3	Clarias sp.	cleithrum	1	30-40	1
LA3	Clarias sp.	cleithrum	1		
LA3	Clarias sp.	cranial roof fragment	4		
LA3	Haplotilapiini indet.	cleithrum	1		
LA3	Haplotilapiini indet.	precaudal vertebra	2	15-20	1
LA3	Clarias sp.	fin ray	1		
LA3	Clarias sp.	cranial roof fragment	1	30-40	1
LA3	Clarias sp.	cranial roof fragment	1	40-50	1
LA3	Clarias sp.	cranial roof fragment	1		
LA3	Clarias sp.	precaudal vertebra	1	15-20	1
LA3	Clarias sp.	caudal vertebra	3	60-70	1
LA3	Clarias sp.	cranial roof fragment	2		
LA3	Clarias sp.	cleithrum	1	30-40	1
LA3	Clarias sp.	epi- & ceratohyale	1	60-70	1
LA3	Clarias sp.	cranial roof fragment	2		
LA3	Haplotilapiini indet.	opercular	1	15-20	1
LA3	Clarias sp.	cleithrum	1	40-50	1
LA3	Haplotilapiini indet.	cleithrum	1		
LA3	Haplotilapiini indet.	opercular	1		
LA3	Clarias sp.	coracoid	1	40-50	1
LA3	Clarias sp.	coracoid	1		
LA3	Clarias sp.	cranial roof fragment	1		
LA3	Clarias sp.	pectoral spine	1		
LA3	Clarias sp.	caudal vertebra	2	60-70	1
LA3	Clarias sp.	caudal vertebra	1	15-20	1
LA3	Haplotilapiini indet.	cleithrum	1	15-20	1
LA3	Haplotilapiini indet.	neurocranium fragment	1	15-20	1
LA3	Haplotilapiini indet.	precaudal vertebra	1	15-20	1
LA3	Haplotilapiini indet.	precaudal vertebra	1	15-20	1
LA3	Haplotilapiini indet.	dorsal or anal pterygophore	1		
LA3	Haplotilapiini indet.	sop	1	20-25	1
LA3	Clarias sp.	cranial roof fragment	2		
LA3	Haplotilapiini indet.	caudal vertebra	1		
LA3	Haplotilapiini indet.	caudal vertebra	2	20-25	1
LA3	Haplotilapiini indet.	dorsal or anal pterygophore	2		
LA3	Haplotilapiini indet.	caudal vertebra	8	15-20	1
LA3	Haplotilapiini indet.	cleithrum	1		
LA3	Haplotilapiini indet.	precaudal vertebra	1	15-20	1
LA3	Haplotilapiini indet.	precaudal vertebra	1	20-25	1
LA3	Haplotilapiini indet.	scapula	1	20-25	1
LA3	Clarias sp.	pectoral spine	1	60-70	1
LA3	Clarias sp.	cranial roof fragment	5		

cultural phase	taxon	skeletal element	NISP	SL in cm	number
LA3	Clarias sp.	cranial roof fragment	1		
LA3	Clarias sp.	cranial roof fragment	1		
LA3	Clarias sp.	pectoral spine	1	70-80	1
LA3	Clarias sp.	cranial roof fragment	2		
LA3	C. gariepinus	vomerine toothplate	1	70-80	1
LA3	Clarias sp.	cranial roof fragment	1		
LA3	Haplotilapiini indet.	opercular	1	20-25	1
LA3	Clarias sp.	cleithrum	1	50-60	1
LA3	Clarias sp.	coracoid	1	50-60	1
LA3	Clarias sp.	epi- & ceratohyale	1	40-50	1
LA3	Clarias sp.	precaudal vertebra	1	60-70	1
LA3	Clarias sp.	caudal vertebra	2	60-70	1
LA3	Clarias sp.	cranial roof fragment	3		
LA3	Clarias sp.	articular	1	50-60	1
LA3	Clarias sp.	articular	1	40-50	1
LA3	Clarias sp.	caudal vertebra	1	40-50	1
LA3	Clarias sp.	caudal vertebra	1	50-60	1
LA3	Clarias sp.	coracoid	1		
LA3	Clarias sp.	coracoid	1		
LA3	Clarias sp.	dentary	1	40-50	1
LA3	Clarias sp.	cranial roof fragment	1		
LA3	Haplotilapiini indet.	caudal vertebra	1	20-25	1
LA3	Haplotilapiini indet.	cleithrum	1	15-20	1
LA3	Haplotilapiini indet.	unidentified	1		
LA3	C. gariepinus	vomerine toothplate	1	40-50	1
LA3	Clarias sp.	articular	1	50-60	1
LA3	Clarias sp.	articular	1	40-50	1
LA3	Clarias sp.	caudal vertebra	1		
LA3	Clarias sp.	coracoid	1		
LA3	Clarias sp.	mesethmoid	1	40-50	1
LA3	Clarias sp.	cranial roof fragment	1	60-70	1
LA3	Clarias sp.	pectoral spine	1	60-70	1
LA3	Clarias sp.	quadrate	1	50-60	1
LA3	Clarias sp.	urohyale	1	50-60	1
LA3	Clarias sp.	caudal vertebra	2	60-70	1
LA3	Clarias sp.	cranial roof fragment	2	40-50	1
LA3	Clarias sp.	cranial roof fragment	2		
LA3	Clarias sp.	cranial roof fragment	2		
LA3	Clarias sp.	cranial roof fragment	6		
LA3	Haplotilapiini indet.	cleithrum	1	20-25	1
LA3	Haplotilapiini indet.	cleithrum	1		
LA3	Haplotilapiini indet.	coracoid	1	20-25	1
LA3	Haplotilapiini indet.	hyomandibula	1	20-25	1
LA3	Haplotilapiini indet.	opercular	1	20-25	1
LA3	Haplotilapiini indet.	postcleithrum	1	20-25	1
LA3	Haplotilapiini indet.	unidentified	2		
LA3	Haplotilapiini indet.	skull roof fragment	2		
LA3	Clarias sp.	articular	1	50-60	1
LA3	Clarias sp.	caudal vertebra	1	40-50	1
LA3	Clarias sp.	caudal vertebra	1	15-20	1
LA3	Clarias sp.	cleithrum	1	60-70	1
LA3	Clarias sp.	precaudal vertebra	1	70-80	1
LA3	Clarias sp.	caudal vertebra	3	60-70	1
LA3	Clarias sp.	cranial roof fragment	3		
LA3	Clarias sp.	cranial roof fragment	9		
LA3	Clarias sp.	caudal vertebra	1	60-70	1
LA3	Clarias sp.	quadrate	1	60-70	1
LA3	Clarias sp.	cranial roof fragment	4		

cultural phase	taxon	skeletal element	NISP	SL in cm	number
LA3	Clarias sp.	mesethmoid	1	70-80	1
LA3	Clarias sp.	cranial roof fragment	3		
LA3	Clarias sp.	caudal vertebra	1	50-60	1
LA3	Clarias sp.	ceratohyale	1	90-100	1
LA3	Clarias sp.	cleithrum	1		
LA3	Clarias sp.	cranial roof fragment	1	60-70	1
LA3	Clarias sp.	cranial roof fragment	1	90-100	1
LA3	Clarias sp.	cranial roof fragment	1		
LA3	Clarias sp.	precaudal vertebra	1	40-50	1
LA3	Haplotilapiini indet.	caudal vertebra	2	15-20	1
LA3	Clarias sp.	articular	1	50-60	1
LA3	Clarias sp.	caudal vertebra	1	40-50	1
LA3	Clarias sp.	cleithrum	1		
LA3	Clarias sp.	coracoid	1		
LA3	Clarias sp.	coracoid	1	40-50	1
LA3	Clarias sp.	cranial roof fragment	1	40-50	1
LA3	Clarias sp.	cranial roof fragment	1		
LA3	Clarias sp.	pectoral spine	1		
LA3	Haplotilapiini indet.	cleithrum	1	15-20	1
LA3	Haplotilapiini indet.	fin spine	1		
LA3	Haplotilapiini indet.	vertebra fragment	1		
LA3	Clarias sp.	caudal vertebra	1	60-70	1
LA3	Clarias sp.	ceratohyale	1	50-60	1
LA3	Clarias sp.	cleithrum	1	50-60	1
LA3	Clarias sp.	cranial roof fragment	1	50-60	1
LA3	Clarias sp.	cranial roof fragment	1	90-100	1
LA3	Clarias sp.	cranial roof fragment	4		
LA3	Haplotilapiini indet.	opercular	1		
LA3	Clarias sp.	articular	1	30-40	1
LA3	Clarias sp.	cranial roof fragment	1	60-70	1
LA3	O. niloticus	urohyale	1	20-25	1
LA3	Clarias sp.	caudal vertebra	1		
LA3	Clarias sp.	cleithrum	1	60-70	1
LA3	Clarias sp.	cleithrum	1	70-80	1
LA3	Clarias sp.	cleithrum	1	60-70	1
LA3	Clarias sp.	cleithrum	1	60-70	1
LA3	Clarias sp.	opercular	1	30-40	1
LA3	Clarias sp.	cranial roof fragment	2	60-70	1
LA3	Clarias sp.	cranial roof fragment	3		
LA3	Clarias sp.	articular	1	60-70	1
LA3	Clarias sp.	cleithrum	1	50-60	1
LA3	Clarias sp.	dentary	1		
LA3	Clarias sp.	unidentified	1		
LA3	Clarias sp.	cranial roof fragment	1		
LA3	Clarias sp.	caudal vertebra	2	30-40	1
LA3	Clarias sp.	cranial roof fragment	1		
LA3	Clarias sp.	caudal vertebra	1		
LA3	Clarias sp.	cleithrum	1	20-30	1
LA3	Clarias sp.	dentary	1	50-60	1
LA3	Clarias sp.	dentary	1	40-50	1
LA3	Clarias sp.	hyomandibula	1		
LA3	Clarias sp.	cranial roof fragment	1	50-60	1
LA3	Clarias sp.	opercular	1	70-80	1
LA3	Clarias sp.	caudal vertebra	2	50-60	1
LA3	Clarias sp.	cranial roof fragment	2		
LA3	Clarias sp.	cranial roof fragment	2		
LA3	Clarias sp.	caudal vertebra	3	40-50	1
LA3	Clarias sp.	cranial roof fragment	5		

cultural phase	taxon	skeletal element	NISP	SL in cm	number
LA3	Haplotilapiini indet.	anal pterygophore	1	20-25	1
LA3	Haplotilapiini indet.	anal pterygophore	1	20-25	1
LA3	Haplotilapiini indet.	anal pterygophore	1	15-20	1
LA3	Haplotilapiini indet.	anal pterygophore	1	15-20	1
LA3	Haplotilapiini indet.	anal pterygophore	1	15-20	1
LA3	Haplotilapiini indet.	anal pterygophore	1	15-20	1
LA3	Haplotilapiini indet.	anal pterygophore	1	15-20	1
LA3	Haplotilapiini indet.	anal pterygophore	1	15-20	1
LA3	Haplotilapiini indet.	anal pterygophore	1	15-20	1
LA3	Haplotilapiini indet.	anal pterygophore	1	20-25	1
LA3	Haplotilapiini indet.	basioccipital	1	20-25	1
LA3	Haplotilapiini indet.	epi- & ceratohyale	1		
LA3	O. niloticus	hyomandibula	1	20-25	1
LA3	O. niloticus	hyomandibula	1	20-25	1
LA3	C. zillii	hyomandibula	1	20-25	1
LA3	Haplotilapiini indet.	hyomandibula	1	25-30	1
LA3	Haplotilapiini indet.	branchial element	1		
LA3	C. zillii	maxilla	1	20-25	1
LA3	C. zillii	mesethmoid	1	15-20	1
LA3	Haplotilapiini indet.	opercular	1	15-20	1
LA3	Haplotilapiini indet.	opercular	1	15-20	1
LA3	Haplotilapiini indet.	opercular	1	15-20	1
LA3	Haplotilapiini indet.	opercular	1	15-20	1
LA3	Haplotilapiini indet.	opercular	1	20-25	1
LA3	Haplotilapiini indet.	opercular	1	20-25	1
LA3	Haplotilapiini indet.	2nd precaudal vertebra	1	20-25	1
LA3	Haplotilapiini indet.	2nd precaudal vertebra	1	20-25	1
LA3	Haplotilapiini indet.	2nd precaudal vertebra	1	15-20	1
LA3	Haplotilapiini indet.	2nd precaudal vertebra	1	20-25	1
LA3	Haplotilapiini indet.	2nd precaudal vertebra	1	20-25	1
LA3	Haplotilapiini indet.	2nd precaudal vertebra	1	15-20	1
LA3	Haplotilapiini indet.	2nd precaudal vertebra	1	20-25	1
LA3	Haplotilapiini indet.	2nd precaudal vertebra	1	15-20	1
LA3	Haplotilapiini indet.	3rd precaudal vertebra	1	20-25	1
LA3	Haplotilapiini indet.	3rd precaudal vertebra	1	20-25	1
LA3	Haplotilapiini indet.	premaxilla	1	20-25	1
LA3	Haplotilapiini indet.	posttemporal	1	20-25	1
LA3	Haplotilapiini indet.	supracleithrum	1	20-25	1
LA3	Haplotilapiini indet.	fin spine	10		
LA3	Haplotilapiini indet.	cleithrum	16		
LA3	Haplotilapiini indet.	skull roof fragment	18		
LA3	Haplotilapiini indet.	dorsal or anal pterygophore	2		
LA3	Haplotilapiini indet.	supracleithrum	2	15-20	1
LA3	Haplotilapiini indet.	urohyale	2	15-20	1
LA3	Haplotilapiini indet.	caudal vertebra	3	20-25	1
LA3	Haplotilapiini indet.	caudal vertebra	30	15-20	1
LA3	Haplotilapiini indet.	precaudal vertebra	30	15-20	1
LA3	Haplotilapiini indet.	cleithrum	4	20-25	1
LA3	Haplotilapiini indet.	preopercular	5		
LA3	Haplotilapiini indet.	precaudal vertebra	9	20-25	1
LA3	C. gariepinus	vomerine toothplate	1	30-40	1
LA3	Clarias sp.	ceratohyale	1	50-60	1
LA3	Clarias sp.	cranial roof fragment	1		
LA3	Clarias sp.	cleithrum	1	40-50	1
LA3	Clarias sp.	coracoid	1		
LA3	Clarias sp.	cranial roof fragment	1	40-50	1
LA3	Clarias sp.	cranial roof fragment	3		
LA3	Clarias sp.	coracoid	1		

cultural phase	taxon	skeletal element	NISP	SL in cm	number
LA3	Clarias sp.	dentary	1	40-50	1
LA3	Clarias sp.	dentary	1	60-70	1
LA3	Clarias sp.	cranial roof fragment	1	30-40	1
LA3	Clarias sp.	cranial roof fragment	1		
LA3	Clarias sp.	cranial roof fragment	1		
LA3	Clarias sp.	opercular	1	15-20	1
LA3	Clarias sp.	pterygophore	1		
LA3	Clarias sp.	quadrate	1	30-40	1
LA3	Clarias sp.	cranial roof fragment	7		
LA3	Haplotilapiini indet.	unidentified	1		
LA3	Clarias sp.	caudal vertebra	1	50-60	1
LA3	Clarias sp.	caudal vertebra	1	60-70	1
LA3	Clarias sp.	cleithrum	1	30-35	1
LA3	Clarias sp.	cleithrum	1	20-30	1
LA3	Clarias sp.	epi- & ceratohyale	1	50-60	1
LA3	Clarias sp.	hyomandibula	1		
LA3	Clarias sp.	cranial roof fragment	1	50-60	1
LA3	Clarias sp.	opercular	1		
LA3	Clarias sp.	quadrate	1	30-40	1
LA3	Clarias sp.	quadrate	1	50-60	1
LA3	Clarias sp.	urohyale	1	40-50	1
LA3	Clarias sp.	cranial roof fragment	10		
LA3	Clarias sp.	articular & dentary	1	50-60	1
LA3	Clarias sp.	cleithrum	1	30-40	1
LA3	Clarias sp.	cleithrum	1		
LA3	Clarias sp.	mesethmoid	1	50-60	1
LA3	Clarias sp.	pectoral spine	1	30-40	1
LA3	Clarias sp.	caudal vertebra	2	60-70	1
LA3	Clarias sp.	cranial roof fragment	2		
LA3	Haplotilapiini indet.	anal pterygophore	1	20-25	1
LA3	Haplotilapiini indet.	anal pterygophore	1	20-25	1
LA3	Haplotilapiini indet.	dentary	1	20-25	1
LA3	Haplotilapiini indet.	fin spine	1		
LA3	Haplotilapiini indet.	skull roof fragment	1		
LA3	Clarias sp.	articular & dentary	1	40-50	1
LA3	Clarias sp.	cleithrum	1	30-40	1
LA3	Clarias sp.	cleithrum	1	20-30	1
LA3	Clarias sp.	cleithrum	1		
LA3	Clarias sp.	coracoid	1		
LA3	Clarias sp.	mesethmoid	1	30-40	1
LA3	Clarias sp.	precaudal vertebra	1	60-70	1
LA3	Clarias sp.	pectoral spine	1	60-70	1
LA3	Clarias sp.	cranial roof fragment	4		
LA3	Haplotilapiini indet.	fin spine	1		
LA3	Haplotilapiini indet.	cleithrum	2		
LA3	Haplotilapiini indet.	skull roof fragment	2		
LA3	C. gariepinus	vomerine toothplate	1		
LA3	Clarias sp.	coracoid	1		
LA3	Clarias sp.	mesethmoid	1	60-70	1
LA3	Clarias sp.	cranial roof fragment	1	70-80	1
LA3	Clarias sp.	pectoral spine	1	50-60	1
LA3	Clarias sp.	pectoral spine	1	60-70	1
LA3	Clarias sp.	cranial roof fragment	6		
LA3	Clarias sp.	cleithrum	1	50-60	1
LA3	Clarias sp.	cranial roof fragment	1		
LA3	Clarias sp.	cranial roof fragment	1		
LA3	Clarias sp.	cranial roof fragment	1		
LA3	Clarias sp.	pectoral spine	1	40-50	1

cultural phase	taxon	skeletal element	NISP	SL in cm	number
LA3	Haplotilapiini indet.	cleithrum	1	20-25	1
LA3	Clarias sp.	caudal vertebra	1	60-70	1
LA3	Clarias sp.	cleithrum	1		
LA3	Clarias sp.	cleithrum	1	40-50	1
LA3	Clarias sp.	opercular	1	60-70	1
LA3	Clarias sp.	cranial roof fragment	7		
LA3	Haplotilapiini indet.	anal pterygophore	1	15-20	1
LA3	Haplotilapiini indet.	cleithrum	1	25-30	1
LA3	O. niloticus	hyomandibula	1	20-25	1
LA3	Haplotilapiini indet.	precaudal vertebra	1	20-25	1
LA3	Haplotilapiini indet.	postcleithrum	1	25-30	1
LA3	Clarias sp.	articular & dentary	1	60-70	1
LA3	Clarias sp.	coracoid	1		
LA3	Clarias sp.	epi- & ceratohyale	1	40-50	1
LA3	Clarias sp.	cranial roof fragment	1		
LA3	Clarias sp.	precaudal vertebra	1	30-40	1
LA3	Clarias sp.	unidentified	1		
LA3	Clarias sp.	cranial roof fragment	1		
LA3	Clarias sp.	cranial roof fragment	1		
LA3	Clarias sp.	cleithrum	1	40-50	1
LA3	O. niloticus	hyomandibula	1	15-20	1
LA3	Haplotilapiini indet.	subopercular	1	15-20	1
LA3	Clarias sp.	articular	1	30-40	1
LA3	Clarias sp.	cleithrum	1	60-70	1
LA3	Clarias sp.	opercular	1	50-60	1
LA3	Clarias sp.	articular	1	30-40	1
LA3	Clarias sp.	hyomandibula	1	50-60	1
LA3	Clarias sp.	cranial roof fragment	1	50-60	1
LA3	Clarias sp.	pectoral spine	1	60-70	1
LA3	Haplotilapiini indet.	parasphenoid	1	20-25	1
LA3	C. gariepinus	vomerine toothplate	1		
LA3	Clarias sp.	caudal vertebra	1	60-70	1
LA3	Clarias sp.	caudal vertebra	1	60-70	1
LA3	Clarias sp.	cleithrum	1	30-40	1
LA3	Clarias sp.	cleithrum	1	50-60	1
LA3	Clarias sp.	cranial roof fragment	1	30-40	1
LA3	Clarias sp.	cranial roof fragment	1	40-50	1
LA3	Clarias sp.	cranial roof fragment	2	50-60	1
LA3	Haplotilapiini indet.	skull roof fragment	1		
LA3	Haplotilapiini indet.	cleithrum	2	20-25	1
LA3	Clarias sp.	articular	1	40-50	1
LA3	Clarias sp.	caudal vertebra	1	50-60	1
LA3	Clarias sp.	caudal vertebra	1	60-70	1
LA3	Clarias sp.	cleithrum	1		
LA3	Clarias sp.	cleithrum	1		
LA3	Clarias sp.	coracoid	1	50-60	1
LA3	Clarias sp.	mesethmoid	1	60-70	1
LA3	Clarias sp.	cranial roof fragment	1	50-60	1
LA3	Clarias sp.	cranial roof fragment	1	60-70	1
LA3	Clarias sp.	precaudal vertebra	1	50-60	1
LA3	Clarias sp.	pectoral spine	1	70-80	1
LA3	Clarias sp.	pectoral spine	1	50-60	1
LA3	Clarias sp.	pectoral spine	1	60-70	1
LA3	Clarias sp.	cranial roof fragment	11		
LA3	Clarias sp.	caudal vertebra	2	50-60	1
LA3	Clarias sp.	cleithrum	2	30-40	1
LA3	Clarias sp.	cranial roof fragment	2	50-60	1
LA3	Clarias sp.	cranial roof fragment	2	70-80	1

cultural phase	taxon	skeletal element	NISP	SL in cm	number
LA3	Clarias sp.	precaudal vertebra	2		
LA3	Clarias sp.	cranial roof fragment	3	30-40	1
LA3	Clarias sp.	cranial roof fragment	5		
LA3	Haplotilapiini indet.	anal pterygophore	1	25-30	1
LA3	Haplotilapiini indet.	basipterygium	1	20-25	1
LA3	Haplotilapiini indet.	opercular	1	25-30	1
LA3	Haplotilapiini indet.	opercular	1		
LA3	Clarias sp.	mesethmoid	1	70-80	1
LA3	Clarias sp.	cranial roof fragment	1		
LA3	Haplotilapiini indet.	cleithrum	1	20-25	1
LA3	C. gariepinus	vomerine toothplate	1		
LA3	Clarias sp.	caudal vertebra	1	50-60	1
LA3	Clarias sp.	hyomandibula	1	50-60	1
LA3	Clarias sp.	cranial roof fragment	1	50-60	1
LA3	Haplotilapiini indet.	cleithrum	1		
LA3	Haplotilapiini indet.	skull roof fragment	1	20-25	1
LA3	Haplotilapiini indet.	precaudal vertebra	1	15-20	1
LA3	Clarias sp.	cranial roof fragment	23	60-70	5
LA3	Clarias sp.	articular	1	50-60	1
LA3	Clarias sp.	articular	1	50-60	1
LA3	Clarias sp.	caudal vertebra	1	50-60	1
LA3	Clarias sp.	caudal vertebra	1	50-60	1
LA3	Clarias sp.	caudal vertebra	1	60-70	1
LA3	Clarias sp.	caudal vertebra	1		
LA3	Clarias sp.	caudal vertebra	1		
LA3	Clarias sp.	caudal vertebra	1	40-50	1
LA3	Clarias sp.	caudal vertebra	1	50-60	1
LA3	Clarias sp.	caudal vertebra	1	60-70	1
LA3	Clarias sp.	caudal vertebra	1	70-80	1
LA3	Clarias sp.	cleithrum	1	70-80	1
LA3	Clarias sp.	cleithrum	1	70-80	1
LA3	Clarias sp.	cleithrum	1	70-80	1
LA3	Clarias sp.	dentary	1	50-60	1
LA3	Clarias sp.	epi- & ceratohyale	1	50-60	1
LA3	Clarias sp.	epi- & ceratohyale	1	60-70	1
LA3	Clarias sp.	branchial element	1		
LA3	Clarias sp.	branchial element	1		
LA3	Clarias sp.	precaudal vertebra	1	40-50	1
LA3	Clarias sp.	precaudal vertebra	1	50-60	1
LA3	Clarias sp.	precaudal vertebra	1	50-60	1
LA3	Clarias sp.	precaudal vertebra	1	90-100	1
LA3	Clarias sp.	pectoral spine	1	60-70	1
LA3	Clarias sp.	pectoral spine	1	60-70	1
LA3	Haplotilapiini indet.	precaudal vertebra	1	10-15	1
LA3	Haplotilapiini indet.	palatinum	2		
LA3	Haplotilapiini indet.	preopercular	2		
LA3	Haplotilapiini indet.	caudal vertebra	24	20-25	1
LA3	Haplotilapiini indet.	precaudal vertebra	27	20-25	1
LA3	Haplotilapiini indet.	skull roof fragment	3		
LA3	Haplotilapiini indet.	caudal vertebra	4	15-20	1
LA3	Haplotilapiini indet.	dorsal or anal pterygophore	4		
LA3	Haplotilapiini indet.	cleithrum	5	20-25	1
LA3	Haplotilapiini indet.	precaudal vertebra	7	15-20	1
LA3	Haplotilapiini indet.	lepidotrich	8		
LA3	Haplotilapiini indet.	basioccipital	1	20-25	1
LA3	Haplotilapiini indet.	basioccipital	1	20-25	1
LA3	Haplotilapiini indet.	basipterygium	1		
LA3	O. niloticus	hyomandibula	1	20-25	1

cultural phase	taxon	skeletal element	NISP	SL in cm	number
LA3	O. niloticus	hyomandibula	1	20-25	1
LA3	O. niloticus	hyomandibula	1	15-20	1
LA3	Haplotilapiini indet.	opercular	1	20-25	1
LA3	Haplotilapiini indet.	opercular	1	20-25	1
LA3	Haplotilapiini indet.	opercular	1	20-25	1
LA3	Haplotilapiini indet.	opercular	1	20-25	1
LA3	Haplotilapiini indet.	opercular	1	20-25	1
LA3	Haplotilapiini indet.	opercular	1	15-20	1
LA3	Haplotilapiini indet.	opercular	1		
LA3	Haplotilapiini indet.	parasphenoid	1	20-25	1
LA3	Haplotilapiini indet.	2nd precaudal vertebra	1	25-30	1
LA3	Haplotilapiini indet.	2nd precaudal vertebra	1	20-25	1
LA3	Haplotilapiini indet.	2nd precaudal vertebra	1	20-25	1
LA3	Haplotilapiini indet.	3rd precaudal vertebra	1	20-25	1
LA3	Haplotilapiini indet.	premaxilla	1	20-25	1
LA3	Clarias sp.	articular & dentary	1	50-60	1
LA3	Clarias sp.	cranial roof fragment	2		
LA3	Clarias sp.	unidentified	1		
LA3	Clarias sp.	parasphenoid	1		
LA3	Clarias sp.	hyomandibula	1		
LA3	Clarias sp.	cranial roof fragment	3		
LA3	Clarias sp.	epi- & ceratohyale	1	50-60	1
LA3	Clarias sp.	cranial roof fragment	2		
LA3	Clarias sp.	articular	1	60-70	1
LA3	Clarias sp.	cleithrum	1	50-60	1
LA3	Clarias sp.	dentary	1	60-70	1
LA3	Clarias sp.	cranial roof fragment	1	40-50	1
LA3	Clarias sp.	cranial roof fragment	1	70-80	1
LA3	Clarias sp.	cranial roof fragment	1		
LA3	Clarias sp.	cranial roof fragment	1	60-70	1
LA3	Clarias sp.	cranial roof fragment	1	70-80	1
LA3	Clarias sp.	precaudal vertebra	1	60-70	1
LA3	Clarias sp.	pectoral spine	1	60-70	1
LA3	Clarias sp.	quadrate	1	60-70	1
LA3	Clarias sp.	cranial roof fragment	5		
LA3	Haplotilapiini indet.	opercular	1		
LA3	Clarias sp.	cranial roof fragment	7		
LA3	Haplotilapiini indet.	precaudal vertebra	1	20-25	1
LA3	Clarias sp.	articular	1		
LA3	Clarias sp.	cleithrum	1		
LA3	Clarias sp.	unidentified	1		
LA3	Clarias sp.	cranial roof fragment	1		
LA3	Clarias sp.	cranial roof fragment	1		
LA3	Clarias sp.	cranial roof fragment	8		
LA3	Haplotilapiini indet.	cleithrum	1	15-20	1
LA3	Haplotilapiini indet.	supracleithrum	2	15-20	1
LA3	Clarias sp.	caudal vertebra	1		
LA3	Clarias sp.	pectoral spine	1		
LA3	Clarias sp.	cranial roof fragment	6		
LA3	Clarias sp.	cranial roof fragment	2		
LA3	Clarias sp.	articular	1	40-50	1
LA3	Clarias sp.	caudal vertebra	1	60-70	1
LA3	Clarias sp.	dentary	1	40-50	1
LA3	Clarias sp.	dentary	1	50-60	1
LA3	Clarias sp.	dentary	1	50-60	1
LA3	Clarias sp.	dentary	1		
LA3	Clarias sp.	branchial element	1		
LA3	Clarias sp.	mesethmoid	1	40-50	1

cultural phase	taxon	skeletal element	NISP	SL in cm	number
LA3	Clarias sp.	cranial roof fragment	1		
LA3	Clarias sp.	precaudal vertebra	1	60-70	1
LA3	Clarias sp.	pectoral spine	1	50-60	1
LA3	Clarias sp.	pectoral spine	1	60-70	1
LA3	Clarias sp.	pectoral spine	1	50-60	1
LA3	Clarias sp.	pectoral spine	1		
LA3	Clarias sp.	urohyale	1	40-50	1
LA3	Clarias sp.	urohyale	1	50-60	1
LA3	Clarias sp.	cleithrum	2		
LA3	Clarias sp.	coracoid	3		
LA3	Clarias sp.	cranial roof fragment	9		
LA3	Haplotilapiini indet.	cleithrum	1		
LA3	Clarias sp.	cranial roof fragment	1		
LA3	Clarias sp.	cleithrum	2	50-70	1
LA3	Clarias sp.	mesethmoid	1	50-60	1
LA3	Clarias sp.	cleithrum	2	40-50	1
LA3	Clarias sp.	cleithrum	2		
LA3	Clarias sp.	coracoid	3		
LA3	Clarias sp.	cranial roof fragment	3		
LA3	Clarias sp.	articular	1	60-70	1
LA3	Clarias sp.	coracoid	1		
LA3	Clarias sp.	cranial roof fragment	1	60-70	1
LA3	Clarias sp.	cranial roof fragment	1		
LA3	Clarias sp.	pectoral spine	1	70-80	1
LA3	Clarias sp.	caudal vertebra	1		
LA3	Clarias sp.	cleithrum	1	40-50	1
LA3	Clarias sp.	epi- & ceratohyale	1	70-80	1
LA3	Clarias sp.	cranial roof fragment	1	40-50	1
LA3	Clarias sp.	cranial roof fragment	1		
LA3	Clarias sp.	pectoral spine	1	60-70	1
LA3	Haplotilapiini indet.	precaudal vertebra	1	20-25	1
LA3	Clarias sp.	cranial roof fragment	16		
LA3	Clarias sp.	cranial roof fragment	2		
LA3	Clarias sp.	hyomandibula	1	50-60	1
LA3	Clarias sp.	cleithrum	1		
LA3	C. gariepinus	vomer	1	60-70	1
LA3	Clarias sp.	cranial roof fragment	1	70-80	1
LA3	Clarias sp.	cranial roof fragment	2	60-70	1
LA3	Clarias sp.	cranial roof fragment	4		
LA3	Clarias sp.	cleithrum	1		
LA3	Clarias sp.	epi- & ceratohyale	1	80-90	1
LA3	Clarias sp.	cranial roof fragment	1	50-60	1
LA3	Clarias sp.	pectoral spine	1	60-70	1
LA3	Clarias sp.	quadrate	1	40-50	1
LA3	Clarias sp.	quadrate	1	50-60	1
LA3	Clarias sp.	cleithrum	2	60-70	1
LA3	Clarias sp.	cranial roof fragment	2	50-60	1
LA3	Clarias sp.	cranial roof fragment	4		
LA3	Haplotilapiini indet.	cleithrum	1	15-20	1
LA3	Haplotilapiini indet.	unidentified	1		
LA3	Clarias sp.	cranial roof fragment	4		
LA3	Clarias sp.	mesethmoid	1	50-60	1
LA3	Clarias sp.	cranial roof fragment	1		
LA3	Clarias sp.	pectoral spine	1	60-70	1
LA3	Clarias sp.	cleithrum	1	70-80	1
LA3	Clarias sp.	cleithrum	1		
LA3	Clarias sp.	hyomandibula	1		
LA3	Clarias sp.	cranial roof fragment	1		

cultural phase	taxon	skeletal element	NISP	SL in cm	number
LA3	Clarias sp.	cranial roof fragment	1		
LA3	Clarias sp.	precaudal vertebra	1	40-50	1
LA3	Clarias sp.	pectoral spine	1	40-50	1
LA3	Clarias sp.	pectoral spine	1	70-80	1
LA3	Clarias sp.	caudal vertebra	2		
LA3	Clarias sp.	cranial roof fragment	2	90-100	1
LA3	Clarias sp.	cranial roof fragment	5		
LA3	Clarias sp.	cranial roof fragment	1		
LA3	Clarias sp.	caudal vertebra	1	70-80	1
LA3	Clarias sp.	cleithrum	1		
LA3	Clarias sp.	coracoid	1	70-80	1
LA3	Clarias sp.	hyomandibula	1		
LA3	Clarias sp.	cranial roof fragment	1	40-50	1
LA3	Clarias sp.	cranial roof fragment	5		
LA3	Clarias sp.	cleithrum	1	30-40	1
LA3	Clarias sp.	cleithrum	1	30-40	1
LA3	Clarias sp.	cleithrum	1	30-40	1
LA3	Clarias sp.	coracoid	1	40-50	1
LA3	Clarias sp.	epi- & ceratohyale	1	40-50	1
LA3	Clarias sp.	mesethmoid	1	15-20	1
LA3	Clarias sp.	cranial roof fragment	1		
LA3	Clarias sp.	pectoral spine	1	30-40	1
LA3	Clarias sp.	pectoral spine	1	40-50	1
LA3	Clarias sp.	cranial roof fragment	10		
LA3	Clarias sp.	cleithrum	2	30-40	1
LA3	Clarias sp.	cleithrum	2	40-50	1
LA3	Clarias sp.	coracoid	2	30-40	1
LA3	Haplotilapiini indet.	fin spine	1	20-25	1
LA3	Haplotilapiini indet.	skull roof fragment	1	20-25	1
LA3	Haplotilapiini indet.	opercular	1	25-30	
LA3	Haplotilapiini indet.	subopercular	1	20-25	1
LA3	Haplotilapiini indet.	cleithrum	4	20-25	1
LA3	Clarias sp.	cleithrum	1		
LA3	Clarias sp.	coracoid	1		
LA3	Clarias sp.	precaudal vertebra	1	60-70	1
LA3	Clarias sp.	pectoral spine	1	70-80	1
LA3	Clarias sp.	pectoral spine	1	50-60	1
LA3	Clarias sp.	caudal vertebra	2	60-70	1
LA3	Clarias sp.	cranial roof fragment	5		
LA3	Clarias sp.	cleithrum	1		
LA3	Clarias sp.	cleithrum	1	60-70	1
LA3	Clarias sp.	hyomandibula	1		
LA3	Clarias sp.	cranial roof fragment	1	40-50	1
LA3	Clarias sp.	cranial roof fragment	1	70-80	1
LA3	Clarias sp.	cranial roof fragment	1		
LA3	Clarias sp.	pectoral spine	1	60-70	1
LA3	Clarias sp.	cranial roof fragment	7		
LA3	Clarias sp.	articular & dentary	1	40-50	1
LA3	Clarias sp.	cranial roof fragment	1	50-60	1
LA3	Clarias sp.	cranial roof fragment	3		
LA3	Clarias sp.	cranial roof fragment	1	40-50	1
LA3	Clarias sp.	articular	1	40-50	1
LA3	Clarias sp.	precaudal vertebra	1	50-60	1
LA3	Clarias sp.	caudal vertebra	3	60-70	1
LA3	Clarias sp.	cranial roof fragment	7		
LA3	Haplotilapiini indet.	caudal vertebra	1	20-25	1
LA3	Haplotilapiini indet.	unidentified	1		
LA3	Haplotilapiini indet.	fin spine	1		

cultural phase	taxon	skeletal element	NISP	SL in cm	number
LA3	Haplotilapiini indet.	precaudal vertebra	1	20-25	1
LA3	Clarias sp.	cleithrum	1	30-40	1
LA3	Clarias sp.	opercular	1	60-70	1
LA3	Clarias sp.	pectoral spine	1	50-60	1
LA3	Clarias sp.	cranial roof fragment	3		
LA3	Clarias sp.	cranial roof fragment	1	40-50	1
LA3	Clarias sp.	quadrate	1	30-40	1
LA3	Clarias sp.	cranial roof fragment	6		
LA3	Haplotilapiini indet.	opercular	1		
LA3	Clarias sp.	caudal vertebra	1	60-70	1
LA3	Clarias sp.	cleithrum	1		
LA3	Clarias sp.	coracoid	1		
LA3	Clarias sp.	hyomandibula	1	30-40	1
LA3	Clarias sp.	cranial roof fragment	1		
LA3	Clarias sp.	cranial roof fragment	4		
LA3	Clarias sp.	cleithrum	1	60-70	1
LA3	Clarias sp.	coracoid	1		
LA3	Clarias sp.	ectopterygoid	1		
LA3	Clarias sp.	epi- & ceratohyale	1	60-70	1
LA3	Clarias sp.	branchial element	1		
LA3	Clarias sp.	cranial roof fragment	1	40-50	1
LA3	Clarias sp.	cranial roof fragment	1		
LA3	Clarias sp.	precaudal vertebra	1	60-70	1
LA3	Clarias sp.	pectoral spine	1	70-80	1
LA3	Clarias sp.	pectoral spine	1	40-50	1
LA3	Clarias sp.	quadrate	1	40-50	1
LA3	Clarias sp.	caudal vertebra	2	60-70	1
LA3	Clarias sp.	caudal vertebra	2	40-50	1
LA3	Clarias sp.	cranial roof fragment	6		
LA3	Haplotilapiini indet.	opercular	1		
LA3	Clarias sp.	cranial roof fragment	1		
LA3	Clarias sp.	cranial roof fragment	1	70-80	1
LA3	Clarias sp.	opercular	1	20-25	1
LA3	Clarias sp.	preopercular	1	20-25	1
LA3	Clarias sp.	caudal vertebra	2	70-80	1
LA3	Clarias sp.	caudal vertebra	1		
LA3	Clarias sp.	caudal vertebra	1	60-70	1
LA3	Clarias sp.	cranial roof fragment	1	40-50	1
LA3	Clarias sp.	cranial roof fragment	1		
LA3	Clarias sp.	precaudal vertebra	1		
LA3	Clarias sp.	precaudal vertebra	1	20-25	1
LA3	Clarias sp.	postcleithrum	1	20-25	1
LA3	Clarias sp.	caudal vertebra	1	30-40	1
LA3	Clarias sp.	cleithrum	1	30-40	1
LA3	Clarias sp.	coracoid	1	30-40	1
LA3	Clarias sp.	cranial roof fragment	2		
LA3	Haplotilapiini indet.	opercular	1	15-20	1
LA3	Clarias sp.	articular	1	30-40	1
LA3	Clarias sp.	articular	1	20-25	1
LA3	Clarias sp.	articular	1	30-40	1
LA3	Clarias sp.	caudal vertebra	1	40-50	1
LA3	Clarias sp.	cleithrum	1	30-40	1
LA3	Clarias sp.	cleithrum	1	20-30	1
LA3	Clarias sp.	cleithrum	1	30-40	1
LA3	Clarias sp.	cleithrum	1	40-50	1
LA3	Clarias sp.	cleithrum	1		
LA3	Clarias sp.	cranial roof fragment	1	30-40	1
LA3	Clarias sp.	precaudal vertebra	1		

cultural phase	taxon	skeletal element	NISP	SL in cm	number
LA3	Clarias sp.	pectoral spine	1	40-50	1
LA3	Clarias sp.	quadrate	1	30-40	1
LA3	Clarias sp.	quadrate	1	50-60	1
LA3	Clarias sp.	quadrate	1	50-60	1
LA3	Clarias sp.	cranial roof fragment	14		
LA3	Clarias sp.	caudal vertebra	2	50-60	1
LA3	Clarias sp.	caudal vertebra	2	60-70	1
LA3	Clarias sp.	coracoid	2	40-50	1
LA3	Clarias sp.	cranial roof fragment	2	50-60	1
LA3	Clarias sp.	precaudal vertebra	2	50-60	1
LA3	Haplotilapiini indet.	lepidotrich	2		
LA3	Haplotilapiini indet.	anal pterygophore	1	20-25	1
LA3	Haplotilapiini indet.	anal pterygophore	1	20-25	1
LA3	Haplotilapiini indet.	basipterygium	1	20-25	1
LA3	Haplotilapiini indet.	dentary	1		
LA3	Haplotilapiini indet.	skull roof fragment	1		
LA3	Haplotilapiini indet.	opercular	1	15-20	1
LA3	Haplotilapiini indet.	opercular	1	25-30	1
LA3	Haplotilapiini indet.	precaudal vertebra	1	15-20	1
LA3	Haplotilapiini indet.	supracleithrum	1	20-25	1
LA3	Haplotilapiini indet.	caudal vertebra	3	15-20	1
LA3	Haplotilapiini indet.	cleithrum	3	20-25	1
LA3	Haplotilapiini indet.	caudal vertebra	4	20-25	1
LA3	Haplotilapiini indet.	dorsal or anal pterygophore	5		
LA3	Haplotilapiini indet.	precaudal vertebra	7	20-25	1
LA3	Haplotilapiini indet.				
LA3	Clarias sp.	articular	1	90-100	1
LA3	Clarias sp.	caudal vertebra	1	60-70	1
LA3	Clarias sp.	ceratohyale	1	60-70	1
LA3	Clarias sp.	cleithrum	1	30-40	1
LA3	Clarias sp.	cleithrum	1		
LA3	Clarias sp.	coracoid	1	40-50	1
LA3	Clarias sp.	coracoid	1		
LA3	Clarias sp.	cranial roof fragment	1		
LA3	Clarias sp.	cranial roof fragment	1	70-80	1
LA3	Clarias sp.	quadrate	1	70-80	1
LA3	Clarias sp.	cranial roof fragment	2	60-70	1
LA3	Clarias sp.	cranial roof fragment	4		
LA3	Haplotilapiini indet.	basipterygium	1	20-25	1
LA3	Haplotilapiini indet.	opercular	1		
LA3	Haplotilapiini indet.	precaudal vertebra	1	15-20	1
LA3	Clarias sp.	caudal vertebra	1	60-70	1
LA3	Clarias sp.	ceratohyale	1	50-60	1
LA3	Clarias sp.	dentary	1		
LA3	Clarias sp.	mesethmoid	1	40-50	1
LA3	Clarias sp.	pectoral spine	1	60-70	1
LA3	Clarias sp.	cranial roof fragment	11		
LA3	Clarias sp.	caudal vertebra	3	50-60	1
LA3	Haplotilapiini indet.	cleithrum	1		
LA3	Clarias sp.	articular & dentary	1	40-50	1
LA3	Clarias sp.	caudal vertebra	1	60-70	1
LA3	Clarias sp.	caudal vertebra	1	50-60	1
LA3	Clarias sp.	caudal vertebra	1	60-70	1
LA3	Clarias sp.	cleithrum	1	50-60	1
LA3	Clarias sp.	dentary	1	60-70	1
LA3	Clarias sp.	epi- & ceratohyale	1	40-50	1
LA3	Clarias sp.	hyomandibula	1	70-80	1
LA3	Clarias sp.	unidentified	1		

cultural phase	taxon	skeletal element	NISP	SL in cm	number
LA3	Clarias sp.	branchial element	1		
LA3	Clarias sp.	cranial roof fragment	1		
LA3	Clarias sp.	opercular	1	30-40	1
LA3	Clarias sp.	precaudal vertebra	1	60-70	1
LA3	Clarias sp.	cranial roof fragment	7		
LA3	Clarias sp.	cranial roof fragment	7		
LA3	Haplotilapiini indet.	basipterygium	1		
LA3	Clarias sp.	caudal vertebra	1	60-70	1
LA3	Clarias sp.	hyomandibula	1	60-70	1
LA3	Clarias sp.	cranial roof fragment	1		
LA3	Clarias sp.	cranial roof fragment	2	40-50	1
LA3	Haplotilapiini indet.	anal pterygophore	1	20-25	1
LA3	Haplotilapiini indet.	ceratohyale	1	20-25	1
LA3	Haplotilapiini indet.	unidentified	1		
LA3	Haplotilapiini indet.	skull roof fragment	1		
LA3	Clarias sp.	cleithrum	1		
LA3	Clarias sp.	cleithrum	1	40-50	1
LA3	Clarias sp.	coracoid	1	40-50	1
LA3	Clarias sp.	cranial roof fragment	1		
LA3	Clarias sp.	cranial roof fragment	1	30-40	1
LA3	Clarias sp.	cranial roof fragment	2		
LA3	Clarias sp.	caudal vertebra	3		
LA3	Clarias sp.	cranial roof fragment	7		
LA3	Haplotilapiini indet.	cleithrum	1	20-25	1
LA3	Haplotilapiini indet.	cleithrum	1		
LA3	Haplotilapiini indet.	cleithrum	2	20-25	1
LA3	Clarias sp.	caudal vertebra	1	40-50	1
LA3	Clarias sp.	cleithrum	1		
LA3	Clarias sp.	quadrate	1	30-40	1
LA3	Clarias sp.	cranial roof fragment	3		
LA3	Clarias sp.	cleithrum	1		
LA3	Clarias sp.	dentary	1	30-40	1
LA3	Clarias sp.	mesethmoid	1	40-50	1
LA3	Clarias sp.	mesethmoid	1	50-60	1
LA3	Clarias sp.	cranial roof fragment	1	40-50	1
LA3	Clarias sp.	precaudal vertebra	1	30-40	1
LA3	Clarias sp.	pectoral spine	1	60-70	1
LA3	Clarias sp.	cranial roof fragment	12		
LA3	C. gariepinus	vomerine toothplate	1	60-70	1
LA3	Clarias sp.	articular	1		
LA3	Clarias sp.	articular & dentary	1	50-60	1
LA3	Clarias sp.	caudal vertebra	1	60-70	1
LA3	Clarias sp.	hyomandibula	1	60-70	1
LA3	Clarias sp.	pectoral spine	1	60-70	1
LA3	Clarias sp.	cranial roof fragment	4		
LA3	Clarias sp.	cranial roof fragment	3		
LA3	Haplotilapiini indet.	skull roof fragment	1		
LA3	Haplotilapiini indet.	opercular	1	25-30	1
LA3	Clarias sp.	cranial roof fragment	2		
LA3	Haplotilapiini indet.	cleithrum	1	20-25	1
LA3	Clarias sp.	cranial roof fragment	2		
LA3	Clarias sp.	caudal vertebra	1	60-70	1
LA3	Clarias sp.	ceratohyale	1	40-50	1
LA3	Clarias sp.	cleithrum	1	50-60	1
LA3	Clarias sp.	cleithrum	1	30-40	1
LA3	Clarias sp.	cleithrum	1	40-50	1
LA3	Clarias sp.	cleithrum	1	50-60	1
LA3	Clarias sp.	coracoid	1		

cultural phase	taxon	skeletal element	NISP	SL in cm	number
LA3	Clarias sp.	hyomandibula	1		
LA3	Clarias sp.	hyomandibula	1	50-60	1
LA3	Clarias sp.	mesethmoid	1	30-40	1
LA3	Clarias sp.	cranial roof fragment	1	40-50	1
LA3	Clarias sp.	cranial roof fragment	1	50-60	1
LA3	Clarias sp.	cranial roof fragment	1	50-60	1
LA3	Clarias sp.	cranial roof fragment	1		
LA3	Clarias sp.	precaudal vertebra	1	60-70	1
LA3	Clarias sp.	pectoral spine	1	70-80	1
LA3	Clarias sp.	cranial roof fragment	9		
LA3	Haplotilapiini indet.	cleithrum	1	20-25	1
LA3	Haplotilapiini indet.	skull roof fragment	1	20-25	1
LA3	Haplotilapiini indet.	opercular	1	20-25	1
LA3	Haplotilapiini indet.	opercular	1	25-30	1
LA3	Clarias sp.	caudal vertebra	1	60-70	1
LA3	Clarias sp.	cleithrum	1	90-100	1
LA3	Clarias sp.	coracoid	1	50-60	1
LA3	Clarias sp.	cranial roof fragment	1	80-90	1
LA3	Clarias sp.	cranial roof fragment	2		
LA3	Clarias sp.	cranial roof fragment	2	50-60	1
LA3	Haplotilapiini indet.	cleithrum	1	15-20	1
LA3	Clarias sp.	coracoid	1		
LA3	Clarias sp.	cranial roof fragment	1	60-70	1
LA3	Clarias sp.	caudal vertebra	3	60-70	1
LA3	Clarias sp.	cranial roof fragment	7		
LA3	Clarias sp.	cleithrum	1	30-40	1
LA3	Clarias sp.	cranial roof fragment	1	40-50	1
LA3	Clarias sp.	opercular	1	50-60	1
LA3	Clarias sp.	opercular	1	50-60	1
LA3	Clarias sp.	cranial roof fragment	5		
LA3	Clarias sp.	cleithrum	1		
LA3	Clarias sp.	cranial roof fragment	2		
LA3	Clarias sp.	dentary	1		
LA3	Clarias sp.	cranial roof fragment	1		
LA3	Clarias sp.	caudal vertebra	1	60-70	1
LA3	Clarias sp.	cranial roof fragment	1		
LA3	Haplotilapiini indet.	opercular	2		
LA3	Clarias sp.	epihyale	1	70-80	1
LA3	Clarias sp.	cranial roof fragment	1		
LA3	Clarias sp.	caudal vertebra	1		
LA3	Clarias sp.	cleithrum	1	50-60	1
LA3	Clarias sp.	cleithrum	1	20-25	1
LA3	Clarias sp.	epi- & ceratohyale	1	80-90	1
LA3	Clarias sp.	unidentified	1		
LA3	Clarias sp.	cranial roof fragment	1		
LA3	Clarias sp.	cranial roof fragment	1		
LA3	Clarias sp.	precaudal vertebra	1		
LA3	Clarias sp.	quadrate	1	40-50	1
LA3	Clarias sp.	cleithrum	1	40-50	1
LA3	Clarias sp.	cranial roof fragment	2		
LA3	Clarias sp.	cranial roof fragment	3		
LA3	Clarias sp.	articular	1	60-70	1
LA3	Clarias sp.	articular	1	40-50	1
LA3	Clarias sp.	cleithrum	1	50-60	1
LA3	Clarias sp.	cranial roof fragment	4		
LA3	Haplotilapiini indet.	cleithrum	1	20-25	1
LA3	C. gariepinus	vomerine toothplate	1	40-50	1
LA3	C. gariepinus	vomerine toothplate	1		

cultural phase	taxon	skeletal element	NISP	SL in cm	number
LA3	Clarias sp.	articular	1	50-60	1
LA3	Clarias sp.	caudal vertebra	1	50-60	1
LA3	Clarias sp.	cleithrum	1	20-30	1
LA3	Clarias sp.	cleithrum	1	50-60	1
LA3	Clarias sp.	epi- & ceratohyale	1	50-60	1
LA3	Clarias sp.	epi- & ceratohyale	1	50-60	1
LA3	Clarias sp.	hyomandibula	1	40-50	1
LA3	Clarias sp.	hyomandibula	1		
LA3	Clarias sp.	mesethmoid	1	20-30	1
LA3	Clarias sp.	mesethmoid	1	50-60	1
LA3	Clarias sp.	mesethmoid	1	60-70	1
LA3	Clarias sp.	cranial roof fragment	1	40-50	1
LA3	Clarias sp.	opercular	1	60-70	1
LA3	Clarias sp.	pectoral spine	1	50-60	1
LA3	Clarias sp.	pectoral spine	1	30-40	1
LA3	Clarias sp.	cranial roof fragment	10		
LA3	Clarias sp.	cleithrum	2	30-40	1
LA3	Clarias sp.	cranial roof fragment	2	30-40	1
LA3	Clarias sp.	caudal vertebra	3		
LA3	Haplotilapiini indet.	anal pterygophore	1	20-25	1
LA3	Haplotilapiini indet.	cleithrum	1		
LA3	O. niloticus	hyomandibula	1	20-25	1
LA3	Haplotilapiini indet.	opercular	1	20-25	1
LA3	Clarias sp.	cranial roof fragment	1	50-60	1
LA3	Clarias sp.	cleithrum	1	60-70	1
LA3	Clarias sp.	cleithrum	1	60-70	1
LA3	Clarias sp.	cleithrum	1		
LA3	Clarias sp.	opercular	1	40-50	1
LA3	Clarias sp.	precaudal vertebra	1	60-70	1
LA3	Clarias sp.	precaudal vertebra	1	70-80	1
LA3	Haplotilapiini indet.	cleithrum	1	20-25	1
LA3	Haplotilapiini indet.	maxilla	1	20-25	1
LA3	Haplotilapiini indet.	opercular	1	25-30	1
LA3	Clarias sp.	caudal vertebra	1	60-70	1
LA3	Clarias sp.	cleithrum	1	30-40	1
LA3	Clarias sp.	cranial roof fragment	1	30-40	1
LA3	Clarias sp.	pectoral spine	1		
LA3	Clarias sp.	preopercular	1	20-25	1
LA3	Clarias sp.	cranial roof fragment	2	60-70	1
LA3	Clarias sp.	cranial roof fragment	4		
LA3	Clarias sp.	caudal vertebra	1		
LA3	Clarias sp.	hyomandibula	1	30-40	1
LA3	Clarias sp.	pectoral spine	1	20-30	1
LA3	Clarias sp.	quadrate	1	20-30	1
LA3	Clarias sp.	cranial roof fragment	2		
LA3	Haplotilapiini indet.	precaudal vertebra	1	20-25	1
LA3	Clarias sp.	articular & dentary	1	30-40	1
LA3	Clarias sp.	coracoid	1		
LA3	Clarias sp.	cranial roof fragment	1	30-40	1
LA3	Clarias sp.	cranial roof fragment	2		
LA3	Haplotilapiini indet.	precaudal vertebra	1	15-20	1
LA3	Haplotilapiini indet.	cleithrum	2	20-25	1
LA3	Haplotilapiini indet.	lepidotrich	2		
LA3	C. gariepinus	vomerine toothplate	1	60-70	1
LA3	Clarias sp.	caudal vertebra	1		
LA3	Clarias sp.	cleithrum	1	50-60	1
LA3	Clarias sp.	hypo- & ceratohyale	1	50-60	1
LA3	Clarias sp.	mesethmoid	1	40-50	1

cultural phase	taxon	skeletal element	NISP	SL in cm	number
LA3	Clarias sp.	cranial roof fragment	1	50-60	1
LA3	Clarias sp.	pectoral spine	1	40-50	1
LA3	Clarias sp.	pectoral spine	1	60-70	1
LA3	Clarias sp.	pectoral spine	1	60-70	1
LA3	Clarias sp.	pectoral spine	1	60-70	1
LA3	Clarias sp.	pectoral spine	1	50-60	1
LA3	Clarias sp.	quadrate	1	60-70	1
LA3	Clarias sp.	cranial roof fragment	11		
LA3	Clarias sp.	cranial roof fragment	4	40-50	1
LA3	Haplotilapiini indet.	skull roof fragment	1		
LA3	Haplotilapiini indet.	precaudal vertebra	1	20-25	1
LA3	Haplotilapiini indet.	quadrate	1	25-30	1
LA3	Clarias sp.	cleithrum	1	30-40	1
LA3	Clarias sp.	cleithrum	1		
LA3	Clarias sp.	cranial roof fragment	1	40-50	1
LA3	Clarias sp.	cranial roof fragment	1		
LA3	Clarias sp.	quadrate	1	40-50	1
LA3	Clarias sp.	urohyale	1	50-60	1
LA3	Clarias sp.	cranial roof fragment	3		
LA3	Clarias sp.	cranial roof fragment	2		
LA3	Clarias sp.	cranial roof fragment	1		
LA3	Clarias sp.	caudal vertebra	1	30-40	1
LA3	Clarias sp.	caudal vertebra	1	40-50	1
LA3	Clarias sp.	cleithrum	1	20-30	1
LA3	Clarias sp.	cleithrum	1		
LA3	Clarias sp.	cleithrum	1		
LA3	Clarias sp.	coracoid	1		
LA3	Clarias sp.	dentary	1	50-60	1
LA3	Clarias sp.	epihyale	1	80-90	1
LA3	Clarias sp.	epihyale	1		
LA3	Clarias sp.	hyomandibula	1		
LA3	Clarias sp.	cranial roof fragment	1	70-80	1
LA3	Clarias sp.	precaudal vertebra	1	40-50	1
LA3	Clarias sp.	pectoral spine	1	50-60	1
LA3	Clarias sp.	pectoral spine	1	30-40	1
LA3	Clarias sp.	pectoral spine	1		
LA3	Clarias sp.	caudal vertebra	3	60-70	1
LA3	Clarias sp.	cranial roof fragment	9		
LA3	Haplotilapiini indet.	cleithrum	1		
LA3	O. niloticus	hyomandibula	1	15-20	1
LA3	O. niloticus	mesethmoid	1	25-30	1
LA3	Haplotilapiini indet.	precaudal vertebra	1	20-25	1
LA3	Haplotilapiini indet.	skull roof fragment	2		
LA3	Clarias sp.	epihyale	1	70-80	1
LA3	Clarias sp.	cranial roof fragment	3		
LA3	Haplotilapiini indet.	cleithrum	1	20-25	1
LA3	Haplotilapiini indet.	skull roof fragment	1		
LA3	Haplotilapiini indet.	parasphenoid	1	15-20	1
LA3	Haplotilapiini indet.	precaudal vertebra	1		
LA3	Clarias sp.	articular	1	30-40	1
LA3	Clarias sp.	cranial roof fragment	1	30-40	1
LA3	Clarias sp.	cranial roof fragment	2		
LA3	Haplotilapiini indet.	basipterygium	1	20-25	1
LA3	Haplotilapiini indet.	caudal vertebra	1	15-20	1
LA3	Haplotilapiini indet.	caudal vertebra	1	20-25	1
LA3	Haplotilapiini indet.	cleithrum	1		
LA3	Haplotilapiini indet.	cleithrum	1	20-25	1
LA3	Haplotilapiini indet.	fin spine	1		

cultural phase	taxon	skeletal element	NISP	SL in cm	number
LA3	Haplotilapiini indet.	precaudal vertebra	1	20-25	1
LA3	Clarias sp.	epi- & ceratohyale	1	30-40	1
LA3	Clarias sp.	cranial roof fragment	1		
LA3	Clarias sp.	epi- & ceratohyale	1	40-50	1
LA3	Clarias sp.	pectoral spine	1	40-50	1
LA3	Clarias sp.	cranial roof fragment	2	60-70	1
LA3	Clarias sp.	cranial roof fragment	2		
LA3	Clarias sp.	cranial roof fragment	1	60-70	1
LA3	Clarias sp.	cranial roof fragment	4		
LA3	Clarias sp.	cleithrum	1	60-70	1
LA3	Clarias sp.	cleithrum	1	60-70	1
LA3	Clarias sp.	cranial roof fragment	1	90-100	1
LA3	Clarias sp.	cranial roof fragment	1		
LA3	O. niloticus	1st precaudal vertebra	1	20-25	1
LA3	Haplotilapiini indet.	1st precaudal vertebra	1	20-25	1
LA3	O. niloticus	1st precaudal vertebra	1	20-25	1
LA3	O. niloticus	1st precaudal vertebra	1	25-30	1
LA3	O. niloticus	1st precaudal vertebra	1	10-15	1
LA3	Clarias sp.	hypohyale	1	90-100	1
LA3	Haplotilapiini indet.	suborbital	1		
LA3	Clarias sp.	mesethmoid	1	50-60	1
LA3	Clarias sp.	caudal vertebra	1	40-50	1
LA3	Clarias sp.	cleithrum	1		
LA3	Clarias sp.	hyomandibula	1	40-50	1
LA3	Clarias sp.	cranial roof fragment	2		
LA3	Clarias sp.	articular	1	30-40	1
LA3	Clarias sp.	articular	1	30-40	1
LA3	Clarias sp.	basioccipital	1	30-40	1
LA3	Clarias sp.	ceratohyale	1	30-40	1
LA3	Clarias sp.	dentary	1	20-30	1
LA3	Clarias sp.	cranial roof fragment	1	20-30	1
LA3	Clarias sp.	cranial roof fragment	1	40-50	1
LA3	Clarias sp.	cranial roof fragment	1	60-70	1
LA3	Clarias sp.	vomerine toothplate	1	50-60	1
LA3	Clarias sp.	cranial roof fragment	8		
LA3	Clarias sp.	articular	1	50-60	1
LA3	Clarias sp.	caudal vertebra	1	40-50	1
LA3	Clarias sp.	precaudal vertebra	1	50-60	1
LA3	Clarias sp.	cranial roof fragment	3		
LA3	Clarias sp.	mesethmoid	1	20-30	1
LA3	Clarias sp.	cranial roof fragment	5		
LA3	Clarias sp.	caudal vertebra	1	40-50	1
LA3	Clarias sp.	coracoid	1		
LA3	Clarias sp.	vertebra	1		
LA3	Clarias sp.	cranial roof fragment	4	60-70	1
LA3	Clarias sp.	articular	1	30-40	1
LA3	Clarias sp.	articular	1	40-50	1
LA3	Clarias sp.	articular	1	40-50	1
LA3	Clarias sp.	articular	1	60-70	1
LA3	Clarias sp.	basioccipital	1	40-50	1
LA3	Clarias sp.	ceratohyale	1	50-60	1
LA3	Clarias sp.	cleithrum	1	30-40	1
LA3	Clarias sp.	cleithrum	1	30-40	1
LA3	Clarias sp.	cleithrum	1	50-60	1
LA3	Clarias sp.	cleithrum	1		
LA3	Clarias sp.	dentary	1	40-50	1
LA3	Clarias sp.	dentary	1		
LA3	Clarias sp.	precaudal vertebra	1	40-50	1

cultural phase	taxon	skeletal element	NISP	SL in cm	number
LA3	Clarias sp.	pectoral spine	1	40-50	1
LA3	Clarias sp.	pectoral spine	1	50-60	1
LA3	Clarias sp.	pectoral spine	1	50-60	1
LA3	Clarias sp.	cranial roof fragment	11		
LA3	Clarias sp.	cranial roof fragment	2	50-60	2
LA3	Clarias sp.	cranial roof fragment	2	60-70	2
LA3	Clarias sp.	caudal vertebra	5	50-60	5
LA3	Clarias sp.	caudal vertebra	5	60-70	5
LA3	Clarias sp.	caudal vertebra	7	40-50	7
LA3	Clarias sp.	coracoid	1		
LA3	Clarias sp.	cranial roof fragment	3	60-70	1
LA3	Clarias sp.	basioccipital	1	50-60	1
LA3	Clarias sp.	caudal vertebra	1	60-70	1
LA3	Clarias sp.	pectoral spine	1	30-40	1
LA3	Clarias sp.	cranial roof fragment	2	60-70	1
LA3	Clarias sp.	precaudal vertebra	2	60-70	2
LA3	Clarias sp.	basioccipital	1	70-80	1
LA3	Clarias sp.	caudal vertebra	1	30-40	1
LA3	Clarias sp.	ceratohyale	1	40-50	1
LA3	Clarias sp.	cleithrum	1		
LA3	Clarias sp.	epihyale	1	50-60	1
LA3	Clarias sp.	epihyale	1		
LA3	Clarias sp.	cranial roof fragment	1	20-30	1
LA3	Clarias sp.	quadrate	1	80-90	1
LA3	Clarias sp.	caudal vertebra	2	40-50	2
LA3	Clarias sp.	cranial roof fragment	2	60-70	2
LA3	Clarias sp.	precaudal vertebra	2	40-50	2
LA3	Clarias sp.	caudal vertebra	5	60-70	5
LA3	Clarias sp.	basioccipital	1	40-50	1
LA3	Clarias sp.	cleithrum	1	20-30	1
LA3	Clarias sp.	cleithrum	1	30-40	1
LA3	Clarias sp.	cleithrum	1		
LA3	Clarias sp.	epihyale	1	40-50	1
LA3	Clarias sp.	pectoral spine	1	60-70	1
LA3	Clarias sp.	pectoral spine	1	60-70	1
LA3	Clarias sp.	pectoral spine	1	50-60	1
LA3	Clarias sp.	caudal vertebra	2	40-50	2
LA3	Clarias sp.	cranial roof fragment	4		
LA3	Clarias sp.	vomerine toothplate	1	60-70	1
LA3	Clarias sp.	articular	1	60-70	1
LA3	Clarias sp.	articular	1	40-50	1
LA3	Clarias sp.	caudal vertebra	1	60-70	1
LA3	Clarias sp.	cleithrum	1		
LA3	Clarias sp.	cranial roof fragment	4	40-50	1
LA3	Clarias sp.	ceratohyale	1	40-50	1
LA3	Clarias sp.	pectoral spine	1	70-80	1
LA3	Clarias sp.	cranial roof fragment	2	60-70	1
LA3	Clarias sp.	cranial roof fragment	1		
LA3	Clarias sp.	epihyale	1	60-70	1
LA3	Clarias sp.	hyomandibula	1	60-70	1
LA3	Clarias sp.	cranial roof fragment	1	50-60	1
LA3	Clarias sp.	cranial roof fragment	1	60-70	1
LA3	Clarias sp.	palatinum	1	40-50	1
LA3	Clarias sp.	precaudal vertebra	1	50-60	1
LA3	Clarias sp.	pectoral spine	1		
LA3	Clarias sp.	coracoid	2		
LA3	Clarias sp.	cleithrum	3		
LA3	Clarias sp.	caudal vertebra	4	40-50	4

cultural phase	taxon	skeletal element	NISP	SL in cm	number
LA3	Clarias sp.	caudal vertebra	4	60-70	4
LA3	Clarias sp.	cranial roof fragment	9		
LA3	Haplotilapiini indet.	articular	1	25-30	1
LA3	Haplotilapiini indet.	basioccipital	1	25-30	1
LA3	Haplotilapiini indet.	cleithrum	1	20-25	1
LA3	Haplotilapiini indet.	precaudal vertebra	1	20-25	1
LA3	Haplotilapiini indet.	preopercular	1		
LA3	Haplotilapiini indet.	caudal vertebra	1		
LA3	Haplotilapiini indet.	hyomandibula	1	15-20	1
LA3	Haplotilapiini indet.	precaudal vertebra	1	20-25	1
LA3	Haplotilapiini indet.	precaudal vertebra	1	25-30	1
LA3	Haplotilapiini indet.	fin spine	1		
LA3	Haplotilapiini indet.	skull roof fragment	1		
LA3	Haplotilapiini indet.	precaudal vertebra	1	25-30	1
LA3	Haplotilapiini indet.	dorsal or anal pterygophore	1		
LA3	Haplotilapiini indet.	subopercular	1	25-30	1
LA3	Haplotilapiini indet.	skull roof fragment	1		
LA3	O. niloticus	hyomandibula	1	15-20	1
LA3	Haplotilapiini indet.	dorsal or anal pterygophore	1	20-25	1
LA3	Haplotilapiini indet.	fin spine	1		
LA3	Haplotilapiini indet.	precaudal vertebra	1	20-25	1
LA3	Haplotilapiini indet.	precaudal vertebra	1	25-30	1
LA3	Haplotilapiini indet.	dorsal or anal pterygophore	1	20-25	1
LA3	Haplotilapiini indet.	cleithrum	2		
LA3	Haplotilapiini indet.	cleithrum	1		
LA3	Haplotilapiini indet.	skull roof fragment	1		
LA3	Haplotilapiini indet.	opercular	1	15-20	1
LA3	Haplotilapiini indet.	skull roof fragment	1		
LA3	Haplotilapiini indet.	articular	1	25-30	1
LA3	Haplotilapiini indet.	basioccipital	1	25-30	1
LA3	Haplotilapiini indet.	basipterygium	1	15-20	1
LA3	Haplotilapiini indet.	basipterygium	1	20-25	1
LA3	Haplotilapiini indet.	opercular	1		
LA3	Haplotilapiini indet.	dorsal or anal pterygophore	1	15-20	1
LA3	Haplotilapiini indet.	caudal vertebra	2	15-20	2
LA3	Haplotilapiini indet.	skull roof fragment	2		
LA3	Haplotilapiini indet.	precaudal vertebra	3	20-25	3
LA3	Haplotilapiini indet.	cleithrum	5		
LA3	Haplotilapiini indet.	precaudal vertebra	8	15-20	8
LA3	Haplotilapiini indet.	basipterygium	1	25-30	1
LA3	Haplotilapiini indet.	ectopterygoid	1		
LA3	Haplotilapiini indet.	3rd precaudal vertebra	1	20-25	1
LA3	Haplotilapiini indet.	precaudal vertebra	1	20-25	1
LA3	Haplotilapiini indet.	posttemporal	1	20-25	1
LA3	Haplotilapiini indet.	precaudal vertebra	2	15-20	2
LA3	Haplotilapiini indet.	dorsal or anal pterygophore	2		
LA3	Haplotilapiini indet.	skull roof fragment	3		
LA3	Haplotilapiini indet.	cleithrum	4		
LA3	Haplotilapiini indet.	cleithrum	1		
LA3	Haplotilapiini indet.	skull roof fragment	2		
LA3	Haplotilapiini indet.	precaudal vertebra	3	15-20	3
LA3	Haplotilapiini indet.	skull roof fragment	2		
LA3	Haplotilapiini indet.	basipterygium	1	20-25	1
LA3	Haplotilapiini indet.	caudal vertebra	1	25-30	1
LA3	Haplotilapiini indet.	skull roof fragment	1		
LA3	Haplotilapiini indet.	palatinum	1	15-20	1
LA3	Haplotilapiini indet.	2nd precaudal vertebra	1	25-30	1
LA3	Haplotilapiini indet.	dorsal or anal pterygophore	1		

cultural phase	taxon	skeletal element	NISP	SL in cm	number
LA3	Haplotilapiini indet.	scapula	1	15-20	1
LA3	Haplotilapiini indet.	caudal vertebra	3	15-20	3
LA3	Haplotilapiini indet.	cleithrum	4	15-20	1
LA3	Haplotilapiini indet.	precaudal vertebra	6	15-20	6
LA3	Clarias sp.	articular	1	30-40	1
LA3	Clarias sp.	caudal vertebra	1	40-50	1
LA3	Clarias sp.	coracoid	1		
LA3	Clarias sp.	cranial roof fragment	1		
LA3	Clarias sp.	quadrate	1	50-60	1
LA3	Clarias sp.	pectoral spine	1	30-40	1
LA3	Clarias sp.	caudal vertebra	2	60-70	2
LA3	Clarias sp.	cranial roof fragment	2	60-70	1
LA3	Clarias sp.	cranial roof fragment	4		
LA3	Haplotilapiini indet.	cleithrum	1		
LA3	Haplotilapiini indet.	hyomandibula	1	15-20	1
LA3	Haplotilapiini indet.	opercular	1	15-20	1
LA3	O. niloticus	palatinum	1	25-30	1
LA3	Haplotilapiini indet.	dorsal or anal pterygophore	1	25-30	1
LA3	Haplotilapiini indet.	quadrate	1	20-25	1
LA3	Haplotilapiini indet.	caudal vertebra	2	15-20	2
LA3	Haplotilapiini indet.	lepidotrich	2		
LA3	Haplotilapiini indet.	articular	1	20-25	1
LA3	Haplotilapiini indet.	basipterygium	1	15-20	1
LA3	Haplotilapiini indet.	fin spine	1		
LA3	Haplotilapiini indet.	opercular	1	20-25	1
LA3	Haplotilapiini indet.	dorsal or anal pterygophore	1		
LA3	Haplotilapiini indet.	cleithrum	3	15-20	3
LA3	Clarias sp.	cleithrum	1	20-30	1
LA3	Clarias sp.	cleithrum	1	30-40	1
LA3	Clarias sp.	hyomandibula	1	50-60	1
LA3	Clarias sp.	mesethmoid	1	50-60	1
LA3	Clarias sp.	cranial roof fragment	1	40-50	1
LA3	Clarias sp.	cranial roof fragment	1	50-60	1
LA3	Clarias sp.	cranial roof fragment	1		
LA3	Clarias sp.	precaudal vertebra	1	70-80	1
LA3	Clarias sp.	quadrate	1	50-60	1
LA3	Clarias sp.	urohyale	1	60-70	1
LA3	Clarias sp.	vomerine toothplate	1	50-60	1
LA3	Clarias sp.	articular	1	40-50	1
LA3	Clarias sp.	articular	1		
LA3	Clarias sp.	caudal vertebra	1	40-50	1
LA3	Clarias sp.	caudal vertebra	1	50-60	1
LA3	Clarias sp.	ceratohyale	1	50-60	1
LA3	Clarias sp.	cleithrum	1	60-70	1
LA3	Clarias sp.	cleithrum	1		
LA3	Clarias sp.	cleithrum	1		
LA3	Clarias sp.	branchial element	1		
LA3	Clarias sp.	cranial roof fragment	1	40-50	1
LA3	Clarias sp.	cranial roof fragment	1	80-90	1
LA3	Clarias sp.	pectoral spine	1	60-70	1
LA3	Clarias sp.	premaxilla	1	50-60	1
LA3	Clarias sp.	cranial roof fragment	2		
LA3	Clarias sp.	cranial roof fragment	4	40-50	1
LA3	Clarias sp.	ceratohyale	1	60-70	1
LA3	Clarias sp.	mesethmoid	1	50-60	1
LA3	Clarias sp.	caudal vertebra	2	50-60	2
LA3	Clarias sp.	cranial roof fragment	8		
LA3	Clarias sp.	articular	1	50-60	1

cultural phase	taxon	skeletal element	NISP	SL in cm	number
LA3	Clarias sp.	caudal vertebra	1	50-60	1
LA3	Clarias sp.	cranial roof fragment	3		
LA3	Clarias sp.	basioccipital	1	60-70	1
LA3	Clarias sp.	ceratohyale	1	60-70	1
LA3	Clarias sp.	hyomandibula	1	40-50	1
LA3	Clarias sp.	cranial roof fragment	3	70-80	1
LA3	Clarias sp.	cleithrum	1	40-50	1
LA3	Clarias sp.	cranial roof fragment	1	50-60	1
LA3	Haplotilapiini indet.	cleithrum	1		
LA3	Haplotilapiini indet.	dorsal or anal pterygophore	1	20-25	1
LA3	Haplotilapiini indet.	articular	1	25-30	1
LA3	Haplotilapiini indet.	cleithrum	1		
LA3	Haplotilapiini indet.	dentary	1		
LA3	Haplotilapiini indet.	fin spine	1		
LA3	Haplotilapiini indet.	skull roof fragment	1		
LA3	Haplotilapiini indet.	preopercular	1		
LA3	Haplotilapiini indet.	postcleithrum	1	20-25	1
LA3	Haplotilapiini indet.	unidentified	1		
LA3	Clarias sp.	caudal vertebra	1	30-40	1
LA3	Clarias sp.	caudal vertebra	1	50-60	1
LA3	Clarias sp.	caudal vertebra	1	60-70	1
LA3	Clarias sp.	cleithrum	1		
LA3	Clarias sp.	dentary	1	40-50	1
LA3	Clarias sp.	hyomandibula	1	60-70	1
LA3	Clarias sp.	cranial roof fragment	1	30-40	1
LA3	Clarias sp.	palatinum	1	70-80	1
LA3	Clarias sp.	pectoral spine	1	50-60	1
LA3	Clarias sp.	premaxilla	1	40-50	1
LA3	Clarias sp.	cranial roof fragment	11		
LA3	Clarias sp.	cranial roof fragment	2	60-70	2
LA3	Clarias sp.	ceratohyale	1	40-50	1
LA3	Clarias sp.	coracoid	1		
LA3	Clarias sp.	cranial roof fragment	1	20-30	1
LA3	Clarias sp.	cranial roof fragment	1	40-50	1
LA3	Clarias sp.	opercular	1	40-50	1
LA3	Haplotilapiini indet.	skull roof fragment	1		
LA3	Haplotilapiini indet.	opercular	1		
LA3	Clarias sp.	articular	1	60-70	1
LA3	Clarias sp.	precaudal vertebra	1	40-50	1
LA3	Clarias sp.	quadrate	1	40-50	1
LA3	Clarias sp.	caudal vertebra	3	60-70	3
LA3	Clarias sp.	cranial roof fragment	7		
LA3	Clarias sp.	articular	1	50-60	1
LA3	Clarias sp.	cleithrum	1	60-70	1
LA3	Clarias sp.	premaxilla	1	70-80	1
LA3	Clarias sp.	quadrate	1	50-60	1
LA3	Clarias sp.	quadrate	1		
LA3	Clarias sp.	cranial roof fragment	3		
LA3	Clarias sp.	precaudal vertebra	1	40-50	1
LA3	Clarias sp.	cleithrum	2		
LA3	Clarias sp.	precaudal vertebra	2	60-70	2
LA3	Clarias sp.	vomerine toothplate	2	70-80	2
LA3	Clarias sp.	cranial roof fragment	4		
LA3	Haplotilapiini indet.	skull roof fragment	1		
LA3	Haplotilapiini indet.	cleithrum	2		
LA3	Haplotilapiini indet.	1st precaudal vertebra	1	20-25	1
LA3	Haplotilapiini indet.	1st precaudal vertebra	1	25-30	1
LA3	O. niloticus	1st precaudal vertebra	1	15-20	1

cultural phase	taxon	skeletal element	NISP	SL in cm	number
LA3	O. niloticus	1st precaudal vertebra	1	20-25	1
LA3	Clarias sp.	cleithrum	1	60-70	1
LA3	Clarias sp.	palatinum	1	40-50	1
LA3	Clarias sp.	cleithrum	2		
LA3	Clarias sp.	cranial roof fragment	4		
LA3	Haplotilapiini indet.	cleithrum	1	15-20	1
LA3	Clarias sp.	cranial roof fragment	1		
LA3	Clarias sp.	pectoral spine	1	60-70	1
LA3	Clarias sp.	costa ?	1		
LA3	Clarias sp.	cranial roof fragment	1	40-50	1
LA3	Clarias sp.	cranial roof fragment	5		
LA3	Clarias sp.	basioccipital	1	60-70	1
LA3	Clarias sp.	caudal vertebra	1	30-40	1
LA3	Clarias sp.	cleithrum	1		
LA3	Clarias sp.	coracoid	1		
LA3	Clarias sp.	hyomandibula	1	30-40	1
LA3	Clarias sp.	hyomandibula	1	60-70	1
LA3	Clarias sp.	cranial roof fragment	1	70-80	1
LA3	Clarias sp.	cranial roof fragment	1	20-30	1
LA3	Clarias sp.	precaudal vertebra	1	60-70	1
LA3	Clarias sp.	pectoral spine	1	60-70	1
LA3	Clarias sp.	pectoral spine	1	50-60	1
LA3	Clarias sp.	premaxilla	1	50-60	1
LA3	Clarias sp.	quadrate	1	40-50	1
LA3	Clarias sp.	cranial roof fragment	18		
LA3	Clarias sp.	caudal vertebra	2	60-70	1
LA3	Clarias sp.	cranial roof fragment	2	60-70	2
LA3	Clarias sp.	caudal vertebra	1	60-70	1
LA3	Clarias sp.	coracoid	1	60-70	1
LA3	Clarias sp.	mesethmoid	1	60-70	1
LA3	Clarias sp.	precaudal vertebra	1	60-70	1
LA3	Clarias sp.	cranial roof fragment	3		
LA3	Clarias sp.	caudal vertebra	1	50-60	1
LA3	Clarias sp.	caudal vertebra	1	60-70	1
LA3	Clarias sp.	ceratohyale	1	60-70	1
LA3	Clarias sp.	ceratohyale	1	40-50	1
LA3	Clarias sp.	cleithrum	1	50-60	1
LA3	Clarias sp.	cleithrum	1	50-60	1
LA3	Clarias sp.	opercular	1		
LA3	Clarias sp.	pectoral spine	1	50-60	1
LA3	Clarias sp.	postcleithrum	1	20-25	1
LA3	Clarias sp.	vomerine toothplate	1	50-60	1
LA3	Clarias sp.	cranial roof fragment	2	30-40	2
LA3	Clarias sp.	cranial roof fragment	4	60-70	4
LA3	Clarias sp.	cleithrum	5		
LA3	Clarias sp.	cranial roof fragment	9		
LA3	Clarias sp.	caudal vertebra	1		
LA3	Clarias sp.	ceratohyale	1	60-70	1
LA3	Clarias sp.	cleithrum	1	30-40	1
LA3	Clarias sp.	cleithrum	1	50-60	1
LA3	Clarias sp.	cleithrum	1	50-60	1
LA3	Clarias sp.	coracoid	1	20-30	1
LA3	Clarias sp.	coracoid	1	50-60	1
LA3	Clarias sp.	coracoid	1		
LA3	Clarias sp.	dentary	1	40-50	1
LA3	Clarias sp.	dentary	1		
LA3	Clarias sp.	hyomandibula	1	30-40	1
LA3	Clarias sp.	hyomandibula	1	50-60	1

cultural phase	taxon	skeletal element	NISP	SL in cm	number
LA3	Clarias sp.	cranial roof fragment	1	30-40	1
LA3	Clarias sp.	cranial roof fragment	1	50-60	1
LA3	Clarias sp.	palatinum	1	50-60	1
LA3	Clarias sp.	pectoral spine	1	50-60	1
LA3	Clarias sp.	pectoral spine	1		
LA3	Clarias sp.	premaxilla	1	60-70	1
LA3	Clarias sp.	urohyale	1	60-70	1
LA3	Clarias sp.	cranial roof fragment	16		
LA3	Clarias sp.	cranial roof fragment	2	60-70	2
LA3	Clarias sp.	cleithrum	3		
LA3	Clarias sp.	precaudal vertebra	4	60-70	4
LA3	Clarias sp.	caudal vertebra	5	40-50	5
LA3	Clarias sp.	caudal vertebra	9	60-70	9
LA3	Clarias sp.	articular	1	60-70	1
LA3	Clarias sp.	pectoral spine	1	30-40	1
LA3	Clarias sp.	caudal vertebra	1	50-60	1
LA3	Clarias sp.	cleithrum	1		
LA3	Clarias sp.	coracoid	1		
LA3	Clarias sp.	precaudal vertebra	1	60-70	1
LA3	Clarias sp.	premaxilla	1	50-60	1
LA3	Clarias sp.	cranial roof fragment	10		
LA3	Haplotilapiini indet.	opercular	1		
LA3	Haplotilapiini indet.	precaudal vertebra	1	15-20	1
LA3	Haplotilapiini indet.	caudal vertebra	1	15-20	1
LA3	O. niloticus	opercular	1	20-25	1
LA3	Haplotilapiini indet.	parasphenoid	1	15-20	1
LA3	O. niloticus	cleithrum	2		
LA3	Haplotilapiini indet.	basioccipital	1	20-25	1
LA3	Haplotilapiini indet.	basipterygium	1	15-20	1
LA3	O. niloticus	hyomandibula	1	20-25	1
LA3	Haplotilapiini indet.	fin spine	1		
LA3	O. niloticus	opercular	1	20-25	1
LA3	Haplotilapiini indet.	precaudal vertebra	1	20-25	1
LA3	Haplotilapiini indet.	posttemporal	1	25-30	1
LA3	Haplotilapiini indet.	skull roof fragment	2		
LA3	Haplotilapiini indet.	cleithrum	4		
LA3	Haplotilapiini indet.	basioccipital	1	20-25	1
LA3	Haplotilapiini indet.	skull roof fragment	1		
LA3	Haplotilapiini indet.	dorsal or anal pterygophore	1	20-25	1
LA3	Haplotilapiini indet.	dorsal or anal pterygophore	1		
LA3	Haplotilapiini indet.	skull roof fragment	2	20-25	1
LA3	Haplotilapiini indet.	caudal vertebra	1	25-30	1
LA3	Haplotilapiini indet.	fin spine	1		
LA3	Haplotilapiini indet.	skull roof fragment	1		
LA3	Haplotilapiini indet.	opercular	1	15-20	1
LA3	Haplotilapiini indet.	opercular	1	20-25	1
LA3	Haplotilapiini indet.	opercular	1		
LA3	Haplotilapiini indet.	precaudal vertebra	1	15-20	1
LA3	Haplotilapiini indet.	precaudal vertebra	1	20-25	1
LA3	Haplotilapiini indet.	precaudal vertebra	1	25-30	1
LA3	Haplotilapiini indet.	postcleithrum	1		
LA3	Haplotilapiini indet.	dorsal or anal pterygophore	1		
LA3	Haplotilapiini indet.	subopercular	1	20-25	1
LA3	Haplotilapiini indet.	cleithrum	2		
LA3	Haplotilapiini indet.	caudal vertebra	3	15-20	3
LA3	Haplotilapiini indet.	palatinum	1	25-30	1
LA3	Haplotilapiini indet.	caudal vertebra	1	20-25	1
LA3	Haplotilapiini indet.	postcleithrum	1		

cultural phase	taxon	skeletal element	NISP	SL in cm	number
LA3	Haplotilapiini indet.	dorsal or anal pterygophore	1	15-20	1
LA3	Haplotilapiini indet.	dorsal or anal pterygophore	1	20-25	1
LA3	Clarias sp.	articular	1	50-60	1
LA3	Clarias sp.	caudal vertebra	1	60-70	1
LA3	Clarias sp.	palatinum	1		
LA3	Clarias sp.	precaudal vertebra	1	60-70	1
LA3	Clarias sp.	pectoral spine	1	50-60	1
LA3	Clarias sp.	pectoral spine	1		
LA3	Clarias sp.	vomerine toothplate	1	60-70	1
LA3	Clarias sp.	cranial roof fragment	4		
LA3	Clarias sp.	ceratohyale	1	50-60	1
LA3	Clarias sp.	parasphenoid	1	40-50	1
LA3	Clarias sp.	parasphenoid	1	50-60	1
LA3	Clarias sp.	caudal vertebra	1	50-60	1
LA3	Clarias sp.	hyomandibula	1	40-50	1
LA3	Clarias sp.	mesethmoid	1	60-70	1
LA3	Clarias sp.	cranial roof fragment	1	70-80	1
LA3	Clarias sp.	quadrate	1	40-50	1
LA3	Clarias sp.	vomerine toothplate	1	50-60	1
LA3	Clarias sp.	cranial roof fragment	7		
LA3	Clarias sp.	articular	1	40-50	1
LA3	Clarias sp.	caudal vertebra	1	30-40	1
LA3	Clarias sp.	ceratohyale	1	50-60	1
LA3	Clarias sp.	coracoid	1	50-60	1
LA3	Clarias sp.	cranial roof fragment	1	30-40	1
LA3	Clarias sp.	cranial roof fragment	1	70-80	1
LA3	Clarias sp.	vomerine toothplate	1	50-60	1
LA3	Clarias sp.	caudal vertebra	2	50-60	2
LA3	Clarias sp.	cleithrum	2		
LA3	Clarias sp.	cranial roof fragment	2	50-60	2
LA3	Clarias sp.	cranial roof fragment	5		
LA3	Clarias sp.	basioccipital	1	40-50	1
LA3	Clarias sp.	ceratohyale	1	60-70	1
LA3	Clarias sp.	cleithrum	1	50-60	1
LA3	Clarias sp.	cleithrum	1	70-80	1
LA3	Clarias sp.	hypohyale	1	60-70	1
LA3	Clarias sp.	cranial roof fragment	1	20-30	1
LA3	Clarias sp.	cranial roof fragment	1	30-40	1
LA3	Clarias sp.	cranial roof fragment	1	60-70	1
LA3	Clarias sp.	cranial roof fragment	1		
LA3	Clarias sp.	caudal vertebra	2	60-70	2
LA3	Clarias sp.	articular	1	30-40	1
LA3	Clarias sp.	cranial roof fragment	2		
LA3	Clarias sp.	hyomandibula	1	40-50	1
LA3	Clarias sp.	hyomandibula	1	60-70	1
LA3	Clarias sp.	cranial roof fragment	1	60-70	1
LA3	Clarias sp.	pectoral spine	1	50-60	1
LA3	Clarias sp.	quadrate	1	50-60	1
LA3	Clarias sp.	vomerine toothplate	1	50-60	1
LA3	Clarias sp.	cranial roof fragment	2	40-50	2
LA3	Clarias sp.	cranial roof fragment	2	50-60	2
LA3	Clarias sp.	cranial roof fragment	9		
LA3	Clarias sp.	articular	1	60-70	1
LA3	Clarias sp.	cleithrum	1	60-70	1
LA3	Clarias sp.	cleithrum	1		
LA3	Clarias sp.	dentary	1	30-40	1
LA3	Clarias sp.	hyomandibula	1		
LA3	Clarias sp.	unidentified	1		

cultural phase	taxon	skeletal element	NISP	SL in cm	number
LA3	Clarias sp.	branchial element	1		
LA3	Clarias sp.	cranial roof fragment	1	30-40	1
LA3	Clarias sp.	palatinum	1	50-60	1
LA3	Clarias sp.	pectoral spine	1	50-60	1
LA3	Clarias sp.	quadrate	1	60-70	1
LA3	Clarias sp.	cranial roof fragment	2	60-70	2
LA3	Clarias sp.	cranial roof fragment	2		
LA3	Clarias sp.	precaudal vertebra	2	60-70	2
LA3	Clarias sp.	cranial roof fragment	3	50-60	3
LA3	Clarias sp.	caudal vertebra	1	50-60	1
LA3	Clarias sp.	coracoid	1	40-50	1
LA3	Clarias sp.	cranial roof fragment	6		
LA3	Clarias sp.	epihyale	1	60-70	1
LA3	Clarias sp.	branchial element	1		
LA3	Clarias sp.	mesethmoid	1	60-70	1
LA3	Clarias sp.	premaxilla	1		
LA3	Clarias sp.	quadrate	1	30-40	1
LA3	Clarias sp.	quadrate	1	50-60	1
LA3	Clarias sp.	cranial roof fragment	11	50-60	1
LA3	Clarias sp.	caudal vertebra	3	40-50	3
LA3	Clarias sp.	caudal vertebra	5	50-60	5
LA3	Clarias sp.	caudal vertebra	5	60-70	5
LA3	Clarias sp.	Weberian apparatus	1	20-30	1
LA3	Clarias sp.	ceratohyale	1	60-70	1
LA3	Clarias sp.	cleithrum	1	20-30	1
LA3	Clarias sp.	dentary	1	30-40	1
LA3	Clarias sp.	dentary	1	40-50	1
LA3	Clarias sp.	hyomandibula	1		
LA3	Clarias sp.	opercular	1	30-40	1
LA3	Clarias sp.	palatinum	1		
LA3	Clarias sp.	precaudal vertebra	1	60-70	1
LA3	Clarias sp.	pectoral spine	1	60-70	1
LA3	Clarias sp.	premaxilla	1	50-60	1
LA3	Clarias sp.	quadrate	1	40-50	1
LA3	Clarias sp.	quadrate	1	40-50	1
LA3	Clarias sp.	quadrate	1	50-60	1
LA3	Clarias sp.	cranial roof fragment	18		
LA3	Clarias sp.	coracoid	2		
LA3	Clarias sp.	caudal vertebra	3	40-50	3
LA3	Clarias sp.	caudal vertebra	4	50-60	4
LA3	Clarias sp.	cleithrum	4		
LA3	Clarias sp.	cranial roof fragment	4	50-60	4
LA3	Clarias sp.	cranial roof fragment	5	40-50	5
LA3	Clarias sp.	caudal vertebra	7	60-70	7
LA3	Haplotilapiini indet.	cleithrum	1		
LA3	Haplotilapiini indet.	fin spine	1		
LA3	Haplotilapiini indet.	skull roof fragment	1		
LA3	Haplotilapiini indet.	opercular	1	20-25	1
LA3	Haplotilapiini indet.	opercular	1		
LA3	Haplotilapiini indet.	preopercular	1	20-25	1
LA3	Haplotilapiini indet.	dorsal or anal pterygophore	1	20-25	1
LA3	Haplotilapiini indet.	dorsal or anal pterygophore	1		
LA3	Haplotilapiini indet.	precaudal vertebra	1	20-25	1
LA3	Haplotilapiini indet.	dorsal or anal pterygophore	1		
LA3	Haplotilapiini indet.	opercular	1	15-20	1
LA3	Haplotilapiini indet.	fin spine	1		
LA3	Haplotilapiini indet.	dorsal or anal pterygophore	1	15-20	1
LA3	Haplotilapiini indet.	basipterygium	1	20-25	1

cultural phase	taxon	skeletal element	NISP	SL in cm	number
LA3	Haplotilapiini indet.	cleithrum	1		
LA3	O. niloticus	hyomandibula	1	15-20	1
LA3	O. niloticus	hyomandibula	1	15-20	1
LA3	Haplotilapiini indet.	skull roof fragment	1		
LA3	Haplotilapiini indet.	precaudal vertebra	1	20-25	1
LA3	Haplotilapiini indet.	preopercular	1	25-30	1
LA3	Haplotilapiini indet.	dorsal or anal pterygophore	1		
LA3	Haplotilapiini indet.	subopercular	1	20-25	1
LA3	Haplotilapiini indet.	precaudal vertebra	2	15-20	1
LA3	Haplotilapiini indet.	basioccipital	1	15-20	1
LA3	Haplotilapiini indet.	cleithrum	1		
LA3	Haplotilapiini indet.	epihyale	1	20-25	1
LA3	Haplotilapiini indet.	interopercular	1		
LA3	Haplotilapiini indet.	opercular	1	15-20	1
LA3	O. niloticus	1st precaudal vertebra	1	20-25	1
LA3	Haplotilapiini indet.	precaudal vertebra	1	15-20	1
LA3	Haplotilapiini indet.	preopercular	1		
LA3	Haplotilapiini indet.	dorsal or anal pterygophore	1	15-20	1
LA3	Haplotilapiini indet.	urohyale	1	15-20	1
LA3	Haplotilapiini indet.	skull roof fragment	2		
LA3	Haplotilapiini indet.	1st precaudal vertebra	1	15-20	1
LA3	Clarias sp.	caudal vertebra	1	70-80	1
LA3	Clarias sp.	cleithrum	1		
LA3	Clarias sp.	dentary	1	50-60	1
LA3	Clarias sp.	vomerine toothplate	1	40-50	1
LA3	Clarias sp.	cranial roof fragment	2		
LA3	Haplotilapiini indet.	fin spine	1		
LA3	Clarias sp.	cranial roof fragment	2		
LA3	Clarias sp.	cranial roof fragment	2		
LA3	Clarias sp.	precaudal vertebra	2	60-70	1
LA3	Clarias sp.	articular	1	40-50	1
LA3	Clarias sp.	articular	1	50-60	1
LA3	Clarias sp.	cranial roof fragment	1		
LA3	Haplotilapiini indet.	skull roof fragment	1		
LA3	Clarias sp.	dentary	1	40-50	1
LA3	Clarias sp.	cranial roof fragment	1		
LA3	Clarias sp.	precaudal vertebra	1	40-50	1
LA3	Clarias sp.	pectoral spine	1	70-80	1
LA3	Clarias sp.	pectoral spine	1		
LA3	Clarias sp.	caudal vertebra	3	60-70	3
LA3	Clarias sp.	cleithrum	1		
LA3	Clarias sp.	cranial roof fragment	1	70-80	1
LA3	Clarias sp.	pectoral spine	1	50-60	1
LA3	Clarias sp.	precaudal vertebra	1	60-70	1
LA3	Clarias sp.	quadrate	1	50-60	1
LA3	Clarias sp.	cranial roof fragment	4		
LA3	Clarias sp.	caudal vertebra	5	60-70	5
LA3	Haplotilapiini indet.	skull roof fragment	1		
LA3	Clarias sp.	caudal vertebra	1	50-60	1
LA3	Clarias sp.	ceratohyale	1	40-50	1
LA3	Clarias sp.	cranial roof fragment	1		
LA3	Clarias sp.	precaudal vertebra	1	50-60	1
LA3	Clarias sp.	pectoral spine	1	50-60	1
LA3	Haplotilapiini indet.	dentary	1	15-20	1
LA3	O. niloticus	premaxilla	1	25-30	1
LA3	Clarias sp.	cleithrum	1		
LA3	Clarias sp.	cranial roof fragment	2		
LA3	Clarias sp.	articular	1	40-50	1

cultural phase	taxon	skeletal element	NISP	SL in cm	number
LA3	Clarias sp.	articular	1	50-60	1
LA3	Clarias sp.	articular	1	50-60	1
LA3	Clarias sp.	articular	1	50-60	1
LA3	Clarias sp.	caudal vertebra	1	60-70	1
LA3	Clarias sp.	ceratohyale	1		
LA3	Clarias sp.	cleithrum	1	40-50	1
LA3	Clarias sp.	mesethmoid	1	50-60	1
LA3	Clarias sp.	cranial roof fragment	1	40-50	1
LA3	Clarias sp.	opercular	1	40-50	1
LA3	Clarias sp.	opercular	1	40-50	1
LA3	Clarias sp.	precaudal vertebra	1	30-40	1
LA3	Clarias sp.	precaudal vertebra	1	50-60	1
LA3	Clarias sp.	pectoral spine	1	40-50	1
LA3	Clarias sp.	pectoral spine	1	40-50	1
LA3	Clarias sp.	pectoral spine	1	50-60	1
LA3	Clarias sp.	pectoral spine	1	60-70	1
LA3	Clarias sp.	pectoral spine	1		
LA3	Clarias sp.	vomerine toothplate	1	50-60	1
LA3	Clarias sp.	urohyale	1	40-50	1
LA3	Clarias sp.	urohyale	1	50-60	1
LA3	Clarias sp.	vomerine toothplate	1		
LA3	Clarias sp.	coracoid	2		
LA3	Clarias sp.	precaudal vertebra	2	60-70	2
LA3	Clarias sp.	caudal vertebra	3	50-60	3
LA3	Clarias sp.	cleithrum	3		
LA3	Clarias sp.	cranial roof fragment	3	50-60	3
LA3	Clarias sp.	cranial roof fragment	5	60-70	5
LA3	Clarias sp.	cranial roof fragment	8		
LA3	Haplotilapiini indet.	basioccipital	1	25-30	1
LA3	Haplotilapiini indet.	ceratohyale	1		
LA3	O. niloticus	hyomandibula	1	20-25	1
LA3	Haplotilapiini indet.	hyomandibula	1	20-25	1
LA3	Haplotilapiini indet.	hyomandibula	1	20-25	1
LA3	Haplotilapiini indet.	hyomandibula	1	15-20	1
LA3	Haplotilapiini indet.	hyomandibula	1	20-25	1
LA3	Haplotilapiini indet.	opercular	1	20-25	1
LA3	Haplotilapiini indet.	opercular	1	20-25	1
LA3	Haplotilapiini indet.	opercular	1	20-25	1
LA3	Haplotilapiini indet.	opercular	1	20-25	1
LA3	Haplotilapiini indet.	opercular	1	20-25	1
LA3	Haplotilapiini indet.	opercular	1	20-25	1
LA3	Haplotilapiini indet.	opercular	1	20-25	1
LA3	Haplotilapiini indet.	opercular	1	20-25	1
LA3	Haplotilapiini indet.	opercular	1	20-25	1
LA3	Haplotilapiini indet.	opercular	1	20-25	1
LA3	Haplotilapiini indet.	opercular	1	15-20	1
LA3	Haplotilapiini indet.	opercular	1	15-20	1
LA3	Haplotilapiini indet.	opercular	1	15-20	1
LA3	Haplotilapiini indet.	opercular	1	20-25	1
LA3	Haplotilapiini indet.	opercular	1	20-25	1
LA3	Haplotilapiini indet.	opercular	1	20-25	1
LA3	Haplotilapiini indet.	opercular	1	20-25	1
LA3	Haplotilapiini indet.	opercular	1	20-25	1
LA3	Haplotilapiini indet.	1st precaudal vertebra	1	20-25	1
LA3	Haplotilapiini indet.	1st precaudal vertebra	1	20-25	1
LA3	Haplotilapiini indet.	2nd precaudal vertebra	1	20-25	1
LA3	Haplotilapiini indet.	2nd precaudal vertebra	1	15-20	1
LA3	Haplotilapiini indet.	postcleithrum	1	15-20	1

cultural phase	taxon	skeletal element	NISP	SL in cm	number
LA3	Haplotilapiini indet.	posttemporal	1	20-25	1
LA3	Haplotilapiini indet.	dorsal or anal pterygophore	1	15-20	1
LA3	Haplotilapiini indet.	dorsal or anal pterygophore	1	20-25	1
LA3	Haplotilapiini indet.	dorsal or anal pterygophore	1	15-20	1
LA3	Haplotilapiini indet.	dorsal or anal pterygophore	1	15-20	1
LA3	Haplotilapiini indet.	dorsal or anal pterygophore	1	15-20	1
LA3	Haplotilapiini indet.	dorsal or anal pterygophore	1	15-20	1
LA3	Haplotilapiini indet.	dorsal or anal pterygophore	1		
LA3	Haplotilapiini indet.	subopercular	1	20-25	1
LA3	Haplotilapiini indet.	caudal vertebra	12	15-20	12
LA3	Haplotilapiini indet.	skull roof fragment	14		
LA3	Haplotilapiini indet.	cleithrum	17	25-30	1
LA3	Haplotilapiini indet.	supracleithrum	2	15-20	2
LA3	Haplotilapiini indet.	supracleithrum	2	20-25	2
LA3	Haplotilapiini indet.	basipterygium	3	15-20	2
LA3	Haplotilapiini indet.	caudal vertebra	3	20-25	3
LA3	Haplotilapiini indet.	precaudal vertebra	3	20-25	3
LA3	Haplotilapiini indet.	preopercular	4		
LA3	Haplotilapiini indet.	opercular	5		
LA3	Haplotilapiini indet.	lepidotrich	9		
LA3	Haplotilapiini indet.	precaudal vertebra	9	15-20	9
LA3	Clarias sp.	caudal vertebra	1	50-60	1
LA3	Clarias sp.	dentary	1	30-40	1
LA3	Clarias sp.	cranial roof fragment	1	30-40	1
LA3	Clarias sp.	cranial roof fragment	1		
LA3	Clarias sp.	opercular	1	15-20	1
LA3	Clarias sp.	opercular	1		
LA3	Clarias sp.	quadrate	1	50-60	1
LA3	Clarias sp.	cranial roof fragment	15		
LA3	Clarias sp.	caudal vertebra	2	40-50	2
LA3	Clarias sp.	cleithrum	2		
LA3	Clarias sp.	coracoid	2		
LA3	Clarias sp.	cranial roof fragment	2	40-50	2
LA3	Haplotilapiini indet.	dorsal or anal pterygophore	1	20-25	1
LA3	Haplotilapiini indet.	dorsal or anal pterygophore	1	20-25	1
LA3	Haplotilapiini indet.	dorsal or anal pterygophore	1	20-25	1
LA3	Haplotilapiini indet.	cleithrum	10		
LA3	Clarias sp.	ceratohyale	1	30-40	1
LA3	Clarias sp.	cleithrum	1	30-40	1
LA3	Clarias sp.	pectoral spine	1	60-70	1
LA3	Clarias sp.	cranial roof fragment	7	50-60	1
LA3	Haplotilapiini indet.	cleithrum	1		
LA3	Haplotilapiini indet.	1st precaudal vertebra	1	20-25	1
LA3	O. niloticus	1st precaudal vertebra	1	20-25	1
LA3	O. niloticus	1st precaudal vertebra	1	25-30	1
LA3	Clarias sp.	articular	1	40-50	1
LA3	Clarias sp.	articular	1	50-60	1
LA3	Clarias sp.	caudal vertebra	1	50-60	1
LA3	Clarias sp.	caudal vertebra	1	60-70	1
LA3	Clarias sp.	cleithrum	1	20-30	1
LA3	Clarias sp.	cleithrum	1	40-50	1
LA3	Clarias sp.	cranial roof fragment	1	60-70	1
LA3	Clarias sp.	precaudal vertebra	1	50-60	1
LA3	Clarias sp.	precaudal vertebra	1	60-70	1
LA3	Clarias sp.	pectoral spine	1	30-40	1
LA3	Clarias sp.	pectoral spine	1	50-60	1
LA3	Clarias sp.	pectoral spine	1	30-40	1
LA3	Clarias sp.	quadrate	1	50-60	1

cultural phase	taxon	skeletal element	NISP	SL in cm	number
LA3	Clarias sp.	vomerine toothplate	1		
LA3	Clarias sp.	coracoid	2		
LA3	Clarias sp.	cranial roof fragment	2	40-50	2
LA3	Clarias sp.	cleithrum	6		
LA3	Clarias sp.	cranial roof fragment	7		
LA3	Haplotilapiini indet.	basipterygium	1	20-25	1
LA3	Haplotilapiini indet.	caudal vertebra	1	10-15	1
LA3	Haplotilapiini indet.	fin spine	1		
LA3	Haplotilapiini indet.	skull roof fragment	1		
LA3	Haplotilapiini indet.	opercular	1	20-25	1
LA3	Haplotilapiini indet.	2nd precaudal vertebra	1	20-25	1
LA3	Haplotilapiini indet.	dorsal or anal pterygophore	1	20-25	1
LA3	Haplotilapiini indet.	cleithrum	5		
LA3	Haplotilapiini indet.	precaudal vertebra	5	10-15	4
LA3	Clarias sp.	caudal vertebra	1	50-60	1
LA3	Clarias sp.	cleithrum	1	60-70	1
LA3	Clarias sp.	pectoral spine	1	40-50	1
LA3	Clarias sp.	pectoral spine	1	60-70	1
LA3	Clarias sp.	cranial roof fragment	3	40-50	3
LA3	Clarias sp.	cranial roof fragment	4		
LA3	Clarias sp.	pectoral spine	1	60-70	1
LA3	Clarias sp.	caudal vertebra	1	40-50	1
LA3	Haplotilapiini indet.	basipterygium	1	15-20	1
LA3	Haplotilapiini indet.	hyomandibula	1	20-25	1
LA3	Haplotilapiini indet.	opercular	1	15-20	1
LA3	Haplotilapiini indet.	opercular	1	20-25	1
LA3	Haplotilapiini indet.	opercular	1	20-25	1
LA3	Haplotilapiini indet.	opercular	1	20-25	1
LA3	Haplotilapiini indet.	opercular	1	20-25	1
LA3	Haplotilapiini indet.	preopercular	1		
LA3	Haplotilapiini indet.	postcleithrum	1	15-20	1
LA3	Haplotilapiini indet.	dorsal or anal pterygophore	1	15-20	1
LA3	Haplotilapiini indet.	dorsal or anal pterygophore	1	15-20	1
LA3	Haplotilapiini indet.	lepidotrich	2		
LA3	Haplotilapiini indet.	skull roof fragment	2	20-25	2
LA3	Haplotilapiini indet.	skull roof fragment	2		
LA3	Haplotilapiini indet.	precaudal vertebra	2	15-20	2
LA3	Haplotilapiini indet.	skull roof fragment	3	15-20	3
LA3	O. niloticus	cleithrum	6	20-25	2
LA3	Clarias sp.	articular	1	40-50	1
LA3	Clarias sp.	articular	1	40-50	1
LA3	Clarias sp.	articular	1	50-60	1
LA3	Clarias sp.	articular	1	60-70	1
LA3	Clarias sp.	basioccipital	1	40-50	1
LA3	Clarias sp.	ceratohyale	1	60-70	1
LA3	Clarias sp.	cleithrum	1	40-50	1
LA3	Clarias sp.	cleithrum	1	40-50	1
LA3	Clarias sp.	cleithrum	1	40-50	1
LA3	Clarias sp.	epihyale	1	50-60	1
LA3	Clarias sp.	mesethmoid	1	50-60	1
LA3	Clarias sp.	cranial roof fragment	1	60-70	1
LA3	Clarias sp.	opercular	1	50-60	1
LA3	Clarias sp.	palatinum	1	50-60	1
LA3	Clarias sp.	pectoral spine	1	50-60	1
LA3	Clarias sp.	pectoral spine	1	60-70	1
LA3	Clarias sp.	pectoral spine	1	70-80	1
LA3	Clarias sp.	pectoral spine	1		
LA3	Clarias sp.	quadrate	1	60-70	1

cultural phase	taxon	skeletal element	NISP	SL in cm	number
LA3	Clarias sp.	urohyale	1	60-70	1
LA3	Clarias sp.	cleithrum	12		
LA3	Clarias sp.	cranial roof fragment	16		
LA3	Clarias sp.	cranial roof fragment	2	70-80	2
LA3	Clarias sp.	caudal vertebra	3	40-50	3
LA3	Clarias sp.	caudal vertebra	3	60-70	3
LA3	Clarias sp.	precaudal vertebra	3	60-70	3
LA3	Clarias sp.	caudal vertebra	8	50-60	8
LA3	Haplotilapiini indet.	caudal vertebra	1	20-25	1
LA3	Haplotilapiini indet.	3rd precaudal vertebra	1	25-30	1
LA3	Haplotilapiini indet.	preopercular	1	20-25	1
LA3	Haplotilapiini indet.	dorsal or anal pterygophore	1	20-25	1
LA3	Haplotilapiini indet.	dorsal or anal pterygophore	1	20-25	1
LA3	Haplotilapiini indet.	cleithrum	3		
LA3	Haplotilapiini indet.	precaudal vertebra	5	15-20	5
LA3	Haplotilapiini indet.	caudal vertebra	6	15-20	6
LA3	Clarias sp.	caudal vertebra	1	70-80	1
LA3	Clarias sp.	ceratohyale	1	30-40	1
LA3	Clarias sp.	hyomandibula	1		
LA3	Clarias sp.	cranial roof fragment	5		
LA3	Haplotilapiini indet.	cleithrum	1		
LA3	Haplotilapiini indet.	opercular	1	20-25	1
LA3	Clarias sp.	cleithrum	1		
LA3	Clarias sp.	opercular	1	70-80	1
LA3	Clarias sp.	cranial roof fragment	2		
LA3	Clarias sp.	caudal vertebra	3	60-70	3
LA3	Clarias sp.	articular	1	50-60	1
LA3	Clarias sp.	cranial roof fragment	1	50-60	1
LA3	Clarias sp.	caudal vertebra	1	60-70	1
LA3	Clarias sp.	cleithrum	1	30-40	1
LA3	Clarias sp.	coracoid	1		
LA3	Clarias sp.	mesethmoid	1	60-70	1
LA3	Clarias sp.	cranial roof fragment	1		
LA3	Clarias sp.	pectoral spine	1	40-50	1
LA3	Clarias sp.	cranial roof fragment	5	30-40	1
LA3	Clarias sp.	articular	1	50-60	1
LA3	Clarias sp.	caudal vertebra	1		
LA3	Clarias sp.	ceratohyale	1	50-60	1
LA3	Clarias sp.	cleithrum	1	50-60	1
LA3	Clarias sp.	cleithrum	1		
LA3	Clarias sp.	coracoid	1	40-50	1
LA3	Clarias sp.	epihyale	1	50-60	1
LA3	Clarias sp.	mesethmoid	1	50-60	1
LA3	Clarias sp.	cranial roof fragment	1		
LA3	Clarias sp.	opercular	1	40-50	1
LA3	Clarias sp.	precaudal vertebra	1	40-50	1
LA3	Clarias sp.	precaudal vertebra	1	50-60	1
LA3	Clarias sp.	precaudal vertebra	1	60-70	1
LA3	Clarias sp.	caudal vertebra	4	60-70	4
LA3	Clarias sp.	cranial roof fragment	9		
LA3	Clarias sp.	caudal vertebra	1	50-60	1
LA3	Clarias sp.	cleithrum	1	40-50	1
LA3	Clarias sp.	cleithrum	1	60-70	1
LA3	Clarias sp.	coracoid	1	40-50	1
LA3	Clarias sp.	cranial roof fragment	3	40-50	3
LA3	Clarias sp.	basioccipital	1	60-70	1
LA3	Clarias sp.	ceratohyale	1	40-50	1
LA3	Clarias sp.	pectoral spine	1		

cultural phase	taxon	skeletal element	NISP	SL in cm	number
LA3	Clarias sp.	cranial roof fragment	2		
LA3	Clarias sp.	cranial roof fragment	2		
LA3	Haplotilapiini indet.	opercular	1		
LA3	Haplotilapiini indet.	cleithrum	1		
LA3	Haplotilapiini indet.	skull roof fragment	1	20-25	1
LA3	Haplotilapiini indet.	opercular	1		
LA3	Haplotilapiini indet.	skull roof fragment	2		
LA3	Haplotilapiini indet.	cleithrum	1		
LA3	Clarias sp.	articular	1	50-60	1
LA3	Clarias sp.	basioccipital	1		
LA3	Clarias sp.	cleithrum	1	80-90	1
LA3	Clarias sp.	cleithrum	1	50-60	1
LA3	Clarias sp.	cranial roof fragment	1	60-70	1
LA3	Clarias sp.	cranial roof fragment	2		
LA3	Clarias sp.	caudal vertebra	1	50-60	1
LA3	Clarias sp.	dentary	1	50-60	1
LA3	Clarias sp.	hyomandibula	1		
LA3	Clarias sp.	cranial roof fragment	1	30-40	1
LA3	Clarias sp.	pectoral spine	1	30-40	1
LA3	Clarias sp.	articular	1	70-80	1
LA3	Clarias sp.	articular	1	40-50	1
LA3	Clarias sp.	articular	1	40-50	1
LA3	Clarias sp.	caudal vertebra	1	60-70	1
LA3	Clarias sp.	ceratohyale	1	40-50	1
LA3	Clarias sp.	dentary	1	30-40	1
LA3	Clarias sp.	dentary	1	40-50	1
LA3	Clarias sp.	dentary	1	70-80	1
LA3	Clarias sp.	epihyale	1	40-50	1
LA3	Clarias sp.	cranial roof fragment	1	60-70	1
LA3	Clarias sp.	opercular	1	60-70	1
LA3	Clarias sp.	palatinum	1	50-60	1
LA3	Clarias sp.	pectoral spine	1	40-50	1
LA3	Clarias sp.	urohyale	1	60-70	1
LA3	Clarias sp.	cranial roof fragment	11		
LA3	Clarias sp.	coracoid	2	30-40	1
LA3	Clarias sp.	precaudal vertebra	2	60-70	2
LA3	Clarias sp.	coracoid	1	60-70	1
LA3	Clarias sp.	parasphenoid	1		
LA3	Clarias sp.	cranial roof fragment	2	50-60	2
LA3	Haplotilapiini indet.	mesethmoid	1	20-25	1
LA3	Haplotilapiini indet.	precaudal vertebra	1	20-25	1
LA3	Haplotilapiini indet.	dorsal or anal pterygophore	1	15-20	1
LA3	Clarias sp.	caudal vertebra	1	60-70	1
LA3	Clarias sp.	articular	1	40-50	1
LA3	Clarias sp.	ceratohyale	1	40-50	1
LA3	Clarias sp.	cleithrum	1	40-50	1
LA3	Clarias sp.	coracoid	1	40-50	1
LA3	Clarias sp.	cranial roof fragment	1	50-60	1
LA3	Clarias sp.	opercular	1	40-50	1
LA3	Clarias sp.	opercular	1	40-50	1
LA3	Clarias sp.	precaudal vertebra	1	50-60	1
LA3	Clarias sp.	pectoral spine	1	60-70	1
LA3	Clarias sp.	quadrate	1	40-50	1
LA3	Clarias sp.	vomerine toothplate	1	60-70	1
LA3	Clarias sp.	caudal vertebra	4	60-70	4
LA3	Clarias sp.	cranial roof fragment	9		
LA3	Haplotilapiini indet.	postcleithrum	1	20-25	1
LA3	O. niloticus	urohyale	1		

cultural phase	taxon	skeletal element	NISP	SL in cm	number
LA3	Clarias sp.	cleithrum	1	30-40	1
LA3	Clarias sp.	dentary	1	50-60	1
LA3	Clarias sp.	unidentified	1		
LA3	Clarias sp.	cranial roof fragment	5	50-60	1
LA3	Clarias sp.	caudal vertebra	1	50-60	1
LA3	Clarias sp.	cleithrum	1	50-60	1
LA3	Clarias sp.	coracoid	1		
LA3	Clarias sp.	caudal vertebra	2	60-70	2
LA3	Clarias sp.	cranial roof fragment	4	60-70	2
LA3	Clarias sp.	cranial roof fragment	4		
LA3	Clarias sp.	caudal vertebra	1	60-70	1
LA3	Clarias sp.	epihyale	1	40-50	1
LA3	Clarias sp.	hyomandibula	1	60-70	1
LA3	Clarias sp.	pectoral spine	1	50-60	1
LA3	Clarias sp.	cranial roof fragment	3	70-80	1
LA3	Clarias sp.	caudal vertebra	1	60-70	1
LA3	Clarias sp.	quadrate	1	50-60	1
LA3	Clarias sp.	cranial roof fragment	6	60-70	1
LA3	Clarias sp.	cranial roof fragment	1		
LA3	Haplotilapiini indet.	cleithrum	1		
LA3	Haplotilapiini indet.	dorsal or anal pterygophore	1	25-30	1
LA3	Clarias sp.	caudal vertebra	1	60-70	1
LA3	Clarias sp.	articular	1	60-70	1
LA3	Clarias sp.	caudal vertebra	1	30-40	1
LA3	Clarias sp.	caudal vertebra	1	50-60	1
LA3	Clarias sp.	ceratohyale	1	30-40	1
LA3	Clarias sp.	ceratohyale	1	50-60	1
LA3	Clarias sp.	dentary	1		
LA3	Clarias sp.	cranial roof fragment	1	50-60	1
LA3	Clarias sp.	opercular	1	30-40	1
LA3	Clarias sp.	pectoral spine	1		
LA3	Clarias sp.	quadrate	1	30-40	1
LA3	Clarias sp.	quadrate	1	40-50	1
LA3	Clarias sp.	cranial roof fragment	11		
LA3	Clarias sp.	cranial roof fragment	2	60-70	2
LA3	Haplotilapiini indet.	caudal vertebra	1	20-25	1
LA3	Haplotilapiini indet.	2nd precaudal vertebra	1	20-25	1
LA3	Haplotilapiini indet.	precaudal vertebra	1	15-20	1
LA3	Haplotilapiini indet.	precaudal vertebra	1	20-25	1
LA3	Haplotilapiini indet.	postcleithrum	1	20-25	1
LA3	Haplotilapiini indet.	cleithrum	2		
EP1	Clarias sp.	cleithrum	1	40-50	1
EP1	O. niloticus	1st precaudal vertebra	1	20-25	1
EP1	Clarias sp.	ceratohyale	1	70-80	1
EP1	Clarias sp.	cleithrum	1		
EP1	Clarias sp.	coracoid	1		
EP1	Clarias sp.	dentary	1	50-60	1
EP1	Clarias sp.	cranial roof fragment	1		
EP1	Clarias sp.	cranial roof fragment	1	30-40	1
EP1	Clarias sp.	cranial roof fragment	1	40-50	1
EP1	Clarias sp.	cranial roof fragment	1		
EP1	Clarias sp.	mesethmoid	1	60-70	1
EP1	Clarias sp.	mesethmoid	1	60-70	1
EP1	Clarias sp.	opercular	1	40-50	1
EP1	Clarias sp.	precaudal vertebra	1	60-70	1
EP1	Clarias sp.	pectoral spine	1	60-70	1
EP1	Clarias sp.	caudal vertebra	2	60-70	2
EP1	Clarias sp.	caudal vertebra	3	70-80	3

cultural phase	taxon	skeletal element	NISP	SL in cm	number
EP1	Haplotilapiini indet.	cleithrum	1		
EP1	Clarias sp.	articular	1	50-60	1
EP1	Clarias sp.	articular	1		
EP1	Clarias sp.	caudal vertebra	1	60-70	1
EP1	Clarias sp.	pectoral spine	1		
EP1	Clarias sp.	quadrate	1	50-60	1
EP1	Clarias sp.	cranial roof fragment	2	30-40	2
EP1	Clarias sp.	cranial roof fragment	2	60-70	2
EP1	Clarias sp.	caudal vertebra	4	50-60	4
EP1	Clarias sp.	cranial roof fragment	4		
EP1	Clarias sp.	articular	1	50-60	1
EP1	Clarias sp.	caudal vertebra	1	50-60	1
EP1	Clarias sp.	cleithrum	1	30-40	1
EP1	Clarias sp.	dentary	1	50-60	1
EP1	Clarias sp.	cranial roof fragment	1		
EP1	Clarias sp.	cranial roof fragment	1	60-70	1
EP1	Clarias sp.	cranial roof fragment	1		
EP1	Clarias sp.	precaudal vertebra	1	70-80	1
EP1	Clarias sp.	pectoral spine	1	40-50	1
EP1	Clarias sp.	pectoral spine	1		
EP1	Haplotilapiini indet.	skull roof fragment	1		
EP1	Clarias sp.	cleithrum	1	50-60	1
EP1	Clarias sp.	cleithrum	1		
EP1	Clarias sp.	coracoid	1	60-70	1
EP1	Clarias sp.	coracoid	1		
EP1	Clarias sp.	hyomandibula	1	50-60	1
EP1	Clarias sp.	precaudal vertebra	1	50-60	1
EP1	Clarias sp.	pectoral spine	1	60-70	1
EP1	Clarias sp.	pectoral spine	1	60-70	1
EP1	Clarias sp.	quadrate	1	50-60	1
EP1	Clarias sp.	quadrate	1	30-40	1
EP1	Clarias sp.	precaudal vertebra	2	60-70	2
EP1	Clarias sp.	caudal vertebra	3	50-60	3
EP1	Clarias sp.	caudal vertebra	8	60-70	8
EP1	Haplotilapiini indet.	caudal vertebra	1	15-20	1
EP1	Haplotilapiini indet.	skull roof fragment	1		
EP1	Clarias sp.	articular	1	50-60	1
EP1	Clarias sp.	articular	1	50-60	1
EP1	Clarias sp.	articular	1	50-60	1
EP1	Clarias sp.	articular	1	50-60	1
EP1	Clarias sp.	caudal vertebra	1	50-60	1
EP1	Clarias sp.	caudal vertebra	1	60-70	1
EP1	Clarias sp.	cleithrum	1	40-50	1
EP1	Clarias sp.	cleithrum	1	50-60	1
EP1	Clarias sp.	cleithrum	1		
EP1	Clarias sp.	cranial roof fragment	1	60-70	1
EP1	Clarias sp.	cranial roof fragment	2		
EP1	Clarias sp.	cranial roof fragment	1	50-60	1
EP1	Haplotilapiini indet.	postcleithrum	1	20-25	1
EP1	Haplotilapiini indet.	lepidotrich	1		
EP1	Clarias sp.	cranial roof fragment	1	50-60	1
EP1	Clarias sp.	cranial roof fragment	1	60-70	1
EP1	Clarias sp.	mesethmoid	1	50-60	1
EP1	Clarias sp.	cranial roof fragment	4		
EP1	Haplotilapiini indet.	caudal vertebra	1	15-20	1
EP1	Haplotilapiini indet.	opercular	1	15-20	1
EP1	Clarias sp.	pectoral spine	1	70-80	1
EP1	Clarias sp.	Weberian apparatus	1	100-110	1

cultural phase	taxon	skeletal element	NISP	SL in cm	number
EP1	Clarias sp.	epihyale	1	50-60	1
EP1	Clarias sp.	cranial roof fragment	3		
EP1	Haplotilapiini indet.	hyomandibula	1	20-25	1
EP1	Clarias sp.	caudal vertebra	1	30-40	1
EP1	Clarias sp.	branchial element	1		
EP1	Clarias sp.	cranial roof fragment	3		
EP1	Clarias sp.	pectoral spine	1	50-60	1
EP1	Clarias sp.	epi- & ceratohyale	1	60-70	1
EP1	Clarias sp.	precaudal vertebra	1		
EP1	Clarias sp.	pectoral spine	1	50-60	1
EP1	Clarias sp.	cranial roof fragment	2		
EP1	Clarias sp.	caudal vertebra	1	60-70	1
EP1	Clarias sp.	cranial roof fragment	1		
EP1	Clarias sp.	cleithrum	1	40-50	1
EP1	Clarias sp.	cleithrum	1	60-70	1
EP1	Clarias sp.	cleithrum	1		
EP1	Clarias sp.	cranial roof fragment	1		
EP1	Clarias sp.	coracoid	1		
EP1	Clarias sp.	metapterygium	1	70-80	1
EP1	Clarias sp.	cleithrum	2	20-25	1
EP1	Clarias sp.	caudal vertebra	1		
EP1	Clarias sp.	cleithrum	1	50-60	1
EP1	Haplotilapiini indet.	anal pterygophore	1	20-25	1
EP1	Haplotilapiini indet.	fin spine	1		
EP1	Clarias sp.	cranial roof fragment	1		
EP1	Clarias sp.	cleithrum	1	40-50	1
EP1	Clarias sp.	cranial roof fragment	1	50-60	1
EP1	Clarias sp.	cranial roof fragment	3		
EP1	Haplotilapiini indet.	opercular	1	20-25	1
EP1	C. gariepinus	vomerine toothplate	1	40-50	1
EP1	Clarias sp.	cranial roof fragment	1	40-50	1
EP1	Haplotilapiini indet.	cleithrum	1	15-20	1
EP1	Clarias sp.	cranial roof fragment	1		
EP1	Clarias sp.	cranial roof fragment	1	60-70	1
EP1	Haplotilapiini indet.	subopercular	1	20-25	1
EP1	Haplotilapiini indet.	cleithrum	2	20-25	1
EP1	Clarias sp.	cranial roof fragment	1		
EP1	Clarias sp.	parasphenoid	1	20-25	1
EP1	Clarias sp.	cleithrum	2		
EP1	Clarias sp.	cranial roof fragment	2		
EP1	Clarias sp.	caudal vertebra	1	30-40	1
EP1	Clarias sp.	caudal vertebra	1	50-60	1
EP1	Clarias sp.	ceratohyale	1	40-50	1
EP1	Clarias sp.	cleithrum	1	30-40	1
EP1	Clarias sp.	cleithrum	1	40-50	1
EP1	Clarias sp.	cleithrum	1	50-60	1
EP1	Clarias sp.	cleithrum	1		
EP1	Clarias sp.	hyomandibula	1		
EP1	Clarias sp.	cranial roof fragment	2		
EP1	Clarias sp.	cranial roof fragment	2		
EP1	Clarias sp.	cranial roof fragment	20		
EP1	Haplotilapiini indet.	caudal vertebra	1	20-25	1
EP1	Haplotilapiini indet.	cleithrum	1		
EP1	Haplotilapiini indet.	cleithrum	1		
EP1	Haplotilapiini indet.	fin spine	1		
EP1	Haplotilapiini indet.	opercular	1	20-25	1
EP1	Haplotilapiini indet.	3rd precaudal vertebra	1	20-25	1
EP1	Haplotilapiini indet.	premaxilla	1	25-30	1

cultural phase	taxon	skeletal element	NISP	SL in cm	number
EP1	Clarias sp.	dentary	1	70-80	1
EP1	Clarias sp.	cranial roof fragment	1	60-70	1
EP1	Clarias sp.	ceratohyale	1	60-70	1
EP1	Clarias sp.	mesethmoid	1	40-50	1
EP1	Clarias sp.	cranial roof fragment	1	60-70	1
EP1	Clarias sp.	cranial roof fragment	2		
EP1	Clarias sp.	articular	1	40-50	1
EP1	Clarias sp.	caudal vertebra	1	50-60	1
EP1	Clarias sp.	ceratohyale	1		
EP1	Clarias sp.	cleithrum	1	60-70	1
EP1	Clarias sp.	cleithrum	1	40-50	1
EP1	Clarias sp.	epihyale	1	50-60	1
EP1	Clarias sp.	precaudal vertebra	1	50-60	1
EP1	Clarias sp.	cranial roof fragment	2		
EP1	Clarias sp.	cranial roof fragment	2	50-60	1
EP1	Clarias sp.	cranial roof fragment	4		
EP1	Haplotilapiini indet.	skull roof fragment	1		
EP1	Clarias sp.	dentary	1	50-60	1
EP1	Clarias sp.	branchial element	1		
EP1	Clarias sp.	cranial roof fragment	1	40-50	1
EP1	Clarias sp.	cranial roof fragment	3		
EP1	Clarias sp.	articular	1	40-50	1
EP1	Clarias sp.	basipterygium	1		
EP1	Clarias sp.	caudal vertebra	1	40-50	1
EP1	Clarias sp.	caudal vertebra	1	60-70	1
EP1	Clarias sp.	caudal vertebra	1	70-90	1
EP1	Clarias sp.	caudal vertebra	1	30-40	1
EP1	Clarias sp.	cleithrum	1		
EP1	Clarias sp.	cleithrum	1	30-40	1
EP1	Clarias sp.	cleithrum	1		
EP1	Clarias sp.	cleithrum	1	50-60	1
EP1	Clarias sp.	coracoid	1		
EP1	Clarias sp.	hyomandibula	1		
EP1	Clarias sp.	unidentified	1		
EP1	Clarias sp.	mesethmoid	1	50-60	1
EP1	Clarias sp.	cranial roof fragment	1	60-70	1
EP1	Clarias sp.	precaudal vertebra	1		
EP1	Clarias sp.	pectoral spine	1		
EP1	Clarias sp.	preopercular	1	20-25	1
EP1	Clarias sp.	cranial roof fragment	11		
EP1	Clarias sp.	caudal vertebra	2	40-50	1
EP1	Clarias sp.	cranial roof fragment	2		
EP1	Clarias sp.	caudal vertebra	3	50-60	1
EP1	Clarias sp.	cranial roof fragment	3		
EP1	Clarias sp.	cranial roof fragment	4		
EP1	Clarias sp.	cranial roof fragment	8		
EP1	Clarias sp.	cranial roof fragment	8		
EP1	Haplotilapiini indet.	anal pterygophore	1	20-25	1
EP1	Haplotilapiini indet.	anal pterygophore	1	15-20	1
EP1	Haplotilapiini indet.	anal pterygophore	1	15-20	1
EP1	Haplotilapiini indet.	anal pterygophore	1	15-20	1
EP1	Haplotilapiini indet.	anal pterygophore	1		
EP1	Haplotilapiini indet.	basioccipital	1	20-25	1
EP1	Haplotilapiini indet.	caudal vertebra	1	15-20	1
EP1	Haplotilapiini indet.	caudal vertebra	1	20-25	1
EP1	Haplotilapiini indet.	cleithrum	1	15-20	1
EP1	Haplotilapiini indet.	cleithrum	1	20-20	1
EP1	Haplotilapiini indet.	cleithrum	1	20-25	1

cultural phase	taxon	skeletal element	NISP	SL in cm	number
EP1	O. niloticus	hyomandibula	1	20-25	1
EP1	O. niloticus	hyomandibula	1	20-25	1
EP1	Haplotilapiini indet.	maxilla	1	15-20	1
EP1	Haplotilapiini indet.	skull roof fragment	1	20-25	1
EP1	Haplotilapiini indet.	skull roof fragment	1		
EP1	Haplotilapiini indet.	parasphenoid	1	20-25	1
EP1	Haplotilapiini indet.	precaudal vertebra	1	20-25	1
EP1	Haplotilapiini indet.	precaudal vertebra	1	15-20	1
EP1	Haplotilapiini indet.	precaudal vertebra	1	20-25	1
EP1	Haplotilapiini indet.	posttemporal	1	20-25	1
EP1	Haplotilapiini indet.	suborbital	1		
EP1	Haplotilapiini indet.	basipterygium	2		
EP1	Haplotilapiini indet.	cleithrum	2	15-20	1
EP1	Haplotilapiini indet.	ectopterygoid	2	20-25	1
EP1	Haplotilapiini indet.	lepidotrich	2		
EP1	Haplotilapiini indet.	precaudal vertebra	3	15-20	1
EP1	Clarias sp.	cranial roof fragment	1		
EP1	Haplotilapiini indet.	fin spine	1		
EP1	Haplotilapiini indet.	dorsal or anal pterygophore	1		
EP1	Clarias sp.	articular	1	60-70	1
EP1	Clarias sp.	cleithrum	1		
EP1	Clarias sp.	cleithrum	1	60-70	1
EP1	Clarias sp.	coracoid	1	50-60	1
EP1	Clarias sp.	cranial roof fragment	1	40-50	1
EP1	Clarias sp.	cranial roof fragment	1	50-60	1
EP1	Clarias sp.	cranial roof fragment	1	60-70	1
EP1	Clarias sp.	cranial roof fragment	1		
EP1	Clarias sp.	precaudal vertebra	1	40-50	1
EP1	Clarias sp.	caudal vertebra	3	50-60	1
EP1	Clarias sp.	caudal vertebra	3	60-70	1
EP1	Clarias sp.	cranial roof fragment	9		
EP1	Haplotilapiini indet.	basioccipital	1	15-20	1
EP1	Haplotilapiini indet.	caudal vertebra	1	20-25	1
EP1	Haplotilapiini indet.	cleithrum	1	20-25	1
EP1	Haplotilapiini indet.	cleithrum	1		
EP1	Haplotilapiini indet.	mesethmoid	1	20-25	1
EP1	Haplotilapiini indet.	skull roof fragment	1		
EP1	Haplotilapiini indet.	opercular	1	15-20	1
EP1	Haplotilapiini indet.	precaudal vertebra	1	15-20	1
EP1	Haplotilapiini indet.	suborbital	1		
EP1	Haplotilapiini indet.	supracleithrum	1		
EP1	Haplotilapiini indet.	urohyale	1		
EP1	Haplotilapiini indet.	skull roof fragment	2		
EP1	Haplotilapiini indet.	cleithrum	4	15-20	1
EP1	Haplotilapiini indet.	caudal vertebra	6	15-20	1
EP1	Clarias sp.	articular	1	40-50	1
EP1	Clarias sp.	caudal vertebra	1	60-70	1
EP1	Clarias sp.	ceratohyale	1		
EP1	Clarias sp.	cleithrum	1		
EP1	Clarias sp.	epihyale	1		
EP1	Clarias sp.	cranial roof fragment	3		
EP1	Clarias sp.	dentary	1	40-50	1
EP1	Clarias sp.	cranial roof fragment	6		
EP1	Haplotilapiini indet.	caudal vertebra	1	20-25	1
EP1	Haplotilapiini indet.	precaudal vertebra	1	20-25	1
EP1	Clarias sp.	cranial roof fragment	1		
EP1	Clarias sp.	cranial roof fragment	1		
EP1	Haplotilapiini indet.	parasphenoid	1	15-20	1

cultural phase	taxon	skeletal element	NISP	SL in cm	number
EP1	Clarias sp.	caudal vertebra	1	60-70	1
EP1	Clarias sp.	cleithrum	1		
EP1	Clarias sp.	unidentified	1		
EP1	Clarias sp.	precaudal vertebra	1		
EP1	Clarias sp.	pectoral spine	1	40-50	1
EP1	Haplotilapiini indet.	ceratohyale	1	20-25	1
EP1	Haplotilapiini indet.	dentary	1	25-30	1
EP1	Haplotilapiini indet.	opercular	1	15-20	1
EP1	Haplotilapiini indet.	urohyale	1	20-25	1
EP1	Haplotilapiini indet.	supracleithrum	2	20-25	1
EP1	Clarias sp.	cleithrum	1		
EP1	Clarias sp.	pectoral spine	1	60-70	1
EP1	Clarias sp.	caudal vertebra	2	60-70	1
EP1	Clarias sp.	cranial roof fragment	5		
EP1	Haplotilapiini indet.	anal pterygophore	1	15-20	1
EP1	O. niloticus	hyomandibula	1	15-20	1
EP1	Haplotilapiini indet.	fin spine	1		
EP1	Haplotilapiini indet.	opercular	1	15-20	1
EP1	Haplotilapiini indet.	3rd precaudal vertebra	1	20-25	1
EP1	Haplotilapiini indet.	3rd precaudal vertebra	1	20-25	1
EP1	Haplotilapiini indet.	posttemporal	1	15-20	1
EP1	O. niloticus	urohyale	1	20-25	1
EP1	O. niloticus	cleithrum	2	15-20	1
EP1	Haplotilapiini indet.	preopercular	2	15-20	1
EP1	Haplotilapiini indet.	caudal vertebra	3	15-20	1
EP1	Haplotilapiini indet.	skull roof fragment	3		
EP1	Haplotilapiini indet.	precaudal vertebra	3	15-20	1
EP1	Haplotilapiini indet.	precaudal vertebra	3	20-25	1
EP1	Clarias sp.	articular	1	50-60	1
EP1	Clarias sp.	articular	1	50-60	1
EP1	Clarias sp.	articular	1	50-60	1
EP1	Clarias sp.	articular	1	50-60	1
EP1	Clarias sp.	articular	1	40-50	1
EP1	Clarias sp.	articular	1	40-50	1
EP1	Clarias sp.	articular	1	30-40	1
EP1	Clarias sp.	articular	1		
EP1	Clarias sp.	caudal vertebra	1	30-40	1
EP1	Clarias sp.	cleithrum	1		
EP1	Clarias sp.	dentary	1	60-70	1
EP1	Clarias sp.	dentary	1		
EP1	Clarias sp.	epihyale	1	40-50	1
EP1	Clarias sp.	hyomandibula	1	50-60	1
EP1	Clarias sp.	hyomandibula	1	40-50	1
EP1	Clarias sp.	mesethmoid	1		
EP1	Clarias sp.	mesethmoid	1	40-50	1
EP1	Clarias sp.	cranial roof fragment	1		
EP1	Clarias sp.	opercular	1	40-50	1
EP1	Clarias sp.	opercular	1	50-60	1
EP1	Clarias sp.	precaudal vertebra	1	50-60	1
EP1	Clarias sp.	precaudal vertebra	1	60-70	1
EP1	Clarias sp.	pectoral spine	1	40-50	1
EP1	Clarias sp.	quadrate	1	50-60	1
EP1	Clarias sp.	quadrate	1	50-60	1
EP1	Clarias sp.	quadrate	1	70-80	1
EP1	Clarias sp.	quadrate	1		
EP1	Clarias sp.	caudal vertebra	2	50-60	1
EP1	Clarias sp.	ceratohyale	2		
EP1	Clarias sp.	ceratohyale	2		

cultural phase	taxon	skeletal element	NISP	SL in cm	number
EP1	Clarias sp.	branchial element	2		
EP1	Clarias sp.	precaudal vertebra	2	30-40	1
EP1	Clarias sp.	caudal vertebra	28	60-70	1
EP1	Clarias sp.	cleithrum	3	30-40	1
EP1	Clarias sp.	cranial roof fragment	35		
EP1	Clarias sp.	cranial roof fragment	4	60-70	1
EP1	Clarias sp.	caudal vertebra	5	70-80	1
EP1	Haplotilapiini indet.	anal pterygophore	1	25-30	1
EP1	Haplotilapiini indet.	anal pterygophore	1	25-30	1
EP1	Haplotilapiini indet.	anal pterygophore	1		
EP1	Haplotilapiini indet.	caudal vertebra	1	20-25	1
EP1	Haplotilapiini indet.	cleithrum	1		
EP1	O. niloticus	mesethmoid	1	25-30	1
EP1	O. niloticus	mesethmoid	1	20-25	1
EP1	Haplotilapiini indet.	mesethmoid	1	20-25	1
EP1	Haplotilapiini indet.	opercular	1	15-20	1
EP1	Haplotilapiini indet.	preopercular	1		
EP1	Haplotilapiini indet.	dorsal or anal pterygophore	1		
EP1	Haplotilapiini indet.	skull roof fragment	10		
EP1	Haplotilapiini indet.	precaudal vertebra	16	15-20	1
EP1	Haplotilapiini indet.	cleithrum	2	20-25	1
EP1	Haplotilapiini indet.	precaudal vertebra	2	20-25	1
EP1	Haplotilapiini indet.	lepidotrich	3		
EP1	Haplotilapiini indet.	caudal vertebra	8	15-20	1
EP1	Clarias sp.	basioccipital	1	30-40	1
EP1	Clarias sp.	cleithrum	1	25-30	1
EP1	Clarias sp.	cleithrum	1	20-30	1
EP1	Clarias sp.	cleithrum	1	30-40	1
EP1	Clarias sp.	cleithrum	1	60-70	1
EP1	Clarias sp.	coracoid	1		
EP1	Clarias sp.	coracoid	1		
EP1	Clarias sp.	epihyale	1	50-60	1
EP1	Clarias sp.	hyomandibula	1	60-70	1
EP1	Clarias sp.	precaudal vertebra	1		
EP1	Clarias sp.	pectoral spine	1	50-60	1
EP1	Clarias sp.	cranial roof fragment	10		
EP1	Clarias sp.	caudal vertebra	2	40-50	1
EP1	Clarias sp.	cranial roof fragment	2	40-50	1
EP1	Clarias sp.	caudal vertebra	3	50-60	1
EP1	Clarias sp.	cranial roof fragment	5	50-60	1
EP1	Haplotilapiini indet.	anal pterygophore	1	15-20	1
EP1	Haplotilapiini indet.	anal pterygophore	1	20-25	1
EP1	Haplotilapiini indet.	basipterygium	1		
EP1	Haplotilapiini indet.	caudal vertebra	1	15-20	1
EP1	Haplotilapiini indet.	cleithrum	1	20-25	1
EP1	Haplotilapiini indet.	skull roof fragment	1		
EP1	Haplotilapiini indet.	precaudal vertebra	2		
EP1	Haplotilapiini indet.	precaudal vertebra	2	20-25	1
EP1	Haplotilapiini indet.	preopercular	2	20-25	1
EP1	Clarias sp.	coracoid	1	70-80	1
EP1	Clarias sp.	cranial roof fragment	2		
EP1	Haplotilapiini indet.	skull roof fragment	1	20-25	1
EP1	Clarias sp.	cranial roof fragment	1		
EP1	Clarias sp.	unidentified	1		
EP1	Clarias sp.	cranial roof fragment	1		
EP1	Clarias sp.	cranial roof fragment	1		
EP1	Clarias sp.	caudal vertebra	2	40-50	1
EP1	Haplotilapiini indet.	basioccipital	1	20-25	1

cultural phase	taxon	skeletal element	NISP	SL in cm	number
EP1	Haplotilapiini indet.	basipterygium	1	15-20	1
EP1	Haplotilapiini indet.	parasphenoid	1	20-25	1
EP1	O. niloticus	preopercular	1	15-20	1
EP1	O. niloticus	preopercular	1	20-25	1
EP1	O. niloticus	suborbital	1	20-25	1
EP1	Haplotilapiini indet.	caudal vertebra	2	20-25	1
EP1	Haplotilapiini indet.	cleithrum	2		
EP1	Haplotilapiini indet.	precaudal vertebra	2	15-20	1
EP1	Haplotilapiini indet.	suborbital	1	15-20	1
EP1	Haplotilapiini indet.	skull roof fragment	1		
EP1	Clarias sp.	cleithrum	1		
EP1	Clarias sp.	coracoid	1		
EP1	Clarias sp.	mesethmoid	1	30-40	1
EP1	Clarias sp.	cranial roof fragment	2		
EP1	Clarias sp.	cranial roof fragment	1		
EP1	Haplotilapiini indet.	cleithrum	1		
EP1	Clarias sp.	caudal vertebra	1		
EP1	Clarias sp.	cleithrum	1	40-50	1
EP1	Clarias sp.	coracoid	1		
EP1	Clarias sp.	cleithrum	2		
EP1	Haplotilapiini indet.	caudal vertebra	1	15-20	1
EP1	Haplotilapiini indet.	cleithrum	1	20-25	1
EP1	Clarias sp.	cleithrum	1	50-60	1
EP1	Clarias sp.	3rd precaudal vertebra	1	20-30	1
EP1	Clarias sp.	quadrate	1	60-70	1
EP1	Clarias sp.	cranial roof fragment	4		
EP1	Clarias sp.	cranial roof fragment	1	70-80	1
EP1	Clarias sp.	epi- & ceratohyale	1	50-60	1
EP1	Clarias sp.	cleithrum	1	30-40	1
EP1	Clarias sp.	cleithrum	1		
EP1	Clarias sp.	dentary	1	40-50	1
EP1	Clarias sp.	cranial roof fragment	1	70-80	1
EP1	Clarias sp.	cranial roof fragment	1		
EP1	Clarias sp.	pectoral spine	1	60-70	1
EP1	Clarias sp.	cranial roof fragment	1		
EP1	Clarias sp.	cranial roof fragment	1	40-50	1
EP1	Clarias sp.	opercular	1	50-60	1
EP1	Clarias sp.	cranial roof fragment	2		
EP1	Clarias sp.	caudal vertebra	1	50-60	1
EP1	Clarias sp.	caudal vertebra	1		
EP1	Clarias sp.	cranial roof fragment	1		
EP1	Clarias sp.	cranial roof fragment	1		
EP1	Clarias sp.	opercular	1	40-50	1
EP1	C. gariepinus	vomerine toothplate	1		
EP1	Clarias sp.	cleithrum	1	70-80	1
EP1	Clarias sp.	dentary	1	30-40	1
EP1	Clarias sp.	mesethmoid	1	60-70	1
EP1	Clarias sp.	opercular	1	40-50	1
EP1	Clarias sp.	cranial roof fragment	7		
EP1	Clarias sp.	pectoral spine	1	50-60	1
EP1	Clarias sp.	cranial roof fragment	2		
EP1	C. zillii	2nd precaudal vertebra	1		
EP1	Clarias sp.	caudal vertebra	1		
EP1	Clarias sp.	cleithrum	1	50-60	1
EP1	Clarias sp.	epihyale	1	70-80	1
EP1	Clarias sp.	epihyale	1	60-70	1
EP1	Clarias sp.	unidentified	1		
EP1	Clarias sp.	cranial roof fragment	3		

cultural phase	taxon	skeletal element	NISP	SL in cm	number
EP1	Clarias sp.	cranial roof fragment	1	60-70	1
EP1	Clarias sp.	pectoral spine	1	50-60	1
EP1	Clarias sp.	coracoid	2		
EP1	Clarias sp.	quadrate	1	50-60	1
EP1	Clarias sp.	quadrate	1	50-60	1
EP1	Clarias sp.	cranial roof fragment	6		
EP1	Clarias sp.	cranial roof fragment	1		
EP1	Haplotilapiini indet.	cleithrum	1	15-20	1
EP1	Clarias sp.	cranial roof fragment	2		
EP1	Clarias sp.	caudal vertebra	1		
EP1	Clarias sp.	cleithrum	1		
EP1	C. gariepinus	vomerine toothplate	1	40-50	1
EP1	Clarias sp.	cranial roof fragment	1	40-50	1
EP1	Clarias sp.	cranial roof fragment	5		
EP1	Haplotilapiini indet.	cleithrum	1	20-25	1
EP1	Haplotilapiini indet.	opercular	1	20-25	1
EP1	Clarias sp.	epi- & ceratohyale	1	40-50	1
EP1	Clarias sp.	cranial roof fragment	1		
EP1	Clarias sp.	pectoral spine	1		
EP1	Clarias sp.	cleithrum	2		
EP1	Clarias sp.	cleithrum	2		
EP1	Clarias sp.	cranial roof fragment	5		
EP1	Clarias sp.	cleithrum	1	50-60	1
EP1	Clarias sp.	cranial roof fragment	1	40-50	1
EP1	Clarias sp.	cranial roof fragment	10		
EP1	Clarias sp.	cleithrum	2		
EP1	Clarias sp.	cleithrum	1	60-70	1
EP1	C. gariepinus	vomerine toothplate	1	40-50	1
EP1	Clarias sp.	caudal vertebra	1	60-70	1
EP1	Clarias sp.	cleithrum	1	30-40	1
EP1	Clarias sp.	cranial roof fragment	1		
EP1	Clarias sp.	precaudal vertebra	1	50-60	1
EP1	Clarias sp.	precaudal vertebra	1	60-70	1
EP1	Clarias sp.	cranial roof fragment	2		
EP1	Haplotilapiini indet.	anal pterygophore	1	15-20	1
EP1	Haplotilapiini indet.	cleithrum	1	15-20	1
EP1	Haplotilapiini indet.	opercular	1	15-20	1
EP1	C. gariepinus	vomerine toothplate	1	40-50	1
EP1	Clarias sp.	cranial roof fragment	1		
EP1	Clarias sp.	precaudal vertebra	1		
EP1	Clarias sp.	cleithrum	2		
EP1	Clarias sp.	cranial roof fragment	2		
EP1	Haplotilapiini indet.	cleithrum	1	15-20	1
EP1	Haplotilapiini indet.	cleithrum	1		
EP1	Haplotilapiini indet.	unidentified	1		
EP1	Haplotilapiini indet.	precaudal vertebra	1	20-25	1
EP1	Clarias sp.	caudal vertebra	1	60-70	1
EP1	Clarias sp.	cranial roof fragment	2	40-50	1
EP1	Clarias sp.	epihyale	1	70-80	1
EP1	Clarias sp.	cranial roof fragment	3		
EP1	Clarias sp.	caudal vertebra	1	40-50	1
EP1	Haplotilapiini indet.	cleithrum	2	20-25	1
EP1	Clarias sp.	cranial roof fragment	1	50-60	1
EP1	Clarias sp.	cranial roof fragment	3		
EP1	Haplotilapiini indet.	unidentified	1		
EP1	Clarias sp.	epi- & ceratohyale	1	40-50	1
EP1	O. niloticus	suborbital	1	20-25	1
EP1	Clarias sp.	cleithrum	1		

cultural phase	taxon	skeletal element	NISP	SL in cm	number
EP1	Haplotilapiini indet.	precaudal vertebra	1	15-20	1
EP1	Haplotilapiini indet.	precaudal vertebra	1	20-25	1
EP1	Clarias sp.	epihyale	1	60-70	1
EP1	C. gariepinus	vomer	1	40-50	1
EP1	Clarias sp.	cleithrum	1	60-70	1
EP1	Clarias sp.	cranial roof fragment	1	60-70	1
EP1	Clarias sp.	caudal vertebra	1		
EP1	Clarias sp.	caudal vertebra	1	50-60	1
EP1	Clarias sp.	hyomandibula	1	40-50	1
EP1	Clarias sp.	cranial roof fragment	1	60-70	1
EP1	Clarias sp.	parasphenoid	1		
EP1	Clarias sp.	precaudal vertebra	1	50-60	1
EP1	Clarias sp.	preopercular	1		
EP1	Clarias sp.	cleithrum	2	20-25	1
EP1	Clarias sp.	cranial roof fragment	2		
EP1	Clarias sp.	cranial roof fragment	5		
EP1	Haplotilapiini indet.	precaudal vertebra	1	20-25	1
EP1	Clarias sp.	articular & hyomandibular	1	50-60	1
EP1	Clarias sp.	cleithrum	1	60-70	1
EP1	Clarias sp.	coracoid	1	40-50	1
EP1	Clarias sp.	dentary	1	50-60	1
EP1	Clarias sp.	epi- & ceratohyale	1	80-90	1
EP1	Clarias sp.	epi- & ceratohyale	1	70-80	1
EP1	Clarias sp.	cranial roof fragment	6		
EP1	Clarias sp.	cranial roof fragment	7		
EP1	Clarias sp.	caudal vertebra	1	60-70	1
EP1	Clarias sp.	ceratohyale	1	50-60	1
EP1	Clarias sp.	mesethmoid	1	50-60	1
EP1	Clarias sp.	precaudal vertebra	1	60-70	1
EP1	Clarias sp.	cranial roof fragment	2	60-70	1
EP1	Clarias sp.	cranial roof fragment	9		
EP1	Clarias sp.	cleithrum	1		
EP1	Clarias sp.	mesethmoid	1	50-60	1
EP1	Clarias sp.	cranial roof fragment	1	50-60	1
EP1	Clarias sp.	cranial roof fragment	2	40-50	1
EP1	Clarias sp.	articular	1	60-70	1
EP1	Clarias sp.	articular	1	50-60	1
EP1	Clarias sp.	caudal vertebra	1	70-80	1
EP1	Clarias sp.	caudal vertebra	1		
EP1	Clarias sp.	caudal vertebra	1	70-80	1
EP1	Clarias sp.	ceratohyale	1	60-70	1
EP1	Clarias sp.	cleithrum	1		
EP1	Clarias sp.	cleithrum	1	50-60	1
EP1	Clarias sp.	coracoid	1	50-60	1
EP1	Clarias sp.	coracoid	1		
EP1	Clarias sp.	dentary	1	50-60	1
EP1	Clarias sp.	dentary	1		
EP1	Clarias sp.	epihyale	1		
EP1	Clarias sp.	hyomandibula	1	30-40	1
EP1	Clarias sp.	mesethmoid	1	70-80	1
EP1	Clarias sp.	cranial roof fragment	1	70-80	1
EP1	Clarias sp.	precaudal vertebra	1	60-70	1
EP1	Clarias sp.	precaudal vertebra	1	40-50	1
EP1	Clarias sp.	precaudal vertebra	1	60-70	1
EP1	Clarias sp.	pectoral spine	1	30-40	1
EP1	Clarias sp.	pectoral spine	1	60-70	1
EP1	Clarias sp.	caudal vertebra	2	60-70	1
EP1	Clarias sp.	caudal vertebra	2	40-50	1

cultural phase	taxon	skeletal element	NISP	SL in cm	number
EP1	Clarias sp.	cranial roof fragment	25		
EP1	Clarias sp.	cleithrum	3		
EP1	Clarias sp.	cranial roof fragment	4		
EP1	Clarias sp.	cranial roof fragment	9		
EP1	Haplotilapiini indet.	anal pterygophore	1	20-25	1
EP1	Haplotilapiini indet.	anal pterygophore	1	20-25	1
EP1	Haplotilapiini indet.	caudal vertebra	1	20-25	1
EP1	Haplotilapiini indet.	cleithrum	1		
EP1	Haplotilapiini indet.	cleithrum	1	20-25	1
EP1	Haplotilapiini indet.	cleithrum	1		
EP1	Haplotilapiini indet.	skull roof fragment	1		
EP1	Haplotilapiini indet.	opercular	1	20-25	1
EP1	Haplotilapiini indet.	precaudal vertebra	1	25-30	1
EP1	Haplotilapiini indet.	subopercular	1		
EP1	Haplotilapiini indet.	cleithrum	2	20-25	1
EP1	Haplotilapiini indet.	precaudal vertebra	2	20-25	1
EP1	Clarias sp.	ceratohyale	1	40-50	1
EP1	Clarias sp.	ceratohyale	1	70-80	1
EP1	Clarias sp.	cleithrum	1		
EP1	Clarias sp.	dentary	1	60-70	1
EP1	Clarias sp.	mesethmoid	1	60-70	1
EP1	Clarias sp.	cranial roof fragment	1	40-50	1
EP1	Clarias sp.	cranial roof fragment	1	50-60	1
EP1	Clarias sp.	cranial roof fragment	3		
EP1	Clarias sp.	dentary	1	50-60	1
EP1	Clarias sp.	pectoral spine	1	50-60	1
EP1	Clarias sp.	cranial roof fragment	2		
EP1	Haplotilapiini indet.	opercular	1	15-20	1
EP1	Clarias sp.	cleithrum	1	30-40	1
EP1	Clarias sp.	cleithrum	1	40-50	1
EP1	Clarias sp.	cleithrum	1	30-40	1
EP1	Clarias sp.	cranial roof fragment	1	30-40	1
EP1	Clarias sp.	precaudal vertebra	1	40-50	1
EP1	Clarias sp.	pectoral spine	1	60-70	1
EP1	Clarias sp.	quadrate	1	60-70	1
EP1	Clarias sp.	quadrate	1	70-80	1
EP1	Clarias sp.	cranial roof fragment	13		
EP1	Clarias sp.	cranial roof fragment	2	50-60	1
EP1	C. gariepinus	vomerine toothplate	1		
EP1	Clarias sp.	caudal vertebra	1	40-50	1
EP1	Clarias sp.	cleithrum	1	70-80	1
EP1	Clarias sp.	precaudal vertebra	1	40-50	1
EP1	Clarias sp.	pectoral spine	1	30-40	1
EP1	Clarias sp.	cranial roof fragment	2	40-50	1
EP1	Clarias sp.	cranial roof fragment	3		
EP1	Haplotilapiini indet.	basipterygium	1	15-20	1
EP1	Haplotilapiini indet.	basipterygium	1	20-25	1
EP1	Clarias sp.	cleithrum	1		
EP1	Clarias sp.	precaudal vertebra	1	50-60	1
EP1	Clarias sp.	cranial roof fragment	4		
EP1	Clarias sp.	cleithrum	1	40-50	1
EP1	Clarias sp.	cranial roof fragment	1	70-80	1
EP1	Clarias sp.	cranial roof fragment	4		
EP1	Clarias sp.	cranial roof fragment	1		
EP1	Clarias sp.	caudal vertebra	31	60-70	1
EP1	Clarias sp.	caudal vertebra	1		
EP1	Clarias sp.	pectoral spine	1	60-70	1
EP1	Clarias sp.	unidentified	1		

cultural phase	taxon	skeletal element	NISP	SL in cm	number
EP1	Clarias sp.	cranial roof fragment	1	40-50	1
EP1	Clarias sp.	cranial roof fragment	4		
EP1	Haplotilapiini indet.	cleithrum	1	20-25	1
EP1	Clarias sp.	cranial roof fragment	2		
EP1	Clarias sp.	articular	1	80-90	1
EP1	Clarias sp.	articular	1	50-60	1
EP1	Clarias sp.	caudal vertebra	1	70-80	1
EP1	Clarias sp.	ceratohyale	1	30-40	1
EP1	Clarias sp.	cleithrum	1	30-40	1
EP1	Clarias sp.	dentary	1	60-70	1
EP1	Clarias sp.	hyomandibula	1	70-80	1
EP1	Clarias sp.	pectoral spine	1	40-50	1
EP1	Clarias sp.	pectoral spine	1	60-70	1
EP1	Clarias sp.	quadrate	1	80-90	1
EP1	Clarias sp.	cranial roof fragment	8		
EP1	Haplotilapiini indet.	cleithrum	1	20-25	1
EP1	Haplotilapiini indet.	precaudal vertebra	2		
EP1	Clarias sp.	cleithrum	1	20-25	1
EP1	Clarias sp.	cranial roof fragment	1		
EP1	Clarias sp.	opercular	1	30-40	1
EP1	C. gariepinus	vomerine toothplate	1	40-50	1
EP1	Clarias sp.	articular	1	50-60	1
EP1	Clarias sp.	caudal vertebra	1		
EP1	Clarias sp.	caudal vertebra	1	40-50	1
EP1	Clarias sp.	cleithrum	1	60-70	1
EP1	Clarias sp.	coracoid	1		
EP1	Clarias sp.	unidentified	1		
EP1	Clarias sp.	cranial roof fragment	1		
EP1	Clarias sp.	cranial roof fragment	1	70-80	1
EP1	Clarias sp.	cranial roof fragment	1	70-80	1
EP1	Clarias sp.	pectoral spine	1	30-40	1
EP1	Clarias sp.	cleithrum	2		
EP1	Clarias sp.	cranial roof fragment	2	40-50	1
EP1	Clarias sp.	precaudal vertebra	2	50-60	1
EP1	Clarias sp.	cranial roof fragment	3		
EP1	Clarias sp.	cranial roof fragment	3	60-70	1
EP1	Clarias sp.	cranial roof fragment	8		
EP1	Haplotilapiini indet.	anal pterygophore	1	15-20	1
EP1	Haplotilapiini indet.	basipterygium	1	15-20	1
EP1	Haplotilapiini indet.	caudal vertebra	1	15-20	1
EP1	Haplotilapiini indet.	caudal vertebra	1	15-20	1
EP1	Haplotilapiini indet.	caudal vertebra	1	20-25	1
EP1	Haplotilapiini indet.	cleithrum	1		
EP1	Haplotilapiini indet.	hyomandibula	1	20-25	1
EP1	Haplotilapiini indet.	precaudal vertebra	1	15-20	1
EP1	Haplotilapiini indet.	lepidotrich	2		
EP1	C. gariepinus	vomerine toothplate	1		
EP1	Clarias sp.	ceratohyale	1	30-40	1
EP1	Clarias sp.	cleithrum	1		
EP1	Clarias sp.	hyomandibula	1		
EP1	Clarias sp.	cranial roof fragment	1	60-70	1
EP1	Clarias sp.	cranial roof fragment	1		
EP1	Clarias sp.	cranial roof fragment	1	70-80	1
EP1	Clarias sp.	precaudal vertebra	1	40-50	1
EP1	Clarias sp.	precaudal vertebra	1	15-20	1
EP1	Clarias sp.	cranial roof fragment	8		
EP1	Haplotilapiini indet.	precaudal vertebra	1	20-25	1
EP1	Clarias sp.	cleithrum	1		

cultural phase	taxon	skeletal element	NISP	SL in cm	number
EP1	Clarias sp.	cranial roof fragment	1		
EP1	C. zillii	1st precaudal vertebra	1	20-25	1
EP1	Clarias sp.	articular	1	30-40	1
EP1	Clarias sp.	cleithrum	1	20-30	1
EP1	Clarias sp.	cleithrum	1		
EP1	Clarias sp.	dentary	1	40-50	1
EP1	Clarias sp.	cranial roof fragment	1		
EP1	Clarias sp.	cranial roof fragment	1	30-40	1
EP1	Clarias sp.	cleithrum	1		
EP1	Clarias sp.	cranial roof fragment	3		
EP1	Clarias sp.	cranial roof fragment	3		
EP1	Clarias sp.	caudal vertebra	1	50-60	1
EP1	Clarias sp.	cleithrum	1		
EP1	Clarias sp.	cranial roof fragment	1		
EP1	Clarias sp.	articular	1	50-60	1
EP1	Clarias sp.	caudal vertebra	1	50-60	1
EP1	Clarias sp.	cleithrum	1		
EP1	Clarias sp.	cleithrum	1		
EP1	Clarias sp.	mesethmoid	1	40-50	1
EP1	Clarias sp.	cranial roof fragment	1		
EP1	Clarias sp.	parasphenoid	1		
EP1	Clarias sp.	precaudal vertebra	1	50-60	1
EP1	Clarias sp.	pectoral spine	1	30-40	1
EP1	Clarias sp.	pectoral spine	1	40-50	1
EP1	Clarias sp.	urohyale	1	60-70	1
EP1	Clarias sp.	basioccipital	1	50-60	1
EP1	Clarias sp.	basioccipital	1	50-60	1
EP1	Clarias sp.	basioccipital	1	40-50	1
EP1	Clarias sp.	caudal vertebra	1	40-50	1
EP1	Clarias sp.	caudal vertebra	1		
EP1	Clarias sp.	ceratohyale	1	40-50	1
EP1	Clarias sp.	cleithrum	1	50-60	1
EP1	Clarias sp.	cleithrum	1	50-60	1
EP1	Clarias sp.	dentary	1	40-50	1
EP1	Clarias sp.	hyomandibula	1	50-60	1
EP1	Clarias sp.	hyomandibula	1	50-60	1
EP1	Clarias sp.	cranial roof fragment	1	60-70	1
EP1	Clarias sp.	cranial roof fragment	1	70-80	1
EP1	Clarias sp.	opercular	1	40-50	1
EP1	Clarias sp.	opercular	1	40-50	1
EP1	Clarias sp.	opercular	1	30-40	1
EP1	Clarias sp.	palatinum	1		
EP1	Clarias sp.	precaudal vertebra	1	40-50	1
EP1	Clarias sp.	precaudal vertebra	1	50-60	1
EP1	Clarias sp.	precaudal vertebra	1		
EP1	Clarias sp.	pectoral spine	1	60-70	1
EP1	Clarias sp.	pectoral spine	1	40-50	1
EP1	Clarias sp.	pectoral spine	1	40-50	1
EP1	Clarias sp.	pectoral spine	1		
EP1	Clarias sp.	quadrate	1	50-60	1
EP1	Clarias sp.	vomerine toothplate	1	50-60	1
EP1	Clarias sp.	vomerine toothplate	1	50-60	1
EP1	Clarias sp.	caudal vertebra	2	50-60	2
EP1	Clarias sp.	cranial roof fragment	2	50-60	2
EP1	Clarias sp.	cranial roof fragment	26		
EP1	Clarias sp.	caudal vertebra	3	60-70	3
EP1	Clarias sp.	coracoid	3		
EP1	Clarias sp.	caudal vertebra	4	30-40	4

cultural phase	taxon	skeletal element	NISP	SL in cm	number
EP1	Clarias sp.	cleithrum	7		
EP1	Clarias sp.	articular	1	40-50	1
EP1	Clarias sp.	caudal vertebra	1	50-60	1
EP1	Clarias sp.	caudal vertebra	1	60-70	1
EP1	Clarias sp.	cleithrum	1	60-70	1
EP1	Clarias sp.	cleithrum	1	50-60	1
EP1	Clarias sp.	coracoid	1		
EP1	Clarias sp.	dentary	1	40-50	1
EP1	Clarias sp.	epihyale	1	50-60	1
EP1	Clarias sp.	pectoral spine	1	50-60	1
EP1	Clarias sp.	cranial roof fragment	5	40-50	1
EP1	Clarias sp.	caudal vertebra	1	30-40	1
EP1	Clarias sp.	caudal vertebra	1	50-60	1
EP1	Clarias sp.	cleithrum	1	50-60	1
EP1	Clarias sp.	cleithrum	1	30-40	1
EP1	Clarias sp.	dentary	1	40-50	1
EP1	Clarias sp.	dentary	1	40-50	1
EP1	Clarias sp.	urohyale	1	50-60	1
EP1	Clarias sp.	cranial roof fragment	3	50-60	1
EP1	Clarias sp.	cranial roof fragment	5	30-40	2
EP1	Clarias sp.	precaudal vertebra	1	60-70	1
EP1	Clarias sp.	cranial roof fragment	3		
EP1	Clarias sp.	caudal vertebra	1	60-70	1
EP1	Clarias sp.	cranial roof fragment	1	40-50	1
EP1	Haplotilapiini indet.	opercular	1	15-20	1
EP1	Haplotilapiini indet.	opercular	1		
EP1	Haplotilapiini indet.	dorsal or anal pterygophore	1	20-25	1
EP1	Haplotilapiini indet.	dorsal or anal pterygophore	1	20-25	1
EP1	Haplotilapiini indet.	hyomandibula	1		
EP1	Haplotilapiini indet.	opercular	1		
EP1	Haplotilapiini indet.	cleithrum	2		
EP1	Haplotilapiini indet.	opercular	1	20-25	1
EP1	Haplotilapiini indet.	opercular	1		
EP1	Haplotilapiini indet.	precaudal vertebra	1	20-25	1
EP1	Haplotilapiini indet.	dorsal or anal pterygophore	1	20-25	1
EP1	Haplotilapiini indet.	dorsal or anal pterygophore	1	15-20	1
EP1	Haplotilapiini indet.	supracleithrum	1		
EP1	Haplotilapiini indet.	cleithrum	1		
EP1	Haplotilapiini indet.	fin spine	1		
EP1	Haplotilapiini indet.	skull roof fragment	1		
EP1	Haplotilapiini indet.	dorsal or anal pterygophore	1	20-25	1
EP1	Haplotilapiini indet.	precaudal vertebra	4	15-20	4
EP1	Haplotilapiini indet.	basioccipital	1	20-25	1
EP1	Haplotilapiini indet.	dentary	1		
EP1	Haplotilapiini indet.	hyomandibula	1	20-25	1
EP1	Haplotilapiini indet.	hyomandibula	1	20-25	1
EP1	Haplotilapiini indet.	hyomandibula	1	20-25	1
EP1	O. niloticus	mesethmoid	1	25-30	1
EP1	Haplotilapiini indet.	opercular	1	20-25	1
EP1	Haplotilapiini indet.	opercular	1	20-25	1
EP1	Haplotilapiini indet.	opercular	1	10-15	1
EP1	Haplotilapiini indet.	opercular	1	15-20	1
EP1	Haplotilapiini indet.	opercular	1	20-25	1
EP1	Haplotilapiini indet.	opercular	1		
EP1	Haplotilapiini indet.	3rd precaudal vertebra	1	15-20	1
EP1	Haplotilapiini indet.	dorsal or anal pterygophore	1	20-25	1
EP1	Haplotilapiini indet.	dorsal or anal pterygophore	1	20-25	1
EP1	Haplotilapiini indet.	dorsal or anal pterygophore	1	20-25	1

cultural phase	taxon	skeletal element	NISP	SL in cm	number
EP1	Haplotilapiini indet.	dorsal or anal pterygophore	1	15-20	1
EP1	Haplotilapiini indet.	dorsal or anal pterygophore	1	20-25	1
EP1	Haplotilapiini indet.	dorsal or anal pterygophore	1	15-20	1
EP1	Haplotilapiini indet.	dorsal or anal pterygophore	1	10-15	1
EP1	Haplotilapiini indet.	dorsal or anal pterygophore	1	15-20	1
EP1	Haplotilapiini indet.	dorsal or anal pterygophore	1	20-25	1
EP1	Haplotilapiini indet.	dorsal or anal pterygophore	1	20-25	1
EP1	Haplotilapiini indet.	supracleithrum	1	20-25	1
EP1	Haplotilapiini indet.	preopercular	10		
EP1	Haplotilapiini indet.	precaudal vertebra	12	15-20	12
EP1	Haplotilapiini indet.	caudal vertebra	14	15-20	14
EP1	Haplotilapiini indet.	skull roof fragment	18	20-25	3
EP1	Haplotilapiini indet.	cleithrum	19	20-25	2
EP1	Haplotilapiini indet.	urohyale	2	20-25	1
EP1	Haplotilapiini indet.	basipterygium	3	15-20	1
EP1	Haplotilapiini indet.	supracleithrum	3	15-20	3
EP1	Haplotilapiini indet.	lepidotrich	4		
EP1	Haplotilapiini indet.	opercular	1	15-20	1
EP1	Haplotilapiini indet.	precaudal vertebra	1	15-20	1
EP1	Haplotilapiini indet.	precaudal vertebra	1	20-25	1
EP1	Haplotilapiini indet.	dorsal or anal pterygophore	1	20-25	1
EP1	Haplotilapiini indet.	cleithrum	2		
EP1	Haplotilapiini indet.	preopercular	2		
EP1	Haplotilapiini indet.	caudal vertebra	1	15-20	1
EP1	Haplotilapiini indet.	cleithrum	1		
EP1	Haplotilapiini indet.	mesethmoid	1	20-25	1
EP1	Haplotilapiini indet.	mesethmoid	1	15-20	1
EP1	Haplotilapiini indet.	opercular	1	15-20	1
EP1	Haplotilapiini indet.	opercular	1		
EP1	Haplotilapiini indet.	3rd precaudal vertebra	1	20-25	1
EP1	Haplotilapiini indet.	precaudal vertebra	1	15-20	1
EP1	Haplotilapiini indet.	dorsal or anal pterygophore	1	20-25	1
EP1	Haplotilapiini indet.	supracleithrum	1	20-25	1
EP1	Haplotilapiini indet.	skull roof fragment	2		
EP1	Haplotilapiini indet.	precaudal vertebra	2	15-20	2
EP1	Haplotilapiini indet.	opercular	3		
EP1	Haplotilapiini indet.	skull roof fragment	1		
EP1	Haplotilapiini indet.	opercular	1		
EP1	Haplotilapiini indet.	cleithrum	2		
EP1	Haplotilapiini indet.	cleithrum	1		
EP1	Haplotilapiini indet.	opercular	1	15-20	1
EP1	O. niloticus	1st precaudal vertebra	1	15-20	1
EP1	Haplotilapiini indet.	preopercular	2		
EP1	Haplotilapiini indet.	1st precaudal vertebra	1	20-25	1
EP1	Haplotilapiini indet.	1st precaudal vertebra	1	20-25	1
EP1	Clarias sp.	caudal vertebra	1		
EP1	Clarias sp.	epihyale	1	30-40	1
EP1	Clarias sp.	precaudal vertebra	1	60-70	1
EP1	Clarias sp.	cranial roof fragment	5	60-70	1
EP1	Clarias sp.	articular	1	40-50	1
EP1	Clarias sp.	caudal vertebra	1	60-70	1
EP1	Clarias sp.	ceratohyale	1	60-70	1
EP1	Clarias sp.	cleithrum	1		
EP1	Clarias sp.	pectoral spine	1	60-70	1
EP1	Clarias sp.	pectoral spine	1	20-30	1
EP1	Clarias sp.	cranial roof fragment	10		
EP1	Clarias sp.	coracoid	2	40-50	1
EP1	Haplotilapiini indet.	hyomandibula	1	20-25	1

cultural phase	taxon	skeletal element	NISP	SL in cm	number
EP1	Haplotilapiini indet.	opercular	1	10-15	1
EP1	Haplotilapiini indet.	precaudal vertebra	1	15-20	1
EP1	Haplotilapiini indet.	opercular	2		
EP1	Clarias sp.	cleithrum	1	30-40	1
EP1	Clarias sp.	cleithrum	1	40-50	1
EP1	Clarias sp.	cleithrum	1	30-40	1
EP1	Clarias sp.	cleithrum	1		
EP1	Clarias sp.	mesethmoid	1	30-40	1
EP1	Clarias sp.	pectoral spine	1	50-60	1
EP1	Clarias sp.	cranial roof fragment	7		
EP1	Clarias sp.	articular	1	30-40	1
EP1	Clarias sp.	cleithrum	1		
EP1	Clarias sp.	palatinum	1	60-70	1
EP1	Clarias sp.	parasphenoid	1	60-70	1
EP1	Clarias sp.	caudal vertebra	3	50-60	3
EP1	Clarias sp.	cranial roof fragment	4	50-60	1
EP1	Clarias sp.	articular	1	50-60	1
EP1	Clarias sp.	cleithrum	1	70-80	1
EP1	Clarias sp.	dentary	1	50-60	1
EP1	Clarias sp.	branchial element	1		
EP1	Clarias sp.	precaudal vertebra	1	60-70	1
EP1	Clarias sp.	cleithrum	2		
EP1	Clarias sp.	cranial roof fragment	2		
EP1	Clarias sp.	cleithrum	1	60-70	1
EP1	Clarias sp.	epihyale	1	50-60	1
EP1	Clarias sp.	unidentified	1		
EP1	Clarias sp.	cleithrum	1		
EP1	Clarias sp.	cranial roof fragment	2		
EP1	Haplotilapiini indet.	articular	1	10-15	1
EP1	Haplotilapiini indet.	caudal vertebra	1	60-70	1
EP1	Haplotilapiini indet.	skull roof fragment	1	20-25	1
EP1	Haplotilapiini indet.	ceratohyale	2		
EP1	Haplotilapiini indet.	skull roof fragment	2	15-20	1
EP1	Haplotilapiini indet.	opercular	2		
EP1	Haplotilapiini indet.	cleithrum	3		
EP1	Haplotilapiini indet.	basioccipital	1	20-25	1
EP1	Haplotilapiini indet.	basipterygium	1	15-20	1
EP1	Haplotilapiini indet.	fin spine	1		
EP1	Haplotilapiini indet.	skull roof fragment	1		
EP1	O. niloticus	1st precaudal vertebra	1	20-25	1
EP1	Haplotilapiini indet.	opercular	2		
EP1	Haplotilapiini indet.	cleithrum	3		
EP1	Haplotilapiini indet.	precaudal vertebra	3	15-20	3
EP1	Haplotilapiini indet.	caudal vertebra	5	15-20	5
EP1	Haplotilapiini indet.	caudal vertebra	1	15-20	1
EP1	Haplotilapiini indet.	maxilla	1	15-20	1
EP1	Haplotilapiini indet.	precaudal vertebra	1	15-20	1
EP1	Haplotilapiini indet.	supracleithrum	1	15-20	1
EP1	Haplotilapiini indet.	cleithrum	2		
EP1	Haplotilapiini indet.	skull roof fragment	2	15-20	1
EP1	Haplotilapiini indet.	cleithrum	1		
EP1	Haplotilapiini indet.	1st precaudal vertebra	1	20-25	1
EP1	Haplotilapiini indet.	1st precaudal vertebra	1	15-20	1
EP1	Clarias sp.	cleithrum	1	50-60	1
EP1	Clarias sp.	cranial roof fragment	1	20-30	1
EP1	Clarias sp.	cranial roof fragment	1	70-80	1
EP1	Clarias sp.	cranial roof fragment	2	60-70	1
EP1	Clarias sp.	cranial roof fragment	1	60-70	1

cultural phase	taxon	skeletal element	NISP	SL in cm	number
EP1	Clarias sp.	cleithrum	3		
EP1	Clarias sp.	cranial roof fragment	6		
EP1	Clarias sp.	epihyale	1	40-50	1
EP1	Clarias sp.	mesethmoid	1	50-60	1
EP1	Clarias sp.	cranial roof fragment	1	60-70	1
EP1	Clarias sp.	pectoral spine	1	60-70	1
EP1	Clarias sp.	cranial roof fragment	4	50-60	1
EP1	Clarias sp.	articular	1	50-60	1
EP1	Clarias sp.	ceratohyale	1	40-50	1
EP1	Clarias sp.	coracoid	1		
EP1	Clarias sp.	dentary	1	40-50	1
EP1	Clarias sp.	hyomandibula	1	40-50	1
EP1	Clarias sp.	hyomandibula	1	60-70	1
EP1	Clarias sp.	hyomandibula	1	40-50	1
EP1	Clarias sp.	hyomandibula	1	50-60	1
EP1	Clarias sp.	cranial roof fragment	1	30-40	1
EP1	Clarias sp.	cranial roof fragment	1	40-50	1
EP1	Clarias sp.	cranial roof fragment	1	50-60	1
EP1	Clarias sp.	opercular	1	40-50	1
EP1	Clarias sp.	quadrate	1	40-50	1
EP1	Clarias sp.	quadrate	1	60-70	1
EP1	Clarias sp.	urohyale	1	50-60	1
EP1	Clarias sp.	urohyale	1	50-60	1
EP1	Clarias sp.	cleithrum	2		
EP1	Clarias sp.	cleithrum	1		
EP1	Clarias sp.	mesethmoid	1	40-50	1
EP1	Clarias sp.	cranial roof fragment	3	50-60	2
EP1	Clarias sp.	articular	1	60-70	1
EP1	Clarias sp.	caudal vertebra	1	60-70	1
EP1	Clarias sp.	coracoid	1	30-40	1
EP1	Clarias sp.	cranial roof fragment	5	40-50	1
EP1	Clarias sp.	articular	1	50-60	1
EP1	Clarias sp.	articular	1	50-60	1
EP1	Clarias sp.	caudal vertebra	1	60-70	1
EP1	Clarias sp.	ceratohyale	1	40-50	1
EP1	Clarias sp.	cleithrum	1	40-50	1
EP1	Clarias sp.	dentary	1	40-50	1
EP1	Clarias sp.	cranial roof fragment	1	30-40	1
EP1	Clarias sp.	cranial roof fragment	1	40-50	1
EP1	Clarias sp.	opercular	1	50-60	1
EP1	Clarias sp.	quadrate	1	50-60	1
EP1	Clarias sp.	quadrate	1	40-50	1
EP1	Clarias sp.	cranial roof fragment	4		
EP1	Clarias sp.	ceratohyale	1	50-60	1
EP1	Clarias sp.	cranial roof fragment	1		
EP1	Clarias sp.	ceratohyale	1	60-70	1
EP1	Clarias sp.	cleithrum	1	30-40	1
EP1	Clarias sp.	cleithrum	1	50-60	1
EP1	Clarias sp.	cleithrum	1		
EP1	Clarias sp.	opercular	1	60-70	1
EP1	Clarias sp.	pectoral spine	1		
EP1	Clarias sp.	cranial roof fragment	2	60-70	2
EP1	Clarias sp.	precaudal vertebra	2	40-50	2
EP1	Clarias sp.	caudal vertebra	4	60-70	4
EP1	Clarias sp.	cranial roof fragment	8		
EP1	Clarias sp.	dentary	1	40-50	1
EP1	Clarias sp.	cranial roof fragment	1		
EP1	Clarias sp.	pectoral spine	1	30-40	1

cultural phase	taxon	skeletal element	NISP	SL in cm	number
EP1	Clarias sp.	pectoral spine	1		
EP1	Clarias sp.	cranial roof fragment	6		
EP1	Clarias sp.	caudal vertebra	1	40-50	1
EP1	Clarias sp.	cranial roof fragment	1		
EP1	Clarias sp.	cleithrum	3		
EP1	Clarias sp.	cranial roof fragment	4	30-40	1
EP1	Haplotilapiini indet.	opercular	1		
EP1	Haplotilapiini indet.	cleithrum	2		
EP1	Haplotilapiini indet.	cleithrum	1		
EP1	O. niloticus	opercular	1	15-20	1
EP1	O. niloticus	opercular	1	20-25	1
EP1	Haplotilapiini indet.	dorsal or anal pterygophore	1	20-25	1
EP1	Haplotilapiini indet.	dorsal or anal pterygophore	1	20-25	1
EP1	Haplotilapiini indet.	cleithrum	1		
EP1	Haplotilapiini indet.	skull roof fragment	1		
EP1	Haplotilapiini indet.	opercular	1	10-15	1
EP1	Haplotilapiini indet.	dorsal or anal pterygophore	1		
EP1	Haplotilapiini indet.	cleithrum	2		
EP1	Haplotilapiini indet.	costa	1		
EP1	O. niloticus	hyomandibula	1	20-25	1
EP1	Haplotilapiini indet.	interopercular	1	20-25	1
EP1	Haplotilapiini indet.	fin spine	1		
EP1	Haplotilapiini indet.	skull roof fragment	1	15-20	1
EP1	Haplotilapiini indet.	skull roof fragment	1	20-25	1
EP1	Haplotilapiini indet.	skull roof fragment	1		
EP1	O. niloticus	opercular	1	15-20	1
EP1	Haplotilapiini indet.	opercular	1	20-25	1
EP1	Haplotilapiini indet.	opercular	1	20-25	1
EP1	Haplotilapiini indet.	opercular	1	20-25	1
EP1	Haplotilapiini indet.	opercular	1	20-25	1
EP1	Haplotilapiini indet.	opercular	1	20-25	1
EP1	Haplotilapiini indet.	opercular	1	20-25	1
EP1	Haplotilapiini indet.	opercular	1	20-25	1
EP1	Haplotilapiini indet.	opercular	1	20-25	1
EP1	Haplotilapiini indet.	opercular	1	20-25	1
EP1	Haplotilapiini indet.	opercular	1	10-15	1
EP1	Haplotilapiini indet.	opercular	1		
EP1	Haplotilapiini indet.	premaxilla	1		
EP1	Haplotilapiini indet.	dorsal or anal pterygophore	1	15-20	1
EP1	Haplotilapiini indet.	dorsal or anal pterygophore	1	20-25	1
EP1	Haplotilapiini indet.	dorsal or anal pterygophore	1	20-25	1
EP1	Haplotilapiini indet.	dorsal or anal pterygophore	1	20-25	1
EP1	Haplotilapiini indet.	dorsal or anal pterygophore	1	20-25	1
EP1	Haplotilapiini indet.	subopercular	1	20-25	1
EP1	Haplotilapiini indet.	cleithrum	12		
EP1	Haplotilapiini indet.	basipterygium	2	15-20	2
EP1	Haplotilapiini indet.	precaudal vertebra	2	15-20	2
EP1	Haplotilapiini indet.	preopercular	3		
EP1	Haplotilapiini indet.	supracleithrum	3	20-25	2
EP1	Haplotilapiini indet.	skull roof fragment	1	15-20	1
EP1	O. niloticus	opercular	1	20-25	1
EP1	Haplotilapiini indet.	opercular	1		
EP1	Haplotilapiini indet.	dorsal or anal pterygophore	1	15-20	1
EP1	Haplotilapiini indet.	opercular	1		
EP1	Haplotilapiini indet.	cleithrum	2		
EP1	Haplotilapiini indet.	fin spine	1		
EP1	Haplotilapiini indet.	opercular	1	20-25	1
EP1	Haplotilapiini indet.	dorsal or anal pterygophore	1	15-20	1

cultural phase	taxon	skeletal element	NISP	SL in cm	number
EP1	Haplotilapiini indet.	cleithrum	4		
EP1	Haplotilapiini indet.	precaudal vertebra	1	20-25	1
EP1	Haplotilapiini indet.	ceratohyale	1	20-25	1
EP1	Haplotilapiini indet.	opercular	1	20-25	1
EP1	O. niloticus	1st precaudal vertebra	1	20-25	1
EP1	Haplotilapiini indet.	cleithrum	4		
EP1	Haplotilapiini indet.	opercular	1		
EP1	Haplotilapiini indet.	skull roof fragment	2	15-20	2
EP1	Haplotilapiini indet.	dorsal or anal pterygophore	1	20-25	1
EP1	O. niloticus	opercular	2		
EP1	Clarias sp.	cranial roof fragment	2		
EP1	Clarias sp.	cranial roof fragment	4		
EP1	Clarias sp.	cranial roof fragment	2	60-70	1
EP1	Haplotilapiini indet.	fin spine	1		
EP1	Haplotilapiini indet.	opercular	1		
EP1	Haplotilapiini indet.	ceratohyale	1	25-30	1
EP1	Haplotilapiini indet.	cleithrum	2		
EP1	Haplotilapiini indet.	cleithrum	1		
EP1	Haplotilapiini indet.	precaudal vertebra	1	15-20	1
EP2	Clarias sp.	articular	1	50-60	1
EP2	Clarias sp.	articular	1	60-70	1
EP2	Clarias sp.	caudal vertebra	1		
EP2	Clarias sp.	caudal vertebra	1	50-60	1
EP2	Clarias sp.	caudal vertebra	1	30-40	1
EP2	Clarias sp.	ceratohyale	1	60-70	1
EP2	Clarias sp.	ceratohyale	1	50-60	1
EP2	Clarias sp.	ceratohyale	1	50-60	1
EP2	Clarias sp.	cleithrum	1	50-60	1
EP2	Clarias sp.	cleithrum	1	50-60	1
EP2	Clarias sp.	cleithrum	1		
EP2	Clarias sp.	cleithrum	1		
EP2	Clarias sp.	cleithrum	1	40-50	1
EP2	Clarias sp.	cleithrum	1	60-70	1
EP2	Clarias sp.	coracoid	1		
EP2	Clarias sp.	coracoid	1		
EP2	Clarias sp.	coracoid	1		
EP2	Clarias sp.	coracoid	1	30-40	1
EP2	Clarias sp.	dentary	1	50-60	1
EP2	Clarias sp.	dentary	1	60-70	1
EP2	Clarias sp.	dentary	1	60-70	1
EP2	Clarias sp.	dentary	1	40-50	1
EP2	Clarias sp.	dentary	1		
EP2	Clarias sp.	unidentified	1		
EP2	Clarias sp.	mesethmoid	1	40-50	1
EP2	Clarias sp.	mesethmoid	1	50-60	1
EP2	Clarias sp.	cranial roof fragment	1		
EP2	Clarias sp.	cranial roof fragment	1	50-60	1
EP2	Clarias sp.	precaudal vertebra	1	60-70	1
EP2	Clarias sp.	precaudal vertebra	1	70-80	1
EP2	Clarias sp.	precaudal vertebra	1	60-70	1
EP2	Clarias sp.	precaudal vertebra	1	60-70	1
EP2	Clarias sp.	precaudal vertebra	1	40-50	1
EP2	Clarias sp.	precaudal vertebra	1	60-70	1
EP2	Clarias sp.	pectoral spine	1	40-50	1
EP2	Clarias sp.	pectoral spine	1		
EP2	Clarias sp.	pectoral spine	1	50-60	1
EP2	Clarias sp.	pectoral spine	1	60-70	1
EP2	Clarias sp.	pectoral spine	1	60-70	1

cultural phase	taxon	skeletal element	NISP	SL in cm	number
EP2	Clarias sp.	pectoral spine	1		
EP2	Clarias sp.	pectoral spine	1		
EP2	Clarias sp.	quadrate	1	40-50	1
EP2	Clarias sp.	quadrate	1	50-60	1
EP2	Clarias sp.	quadrate	1	50-60	1
EP2	Clarias sp.	vomerine toothplate	1	60-70	1
EP2	Clarias sp.	vomerine toothplate	1	60-70	1
EP2	Clarias sp.	vomerine toothplate	1	60-70	1
EP2	Clarias sp.	vertebra	1		
EP2	Clarias sp.	cranial roof fragment	13		
EP2	Clarias sp.	cranial roof fragment	13		
EP2	Clarias sp.	cranial roof fragment	14	60-70	1
EP2	Clarias sp.	caudal vertebra	2	50-60	2
EP2	Clarias sp.	caudal vertebra	2	60-70	2
EP2	Clarias sp.	caudal vertebra	2	50-60	2
EP2	Clarias sp.	caudal vertebra	2	40-50	2
EP2	Clarias sp.	cleithrum	2		
EP2	Clarias sp.	cranial roof fragment	2		
EP2	Clarias sp.	cranial roof fragment	20	50-60	1
EP2	Clarias sp.	cranial roof fragment	3		
EP2	Clarias sp.	cranial roof fragment	5		
EP2	Clarias sp.	cranial roof fragment	5		
EP2	Clarias sp.	cranial roof fragment	6		
EP2	Clarias sp.	cranial roof fragment	9		
EP2	Clarias sp.	cranial roof fragment	9		
EP2	Haplotilapiini indet.	articular	1	20-25	1
EP2	Haplotilapiini indet.	articular	1		
EP2	Haplotilapiini indet.	basipterygium	1		
EP2	Haplotilapiini indet.	basipterygium	1		
EP2	Haplotilapiini indet.	caudal vertebra	1	15-20	1
EP2	Haplotilapiini indet.	caudal vertebra	1	15-20	1
EP2	Haplotilapiini indet.	caudal vertebra	1	20-25	1
EP2	Haplotilapiini indet.	cleithrum	1	15-20	1
EP2	Haplotilapiini indet.	cleithrum	1	15-20	1
EP2	O. niloticus	cleithrum	1	15-20	1
EP2	Haplotilapiini indet.	cleithrum	1		
EP2	Haplotilapiini indet.	cleithrum	1	20-25	1
EP2	Haplotilapiini indet.	hyomandibula	1	15-20	1
EP2	Haplotilapiini indet.	hyomandibula	1	20-25	1
EP2	O. niloticus	hyomandibula	1	25-30	1
EP2	Haplotilapiini indet.	hyomandibula	1	15-20	1
EP2	Haplotilapiini indet.	unidentified	1		
EP2	Haplotilapiini indet.	unidentified	1		
EP2	Haplotilapiini indet.	fin spine	1		
EP2	Haplotilapiini indet.	skull roof fragment	1	20-25	1
EP2	Haplotilapiini indet.	skull roof fragment	1	15-20	1
EP2	Haplotilapiini indet.	skull roof fragment	1		
EP2	Haplotilapiini indet.	skull roof fragment	1		
EP2	O. niloticus	opercular	1	15-20	1
EP2	Haplotilapiini indet.	opercular	1	15-20	1
EP2	Haplotilapiini indet.	opercular	1		
EP2	O. niloticus	opercular	1	15-20	1
EP2	Haplotilapiini indet.	opercular	1	15-20	1
EP2	O. niloticus	opercular	1	20-25	1
EP2	Haplotilapiini indet.	opercular	1		
EP2	Haplotilapiini indet.	opercular	1	15-20	1
EP2	Haplotilapiini indet.	opercular	1		
EP2	Haplotilapiini indet.	opercular	1	20-25	1

cultural phase	taxon	skeletal element	NISP	SL in cm	number
EP2	Haplotilapiini indet.	opercular	1	15-20	1
EP2	Haplotilapiini indet.	opercular	1	20-25	1
EP2	Haplotilapiini indet.	opercular	1	15-20	1
EP2	Haplotilapiini indet.	opercular	1		
EP2	Haplotilapiini indet.	parasphenoid	1		
EP2	Haplotilapiini indet.	2nd precaudal vertebra	1	20-25	1
EP2	Haplotilapiini indet.	2nd precaudal vertebra	1	20-25	1
EP2	Haplotilapiini indet.	3rd precaudal vertebra	1	15-20	1
EP2	Haplotilapiini indet.	3rd precaudal vertebra	1	20-25	1
EP2	Haplotilapiini indet.	precaudal vertebra	1	15-20	1
EP2	Haplotilapiini indet.	precaudal vertebra	1	20-25	1
EP2	Haplotilapiini indet.	precaudal vertebra	1	15-20	1
EP2	Haplotilapiini indet.	preopercular	1		
EP2	Haplotilapiini indet.	postcleithrum	1		
EP2	O. niloticus	posttemporal	1	20-25	1
EP2	Haplotilapiini indet.	posttemporal	1	15-20	1
EP2	Haplotilapiini indet.	dorsal or anal pterygophore	1	15-20	1
EP2	Haplotilapiini indet.	dorsal or anal pterygophore	1	15-20	1
EP2	Haplotilapiini indet.	dorsal or anal pterygophore	1	20-25	1
EP2	Haplotilapiini indet.	dorsal or anal pterygophore	1		
EP2	Haplotilapiini indet.	dorsal or anal pterygophore	1	15-20	1
EP2	Haplotilapiini indet.	dorsal or anal pterygophore	1	15-20	1
EP2	Haplotilapiini indet.	dorsal or anal pterygophore	1	15-20	1
EP2	Haplotilapiini indet.	dorsal or anal pterygophore	1	15-20	1
EP2	Haplotilapiini indet.	subopercular	1		
EP2	Haplotilapiini indet.	supracleithrum	1	15-20	1
EP2	Haplotilapiini indet.	supracleithrum	1		
EP2	Haplotilapiini indet.	supracleithrum	1	15-20	1
EP2	Haplotilapiini indet.	caudal vertebra	2	15-20	2
EP2	Haplotilapiini indet.	caudal vertebra	2		
EP2	Haplotilapiini indet.	unidentified	2		
EP2	Haplotilapiini indet.	branchial element	2		
EP2	Haplotilapiini indet.	skull roof fragment	2	15-20	2
EP2	Haplotilapiini indet.	skull roof fragment	2	15-20	2
EP2	Haplotilapiini indet.	skull roof fragment	2	20-25	1
EP2	Haplotilapiini indet.	precaudal vertebra	2	15-20	2
EP2	Haplotilapiini indet.	lepidotrich	3		
EP2	Haplotilapiini indet.	skull roof fragment	3		
EP2	Haplotilapiini indet.	skull roof fragment	3	15-20	2
EP2	Haplotilapiini indet.	precaudal vertebra	3	15-20	3
EP2	Haplotilapiini indet.	urohyale	3	15-20	3
EP2	Haplotilapiini indet.	caudal vertebra	4	15-20	4
EP2	Haplotilapiini indet.	cleithrum	4	15-20	1
EP2	Haplotilapiini indet.	cleithrum	4		
EP2	Haplotilapiini indet.	cleithrum	4	20-25	2
EP2	O. niloticus	cleithrum	4	15-20	4
EP2	Haplotilapiini indet.	precaudal vertebra	4	15-20	4
EP2	Haplotilapiini indet.	cleithrum	5	20-25	1
EP2	Haplotilapiini indet.	cleithrum	7		
EP2	Clarias sp.	cranial roof fragment	1		
EP2	Haplotilapiini indet.	hyomandibula	1	15-20	1
EP2	Haplotilapiini indet.	urohyale	1	20-25	1
EP2	Clarias sp.	parasphenoid	1	60-70	1
EP2	Clarias sp.	cranial roof fragment	2		
EP2	Haplotilapiini indet.	caudal vertebra	1		
EP2	Clarias sp.	caudal vertebra	1	40-50	1
EP2	Clarias sp.	cleithrum	1	30-40	1
EP2	Clarias sp.	cleithrum	1	60-70	1

cultural phase	taxon	skeletal element	NISP	SL in cm	number
EP2	Clarias sp.	hyomandibula	1	40-50	1
EP2	Clarias sp.	pectoral spine	1	30-40	1
EP2	Clarias sp.	pectoral spine	1	50-60	1
EP2	Clarias sp.	quadrate	1	50-60	1
EP2	Clarias sp.	urohyale	1	60-70	1
EP2	Clarias sp.	caudal vertebra	2	50-60	2
EP2	Clarias sp.	cranial roof fragment	6		
EP2	Haplotilapiini indet.	basioccipital	1	20-25	1
EP2	Haplotilapiini indet.	caudal vertebra	1	20-25	1
EP2	C. zillii	hyomandibula	1	25-30	1
EP2	Haplotilapiini indet.	hyomandibula	1		
EP2	Haplotilapiini indet.	branchial element	1		
EP2	O. niloticus	opercular	1	20-25	1
EP2	Haplotilapiini indet.	opercular	1	20-25	1
EP2	Haplotilapiini indet.	opercular	1	20-25	1
EP2	Haplotilapiini indet.	opercular	1	15-20	1
EP2	Haplotilapiini indet.	3rd precaudal vertebra	1	15-20	1
EP2	Haplotilapiini indet.	precaudal vertebra	1	20-25	1
EP2	Haplotilapiini indet.	dorsal or anal pterygophore	1	15-20	1
EP2	Haplotilapiini indet.	caudal vertebra	2	15-20	2
EP2	Haplotilapiini indet.	lepidotrich	3		
EP2	Haplotilapiini indet.	precaudal vertebra	3	15-20	3
EP2	Haplotilapiini indet.	cleithrum	4		
EP2	Haplotilapiini indet.	skull roof fragment	6	20-25	1
EP2	O. niloticus	suborbital	1	20-25	2
EP2	C. zillii	1st precaudal vertebra	1	20-25	1
EP2	Clarias sp.	cleithrum	1	60-70	1
EP2	Clarias sp.	coracoid	1	60-70	1
EP2	Clarias sp.	quadrate	1	50-60	1
EP2	Haplotilapiini indet.	anal pterygophore	1	20-25	1
EP2	Clarias sp.	cranial roof fragment	1		
EP2	Haplotilapiini indet.	caudal vertebra	1	15-20	1
EP2	Haplotilapiini indet.	caudal vertebra	1	15-20	1
EP2	Haplotilapiini indet.	neurocranium fragment	1	15-20	1
EP2	Haplotilapiini indet.	parasphenoid	1	20-25	1
EP2	Haplotilapiini indet.	precaudal vertebra	1	15-20	1
EP2	Haplotilapiini indet.	precaudal vertebra	1	15-20	1
EP2	Haplotilapiini indet.	precaudal vertebra	1	15-20	1
EP2	Clarias sp.	cranial roof fragment	1	40-50	1
EP2	Clarias sp.	pectoral spine	1	60-70	1
EP2	Haplotilapiini indet.	cleithrum	1	20-25	1
EP2	Clarias sp.	caudal vertebra	1		
EP2	Clarias sp.	cranial roof fragment	1		
EP2	Clarias sp.	coracoid	2		
EP2	Haplotilapiini indet.	cleithrum	1	15-20	1
EP2	Haplotilapiini indet.	unidentified	1		
EP2	Clarias sp.	cranial roof fragment	1		
EP2	Haplotilapiini indet.	skull roof fragment	1		
EP2	Clarias sp.	cleithrum	1	60-70	1
EP2	Clarias sp.	cleithrum	1		
EP2	Clarias sp.	cranial roof fragment	1		
EP2	Clarias sp.	cranial roof fragment	1	60-70	1
EP2	Clarias sp.	pectoral spine	1	50-60	1
EP2	Clarias sp.	cranial roof fragment	5		
EP2	Haplotilapiini indet.	skull roof fragment	1	20-25	1
EP2	Clarias sp.	cleithrum	1		
EP2	Clarias sp.	epi- & ceratohyale	1	50-60	1
EP2	Clarias sp.	mesethmoid	1	30-40	1

cultural phase	taxon	skeletal element	NISP	SL in cm	number
EP2	Clarias sp.	cranial roof fragment	1	50-60	1
EP2	Clarias sp.	cranial roof fragment	11		
EP2	Clarias sp.	cranial roof fragment	2	40-50	1
EP2	Haplotilapiini indet.	skull roof fragment	1	20-25	1
EP2	Haplotilapiini indet.	opercular	1		
EP2	Haplotilapiini indet.	preopercular	1		
EP2	Haplotilapiini indet.	cleithrum	3	20-25	1
EP2	Clarias sp.	caudal vertebra	1	60-70	1
EP2	Clarias sp.	cranial roof fragment	1		
EP2	Clarias sp.	cranial roof fragment	1	40-50	1
EP2	Haplotilapiini indet.	opercular	1	20-25	1
EP2	Clarias sp.	cranial roof fragment	3		
EP2	Haplotilapiini indet.	skull roof fragment	1		
EP2	Clarias sp.	cranial roof fragment	1		
EP2	Clarias sp.	cranial roof fragment	1		
EP2	Clarias sp.	articular	1	40-50	1
EP2	Clarias sp.	cleithrum	1	50-60	1
EP2	Clarias sp.	cleithrum	1	30-40	1
EP2	Clarias sp.	cranial roof fragment	1		
EP2	Clarias sp.	precaudal vertebra	1	60-70	1
EP2	Clarias sp.	cranial roof fragment	1		
EP2	Clarias sp.	cranial roof fragment	2		
EP2	Clarias sp.	coracoid	1		
EP2	Clarias sp.	cranial roof fragment	1		1
EP2	Clarias sp.	cranial roof fragment	2		
EP2	Haplotilapiini indet.	anal pterygophore	1	20-25	1
EP2	Clarias sp.	cranial roof fragment	1	70-80	1
EP2	Clarias sp.	cranial roof fragment	2		
EP2	O. niloticus	suborbital	1	20-25	1
EP2	O. niloticus	suborbital	1	20-25	1
EP2	Clarias sp.	pectoral spine	1		
EP2	Clarias sp.	cleithrum	1		
EP2	Clarias sp.	pectoral spine	1	30-40	1
EP2	Clarias sp.	cranial roof fragment	6		
EP2	Clarias sp.	cranial roof fragment	3		
EP2	Clarias sp.	cranial roof fragment	1	20-30	1
EP2	Clarias sp.	cranial roof fragment	2	50-60	1
EP2	Clarias sp.	cranial roof fragment	3		
EP2	Haplotilapiini indet.	basipterygium	1	25-30	1
EP2	Haplotilapiini indet.	precaudal vertebra	1	15-20	1
EP2	Clarias sp.	cranial roof fragment	1	60-70	1
EP2	Clarias sp.	coracoid	1		
EP2	Clarias sp.	hypohyale	1		
EP2	Clarias sp.	unidentified	1		
EP2	Clarias sp.	pectoral spine	1	50-60	1
EP2	Clarias sp.	vomerine toothplate	1	50-60	1
EP2	Clarias sp.	caudal vertebra	2	50-60	2
EP2	Clarias sp.	caudal vertebra	2	60-70	2
EP2	Clarias sp.	cleithrum	2		
EP2	Clarias sp.	cranial roof fragment	5		
EP2	Clarias sp.	articular	1	30-40	1
EP2	Clarias sp.	caudal vertebra	1	40-50	1
EP2	Clarias sp.	ceratohyale	1	60-70	1
EP2	Clarias sp.	opercular	1	40-50	1
EP2	Clarias sp.	cranial roof fragment	3		
EP2	Clarias sp.	cleithrum	1		
EP2	Clarias sp.	coracoid	1	40-50	1
EP2	Clarias sp.	coracoid	1	50-60	1

cultural phase	taxon	skeletal element	NISP	SL in cm	number
EP2	Clarias sp.	cranial roof fragment	2		
EP2	Clarias sp.	caudal vertebra	1	30-40	1
EP2	Clarias sp.	cleithrum	1	30-40	1
EP2	Clarias sp.	pectoral spine	1	40-50	1
EP2	Clarias sp.	urohyale	1	30-40	1
EP2	Clarias sp.	cranial roof fragment	5		
EP2	Clarias sp.	ceratohyale	1	50-60	1
EP2	Clarias sp.	coracoid	1	50-60	1
EP2	Clarias sp.	caudal vertebra	3	40-50	3
EP2	Clarias sp.	cranial roof fragment	5		
EP2	Clarias sp.	articular	1		
EP2	Clarias sp.	caudal vertebra	1	30-40	1
EP2	Clarias sp.	caudal vertebra	1	40-50	1
EP2	Clarias sp.	caudal vertebra	1	50-60	1
EP2	Clarias sp.	cleithrum	1	50-60	1
EP2	Clarias sp.	cranial roof fragment	4	40-50	1
EP2	Clarias sp.	caudal vertebra	1	50-60	1
EP2	Clarias sp.	pectoral spine	1	60-70	1
EP2	Clarias sp.	cranial roof fragment	6		
EP2	Clarias sp.	hyomandibula	1	40-50	1
EP2	Clarias sp.	cranial roof fragment	1	40-50	1
EP2	Haplotilapiini indet.	basipterygium	1	20-25	1
EP2	Haplotilapiini indet.	caudal vertebra	1	15-20	1
EP2	Haplotilapiini indet.	mesethmoid	1	20-25	1
EP2	Haplotilapiini indet.	opercular	1	15-20	1
EP2	O. niloticus	opercular	1	20-25	1
EP2	Haplotilapiini indet.	opercular	1	20-25	1
EP2	Haplotilapiini indet.	preopercular	1	15-20	1
EP2	Haplotilapiini indet.	preopercular	1	20-25	1
EP2	Haplotilapiini indet.	dorsal or anal pterygophore	1	20-25	1
EP2	Haplotilapiini indet.	dorsal or anal pterygophore	1	20-25	1
EP2	Haplotilapiini indet.	dorsal or anal pterygophore	1		
EP2	O. niloticus	urohyale	1	15-20	1
EP2	Haplotilapiini indet.	postcleithrum	2	15-20	2
EP2	Haplotilapiini indet.	lepidotrich	3		
EP2	Haplotilapiini indet.	precaudal vertebra	4	15-20	4
EP2	Haplotilapiini indet.	skull roof fragment	5	15-20	1
EP2	Haplotilapiini indet.	cleithrum	7		
EP2	Haplotilapiini indet.	cleithrum	1		
EP2	Haplotilapiini indet.	skull roof fragment	1	20-25	1
EP2	Haplotilapiini indet.	opercular	1	20-25	1
EP2	Haplotilapiini indet.	precaudal vertebra	1	20-25	1
EP2	Haplotilapiini indet.	preopercular	1	20-25	1
EP2	Haplotilapiini indet.	dorsal or anal pterygophore	1	20-25	1
EP2	Haplotilapiini indet.	cleithrum	1		
EP2	Haplotilapiini indet.	2nd precaudal vertebra	1	20-25	1
EP2	Haplotilapiini indet.	subopercular	1	20-25	1
EP2	Haplotilapiini indet.	3rd precaudal vertebra	1	15-20	1
EP2	Haplotilapiini indet.	precaudal vertebra	1	20-25	1
EP2	Haplotilapiini indet.	preopercular	1		
EP2	Haplotilapiini indet.	dorsal or anal pterygophore	1	15-20	1
EP2	Haplotilapiini indet.	dorsal or anal pterygophore	1		
EP2	Haplotilapiini indet.	basipterygium	2	20-25	2
EP2	Haplotilapiini indet.	cleithrum	2	25-30	1
EP2	Haplotilapiini indet.	lepidotrich	2		
EP2	Haplotilapiini indet.	postcleithrum	2	20-25	2
EP2	Haplotilapiini indet.	precaudal vertebra	3	15-20	3
EP2	Haplotilapiini indet.	opercular	4		

cultural phase	taxon	skeletal element	NISP	SL in cm	number
EP2	Haplotilapiini indet.	skull roof fragment	5	20-25	2
EP2	Haplotilapiini indet.	basioccipital	1	15-20	1
EP2	Haplotilapiini indet.	caudal vertebra	1	15-20	1
EP2	C. zillii	hyomandibula	1	15-20	
EP2	Haplotilapiini indet.	opercular	1	15-20	
EP2	Haplotilapiini indet.	dorsal or anal pterygophore	1	20-25	
EP2	Haplotilapiini indet.	lepidotrich	2		
EP2	Haplotilapiini indet.	precaudal vertebra	3	15-20	3
EP2	Haplotilapiini indet.	cleithrum	4		
EP2	Haplotilapiini indet.	dorsal or anal pterygophore	1	15-20	1
EP2	Haplotilapiini indet.	dorsal or anal pterygophore	1	10-15	1
EP2	Haplotilapiini indet.	dorsal or anal pterygophore	1	20-25	1
EP2	O. niloticus	suborbital	1	20-25	1
EP2	Haplotilapiini indet.	caudal vertebra	2	15-20	2
EP2	Haplotilapiini indet.	lepidotrich	2		
EP2	Haplotilapiini indet.	precaudal vertebra	3	15-20	3
EP2	Clarias sp.	caudal vertebra	1	50-60	1
EP2	Clarias sp.	cleithrum	1		
EP2	Clarias sp.	premaxilla	1	50-60	1
EP2	Clarias sp.	cranial roof fragment	7		
EP2	Haplotilapiini indet.	basipterygium	1	20-25	1
EP2	Haplotilapiini indet.	caudal vertebra	1	20-25	1
EP2	Haplotilapiini indet.	cleithrum	1		
EP2	Haplotilapiini indet.	coracoid	1	20-25	1
EP2	C. zillii	dentary	1	30-40	1
EP2	C. zillii	hyomandibula	1	20-25	1
EP2	Haplotilapiini indet.	hyomandibula	1	20-25	1
EP2	Haplotilapiini indet.	skull roof fragment	1	15-20	1
EP2	Haplotilapiini indet.	skull roof fragment	1	20-25	1
EP2	Haplotilapiini indet.	3rd precaudal vertebra	1	25-30	1
EP2	Haplotilapiini indet.	precaudal vertebra	1	20-25	1
EP2	Haplotilapiini indet.	preopercular	1	20-25	1
EP2	Haplotilapiini indet.	dorsal or anal pterygophore	1	15-20	1
EP2	Haplotilapiini indet.	opercular	2		
EP2	Haplotilapiini indet.	precaudal vertebra	2	15-20	2
EP2	Haplotilapiini indet.	caudal vertebra	3	15-20	3
EP2	O. niloticus	cleithrum	1	20-25	1
EP2	Haplotilapiini indet.	interopercular	1	20-25	1
EP2	Haplotilapiini indet.	opercular	1		
EP2	Haplotilapiini indet.	precaudal vertebra	1	20-25	1
EP2	O. niloticus	suborbital	1	20-25	1
EP2	Clarias sp.	caudal vertebra	1	40-50	1
EP2	Clarias sp.	caudal vertebra	1	50-60	1
EP2	Clarias sp.	pectoral spine	1	40-50	1
EP2	Clarias sp.	pectoral spine	1	60-70	1
EP2	Clarias sp.	cranial roof fragment	3		
EP2	Haplotilapiini indet.	dorsal or anal pterygophore	1	15-20	1
EP2	Clarias sp.	cleithrum	1	50-60	1
EP2	Clarias sp.	cleithrum	1	30-40	1
EP2	Clarias sp.	opercular	1	15-20	1
EP2	Clarias sp.	quadrate	1	30-40	1
EP2	Clarias sp.	cranial roof fragment	10		
EP2	Clarias sp.	caudal vertebra	2	40-50	1
EP2	Haplotilapiini indet.	cleithrum	1	15-20	1
EP2	Haplotilapiini indet.	cleithrum	1	20-25	1
EP2	Clarias sp.	articular	1		
EP2	Clarias sp.	cleithrum	1		
EP2	Clarias sp.	cranial roof fragment	2		

cultural phase	taxon	skeletal element	NISP	SL in cm	number
EP2	Haplotilapiini indet.	cleithrum	1		
EP2	Haplotilapiini indet.	skull roof fragment	1		
EP2	Clarias sp.	articular	1	40-50	1
EP2	Clarias sp.	caudal vertebra	1	50-60	1
EP2	Clarias sp.	cleithrum	1	50-60	1
EP2	Clarias sp.	coracoid	1	50-60	1
EP2	Clarias sp.	opercular	1	40-50	1
EP2	Clarias sp.	pectoral spine	1	50-60	1
EP2	Clarias sp.	cranial roof fragment	4	30-40	1
EP2	Clarias sp.	cranial roof fragment	2		
EP2	Haplotilapiini indet.	precaudal vertebra	1	15-20	1
EP2	Clarias sp.	caudal vertebra	1	50-60	1
EP2	Clarias sp.	ceratohyale	1	50-60	1
EP2	Clarias sp.	pectoral spine	1	40-50	1
EP2	Clarias sp.	pectoral spine	1	50-60	1
EP2	Clarias sp.	cranial roof fragment	2		
EP2	Haplotilapiini indet.	cleithrum	2		
EP2	Haplotilapiini indet.	lepidotrich	2		
EP2	Clarias sp.	dentary	1	50-60	1
EP2	Clarias sp.	cranial roof fragment	1	60-70	1
EP2	Clarias sp.	cranial roof fragment	2		
EP2	Clarias sp.	caudal vertebra	1	60-70	1
EP2	Clarias sp.	cranial roof fragment	8		
EP2	Haplotilapiini indet.	caudal vertebra	1	15-20	1
EP2	Haplotilapiini indet.	preopercular	1	15-20	1
EP2	Haplotilapiini indet.	dorsal or anal pterygophore	1	20-25	1
EP2	Haplotilapiini indet.	skull roof fragment	3		
EP2	Clarias sp.	quadrate	1	40-50	1
EP2	Clarias sp.	cranial roof fragment	2		
EP2	Clarias sp.	mesethmoid	1	40-50	1
EP2	Clarias sp.	cranial roof fragment	1	20-30	1
EP2	Clarias sp.	opercular	1	60-70	1
EP2	Clarias sp.	caudal vertebra	2	50-60	1
EP2	Clarias sp.	cranial roof fragment	4		
EP2	Haplotilapiini indet.	cleithrum	1		
EP2	Haplotilapiini indet.	skull roof fragment	1		
EP2	Haplotilapiini indet.	precaudal vertebra	1	20-25	1
EP2	Haplotilapiini indet.	postcleithrum	1		
EP2	Clarias sp.	caudal vertebra	1	40-50	1
EP2	Clarias sp.	cleithrum	1	30-40	1
EP2	Clarias sp.	cranial roof fragment	1	40-50	1
EP2	Clarias sp.	parasphenoid	1	60-70	1
EP2	Clarias sp.	cleithrum	2		
EP2	Clarias sp.	cranial roof fragment	4		
EP2	Haplotilapiini indet.	dorsal or anal pterygophore	1	20-25	1
EP2	Clarias sp.	mesethmoid	1	60-70	1
EP2	Clarias sp.	cranial roof fragment	1	40-50	1
EP2	Clarias sp.	opercular	1	30-40	1
EP2	Clarias sp.	cranial roof fragment	6		
EP2	Haplotilapiini indet.	cleithrum	1		
EP2	Haplotilapiini indet.	opercular	1		
EP2	Clarias sp.	articular	1	40-50	1
EP2	Clarias sp.	articular	1	40-50	1
EP2	Clarias sp.	articular	1	40-50	1
EP2	Clarias sp.	articular	1	40-50	1
EP2	Clarias sp.	articular	1		
EP2	Clarias sp.	dentary	1	40-50	1
EP2	Clarias sp.	dentary	1	50-60	1

cultural phase	taxon	skeletal element	NISP	SL in cm	number
EP2	Clarias sp.	epihyale	1	50-60	1
EP2	Clarias sp.	opercular	1	30-40	1
EP2	Clarias sp.	precaudal vertebra	1	40-50	1
EP2	Clarias sp.	pectoral spine	1	40-50	1
EP2	Clarias sp.	quadrate	1	40-50	1
EP2	Clarias sp.	quadrate	1	40-50	1
EP2	Clarias sp.	quadrate	1	40-50	1
EP2	Clarias sp.	vomerine toothplate	1	40-50	1
EP2	Clarias sp.	cranial roof fragment	11		
EP2	Clarias sp.	caudal vertebra	2	60-70	2
EP2	Clarias sp.	cranial roof fragment	2	50-60	2
EP2	Clarias sp.	cranial roof fragment	3	40-50	3
EP2	Haplotilapiini indet.	opercular	1		
EP2	Haplotilapiini indet.	3rd precaudal vertebra	1	20-25	1
EP2	Haplotilapiini indet.	precaudal vertebra	1	15-20	1
EP2	Haplotilapiini indet.	postcleithrum	1		
EP2	Haplotilapiini indet.	dorsal or anal pterygophore	1	20-25	1
EP2	Haplotilapiini indet.	dorsal or anal pterygophore	1	20-25	1
EP2	Haplotilapiini indet.	dorsal or anal pterygophore	1	20-25	1
EP2	O. niloticus	cleithrum	10		
EP2	Haplotilapiini indet.	skull roof fragment	12		
EP2	Haplotilapiini indet.	lepidotrich	2		
EP2	Haplotilapiini indet.	preopercular	4		
EP2	Clarias sp.	cranial roof fragment	1		
EP2	Clarias sp.	cranial roof fragment	1		
EP2	Clarias sp.	dentary	1		
EP2	Clarias sp.	quadrate	1	30-40	1
EP2	Clarias sp.	cranial roof fragment	2		
EP2	Clarias sp.	caudal vertebra	1	50-60	1
EP2	Clarias sp.	cleithrum	1	30-40	1
EP2	Clarias sp.	cranial roof fragment	1		
EP2	Clarias sp.	articular	1	50-60	1
EP2	Clarias sp.	ceratohyale	1	60-70	1
EP2	Clarias sp.	cleithrum	1		
EP2	Clarias sp.	mesethmoid	1	40-50	1
EP2	Clarias sp.	cranial roof fragment	1	40-50	1
EP2	Clarias sp.	cranial roof fragment	1	60-70	1
EP2	Clarias sp.	pectoral spine	1	40-50	1
EP2	Clarias sp.	coracoid	2		
EP2	Clarias sp.	caudal vertebra	5	50-60	5
EP2	Clarias sp.	cranial roof fragment	7		
EP2	Clarias sp.	articular	1	40-50	1
EP2	Clarias sp.	articular	1	40-50	1
EP2	Clarias sp.	cleithrum	1	40-50	1
EP2	Clarias sp.	coracoid	1	30-40	1
EP2	Clarias sp.	coracoid	1		
EP2	Clarias sp.	cranial roof fragment	3		
EP2	Clarias sp.	ceratohyale	1	40-50	1
EP2	Clarias sp.	epihyale	1	40-50	1
EP2	Clarias sp.	cranial roof fragment	5		
EP2	Clarias sp.	caudal vertebra	8	50-60	1
EP2	Clarias sp.	caudal vertebra	1	50-60	1
EP2	Clarias sp.	caudal vertebra	1	60-70	1
EP2	Clarias sp.	cleithrum	1	30-40	1
EP2	Clarias sp.	pectoral spine	1	60-70	1
EP2	Clarias sp.	cranial roof fragment	5		
EP2	Clarias sp.	articular	1	30-40	1
EP2	Clarias sp.	caudal vertebra	1	40-50	1

cultural phase	taxon	skeletal element	NISP	SL in cm	number
EP2	Clarias sp.	ceratohyale	1	40-50	1
EP2	Clarias sp.	cleithrum	1	30-40	1
EP2	Clarias sp.	hyomandibula	1	50-60	1
EP2	Clarias sp.	unidentified	1		
EP2	Clarias sp.	pectoral spine	1	50-60	1
EP2	Clarias sp.	pectoral spine	1	50-60	1
EP2	Clarias sp.	pectoral spine	1		
EP2	Clarias sp.	cleithrum	2		
EP2	Clarias sp.	cranial roof fragment	8	50-60	1
EP2	Clarias sp.	articular	1	60-70	1
EP2	Clarias sp.	basioccipital	1	30-40	1
EP2	Clarias sp.	caudal vertebra	1	60-70	1
EP2	Clarias sp.	cleithrum	1		
EP2	Clarias sp.	dentary	1	30-40	1
EP2	Clarias sp.	dentary	1	30-40	1
EP2	Clarias sp.	dentary	1	30-40	1
EP2	Clarias sp.	precaudal vertebra	1	60-70	1
EP2	Clarias sp.	pectoral spine	1	50-60	1
EP2	Clarias sp.	pectoral spine	1		
EP2	Clarias sp.	quadrate	1	30-40	1
EP2	Clarias sp.	cranial roof fragment	2		
EP2	Clarias sp.	caudal vertebra	5	50-60	5
EP2	Clarias sp.	cranial roof fragment	6	20-30	2
EP2	Clarias sp.	articular	1	60-70	1
EP2	Clarias sp.	caudal vertebra	1	40-50	1
EP2	Clarias sp.	cleithrum	1	30-40	1
EP2	Clarias sp.	epihyale	1	50-60	1
EP2	Clarias sp.	epihyale	1	60-70	1
EP2	Clarias sp.	hyomandibula	1		
EP2	Clarias sp.	pectoral spine	1	60-70	1
EP2	Clarias sp.	premaxilla	1	60-70	1
EP2	Clarias sp.	cleithrum	2		
EP2	Clarias sp.	caudal vertebra	3	50-60	3
EP2	Clarias sp.	cranial roof fragment	7		
EP2	Clarias sp.	cranial roof fragment	1	30-40	1
EP2	Clarias sp.	cranial roof fragment	2		
EP2	Clarias sp.	mesethmoid	1	60-70	1
EP2	Clarias sp.	mesethmoid	1		
EP2	Clarias sp.	cranial roof fragment	3		
EP2	Clarias sp.	cranial roof fragment	1	50-60	1
EP2	Clarias sp.	cranial roof fragment	1		
EP2	Haplotilapiini indet.	cleithrum	1		
EP2	Haplotilapiini indet.	interopercular	1	20-25	1
EP2	Haplotilapiini indet.	fin spine	1		
EP2	Haplotilapiini indet.	skull roof fragment	1		
EP2	Haplotilapiini indet.	preopercular	1	20-25	1
EP2	Haplotilapiini indet.	opercular	1		
EP2	Haplotilapiini indet.	opercular	1	20-25	1
EP2	Haplotilapiini indet.	supracleithrum	1	15-20	1
EP2	Haplotilapiini indet.	dorsal or anal pterygophore	1	20-25	1
EP2	Haplotilapiini indet.	supracleithrum	1	15-20	1
EP2	Haplotilapiini indet.	opercular	1		
EP2	Haplotilapiini indet.	cleithrum	3		
EP2	O. niloticus	opercular	1	15-20	1
EP2	Haplotilapiini indet.	opercular	1		
EP2	Haplotilapiini indet.	parasphenoid	1		
EP2	Haplotilapiini indet.	cleithrum	3		
EP2	Haplotilapiini indet.	opercular	1	15-20	1

cultural phase	taxon	skeletal element	NISP	SL in cm	number
EP2	Haplotilapiini indet.	opercular	1	15-20	1
EP2	Haplotilapiini indet.	dorsal or anal pterygophore	1	20-25	1
EP2	Haplotilapiini indet.	cleithrum	2		
EP2	Haplotilapiini indet.	skull roof fragment	2	20-25	1
EP2	Haplotilapiini indet.	cleithrum	1		
EP2	Haplotilapiini indet.	mesethmoid	1	20-25	1
EP2	Haplotilapiini indet.	opercular	1		
EP2	Haplotilapiini indet.	skull roof fragment	2		
EP2	Haplotilapiini indet.	skull roof fragment	3	15-20	3
EP2	Haplotilapiini indet.	cleithrum	1		
EP2	Haplotilapiini indet.	fin spine	1		
EP2	Haplotilapiini indet.	dorsal or anal pterygophore	1	20-25	1
EP2	Haplotilapiini indet.	precaudal vertebra	1	15-20	1
EP2	Haplotilapiini indet.	dorsal or anal pterygophore	1	20-25	1
EP2	Clarias sp.	cranial roof fragment	2		
EP2	Clarias sp.	cranial roof fragment	1	50-60	1
EP2	Clarias sp.	cranial roof fragment	7		
EP2	Clarias sp.	caudal vertebra	1	50-60	1
EP2	Clarias sp.	cleithrum	1	20-30	1
EP2	Clarias sp.	cranial roof fragment	1	60-70	1
EP2	Clarias sp.	cranial roof fragment	10		
EP2	Clarias sp.	cleithrum	1		
EP2	Clarias sp.	precaudal vertebra	1	60-70	1
EP2	Clarias sp.	cranial roof fragment	3	60-70	1
EP2	Haplotilapiini indet.	cleithrum	1		
EP2	Haplotilapiini indet.	precaudal vertebra	1	20-25	1
EP2	Haplotilapiini indet.	opercular	1		
EP2	Haplotilapiini indet.	dorsal or anal pterygophore	1	20-25	1
EP2	Clarias sp.	articular	1	80-90	1
EP2	Clarias sp.	cleithrum	1	50-60	1
EP2	Clarias sp.	coracoid	1		
EP2	Clarias sp.	cranial roof fragment	3		
EP2	O. niloticus	hyomandibula	1	20-25	1
EP2	Haplotilapiini indet.	fin spine	1		
EP2	O. niloticus	opercular	1	15-20	1
EP2	O. niloticus	opercular	1	20-25	1
EP2	Haplotilapiini indet.	2nd precaudal vertebra	1	25-30	1
EP2	Haplotilapiini indet.	precaudal vertebra	1	15-20	1
EP2	O. niloticus	premaxilla	1	25-30	1
EP2	Haplotilapiini indet.	dorsal or anal pterygophore	1	15-20	1
EP2	Haplotilapiini indet.	dorsal or anal pterygophore	1	20-25	1
EP2	Haplotilapiini indet.	cleithrum	2		
EP2	Clarias sp.	cleithrum	1	20-30	1
EP2	Clarias sp.	hyomandibula	1		
EP2	Clarias sp.	precaudal vertebra	1	50-60	1
EP2	Clarias sp.	caudal vertebra	3	50-60	3
EP2	Clarias sp.	cranial roof fragment	3		
EP2	Clarias sp.	cranial roof fragment	5	30-40	2
EP2	Clarias sp.	cleithrum	1		
EP2	Clarias sp.	precaudal vertebra	1	50-60	1
EP2	Clarias sp.	pectoral spine	1	40-50	1
EP2	Clarias sp.	coracoid	2		
EP2	Clarias sp.	cranial roof fragment	2		
EP2	Clarias sp.	caudal vertebra	1	50-60	1
EP2	Clarias sp.	epihyale	1		
EP2	Clarias sp.	hyomandibula	1	40-50	1
EP2	Clarias sp.	cranial roof fragment	1	50-60	1
EP2	Clarias sp.	pectoral spine	1	30-40	1

cultural phase	taxon	skeletal element	NISP	SL in cm	number
EP2	Clarias sp.	pectoral spine	1		
EP2	Clarias sp.	cleithrum	3	60-70	1
EP2	Clarias sp.	cranial roof fragment	9		
EP2	Clarias sp.	articular	1		
EP2	Clarias sp.	caudal vertebra	1	40-50	1
EP2	Clarias sp.	dentary	1		
EP2	Clarias sp.	cranial roof fragment	1	20-30	1
EP2	Clarias sp.	caudal vertebra	2	60-70	2
EP2	Clarias sp.	cranial roof fragment	2	50-60	2
EP2	Clarias sp.	cranial roof fragment	8		
EP2	O. niloticus	mesethmoid	1	25-30	1
EP2	Haplotilapiini indet.	dorsal or anal pterygophore	1	15-20	1
EP2	Haplotilapiini indet.	caudal vertebra	2	15-20	2
EP2	Haplotilapiini indet.	cleithrum	2		
EP2	Haplotilapiini indet.	basipterygium	1	15-20	1
EP2	Haplotilapiini indet.	cleithrum	1		
EP2	Haplotilapiini indet.	preopercular	1		
EP2	Haplotilapiini indet.	fin spine	1		
EP2	Haplotilapiini indet.	precaudal vertebra	1	15-20	1
EP2	Haplotilapiini indet.	dorsal or anal pterygophore	1	15-20	1
EP2	Haplotilapiini indet.	dorsal or anal pterygophore	1	15-20	1
EP2	Haplotilapiini indet.	caudal vertebra	1	25-30	1
EP2	Haplotilapiini indet.	fin spine	1		
EP2	Haplotilapiini indet.	opercular	1		
EP2	Haplotilapiini indet.	supracleithrum	1	20-25	1
EP2	Haplotilapiini indet.	cleithrum	2		
EP2	Haplotilapiini indet.	skull roof fragment	2	15-20	1
EP2	Haplotilapiini indet.	caudal vertebra	3	15-20	3
EP2	Haplotilapiini indet.	precaudal vertebra	3	15-20	3
EP2	Clarias sp.	articular	1	40-50	1
EP2	Clarias sp.	basioccipital	1	70-80	1
EP2	Clarias sp.	cleithrum	1	30-40	1
EP2	Clarias sp.	cleithrum	1	50-60	1
EP2	Clarias sp.	cleithrum	1	50-60	1
EP2	Clarias sp.	coracoid	1		
EP2	Clarias sp.	cranial roof fragment	1	30-40	1
EP2	Clarias sp.	cranial roof fragment	1	40-50	1
EP2	Clarias sp.	pectoral spine	1	50-60	1
EP2	Clarias sp.	pectoral spine	1	60-70	1
EP2	Clarias sp.	quadrate	1	30-40	1
EP2	Clarias sp.	cranial roof fragment	13		
EP2	Clarias sp.	cranial roof fragment	3	60-70	3
EP2	Clarias sp.	ceratohyale	1	30-40	1
EP2	Clarias sp.	cleithrum	1	30-40	1
EP2	Clarias sp.	cranial roof fragment	4	50-60	1
EP2	Clarias sp.	caudal vertebra	1	60-70	1
EP2	Clarias sp.	cleithrum	1	40-50	1
EP2	Clarias sp.	pectoral spine	1	40-50	1
EP2	Clarias sp.	pectoral spine	1	40-50	1
EP2	Clarias sp.	vomerine toothplate	1	50-60	1
EP2	Clarias sp.	cranial roof fragment	12		
EP2	Clarias sp.	coracoid	2		
EP2	Clarias sp.	precaudal vertebra	2	60-70	2
EP2	Clarias sp.	pectoral spine	2		
EP2	Clarias sp.	cleithrum	3		
EP2	Clarias sp.	articular	1	40-50	1
EP2	Clarias sp.	cleithrum	1		
EP2	Clarias sp.	coracoid	1		

cultural phase	taxon	skeletal element	NISP	SL in cm	number
EP2	Clarias sp.	dentary	1	50-60	1
EP2	Clarias sp.	opercular	1	40-50	1
EP2	Clarias sp.	precaudal vertebra	1	50-60	1
EP2	Clarias sp.	caudal vertebra	4	50-60	3
EP2	Clarias sp.	cranial roof fragment	4		
EP2	Haplotilapiini indet.	opercular	1	20-25	1
EP2	Haplotilapiini indet.	opercular	1	15-20	1
EP2	Haplotilapiini indet.	dorsal or anal pterygophore	1	20-25	1
EP2	Haplotilapiini indet.	cleithrum	3		
EP2	Haplotilapiini indet.	mesethmoid	1	15-20	1
EP2	O. niloticus	mesethmoid	1	20-25	1
EP2	Haplotilapiini indet.	skull roof fragment	1	15-20	1
EP2	Haplotilapiini indet.	skull roof fragment	1	20-25	1
EP2	Haplotilapiini indet.	opercular	1	15-20	1
EP2	Haplotilapiini indet.	opercular	1	15-20	1
EP2	Haplotilapiini indet.	opercular	1	20-25	1
EP2	Haplotilapiini indet.	opercular	1	15-20	1
EP2	Haplotilapiini indet.	opercular	1	20-25	1
EP2	Haplotilapiini indet.	opercular	1	10-15	1
EP2	Haplotilapiini indet.	opercular	1	15-20	1
EP2	Haplotilapiini indet.	opercular	1		
EP2	Haplotilapiini indet.	3rd precaudal vertebra	1	20-25	1
EP2	Haplotilapiini indet.	preopercular	1	15-20	1
EP2	Haplotilapiini indet.	dorsal or anal pterygophore	1	20-25	1
EP2	Haplotilapiini indet.	dorsal or anal pterygophore	1	20-25	1
EP2	Haplotilapiini indet.	dorsal or anal pterygophore	1	20-25	1
EP2	Haplotilapiini indet.	lepidotrich	2		
EP2	Haplotilapiini indet.	dorsal or anal pterygophore	2		
EP2	Haplotilapiini indet.	supracleithrum	2	20-25	2
EP2	Haplotilapiini indet.	skull roof fragment	4		
EP2	Haplotilapiini indet.	cleithrum	8		
EP2	Haplotilapiini indet.	caudal vertebra	1	15-20	1
EP2	C. zillii	mesethmoid	1	20-25	1
EP2	Haplotilapiini indet.	mesethmoid	1	20-25	1
EP2	Haplotilapiini indet.	mesethmoid	1		
EP2	Haplotilapiini indet.	opercular	1	15-20	1
EP2	Haplotilapiini indet.	opercular	1		
EP2	Haplotilapiini indet.	dorsal or anal pterygophore	1	15-20	1
EP2	Haplotilapiini indet.	dorsal or anal pterygophore	1	20-25	1
EP2	Haplotilapiini indet.	dorsal or anal pterygophore	1	15-20	1
EP2	Haplotilapiini indet.	dorsal or anal pterygophore	1	15-20	1
EP2	Haplotilapiini indet.	dorsal or anal pterygophore	1	20-25	1
EP2	Haplotilapiini indet.	dorsal or anal pterygophore	1	15-20	1
EP2	Haplotilapiini indet.	supracleithrum	1	15-20	1
EP2	Haplotilapiini indet.	lepidotrich	2		
EP2	Haplotilapiini indet.	basipterygium	3	15-20	3
EP2	Haplotilapiini indet.	cleithrum	5		
EP2	Haplotilapiini indet.	skull roof fragment	5		
EP2	Haplotilapiini indet.	precaudal vertebra	5	15-20	5
EP2	C. zillii	1st precaudal vertebra	1	20-25	1
EP2	O. niloticus	1st precaudal vertebra	1	25-30	1
EP2	C. zillii	1st precaudal vertebra	1	20-25	1
EP2	Clarias sp.	caudal vertebra	1	60-70	1
EP2	Clarias sp.	cleithrum	1	30-40	1
EP2	Clarias sp.	coracoid	1	30-40	1
EP2	Clarias sp.	hyomandibula	1	40-50	1
EP2	Clarias sp.	cranial roof fragment	1		
EP2	Haplotilapiini indet.	preopercular	1	15-20	1

cultural phase	taxon	skeletal element	NISP	SL in cm	number
EP2	Haplotilapiini indet.	cleithrum	3		
EP2	Clarias sp.	caudal vertebra	1	50-60	1
EP2	Clarias sp.	cleithrum	1		
EP2	Clarias sp.	dentary	1	50-60	1
EP2	Clarias sp.	cranial roof fragment	9		
EP2	O. niloticus	hyomandibula	1	20-25	1
EP2	C. zillii	hyomandibula	1	20-25	1
EP2	Haplotilapiini indet.	skull roof fragment	1		
EP2	Haplotilapiini indet.	opercular	1		
EP2	Haplotilapiini indet.	postcleithrum	1	15-20	1
EP2	Haplotilapiini indet.	dorsal or anal pterygophore	1	15-20	1
EP2	Haplotilapiini indet.	caudal vertebra	2	15-20	2
EP2	Haplotilapiini indet.	cleithrum	4		
MP1	Clarias sp.	caudal vertebra	1	40-50	1
MP1	Clarias sp.	caudal vertebra	1	60-70	1
MP1	Clarias sp.	coracoid	1		
MP1	Clarias sp.	coracoid	1		
MP1	Clarias sp.	hyomandibula	1		
MP1	Clarias sp.	cranial roof fragment	1		
MP1	Clarias sp.	cranial roof fragment	1	60-70	1
MP1	Clarias sp.	vomerine toothplate	1	50-60	1
MP1	Clarias sp.	caudal vertebra	2	50-60	2
MP1	Clarias sp.	caudal vertebra	2	40-50	2
MP1	Clarias sp.	coracoid	2		
MP1	Clarias sp.	cranial roof fragment	2		
MP1	Clarias sp.	cranial roof fragment	2	60-70	1
MP1	Clarias sp.	cranial roof fragment	2		
MP1	Clarias sp.	cleithrum	3		
MP1	Clarias sp.	cranial roof fragment	3		
MP1	Clarias sp.	cranial roof fragment	4		
MP1	Haplotilapiini indet.	basipterygium	1	15-20	1
MP1	Haplotilapiini indet.	caudal vertebra	1	15-20	1
MP1	Haplotilapiini indet.	caudal vertebra	1	15-20	1
MP1	O. niloticus	hyomandibula	1	20-25	1
MP1	Haplotilapiini indet.	lepidotrich	1		
MP1	O. niloticus	opercular	1	20-25	1
MP1	Haplotilapiini indet.	opercular	1	20-25	1
MP1	Haplotilapiini indet.	parasphenoid	1	15-20	1
MP1	Haplotilapiini indet.	2nd precaudal vertebra	1	15-20	1
MP1	Haplotilapiini indet.	3rd precaudal vertebra	1	30-40	1
MP1	Haplotilapiini indet.	precaudal vertebra	1	20-25	1
MP1	Haplotilapiini indet.	dorsal or anal pterygophore	1	15-20	1
MP1	Haplotilapiini indet.	dorsal or anal dorsal or anal pterygophore	1	20-25	1
MP1	Haplotilapiini indet.	dorsal or anal pterygophore	1		
MP1	Haplotilapiini indet.	subopercular	1		
MP1	Haplotilapiini indet.	supracleithrum	1	15-20	1
MP1	Haplotilapiini indet.	mesethmoid	1	25-30	1
MP1	Haplotilapiini indet.	caudal vertebra	2	15-20	2
MP1	Haplotilapiini indet.	opercular	2		
MP1	Haplotilapiini indet.	cleithrum	3		
MP1	Haplotilapiini indet.	skull roof fragment	5	20-25	1
MP1	Haplotilapiini indet.	caudal vertebra	1	15-20	1
MP1	Haplotilapiini indet.	opercular	1		
MP1	Clarias sp.	articular	1	70-80	1
MP1	Clarias sp.	cranial roof fragment	1		
MP1	Clarias sp.	pectoral spine	1	60-70	1
MP1	Clarias sp.	quadrate	1	70-80	1
MP1	Clarias sp.	cleithrum	2		

cultural phase	taxon	skeletal element	NISP	SL in cm	number
MP1	Haplotilapiini indet.	cleithrum	1	15-20	1
MP1	Clarias sp.	cranial roof fragment	1		
MP1	Clarias sp.	cranial roof fragment	1		
MP1	Haplotilapiini indet.	fin spine	1		
MP1	Haplotilapiini indet.	dorsal or anal pterygophore	1	20-25	1
MP1	Haplotilapiini indet.	1st precaudal vertebra	1	15-20	1
MP1	Clarias sp.	urohyale	1		
MP1	Haplotilapiini indet.	dorsal or anal pterygophore	1		
MP1	Haplotilapiini indet.	dorsal or anal pterygophore	1		
MP1	Clarias sp.	caudal vertebra	1	40-50	1
MP1	Clarias sp.	caudal vertebra	1	60-70	1
MP1	Clarias sp.	cranial roof fragment	1	50-60	1
MP1	Clarias sp.	cranial roof fragment	1	60-70	1
MP1	Clarias sp.	mesethmoid	1	60-70	1
MP1	Clarias sp.	cleithrum	2		
MP1	Clarias sp.	cranial roof fragment	3		
MP1	Haplotilapiini indet.	caudal vertebra	1	15-20	1
MP1	Haplotilapiini indet.	dorsal or anal pterygophore	1	25-30	1
MP1	Clarias sp.	cleithrum	1		
MP1	Clarias sp.	cranial roof fragment	1		
MP1	Clarias sp.	cranial roof fragment	1	30-40	1
MP1	Clarias sp.	cranial roof fragment	4		
MP1	Haplotilapiini indet.	caudal vertebra	1	20-25	1
MP1	Haplotilapiini indet.	opercular	1		
MP1	Clarias sp.	opercular	1	40-50	1
MP1	Clarias sp.	precaudal vertebra	1	50-60	1
MP1	Clarias sp.	cranial roof fragment	3		
MP1	Haplotilapiini indet.	ceratohyale	1	20-25	1
MP1	Haplotilapiini indet.	precaudal vertebra	1	15-20	1
MP1	Haplotilapiini indet.	caudal vertebra	2	20-25	2
MP1	Clarias sp.	coracoid	1	60-70	1
MP1	Clarias sp.	cranial roof fragment	1	50-60	1
MP1	Clarias sp.	cranial roof fragment	1	70-80	1
MP1	Clarias sp.	precaudal vertebra	1	60-70	1
MP1	Clarias sp.	quadrate	1	60-70	1
MP1	Clarias sp.	caudal vertebra	2	50-60	2
MP1	Clarias sp.	cranial roof fragment	8		
MP1	Haplotilapiini indet.	caudal vertebra	1	15-20	1
MP1	O. niloticus	hyomandibula	1	20-25	1
MP1	Haplotilapiini indet.	opercular	1		
MP1	Clarias sp.	basipterygium	1	40-50	1
MP1	Clarias sp.	cleithrum	1	30-40	1
MP1	Clarias sp.	cleithrum	1		
MP1	Clarias sp.	cranial roof fragment	1		
MP1	Clarias sp.	cranial roof fragment	1	40-50	1
MP1	Clarias sp.	precaudal vertebra	1	40-50	1
MP1	Clarias sp.	caudal vertebra	2	40-50	2
MP1	Clarias sp.	caudal vertebra	2	50-60	2
MP1	Clarias sp.	cranial roof fragment	5		
MP1	Haplotilapiini indet.	basipterygium	1	15-20	1
MP1	Haplotilapiini indet.	caudal vertebra	1	15-20	1
MP1	Haplotilapiini indet.	cleithrum	1		
MP1	Haplotilapiini indet.	skull roof fragment	1		
MP1	Haplotilapiini indet.	precaudal vertebra	1	15-20	1
MP1	Haplotilapiini indet.	precaudal vertebra	1	20-25	1
MP1	Haplotilapiini indet.	dorsal or anal pterygophore	1	25-30	1
MP1	Clarias sp.	mesethmoid	1	40-50	1
MP1	Clarias sp.	vomerine toothplate	1	60-70	1

cultural phase	taxon	skeletal element	NISP	SL in cm	number
MP1	Clarias sp.	cleithrum	2		
MP1	Clarias sp.	cranial roof fragment	5		
MP1	Haplotilapiini indet.	caudal vertebra	1	20-25	1
MP1	Haplotilapiini indet.	skull roof fragment	1		
MP1	O. niloticus	hyomandibula	1	15-20	1
MP1	Haplotilapiini indet.	preopercular	1	20-25	1
MP1	Haplotilapiini indet.	dorsal or anal pterygophore	1	15-20	1
MP1	Clarias sp.	cranial roof fragment	1	40-50	1
MP1	Clarias sp.	cranial roof fragment	1	60-70	1
MP1	Clarias sp.	hyomandibula	1	30-40	1
MP1	Clarias sp.	caudal vertebra	3	60-70	3
MP1	Clarias sp.	cleithrum	3		
MP1	Clarias sp.	cranial roof fragment	4		
MP1	Haplotilapiini indet.	precaudal vertebra	1	20-25	1
MP1	Haplotilapiini indet.	3rd precaudal vertebra	1	25-30	1
MP1	Haplotilapiini indet.	cleithrum	2		
MP1	Haplotilapiini indet.	1st precaudal vertebra	1	25-30	1
MP1	Clarias sp.	basioccipital	1	50-60	1
MP1	Clarias sp.	basioccipital	1	30-40	1
MP1	Clarias sp.	basioccipital	1	10-20	1
MP1	Clarias sp.	cleithrum	1	50-60	1
MP1	Clarias sp.	cleithrum	1		
MP1	Clarias sp.	coracoid	1	40-50	1
MP1	Clarias sp.	coracoid	1	50-60	1
MP1	Clarias sp.	epihyale	1	40-50	1
MP1	Clarias sp.	hyomandibula	1	30-40	1
MP1	Clarias sp.	hyomandibula	1	40-50	1
MP1	Clarias sp.	hyomandibula	1	40-50	1
MP1	Clarias sp.	hyomandibula	1	40-50	1
MP1	Clarias sp.	mesethmoid	1	20-30	1
MP1	Clarias sp.	palatinum	1	60-70	1
MP1	Clarias sp.	precaudal vertebra	1	40-50	1
MP1	Clarias sp.	precaudal vertebra	1	50-60	1
MP1	Clarias sp.	caudal vertebra	2	40-50	2
MP1	Clarias sp.	cranial roof fragment	2	20-30	2
MP1	Clarias sp.	precaudal vertebra	2	60-70	2
MP1	Clarias sp.	caudal vertebra	3	60-70	3
MP1	Clarias sp.	cranial roof fragment	3	50-60	3
MP1	Clarias sp.	caudal vertebra	4	50-60	4
MP1	Clarias sp.	cranial roof fragment	7	60-70	7
MP1	Clarias sp.	cranial roof fragment	9		
MP1	Haplotilapiini indet.	cleithrum	1		
MP1	O. niloticus	premaxilla	1	20-25	1
MP1	O. niloticus	1st precaudal vertebra	1	30-35	1
MP1	Clarias sp.	caudal vertebra	1	60-70	1
MP1	Clarias sp.	cranial roof fragment	1	50-60	1
MP1	Clarias sp.	unidentified	1		
MP1	Clarias sp.	vomerine toothplate	1	50-60	1
MP1	Clarias sp.	cranial roof fragment	2		
MP1	Clarias sp.	cranial roof fragment	4	60-70	4
MP1	Clarias sp.	cranial roof fragment	1		
MP1	Haplotilapiini indet.	caudal vertebra	1	15-20	1
MP1	Haplotilapiini indet.	interopercular	1		
MP1	Haplotilapiini indet.	precaudal vertebra	1	20-25	1
MP1	Clarias sp.	caudal vertebra	1	60-70	1
MP1	Clarias sp.	cranial roof fragment	1		
MP1	Clarias sp.	cranial roof fragment	1		
MP1	Clarias sp.	Weberian apparatus	1	60-70	1

cultural phase	taxon	skeletal element	NISP	SL in cm	number
MP1	Clarias sp.	Weberian apparatus	1	50-60	1
MP1	Haplotilapiini indet.	premaxilla	1	20-25	1
MP1	Haplotilapiini indet.	scapula	1	20-25	1
MP1	Clarias sp.	epihyale	1	60-70	1
MP1	Clarias sp.	cranial roof fragment	1		
MP1	Clarias sp.	ceratohyale	1		
MP1	Clarias sp.	cleithrum	1		
MP1	Clarias sp.	unidentified	1		
MP1	Clarias sp.	caudal vertebra	1	50-60	1
MP1	Clarias sp.	cranial roof fragment	1		
MP1	Clarias sp.	precaudal vertebra	1	70-80	1
MP1	Clarias sp.	cranial roof fragment	7		
MP1	Clarias sp.	caudal vertebra	1	80-90	1
MP1	Clarias sp.	cleithrum	1		
MP1	Clarias sp.	cranial roof fragment	1		
MP1	Clarias sp.	precaudal vertebra	2	15-20	1
MP1	Clarias sp.	caudal vertebra	1	50-60	1
MP1	Clarias sp.	cleithrum	1		
MP1	Clarias sp.	coracoid	1	40-50	1
MP1	Clarias sp.	cranial roof fragment	1		
MP1	Clarias sp.	cranial roof fragment	3		
MP1	Clarias sp.	cranial roof fragment	4		
MP1	Haplotilapiini indet.	cleithrum	1	20-25	1
MP1	Haplotilapiini indet.	cleithrum	1	15-20	1
MP1	Haplotilapiini indet.	cleithrum	1	20-25	1
MP1	Clarias sp.	caudal vertebra	1	60-70	1
MP1	Clarias sp.	preopercular	1		
MP1	Clarias sp.	urohyale	1	15-20	1
MP1	Clarias sp.	cranial roof fragment	2		
MP1	Clarias sp.	cranial roof fragment	4		
MP1	Clarias sp.	cranial roof fragment	2		
MP1	Clarias sp.	cranial roof fragment	1		
MP1	Clarias sp.	articular	1	30-40	1
MP1	Clarias sp.	cleithrum	1	30-40	1
MP1	Clarias sp.	opercular	1	30-40	1
MP1	Clarias sp.	precaudal vertebra	1	40-50	1
MP1	Clarias sp.	pectoral spine	1	40-50	1
MP1	Clarias sp.	pectoral spine	1	50-60	1
MP1	Clarias sp.	caudal vertebra	3	40-50	3
MP1	Clarias sp.	articular	1	50-60	1
MP1	Clarias sp.	caudal vertebra	1	40-50	1
MP1	Clarias sp.	cleithrum	1		
MP1	Clarias sp.	caudal vertebra	2	50-60	2
MP1	Clarias sp.	cranial roof fragment	8	60-70	1
MP1	Haplotilapiini indet.	articular	1		
MP1	Haplotilapiini indet.	opercular	1		
MP1	Haplotilapiini indet.	postcleithrum	1	15-20	1
MP1	Haplotilapiini indet.	dorsal or anal pterygophore	1	15-20	1
MP1	Haplotilapiini indet.	caudal vertebra	2	15-20	2
MP1	Haplotilapiini indet.	opercular	1	20-25	1
MP1	Haplotilapiini indet.	opercular	1	20-25	1
MP1	Haplotilapiini indet.	precaudal vertebra	1	15-20	1
MP1	Haplotilapiini indet.	caudal vertebra	2	15-20	2
MP1	Haplotilapiini indet.	cleithrum	2		
MP1	Clarias sp.	basioccipital	1		
MP1	Clarias sp.	caudal vertebra	1	50-60	1
MP1	Clarias sp.	cleithrum	1		
MP1	Clarias sp.	cranial roof fragment	1	40-50	1

cultural phase	taxon	skeletal element	NISP	SL in cm	number
MP1	Clarias sp.	vomerine toothplate	1	50-60	1
MP1	Clarias sp.	cranial roof fragment	4		
MP1	Haplotilapiini indet.	cleithrum	1		
MP1	Haplotilapiini indet.	skull roof fragment	1	20-25	1
MP1	O. niloticus	opercular	1	15-20	1
MP1	O. niloticus	urohyale	1	20-25	1
MP1	Clarias sp.	cranial roof fragment	2		
MP1	Clarias sp.	cleithrum	1	40-50	1
MP1	Clarias sp.	cranial roof fragment	8		
MP1	Clarias sp.	ceratohyale	1		
MP1	Clarias sp.	coracoid	1	50-60	1
MP1	Clarias sp.	cranial roof fragment	1	50-60	1
MP1	Clarias sp.	cranial roof fragment	1	70-80	1
MP1	Clarias sp.	pectoral spine	1	40-50	1
MP1	Clarias sp.	pectoral spine	1	50-60	1
MP1	Clarias sp.	subopercular	1		
MP1	Clarias sp.	cleithrum	3		
MP1	Clarias sp.	cranial roof fragment	8		
MP1	Clarias sp.	cleithrum	1		
MP1	Clarias sp.	cranial roof fragment	1		
MP1	Clarias sp.	quadrate	1	60-70	1
MP1	Clarias sp.	cranial roof fragment	4		
MP1	Clarias sp.	articular	1	20-30	1
MP1	Clarias sp.	caudal vertebra	1	60-70	1
MP1	Clarias sp.	cranial roof fragment	5	60-70	1
MP1	Haplotilapiini indet.	opercular	1		
MP1	Haplotilapiini indet.	cleithrum	1	20-25	1
MP1	Haplotilapiini indet.	preopercular	1	20-25	1
MP1	Haplotilapiini indet.	cleithrum	1		
MP1	Haplotilapiini indet.	dorsal or anal pterygophore	1	20-25	1
MP1	Haplotilapiini indet.	cleithrum	1		
MP1	Haplotilapiini indet.	opercular	1	15-20	1
MP1	Haplotilapiini indet.	cleithrum	1		
MP1	Haplotilapiini indet.	caudal vertebra	1	15-20	1
MP1	Haplotilapiini indet.	opercular	1	15-20	1
MP1	Haplotilapiini indet.	supracleithrum	1	15-20	1
MP2	Clarias sp.	cleithrum	1		
MP2	Clarias sp.	cranial roof fragment	3		
MP2	Haplotilapiini indet.	unidentified	1		
MP2	Haplotilapiini indet.	fin spine	1		
MP2	Haplotilapiini indet.	precaudal vertebra	1	15-20	1
MP2	Clarias sp.	articular	1	30-40	1
MP2	Clarias sp.	caudal vertebra	1	40-50	1
MP2	Clarias sp.	cleithrum	1		
MP2	Clarias sp.	dentary	1	60-70	1
MP2	Clarias sp.	precaudal vertebra	1	50-60	1
MP2	Clarias sp.	cranial roof fragment	43		
MP2	Haplotilapiini indet.	fin spine	1		
MP2	Haplotilapiini indet.	opercular	1	15-20	1
MP2	Haplotilapiini indet.	opercular	1	20-25	1
MP2	Haplotilapiini indet.	dorsal or anal pterygophore	1	15-20	1
MP2	Haplotilapiini indet.	quadrate	1	20-30	1
MP2	Haplotilapiini indet.	caudal vertebra	3	15-20	3
MP2	Haplotilapiini indet.	skull roof fragment	3		
MP2	Clarias sp.	caudal vertebra	1	60-70	1
MP2	Clarias sp.	coracoid	1	30-40	1
MP2	Clarias sp.	dentary	1	60-70	1
MP2	Clarias sp.	epihyale	1	40-50	1

cultural phase	taxon	skeletal element	NISP	SL in cm	number
MP2	Clarias sp.	precaudal vertebra	1	60-70	1
MP2	Clarias sp.	pectoral spine	1		
MP2	Clarias sp.	pectoral spine	1	30-40	1
MP2	Clarias sp.	pectoral spine	1	70-80	1
MP2	Clarias sp.	vomerine toothplate	1	50-60	1
MP2	Clarias sp.	cranial roof fragment	16		
MP2	Clarias sp.	cleithrum	2		
MP2	Haplotilapiini indet.	basioccipital	1	20-25	1
MP2	Haplotilapiini indet.	cleithrum	1	15-20	1
MP2	Haplotilapiini indet.	postcleithrum	1	15-20	1
MP2	Haplotilapiini indet.	precaudal vertebra	2	15-20	2
MP2	Clarias sp.	cleithrum	1		
MP2	Clarias sp.	cranial roof fragment	1		
MP2	Clarias sp.	pectoral spine	1	50-60	1
MP2	Clarias sp.	articular	1	50-60	1
MP2	Clarias sp.	articular	1	30-40	1
MP2	Clarias sp.	articular	1	30-40	1
MP2	Clarias sp.	articular	1	60-70	1
MP2	Clarias sp.	articular	1	70-80	1
MP2	Clarias sp.	articular	1	50-60	1
MP2	Clarias sp.	articular	1	60-70	1
MP2	Clarias sp.	articular	1	50-60	1
MP2	Clarias sp.	articular	1	40-50	1
MP2	Clarias sp.	articular	1	50-60	1
MP2	Clarias sp.	articular	1	30-40	1
MP2	Clarias sp.	articular	1	50-60	1
MP2	Clarias sp.	articular	1	20-25	1
MP2	Clarias sp.	articular	1	50-60	1
MP2	Clarias sp.	articular	1	70-80	1
MP2	Clarias sp.	articular	1	50-60	1
MP2	Clarias sp.	basioccipital	1	50-60	1
MP2	Clarias sp.	basipterygium	1	20-30	1
MP2	Clarias sp.	caudal vertebra	1	30-40	1
MP2	Clarias sp.	caudal vertebra	1	50-60	1
MP2	Clarias sp.	caudal vertebra	1	50-60	1
MP2	Clarias sp.	caudal vertebra	1	50-60	1
MP2	Clarias sp.	caudal vertebra	1	40-50	1
MP2	Clarias sp.	caudal vertebra	1	40-50	1
MP2	Clarias sp.	caudal vertebra	1	60-70	1
MP2	Clarias sp.	caudal vertebra	1	50-60	1
MP2	Clarias sp.	caudal vertebra	1	60-70	1
MP2	Clarias sp.	caudal vertebra	1	50-60	1
MP2	Clarias sp.	caudal vertebra	1	50-60	1
MP2	Clarias sp.	caudal vertebra	1	60-70	1
MP2	Clarias sp.	caudal vertebra	1	50-60	1
MP2	Clarias sp.	caudal vertebra	1	50-60	1
MP2	Clarias sp.	caudal vertebra	1		
MP2	Clarias sp.	caudal vertebra	1	40-50	1
MP2	Clarias sp.	caudal vertebra	1	60-70	1
MP2	Clarias sp.	caudal vertebra	1	50-60	1
MP2	Clarias sp.	caudal vertebra	1	60-70	1
MP2	Clarias sp.	caudal vertebra	1	60-70	1
MP2	Clarias sp.	vertebra	1	50-60	1
MP2	Clarias sp.	ceratohyale	1	60-70	1
MP2	Clarias sp.	ceratohyale	1	50-60	1
MP2	Clarias sp.	ceratohyale	1	40-50	1
MP2	Clarias sp.	ceratohyale	1	60-70	1
MP2	Clarias sp.	ceratohyale	1	40-50	1

cultural phase	taxon	skeletal element	NISP	SL in cm	number
MP2	Clarias sp.	ceratohyale	1	40-50	1
MP2	Clarias sp.	cleithrum	1		
MP2	Clarias sp.	cleithrum	1	40-50	1
MP2	Clarias sp.	cleithrum	1	40-50	1
MP2	Clarias sp.	cleithrum	1		
MP2	Clarias sp.	cleithrum	1		
MP2	Clarias sp.	cleithrum	1		
MP2	Clarias sp.	cleithrum	1	20-30	1
MP2	Clarias sp.	cleithrum	1		
MP2	Clarias sp.	cleithrum	1	40-50	1
MP2	Clarias sp.	cleithrum	1	40-50	1
MP2	Clarias sp.	cleithrum	1		
MP2	Clarias sp.	cleithrum	1	40-50	1
MP2	Clarias sp.	cleithrum	1	40-50	1
MP2	Clarias sp.	cleithrum	1	40-50	1
MP2	Clarias sp.	cleithrum	1	50-60	1
MP2	Clarias sp.	cleithrum	1		
MP2	Clarias sp.	cleithrum	1		
MP2	Clarias sp.	cleithrum	1		
MP2	Clarias sp.	cleithrum	1	40-50	1
MP2	Clarias sp.	cleithrum	1	50-60	1
MP2	Clarias sp.	cleithrum	1	30-40	1
MP2	Clarias sp.	cleithrum	1		
MP2	Clarias sp.	cleithrum	1	50-60	1
MP2	Clarias sp.	cleithrum	1	30-40	1
MP2	Clarias sp.	cleithrum	1	50-60	1
MP2	Clarias sp.	cleithrum	1	50-60	1
MP2	Clarias sp.	cleithrum	1		
MP2	Clarias sp.	cleithrum	1		
MP2	Clarias sp.	cleithrum	1	70-80	1
MP2	Clarias sp.	cleithrum	1	50-60	1
MP2	Clarias sp.	coracoid	1		
MP2	Clarias sp.	coracoid	1	20-30	1
MP2	Clarias sp.	coracoid	1	40-50	1
MP2	Clarias sp.	coracoid	1	40-50	1
MP2	Clarias sp.	coracoid	1	30-40	1
MP2	Clarias sp.	coracoid	1		
MP2	Clarias sp.	coracoid	1		
MP2	Clarias sp.	dentary	1	50-60	1
MP2	Clarias sp.	dentary	1	30-40	1
MP2	Clarias sp.	dentary	1	50-60	1
MP2	Clarias sp.	epihyale	1	40-50	1
MP2	Clarias sp.	epihyale	1	40-50	1
MP2	Clarias sp.	epihyale	1	60-70	1
MP2	Clarias sp.	hyomandibula	1		
MP2	Clarias sp.	hyomandibula	1	40-50	1
MP2	Clarias sp.	hyomandibula	1	30-40	1
MP2	Clarias sp.	hyomandibula	1	60-70	1
MP2	Clarias sp.	mesethmoid	1	30-40	1
MP2	Clarias sp.	mesethmoid	1	50-60	1
MP2	Clarias sp.	mesethmoid	1	40-50	1
MP2	Clarias sp.	mesethmoid	1	30-40	1
MP2	Clarias sp.	mesethmoid	1		
MP2	Clarias sp.	mesethmoid	1	40-50	1
MP2	Clarias sp.	cranial roof fragment	1		
MP2	Clarias sp.	cranial roof fragment	1		
MP2	Clarias sp.	cranial roof fragment	1		
MP2	Clarias sp.	cranial roof fragment	1		

cultural phase	taxon	skeletal element	NISP	SL in cm	number
MP2	Clarias sp.	cranial roof fragment	1		
MP2	Clarias sp.	cranial roof fragment	1		
MP2	Clarias sp.	cranial roof fragment	1		
MP2	Clarias sp.	cranial roof fragment	1	50-60	1
MP2	Clarias sp.	cranial roof fragment	1	40-50	1
MP2	Clarias sp.	cranial roof fragment	1	50-60	1
MP2	Clarias sp.	opercular	1		
MP2	Clarias sp.	palatinum	1	40-50	1
MP2	Clarias sp.	palatinum	1	40-50	1
MP2	Clarias sp.	precaudal vertebra	1	40-50	1
MP2	Clarias sp.	precaudal vertebra	1	60-70	1
MP2	Clarias sp.	precaudal vertebra	1	50-60	1
MP2	Clarias sp.	precaudal vertebra	1	20-25	1
MP2	Clarias sp.	precaudal vertebra	1	40-50	1
MP2	Clarias sp.	precaudal vertebra	1	40-50	1
MP2	Clarias sp.	precaudal vertebra	1	60-70	1
MP2	Clarias sp.	precaudal vertebra	1	60-70	1
MP2	Clarias sp.	precaudal vertebra	1	50-60	1
MP2	Clarias sp.	precaudal vertebra	1	60-70	1
MP2	Clarias sp.	precaudal vertebra	1	50-60	1
MP2	Clarias sp.	precaudal vertebra	1	40-50	1
MP2	Clarias sp.	precaudal vertebra	1	40-50	1
MP2	Clarias sp.	precaudal vertebra	1	60-70	1
MP2	Clarias sp.	precaudal vertebra	1	50-60	1
MP2	Clarias sp.	precaudal vertebra	1	60-70	1
MP2	Clarias sp.	precaudal vertebra	1	60-70	1
MP2	Clarias sp.	pectoral spine	1	40-50	1
MP2	Clarias sp.	pectoral spine	1	60-70	1
MP2	Clarias sp.	pectoral spine	1	60-70	1
MP2	Clarias sp.	pectoral spine	1	60-70	1
MP2	Clarias sp.	pectoral spine	1	60-70	1
MP2	Clarias sp.	pectoral spine	1	50-60	1
MP2	Clarias sp.	pectoral spine	1	50-60	1
MP2	Clarias sp.	pectoral spine	1	60-70	1
MP2	Clarias sp.	pectoral spine	1	40-50	1
MP2	Clarias sp.	quadrate	1	50-60	1
MP2	Clarias sp.	quadrate	1	70-80	1
MP2	Clarias sp.	quadrate	1	60-70	1
MP2	Clarias sp.	vomerine toothplate	1	50-60	1
MP2	Clarias sp.	urohyale	1	60-70	1
MP2	Clarias sp.	urohyale	1	50-60	1
MP2	Clarias sp.	vomerine toothplate	1	70-80	1
MP2	Clarias sp.	cranial roof fragment	10		
MP2	Clarias sp.	cranial roof fragment	13		
MP2	Clarias sp.	cranial roof fragment	14	50-60	1
MP2	Clarias sp.	cranial roof fragment	14		
MP2	Clarias sp.	cranial roof fragment	15	50-60	1
MP2	Clarias sp.	cranial roof fragment	15	70-80	1
MP2	Clarias sp.	cranial roof fragment	17		
MP2	Clarias sp.	cranial roof fragment	19	40-50	2
MP2	Clarias sp.	caudal vertebra	2	60-70	2
MP2	Clarias sp.	caudal vertebra	2	50-60	2
MP2	Clarias sp.	caudal vertebra	2	60-70	2
MP2	Clarias sp.	caudal vertebra	2	60-70	2
MP2	Clarias sp.	caudal vertebra	2	60-70	2
MP2	Clarias sp.	caudal vertebra	2	50-60	2
MP2	Clarias sp.	caudal vertebra	2	40-50	2
MP2	Clarias sp.	caudal vertebra	2	50-60	2

cultural phase	taxon	skeletal element	NISP	SL in cm	number
MP2	Clarias sp.	cranial roof fragment	2		
MP2	Clarias sp.	cranial roof fragment	2		
MP2	Clarias sp.	cranial roof fragment	2		
MP2	Clarias sp.	cranial roof fragment	2		
MP2	Clarias sp.	cranial roof fragment	2		
MP2	Clarias sp.	cranial roof fragment	2		
MP2	Clarias sp.	cranial roof fragment	2	60-70	2
MP2	Clarias sp.	cranial roof fragment	2	70-80	2
MP2	Clarias sp.	cranial roof fragment	2		
MP2	Clarias sp.	cranial roof fragment	2		
MP2	Clarias sp.	precaudal vertebra	2	60-70	2
MP2	Clarias sp.	precaudal vertebra	2	40-50	2
MP2	Clarias sp.	precaudal vertebra	2	50-60	2
MP2	Clarias sp.	precaudal vertebra	2	50-60	2
MP2	Clarias sp.	vertebra	2	40-50	2
MP2	Clarias sp.	caudal vertebra	3	50-60	
MP2	Clarias sp.	cranial roof fragment	3		
MP2	Clarias sp.	cranial roof fragment	3		
MP2	Clarias sp.	cranial roof fragment	3		
MP2	Clarias sp.	cranial roof fragment	3		
MP2	Clarias sp.	cranial roof fragment	3		
MP2	Clarias sp.	caudal vertebra	4	40-50	4
MP2	Clarias sp.	caudal vertebra	4	60-70	4
MP2	Clarias sp.	caudal vertebra	4	60-70	4
MP2	Clarias sp.	caudal vertebra	4	70-80	4
MP2	Clarias sp.	cleithrum	4		
MP2	Clarias sp.	cranial roof fragment	4	50-60	1
MP2	Clarias sp.	cranial roof fragment	4		
MP2	Clarias sp.	cranial roof fragment	4	60-70	1
MP2	Clarias sp.	cranial roof fragment	4	60-70	1
MP2	Clarias sp.	cranial roof fragment	4		
MP2	Clarias sp.	cranial roof fragment	4		
MP2	Clarias sp.	cranial roof fragment	4		
MP2	Clarias sp.	cranial roof fragment	4	20-30	4
MP2	Clarias sp.	cranial roof fragment	5	70-80	1
MP2	Clarias sp.	cranial roof fragment	5		
MP2	Clarias sp.	cranial roof fragment	5		
MP2	Clarias sp.	cranial roof fragment	5		
MP2	Clarias sp.	cranial roof fragment	6		
MP2	Clarias sp.	cranial roof fragment	6	40-50	1
MP2	Clarias sp.	cranial roof fragment	7		
MP2	Clarias sp.	cranial roof fragment	7	40-50	1
MP2	Clarias sp.	cranial roof fragment	9	40-50	2
MP2	Clarias sp.	cranial roof fragment	9	50-60	1
MP2	Haplotilapiini indet.	articular	1	15-20	1
MP2	Haplotilapiini indet.	articular	1	20-25	1
MP2	Haplotilapiini indet.	articular	1	25-30	1
MP2	Haplotilapiini indet.	basipterygium	1	15-20	1
MP2	Haplotilapiini indet.	basipterygium	1	15-20	1
MP2	Haplotilapiini indet.	basipterygium	1	15-20	1
MP2	Haplotilapiini indet.	basipterygium	1	15-20	1
MP2	Haplotilapiini indet.	caudal vertebra	1	15-20	1
MP2	Haplotilapiini indet.	caudal vertebra	1	15-20	1
MP2	Haplotilapiini indet.	caudal vertebra	1	15-20	1
MP2	Haplotilapiini indet.	caudal vertebra	1	20-25	1
MP2	Haplotilapiini indet.	caudal vertebra	1	15-20	1
MP2	Haplotilapiini indet.	caudal vertebra	1	20-25	1
MP2	Haplotilapiini indet.	caudal vertebra	1	15-20	1

cultural phase	taxon	skeletal element	NISP	SL in cm	number
MP2	Haplotilapiini indet.	caudal vertebra	1	15-20	1
MP2	Haplotilapiini indet.	caudal vertebra	1	15-20	1
MP2	Haplotilapiini indet.	caudal vertebra	1	15-20	1
MP2	Haplotilapiini indet.	caudal vertebra	1	20-25	1
MP2	Haplotilapiini indet.	cleithrum	1		
MP2	Haplotilapiini indet.	cleithrum	1	20-25	1
MP2	Haplotilapiini indet.	cleithrum	1		
MP2	Haplotilapiini indet.	cleithrum	1	15-20	1
MP2	Haplotilapiini indet.	cleithrum	1		
MP2	Haplotilapiini indet.	cleithrum	1	15-20	1
MP2	Haplotilapiini indet.	cleithrum	1	20-25	1
MP2	Haplotilapiini indet.	cleithrum	1	15-20	1
MP2	Haplotilapiini indet.	cleithrum	1		
MP2	Haplotilapiini indet.	cleithrum	1	20-25	1
MP2	Haplotilapiini indet.	cleithrum	1	15-20	1
MP2	O. niloticus	hyomandibula	1	15-20	1
MP2	Haplotilapiini indet.	hyomandibula	1	20-25	1
MP2	O. niloticus	hyomandibula	1	20-25	1
MP2	O. niloticus	hyomandibula	1	20-25	1
MP2	C. zillii	hyomandibula	1	20-25	1
MP2	Haplotilapiini indet.	hyomandibula	1	20-25	1
MP2	Haplotilapiini indet.	unidentified	1		
MP2	Haplotilapiini indet.	unidentified	1		
MP2	Haplotilapiini indet.	unidentified	1		
MP2	Haplotilapiini indet.	unidentified	1		
MP2	Haplotilapiini indet.	fin spine	1		
MP2	Haplotilapiini indet.	maxilla	1	25-30	1
MP2	Haplotilapiini indet.	skull roof fragment	1	15-20	1
MP2	Haplotilapiini indet.	skull roof fragment	1		
MP2	Haplotilapiini indet.	skull roof fragment	1	20-25	1
MP2	Haplotilapiini indet.	skull roof fragment	1		
MP2	Haplotilapiini indet.	skull roof fragment	1	15-20	1
MP2	Haplotilapiini indet.	opercular	1	15-20	1
MP2	Haplotilapiini indet.	opercular	1		
MP2	Haplotilapiini indet.	opercular	1		
MP2	Haplotilapiini indet.	opercular	1	15-20	1
MP2	Haplotilapiini indet.	opercular	1		
MP2	Haplotilapiini indet.	opercular	1	20-25	1
MP2	Haplotilapiini indet.	opercular	1		
MP2	Haplotilapiini indet.	opercular	1		
MP2	Haplotilapiini indet.	opercular	1		
MP2	Haplotilapiini indet.	opercular	1	20-25	1
MP2	Haplotilapiini indet.	opercular	1	15-20	1
MP2	Haplotilapiini indet.	opercular	1	15-20	1
MP2	Haplotilapiini indet.	opercular	1	20-25	1
MP2	Haplotilapiini indet.	opercular	1		
MP2	Haplotilapiini indet.	opercular	1	20-25	1
MP2	Haplotilapiini indet.	opercular	1	20-25	1
MP2	Haplotilapiini indet.	opercular	1		
MP2	Haplotilapiini indet.	opercular	1	25-30	1
MP2	Haplotilapiini indet.	opercular	1	15-20	1
MP2	Haplotilapiini indet.	opercular	1	15-20	1
MP2	Haplotilapiini indet.	opercular	1	15-20	1
MP2	Haplotilapiini indet.	opercular	1	20-25	1
MP2	Haplotilapiini indet.	opercular	1	15-20	1
MP2	Haplotilapiini indet.	opercular	1		
MP2	Haplotilapiini indet.	opercular	1	15-20	1
MP2	Haplotilapiini indet.	opercular	1	15-20	1

cultural phase	taxon	skeletal element	NISP	SL in cm	number
MP2	Haplotilapiini indet.	opercular	1	20-25	1
MP2	Haplotilapiini indet.	opercular	1		
MP2	Haplotilapiini indet.	opercular	1	20-25	1
MP2	Haplotilapiini indet.	2nd precaudal vertebra	1	25-30	1
MP2	Haplotilapiini indet.	2nd precaudal vertebra	1	20-25	1
MP2	C. zillii	1st precaudal vertebra	1	20-25	1
MP2	O. niloticus	1st precaudal vertebra	1	25-30	1
MP2	Haplotilapiini indet.	2nd precaudal vertebra	1	20-25	1
MP2	Haplotilapiini indet.	3rd precaudal vertebra	1	25-30	1
MP2	Haplotilapiini indet.	precaudal vertebra	1	15-20	1
MP2	Haplotilapiini indet.	precaudal vertebra	1	15-20	1
MP2	Haplotilapiini indet.	precaudal vertebra	1	15-20	1
MP2	Haplotilapiini indet.	precaudal vertebra	1	15-20	1
MP2	Haplotilapiini indet.	precaudal vertebra	1	15-20	1
MP2	Haplotilapiini indet.	precaudal vertebra	1	20-25	1
MP2	Haplotilapiini indet.	precaudal vertebra	1	15-20	1
MP2	Haplotilapiini indet.	precaudal vertebra	1	15-20	1
MP2	Haplotilapiini indet.	precaudal vertebra	1	15-20	1
MP2	Haplotilapiini indet.	premaxilla	1	15-20	1
MP2	Haplotilapiini indet.	preopercular	1	15-20	1
MP2	Haplotilapiini indet.	preopercular	1	20-25	1
MP2	Haplotilapiini indet.	preopercular	1	15-20	1
MP2	Haplotilapiini indet.	preopercular	1	20-25	1
MP2	Haplotilapiini indet.	preopercular	1	15-20	1
MP2	Haplotilapiini indet.	postcleithrum	1		
MP2	Haplotilapiini indet.	postcleithrum	1	15-20	1
MP2	Haplotilapiini indet.	postcleithrum	1	15-20	1
MP2	Haplotilapiini indet.	postcleithrum	1	20-20	1
MP2	Haplotilapiini indet.	dorsal or anal pterygophore	1		
MP2	Haplotilapiini indet.	dorsal or anal pterygophore	1	15-20	1
MP2	Haplotilapiini indet.	dorsal or anal pterygophore	1	20-25	1
MP2	Haplotilapiini indet.	dorsal or anal pterygophore	1	20-25	1
MP2	Haplotilapiini indet.	dorsal or anal pterygophore	1	15-20	1
MP2	Haplotilapiini indet.	dorsal or anal pterygophore	1	20-25	1
MP2	Haplotilapiini indet.	dorsal or anal pterygophore	1	15-20	1
MP2	Haplotilapiini indet.	dorsal or anal pterygophore	1	20-25	1
MP2	Haplotilapiini indet.	dorsal or anal pterygophore	1	20-25	1
MP2	Haplotilapiini indet.	dorsal or anal pterygophore	1	15-20	1
MP2	Haplotilapiini indet.	dorsal or anal pterygophore	1	15-20	1
MP2	Haplotilapiini indet.	dorsal or anal pterygophore	1	20-25	1
MP2	Haplotilapiini indet.	quadrate	1	15-20	1
MP2	Haplotilapiini indet.	quadrate	1	20-25	1
MP2	Haplotilapiini indet.	subopercular	1	20-25	1
MP2	Haplotilapiini indet.	supracleithrum	1		
MP2	Haplotilapiini indet.	supracleithrum	1	20-25	1
MP2	Haplotilapiini indet.	supracleithrum	1	20-25	1
MP2	Haplotilapiini indet.	supracleithrum	1	20-25	1
MP2	Haplotilapiini indet.	urophore	1	15-20	1
MP2	Haplotilapiini indet.	urohyale	1	15-20	1
MP2	Haplotilapiini indet.	urohyale	1	15-20	1
MP2	Haplotilapiini indet.	urohyale	1	15-20	1
MP2	Haplotilapiini indet.	caudal vertebra	2	15-20	2
MP2	Haplotilapiini indet.	caudal vertebra	2	15-20	2
MP2	Haplotilapiini indet.	caudal vertebra	2	15-20	2
MP2	Haplotilapiini indet.	caudal vertebra	2	15-20	2
MP2	Haplotilapiini indet.	cleithrum	2		
MP2	Haplotilapiini indet.	cleithrum	2		
MP2	Haplotilapiini indet.	cleithrum	2		

cultural phase	taxon	skeletal element	NISP	SL in cm	number
MP2	O. niloticus	cleithrum	2		
MP2	Haplotilapiini indet.	cleithrum	2	20-25	2
MP2	Haplotilapiini indet.	unidentified	2		
MP2	Haplotilapiini indet.	lepidotrich	2		
MP2	Haplotilapiini indet.	lepidotrich	2		
MP2	Haplotilapiini indet.	skull roof fragment	2	15-20	1
MP2	Haplotilapiini indet.	skull roof fragment	2	20-25	1
MP2	Haplotilapiini indet.	skull roof fragment	2	20-25	1
MP2	Haplotilapiini indet.	skull roof fragment	2	15-20	2
MP2	Haplotilapiini indet.	skull roof fragment	2		
MP2	Haplotilapiini indet.	skull roof fragment	2		
MP2	Haplotilapiini indet.	skull roof fragment	2		
MP2	Haplotilapiini indet.	precaudal vertebra	2	15-20	2
MP2	Haplotilapiini indet.	precaudal vertebra	2	15-20	2
MP2	Haplotilapiini indet.	precaudal vertebra	2	15-20	2
MP2	Haplotilapiini indet.	supracleithrum	2	20-25	2
MP2	Haplotilapiini indet.	caudal vertebra	3	15-20	3
MP2	Haplotilapiini indet.	skull roof fragment	3		
MP2	Haplotilapiini indet.	precaudal vertebra	3	15-20	3
MP2	Haplotilapiini indet.	cleithrum	4	20-25	1
MP2	Haplotilapiini indet.	cleithrum	5		
MP2	Haplotilapiini indet.	cleithrum	6		
MP2	Haplotilapiini indet.	cleithrum	7		
MP2	Haplotilapiini indet.	skull roof fragment	7	15-20	3
MP2	Clarias sp.	cranial roof fragment	4		
MP2	Clarias sp.	cleithrum	1		
MP2	Clarias sp.	hyomandibula	1		
MP2	Clarias sp.	pectoral spine	1		
MP2	Clarias sp.	articular	1		
MP2	Clarias sp.	caudal vertebra	3	50-60	3
MP2	Clarias sp.	caudal vertebra	1	60-70	1
MP2	Clarias sp.	caudal vertebra	1	70-80	1
MP2	Haplotilapiini indet.	basioccipital	1		
MP2	Haplotilapiini indet.	2nd precaudal vertebra	1		
MP2	Haplotilapiini indet.	cleithrum	1	20-25	1
MP2	Clarias sp.	articular	1	60-70	1
MP2	Clarias sp.	caudal vertebra	1	60-70	1
MP2	Clarias sp.	caudal vertebra	1	40-50	1
MP2	Clarias sp.	cleithrum	1	60-70	1
MP2	Clarias sp.	epihyale	1	70-80	1
MP2	Clarias sp.	precaudal vertebra	4	60-70	2
MP2	Clarias sp.	cranial roof fragment	6		
MP2	Haplotilapiini indet.	basipterygium	1		
MP2	Haplotilapiini indet.	skull roof fragment	1		
MP2	Haplotilapiini indet.	1st precaudal vertebra	1	15-20	1
MP2	Haplotilapiini indet.	1st precaudal vertebra	1	20-25	1
MP2	Haplotilapiini indet.	1st precaudal vertebra	1	25-30	1
MP2	Clarias sp.	caudal vertebra	1	50-60	1
MP2	Clarias sp.	ceratohyale	1	40-50	1
MP2	Clarias sp.	parasphenoid	1	60-70	1
MP2	Clarias sp.	dentary	1	50-60	1
MP2	Clarias sp.	dentary	1	50-60	1
MP2	Clarias sp.	cranial roof fragment	1		
MP2	Clarias sp.	caudal vertebra	1	60-70	1
MP2	Clarias sp.	cleithrum	1	40-50	1
MP2	Clarias sp.	coracoid	1	60-70	1
MP2	Clarias sp.	dentary	1	40-50	1
MP2	Clarias sp.	unidentified	1		

cultural phase	taxon	skeletal element	NISP	SL in cm	number
MP2	Clarias sp.	cranial roof fragment	10		
MP2	Haplotilapiini indet.	skull roof fragment	1		
MP2	Clarias sp.	precaudal vertebra	1	30-40	1
MP2	Clarias sp.	pectoral spine	1	60-70	1
MP2	Clarias sp.	cranial roof fragment	8	60-70	1
MP2	Haplotilapiini indet.	skull roof fragment	1	20-25	1
MP2	Haplotilapiini indet.	precaudal vertebra	1	20-25	1
MP2	Haplotilapiini indet.	dorsal or anal pterygophore	1	10-25	1
MP2	Haplotilapiini indet.	cleithrum	2		
MP2	Clarias sp.	caudal vertebra	1	60-70	1
MP2	Clarias sp.	branchial element	1		
MP2	Clarias sp.	precaudal vertebra	1	60-70	1
MP2	Clarias sp.	cranial roof fragment	3		
MP2	Haplotilapiini indet.	cleithrum	1	15-20	1
MP2	Haplotilapiini indet.	skull roof fragment	1	15-20	1
MP2	Haplotilapiini indet.	dorsal or anal pterygophore	1	10-15	1
MP2	Clarias sp.	caudal vertebra	1	70-80	1
MP2	Clarias sp.	mesethmoid	1	60-70	1
MP2	Clarias sp.	precaudal vertebra	1	40-50	1
MP2	Clarias sp.	cranial roof fragment	2		
MP2	Clarias sp.	cranial roof fragment	1	60-70	1
MP2	Clarias sp.	quadrate	1	70-80	1
MP2	Clarias sp.	cranial roof fragment	1		
MP2	Clarias sp.	pectoral spine	1		
MP2	Clarias sp.	cleithrum	1		
MP2	Clarias sp.	cranial roof fragment	1		
MP2	Clarias sp.	epihyale	1	60-70	1
MP2	Clarias sp.	cranial roof fragment	1		
MP2	Clarias sp.	precaudal vertebra	1		
MP2	Clarias sp.	cranial roof fragment	2		
MP2	Haplotilapiini indet.	basioccipital	1	20-25	1
MP2	Haplotilapiini indet.	dentary	1		
MP2	O. niloticus	hyomandibula	1	20-25	1
MP2	Haplotilapiini indet.	opercular	1	20-25	1
MP2	C. zillii	2nd precaudal vertebra	1	20-25	1
MP2	Haplotilapiini indet.	caudal vertebra	2	20-20	1
MP2	Clarias sp.	cleithrum	1		
MP2	Clarias sp.	cleithrum	1	80-90	1
MP2	Clarias sp.	cranial roof fragment	1		
MP2	Clarias sp.	cleithrum	2	40-50	1
MP2	Clarias sp.	cleithrum	2	50-60	1
MP2	O. niloticus	cleithrum	1	20-25	1
MP2	Haplotilapiini indet.	opercular	1	20-25	1
MP2	C. gariepinus	vomerine toothplate	1	50-60	1
MP2	Clarias sp.	articular & dentary	1	40-50	1
MP2	Clarias sp.	articular & dentary	1	30-40	1
MP2	Clarias sp.	cranial roof fragment	1		
MP2	Clarias sp.	cranial roof fragment	1	50-60	1
MP2	Clarias sp.	cranial roof fragment	1		
MP2	Clarias sp.	cranial roof fragment	1		
MP2	Clarias sp.	cranial roof fragment	1	90-100	1
MP2	Clarias sp.	precaudal vertebra	1	60-70	1
MP2	Clarias sp.	cranial roof fragment	2		
MP2	Haplotilapiini indet.	cleithrum	1	20-25	1
MP2	Clarias sp.	cranial roof fragment	2	50-60	1
MP2	Clarias sp.	cranial roof fragment	3		
MP2	Haplotilapiini indet.	opercular	1		
MP2	Clarias sp.	articular	1	50-60	1

cultural phase	taxon	skeletal element	NISP	SL in cm	number
MP2	Clarias sp.	cleithrum	1	50-60	1
MP2	Clarias sp.	cleithrum	1		
MP2	Clarias sp.	coracoid	1	40-50	1
MP2	Clarias sp.	coracoid	1		
MP2	Clarias sp.	dentary	1		
MP2	Clarias sp.	cranial roof fragment	1		
MP2	Clarias sp.	cranial roof fragment	1	50-60	1
MP2	Clarias sp.	cranial roof fragment	1	70-80	1
MP2	Clarias sp.	opercular	1	90-100	1
MP2	Clarias sp.	precaudal vertebra	1	50-60	1
MP2	Clarias sp.	urohyale	1	40-50	1
MP2	Clarias sp.	cranial roof fragment	14		
MP2	Clarias sp.	caudal vertebra	2		
MP2	Clarias sp.	cleithrum	2	40-50	1
MP2	Clarias sp.	cranial roof fragment	2		
MP2	Haplotilapiini indet.	anal pterygophore	1	15-20	1
MP2	Haplotilapiini indet.	anal pterygophore	1	20-25	1
MP2	Haplotilapiini indet.	cleithrum	1		
MP2	Haplotilapiini indet.	fin spine	1		
MP2	Haplotilapiini indet.	mesethmoid	1	20-25	1
MP2	Haplotilapiini indet.	mesethmoid	1	20-25	1
MP2	Haplotilapiini indet.	opercular	1	20-25	1
MP2	Haplotilapiini indet.	opercular	1	15-20	1
MP2	Haplotilapiini indet.	3rd precaudal vertebra	1	15-20	1
MP2	C. zillii	quadrate	1	15-20	1
MP2	O. niloticus	urohyale	1	15-20	1
MP2	Haplotilapiini indet.	cleithrum	3		
MP2	Clarias sp.	epi- & ceratohyale	1	40-50	1
MP2	Haplotilapiini indet.	suborbital	1	15-20	1
MP2	Clarias sp.	pectoral spine	1	60-70	1
MP2	Clarias sp.	cranial roof fragment	3		
MP2	Clarias sp.	cranial roof fragment	1	30-40	1
MP2	Clarias sp.	pectoral spine	1	40-50	1
MP2	Clarias sp.	cranial roof fragment	5	40-50	1
MP2	Clarias sp.	cleithrum	1		
MP2	Clarias sp.	cranial roof fragment	2		
MP2	Clarias sp.	cranial roof fragment	4		
MP2	Clarias sp.	precaudal vertebra	1	60-70	1
MP2	Clarias sp.	cranial roof fragment	2		
MP2	Clarias sp.	caudal vertebra	1	60-70	1
MP2	Clarias sp.	coracoid	1	60-70	1
MP2	Clarias sp.	cranial roof fragment	1	60-70	1
MP2	Clarias sp.	quadrate	1	50-60	1
MP2	Clarias sp.	hyomandibula	1		
MP2	Clarias sp.	cranial roof fragment	1		
MP2	Clarias sp.	precaudal vertebra	1	50-60	1
MP2	Clarias sp.	precaudal vertebra	1	60-70	1
MP2	Clarias sp.	precaudal vertebra	1	40-50	1
MP2	Clarias sp.	caudal vertebra	1	50-60	1
MP2	Clarias sp.	cleithrum	1		
MP2	Clarias sp.	pectoral spine	1	40-50	1
MP2	Clarias sp.	pectoral spine	1		
MP2	Clarias sp.	cranial roof fragment	2		
MP2	Clarias sp.	cranial roof fragment	1		
MP2	Clarias sp.	caudal vertebra	1	50-60	1
MP2	Clarias sp.	pectoral spine	1		
MP2	Clarias sp.	urohyale	1	60-70	1
MP2	Clarias sp.	cranial roof fragment	4	60-70	1

cultural phase	taxon	skeletal element	NISP	SL in cm	number
MP2	Clarias sp.	coracoid	1	40-50	1
MP2	Clarias sp.	mesethmoid	1	40-50	1
MP2	Clarias sp.	pectoral spine	1	50-60	1
MP2	Clarias sp.	cranial roof fragment	5	40-50	1
MP2	Clarias sp.	cranial roof fragment	1		
MP2	Clarias sp.	cranial roof fragment	1	40-50	1
MP2	Clarias sp.	cleithrum	1		
MP2	Clarias sp.	dentary	1	30-40	1
MP2	Clarias sp.	cranial roof fragment	3		
MP2	Clarias sp.	mesethmoid	1	40-50	1
MP2	Clarias sp.	cranial roof fragment	2		
MP2	Clarias sp.	precaudal vertebra	1	60-70	1
MP2	Clarias sp.	cleithrum	1		
MP2	Clarias sp.	cranial roof fragment	2		
MP2	Clarias sp.	cleithrum	1	50-60	1
MP2	Clarias sp.	pectoral spine	1	40-50	1
MP2	Clarias sp.	quadrate	1	30-40	1
MP2	Clarias sp.	hyomandibula	1		
MP2	Clarias sp.	cranial roof fragment	1		
MP2	Clarias sp.	basioccipital	1	70-80	1
MP2	Clarias sp.	caudal vertebra	1	40-50	1
MP2	Clarias sp.	caudal vertebra	1	50-60	1
MP2	Clarias sp.	caudal vertebra	1	60-70	1
MP2	Clarias sp.	cranial roof fragment	1	30-40	1
MP2	Clarias sp.	cranial roof fragment	1	40-50	2
MP2	Clarias sp.	cranial roof fragment	3		
MP2	Clarias sp.	caudal vertebra	1	30-40	1
MP2	Clarias sp.	hyomandibula	1	30-40	1
MP2	Clarias sp.	hyomandibula	1	60-70	1
MP2	Clarias sp.	cranial roof fragment	1	60-70	1
MP2	Clarias sp.	opercular	1	40-50	1
MP2	Clarias sp.	quadrate	1	60-70	1
MP2	Clarias sp.	cranial roof fragment	2	30-40	2
MP2	Clarias sp.	cranial roof fragment	9		
MP2	Clarias sp.	ceratohyale	1	60-70	1
MP2	Clarias sp.	cleithrum	1	40-50	1
MP2	Clarias sp.	cleithrum	1		
MP2	Clarias sp.	coracoid	1		
MP2	Clarias sp.	mesethmoid	1	70-80	1
MP2	Clarias sp.	precaudal vertebra	1	40-50	1
MP2	Clarias sp.	precaudal vertebra	1	60-70	1
MP2	Clarias sp.	quadrate	1	30-40	1
MP2	Clarias sp.	caudal vertebra	2	60-70	2
MP2	Clarias sp.	cranial roof fragment	8	60-70	1
MP2	Clarias sp.	opercular	1	30-40	1
MP2	Clarias sp.	precaudal vertebra	1	60-70	1
MP2	Clarias sp.	cranial roof fragment	2		
MP2	Clarias sp.	caudal vertebra	1	60-70	1
MP2	Clarias sp.	cranial roof fragment	3		
MP2	Clarias sp.	pectoral spine	1	50-60	1
MP2	Clarias sp.	ceratohyale	1	50-60	1
MP2	Clarias sp.	hyomandibula	1		
MP2	Clarias sp.	parasphenoid	1	60-70	1
MP2	Clarias sp.	precaudal vertebra	1	40-50	1
MP2	Clarias sp.	pectoral spine	1	50-60	1
MP2	Clarias sp.	quadrate	1	50-60	1
MP2	Clarias sp.	cranial roof fragment	4		
MP2	Clarias sp.	mesethmoid	1	60-70	1

cultural phase	taxon	skeletal element	NISP	SL in cm	number
MP2	Clarias sp.	cranial roof fragment	2		
MP2	Clarias sp.	cranial roof fragment	1		
MP2	Clarias sp.	precaudal vertebra	1	60-70	1
MP2	Clarias sp.	quadrate	1	50-60	1
MP2	Clarias sp.	quadrate	1	50-60	1
MP2	Clarias sp.	cleithrum	1		
MP2	Clarias sp.	precaudal vertebra	1	40-50	1
MP2	Clarias sp.	caudal vertebra	2	50-60	2
MP2	Clarias sp.	cranial roof fragment	5		
MP2	Haplotilapiini indet.	opercular	1		
MP2	Haplotilapiini indet.	dorsal or anal pterygophore	1	15-20	1
MP2	Haplotilapiini indet.	precaudal vertebra	1	15-20	1
MP2	Haplotilapiini indet.	cleithrum	1		
MP2	Haplotilapiini indet.	skull roof fragment	1		
MP2	Haplotilapiini indet.	opercular	1		
MP2	Haplotilapiini indet.	dorsal or anal pterygophore	1	15-20	1
MP2	Haplotilapiini indet.	dorsal or anal pterygophore	1	20-25	1
MP2	Haplotilapiini indet.	skull roof fragment	1		
MP2	Haplotilapiini indet.	fin spine	1		
MP2	Haplotilapiini indet.	opercular	1	20-25	1
MP2	C. zillii	preopercular	1	20-25	1
MP2	Haplotilapiini indet.	skull roof fragment	1		
MP2	O. niloticus	opercular	1		
MP2	Haplotilapiini indet.	caudal vertebra	1	15-20	1
MP2	Haplotilapiini indet.	caudal vertebra	1	25-30	1
MP2	Haplotilapiini indet.	cleithrum	1		
MP2	O. niloticus	mesethmoid	1		
MP2	Haplotilapiini indet.	postcleithrum	1		
MP2	Haplotilapiini indet.	3rd precaudal vertebra	1	20-25	1
MP2	Haplotilapiini indet.	preopercular	1		
MP2	Haplotilapiini indet.	cleithrum	2		
MP2	Haplotilapiini indet.	skull roof fragment	2		
MP2	Haplotilapiini indet.	skull roof fragment	1		
MP2	C. zillii	opercular	1	15-20	1
MP2	Haplotilapiini indet.	precaudal vertebra	1	15-20	1
MP2	Haplotilapiini indet.	dorsal or anal pterygophore	1	15-20	1
MP2	Haplotilapiini indet.	cleithrum	3		
MP2	O. niloticus	opercular	1	20-25	1
MP2	Haplotilapiini indet.	precaudal vertebra	1	15-20	1
MP2	Haplotilapiini indet.	dorsal or anal pterygophore	1	15-20	1
MP2	Haplotilapiini indet.	skull roof fragment	1		
MP2	Haplotilapiini indet.	opercular	1		
MP2	Haplotilapiini indet.	vertebra	1	15-20	1
MP2	Haplotilapiini indet.	precaudal vertebra	2	15-20	2
MP2	Haplotilapiini indet.	caudal vertebra	1	20-25	1
MP2	Haplotilapiini indet.	cleithrum	1		
MP2	Haplotilapiini indet.	opercular	1		
MP2	Haplotilapiini indet.	precaudal vertebra	1	20-25	1
MP2	Haplotilapiini indet.	opercular	1	20-25	1
MP2	Haplotilapiini indet.	dorsal or anal pterygophore	1		
MP2	Haplotilapiini indet.	caudal vertebra	1	15-20	1
MP2	Haplotilapiini indet.	precaudal vertebra	1	15-20	1
MP2	Haplotilapiini indet.	dorsal or anal pterygophore	1	15-20	1
MP2	Haplotilapiini indet.	skull roof fragment	1	15-20	1
MP2	Haplotilapiini indet.	dorsal or anal pterygophore	1	20-25	1
MP2	Haplotilapiini indet.	cleithrum	2		
MP2	O. niloticus	hyomandibula	1	20-25	1
MP2	Haplotilapiini indet.	dorsal or anal pterygophore	1	15-20	1

cultural phase	taxon	skeletal element	NISP	SL in cm	number
MP2	Haplotilapiini indet.	scapula	1		
MP2	Haplotilapiini indet.	cleithrum	2		
MP2	Haplotilapiini indet.	skull roof fragment	1		
MP2	Haplotilapiini indet.	opercular	1	15-20	1
MP2	Haplotilapiini indet.	preopercular	1	20-25	1
MP2	Haplotilapiini indet.	parasphenoid	1		
MP2	Haplotilapiini indet.	caudal vertebra	1	15-20	1
MP2	Haplotilapiini indet.	ceratohyale	1	15-20	1
MP2	Haplotilapiini indet.	opercular	1	15-20	1
MP2	Haplotilapiini indet.	precaudal vertebra	1	15-20	1
MP2	Haplotilapiini indet.	postcleithrum	1	15-20	1
MP2	Haplotilapiini indet.	dorsal or anal pterygophore	1	15-20	1
MP2	Haplotilapiini indet.	preopercular	1	20-25	1
MP2	Haplotilapiini indet.	dorsal or anal pterygophore	1	20-25	1
MP2	Haplotilapiini indet.	basipterygium	1	15-20	1
MP2	Haplotilapiini indet.	cleithrum	1		
MP2	Haplotilapiini indet.	precaudal vertebra	1	15-20	1
MP2	Clarias sp.	coracoid	1	40-50	1
MP2	Clarias sp.	cranial roof fragment	1		
MP2	Clarias sp.	cranial roof fragment	3		
MP2	Clarias sp.	cleithrum	1	40-50	1
MP2	Clarias sp.	dentary	1	40-50	1
MP2	Clarias sp.	cranial roof fragment	1		
MP2	Clarias sp.	articular	1	40-50	1
MP2	Clarias sp.	caudal vertebra	1	60-70	1
MP2	Clarias sp.	vomerine toothplate	1	50-60	1
MP2	Clarias sp.	cranial roof fragment	6		
MP2	Clarias sp.	cranial roof fragment	1		
MP2	Clarias sp.	basioccipital	1	30-40	1
MP2	Clarias sp.	caudal vertebra	1	50-60	1
MP2	Clarias sp.	hyomandibula	1	50-60	1
MP2	Clarias sp.	pectoral spine	1		
MP2	Clarias sp.	quadrate	1	50-60	1
MP2	Clarias sp.	cranial roof fragment	3	50-60	1
MP2	Clarias sp.	costa	1		
MP2	Clarias sp.	hyomandibula	1	40-50	1
MP2	Clarias sp.	opercular	1	30-40	1
MP2	Clarias sp.	quadrate	1	30-40	1
MP2	Clarias sp.	caudal vertebra	2	50-60	1
MP2	Clarias sp.	cranial roof fragment	5	30-40	1
MP2	Clarias sp.	cranial roof fragment	2		
MP2	Clarias sp.	caudal vertebra	1	50-60	1
MP2	Clarias sp.	ceratohyale	1	40-50	1
MP2	Clarias sp.	cleithrum	1		
MP2	Clarias sp.	urohyale	1	50-60	1
MP2	Clarias sp.	cranial roof fragment	5		
MP2	Clarias sp.	caudal vertebra	1	60-70	1
MP2	Clarias sp.	epihyale	1	60-70	1
MP2	Clarias sp.	epihyale	1	50-60	1
MP2	Clarias sp.	epihyale	1	30-40	1
MP2	Clarias sp.	cranial roof fragment	3	30-40	3
MP2	Clarias sp.	cranial roof fragment	8		
MP2	Clarias sp.	dentary	1		
MP2	Clarias sp.	cranial roof fragment	2		
MP2	Clarias sp.	cleithrum	1		
MP2	Clarias sp.	cranial roof fragment	3		
MP2	Clarias sp.	precaudal vertebra	1	60-70	1
MP2	Clarias sp.	cranial roof fragment	10	50-60	1

cultural phase	taxon	skeletal element	NISP	SL in cm	number
MP2	Clarias sp.	cleithrum	2		
MP2	Clarias sp.	cranial roof fragment	2	50-60	1
MP2	Clarias sp.	caudal vertebra	1	70-80	1
MP2	Clarias sp.	cranial roof fragment	1	30-40	1
MP2	Clarias sp.	cranial roof fragment	1	40-50	1
MP2	Clarias sp.	pectoral spine	1	50-60	1
MP2	Clarias sp.	cranial roof fragment	2		
MP2	Clarias sp.	caudal vertebra	1	40-50	1
MP2	Clarias sp.	ceratohyale	1	50-60	1
MP2	Clarias sp.	ceratohyale	1	40-50	1
MP2	Clarias sp.	cleithrum	1	30-40	1
MP2	Clarias sp.	cleithrum	1	40-50	1
MP2	Clarias sp.	cranial roof fragment	1	30-40	1
MP2	Clarias sp.	cranial roof fragment	1	60-70	1
MP2	Clarias sp.	opercular	1	50-60	1
MP2	Clarias sp.	precaudal vertebra	1	40-50	1
MP2	Clarias sp.	pectoral spine	1	50-60	1
MP2	Clarias sp.	cranial roof fragment	12		
MP2	Clarias sp.	caudal vertebra	2	60-70	2
MP2	Clarias sp.	coracoid	2		
MP2	Clarias sp.	cranial roof fragment	2	40-50	2
MP2	Clarias sp.	cranial roof fragment	2	50-60	2
MP2	Clarias sp.	basioccipital	1	60-70	1
MP2	Clarias sp.	cleithrum	1	40-50	1
MP2	Clarias sp.	cleithrum	1		
MP2	Clarias sp.	cranial roof fragment	1	50-60	1
MP2	Clarias sp.	cranial roof fragment	1	60-70	1
MP2	Clarias sp.	cranial roof fragment	1		
MP2	Clarias sp.	precaudal vertebra	1	40-50	1
MP2	Clarias sp.	precaudal vertebra	1	50-60	1
MP2	Clarias sp.	cranial roof fragment	2		
MP2	Clarias sp.	cranial roof fragment	1	50-60	1
MP2	Clarias sp.	cranial roof fragment	3		
MP2	Clarias sp.	cleithrum	1		
MP2	Clarias sp.	ceratohyale	1	30-40	1
MP2	Haplotilapiini indet.	opercular	1	15-20	1
MP2	Haplotilapiini indet.	3rd precaudal vertebra	1	15-20	1
MP2	Haplotilapiini indet.	precaudal vertebra	2	15-20	2
MP2	Haplotilapiini indet.	skull roof fragment	4		
MP2	Haplotilapiini indet.	cleithrum	5		
MP2	Haplotilapiini indet.	caudal vertebra	1	15-20	1
MP2	Haplotilapiini indet.	basipterygium	1		
MP2	Haplotilapiini indet.	cleithrum	1		
MP2	O. niloticus	opercular	1	20-25	1
MP2	Haplotilapiini indet.	3rd precaudal vertebra	1	20-25	1
MP2	Haplotilapiini indet.	preopercular	1	15-20	1
MP2	Haplotilapiini indet.	postcleithrum	1		
MP2	Haplotilapiini indet.	basioccipital	1	15-20	1
MP2	Haplotilapiini indet.	basipterygium	1	20-25	1
MP2	O. niloticus	hyomandibula	1	15-20	1
MP2	Haplotilapiini indet.	fin spine	1		
MP2	Haplotilapiini indet.	skull roof fragment	1		
MP2	Haplotilapiini indet.	opercular	1	15-20	1
MP2	Haplotilapiini indet.	3rd precaudal vertebra	1	15-20	1
MP2	Haplotilapiini indet.	precaudal vertebra	1	20-25	1
MP2	Haplotilapiini indet.	dorsal or anal pterygophore	1	20-25	1
MP2	Haplotilapiini indet.	precaudal vertebra	10	15-20	1
MP2	Haplotilapiini indet.	basipterygium	2	15-20	2

cultural phase	taxon	skeletal element	NISP	SL in cm	number
MP2	Haplotilapiini indet.	cleithrum	2		
MP2	Haplotilapiini indet.	preopercular	2		
MP2	Haplotilapiini indet.	caudal vertebra	3	15-20	3
MP2	Haplotilapiini indet.	interopercular	1		
MP2	Haplotilapiini indet.	opercular	1	20-25	1
MP2	Haplotilapiini indet.	caudal vertebra	2	15-20	2
MP2	Haplotilapiini indet.	cleithrum	2		
MP2	Haplotilapiini indet.	opercular	1		
MP2	Haplotilapiini indet.	basipterygium	1	15-20	1
MP2	Haplotilapiini indet.	caudal vertebra	1	15-20	1
MP2	O. niloticus	hyomandibula	1	20-25	1
MP2	Haplotilapiini indet.	lepidotrich	2		
MP2	Haplotilapiini indet.	cleithrum	4	15-20	3
MP2	Haplotilapiini indet.	skull roof fragment	1		
MP2	Haplotilapiini indet.	opercular	1		
MP2	Haplotilapiini indet.	basipterygium	1	20-25	1
MP2	Haplotilapiini indet.	caudal vertebra	1	15-20	1
MP2	Haplotilapiini indet.	fin spine	1		
MP2	Haplotilapiini indet.	skull roof fragment	1	20-25	1
MP2	Haplotilapiini indet.	opercular	1		
MP2	Haplotilapiini indet.	precaudal vertebra	1	15-20	1
MP2	Haplotilapiini indet.	subopercular	1		
MP2	Haplotilapiini indet.	supracleithrum	1	15-20	1
MP2	O. niloticus	urohyale	1	20-25	1
MP2	Haplotilapiini indet.	cleithrum	3		
MP2	Haplotilapiini indet.	cleithrum	1		
MP2	Haplotilapiini indet.	opercular	1		
MP2	C. zillii	maxilla	1	25-30	1
MP2	C. zillii	mesethmoid	1	15-20	1
MP2	Haplotilapiini indet.	skull roof fragment	2		
MP2	Haplotilapiini indet.	opercular	1	25-30	1
MP2	Haplotilapiini indet.	parasphenoid	1		
MP2	Clarias sp.	articular	1	30-40	1
MP2	Clarias sp.	caudal vertebra	1	60-70	1
MP2	Clarias sp.	mesethmoid	1	20-30	1
MP2	Clarias sp.	cranial roof fragment	1	30-40	1
MP2	Clarias sp.	cranial roof fragment	1	50-60	1
MP2	Clarias sp.	cranial roof fragment	5		
MP2	Clarias sp.	caudal vertebra	1	30-40	1
MP2	Clarias sp.	cleithrum	1	20-30	1
MP2	Clarias sp.	cleithrum	1	30-40	1
MP2	Clarias sp.	cleithrum	1	40-50	1
MP2	Clarias sp.	cleithrum	1	40-50	1
MP2	Clarias sp.	cleithrum	1	30-40	1
MP2	Clarias sp.	coracoid	1		
MP2	Clarias sp.	dentary	1	30-40	1
MP2	Clarias sp.	hyomandibula	1	40-50	1
MP2	Clarias sp.	cranial roof fragment	1	50-60	1
MP2	Clarias sp.	cranial roof fragment	1	60-70	1
MP2	Clarias sp.	precaudal vertebra	1	60-70	1
MP2	Clarias sp.	caudal vertebra	3	50-60	3
MP2	Clarias sp.	caudal vertebra	3	60-70	3
MP2	Clarias sp.	cleithrum	5		
MP2	Clarias sp.	cranial roof fragment	8		
MP2	Haplotilapiini indet.	basipterygium	1	20-25	1
MP2	Haplotilapiini indet.	opercular	1	20-25	1
MP2	Haplotilapiini indet.	opercular	1	15-20	1
MP2	Haplotilapiini indet.	opercular	1		

cultural phase	taxon	skeletal element	NISP	SL in cm	number
MP2	Haplotilapiini indet.	dorsal or anal pterygophore	1	15-20	1
MP2	Haplotilapiini indet.	dorsal or anal pterygophore	1	15-20	1
MP2	Haplotilapiini indet.	supracleithrum	1		
MP2	Haplotilapiini indet.	skull roof fragment	2		
MP2	Haplotilapiini indet.	precaudal vertebra	3	20-25	3
MP2	Haplotilapiini indet.	caudal vertebra	6	15-20	6
MP2	Haplotilapiini indet.	cleithrum	6		
MP2	Haplotilapiini indet.	precaudal vertebra	6	15-20	6
MP2	Haplotilapiini indet.	ceratohyale	1	20-25	1
MP2	Haplotilapiini indet.	cleithrum	1	15-20	1
MP2	Haplotilapiini indet.	cleithrum	1	20-25	1
MP2	Haplotilapiini indet.	dentary	1	25-30	1
MP2	O. niloticus	hyomandibula	1	15-20	1
MP2	O. niloticus	hyomandibula	1	15-20	1
MP2	Haplotilapiini indet.	opercular	1		
MP2	Haplotilapiini indet.	preopercular	1		
MP2	Haplotilapiini indet.	dorsal or anal pterygophore	1	20-25	1
MP2	Haplotilapiini indet.	dorsal or anal pterygophore	1	20-25	1
MP2	Haplotilapiini indet.	dorsal or anal pterygophore	1		
MP2	Haplotilapiini indet.	lepidotrich	2		
MP2	Haplotilapiini indet.	precaudal vertebra	5	20-25	5
MP2	Haplotilapiini indet.	caudal vertebra	6	15-20	6
MP2	Haplotilapiini indet.	precaudal vertebra	6	15-20	6
MP2	Clarias sp.	cranial roof fragment	1		
MP2	O. niloticus	hyomandibula	1	20-25	1
MP2	Haplotilapiini indet.	hyomandibula	1		
MP2	Haplotilapiini indet.	opercular	1	15-20	1
MP2	Haplotilapiini indet.	precaudal vertebra	1	15-20	1
MP2	O. niloticus	cleithrum	2		
MP2	Haplotilapiini indet.	skull roof fragment	3		
MP2	Clarias sp.	articular	1	30-40	1
MP2	Haplotilapiini indet.	opercular	1		
MP2	Clarias sp.	opercular	1	40-50	1
MP2	Clarias sp.	caudal vertebra	3	40-50	3
MP2	Clarias sp.	cranial roof fragment	3		
MP2	Clarias sp.	cranial roof fragment	2		
MP2	Haplotilapiini indet.	skull roof fragment	1		
MP2	Clarias sp.	cranial roof fragment	1	40-50	1
MP2	Clarias sp.	cranial roof fragment	1	60-70	1
MP2	Clarias sp.	cranial roof fragment	2		
MP2	Clarias sp.	basioccipital	1	30-40	1
MP2	Clarias sp.	precaudal vertebra	1	50-60	1
MP2	Clarias sp.	articular	1	50-60	1
MP2	Clarias sp.	cleithrum	2		
MP2	Clarias sp.	cranial roof fragment	5		
MP2	Clarias sp.	cranial roof fragment	1		
MP2	Clarias sp.	palatinum	1	70-80	1
MP2	Clarias sp.	urohyale	1	50-60	1
MP2	Clarias sp.	cranial roof fragment	2		
MP2	Clarias sp.	cleithrum	1		
MP2	Clarias sp.	mesethmoid	1	40-50	1
MP2	Clarias sp.	pectoral spine	1	40-50	1
MP2	Clarias sp.	caudal vertebra	2	50-60	1
MP2	Clarias sp.	cranial roof fragment	7		
MP2	Clarias sp.	epihyale	1	30-40	1
MP2	Clarias sp.	vomerine toothplate	1	50-60	1
MP2	Clarias sp.	cranial roof fragment	2		
MP2	Clarias sp.	caudal vertebra	1	60-70	1

cultural phase	taxon	skeletal element	NISP	SL in cm	number
MP2	Clarias sp.	mesethmoid	1	40-50	1
MP2	Clarias sp.	mesethmoid	1	40-50	1
MP2	Clarias sp.	opercular	1	30-40	1
MP2	Clarias sp.	cranial roof fragment	5		
MP2	Clarias sp.	articular	1	40-50	1
MP2	Clarias sp.	ceratohyale	1	40-50	1
MP2	Clarias sp.	dentary	1	40-50	1
MP2	Clarias sp.	hyomandibula	1	50-60	1
MP2	Clarias sp.	caudal vertebra	2	60-70	2
MP2	Clarias sp.	cleithrum	2		
MP2	Clarias sp.	cranial roof fragment	2	40-50	2
MP2	Clarias sp.	precaudal vertebra	2	60-70	2
MP2	Clarias sp.	cranial roof fragment	3	60-70	3
MP2	Clarias sp.	cranial roof fragment	7		
MP2	Clarias sp.	cleithrum	1	40-50	1
MP2	Clarias sp.	coracoid	1	50-60	1
MP2	Clarias sp.	cranial roof fragment	1	30-40	1
MP2	Clarias sp.	quadrate	1	40-50	1
MP2	Clarias sp.	caudal vertebra	2	50-60	2
MP2	O. niloticus	cleithrum	1		
MP2	O. niloticus	cleithrum	1		
MP2	Haplotilapiini indet.	parasphenoid	1		
MP2	Haplotilapiini indet.	caudal vertebra	1	15-20	1
MP2	Haplotilapiini indet.	caudal vertebra	1	25-30	1
MP2	Haplotilapiini indet.	fin spine	1		
MP2	Haplotilapiini indet.	2nd precaudal vertebra	1	25-30	1
MP2	Haplotilapiini indet.	cleithrum	1		
MP2	Haplotilapiini indet.	opercular	1		
MP2	Haplotilapiini indet.	supracleithrum	1		
MP2	Haplotilapiini indet.	preopercular	1	20-25	1
MP2	Haplotilapiini indet.	basipterygium	1	15-20	1
MP2	Haplotilapiini indet.	caudal vertebra	1	15-20	1
MP2	Haplotilapiini indet.	cleithrum	1		
MP2	Haplotilapiini indet.	3rd precaudal vertebra	1	25-30	1
MP2	Haplotilapiini indet.	precaudal vertebra	1	15-20	1
MP2	Haplotilapiini indet.	premaxilla	1	20-25	1
MP2	Haplotilapiini indet.	dorsal or anal pterygophore	1	15-20	1
MP2	Haplotilapiini indet.	lepidotrich	2		
MP2	Haplotilapiini indet.	opercular	2		
MP2	Haplotilapiini indet.	skull roof fragment	3		
MP2	Haplotilapiini indet.	cleithrum	1		
MP2	Haplotilapiini indet.	preopercular	1	20-25	1
MP2	Haplotilapiini indet.	supracleithrum	1	20-25	1
MP2	Clarias sp.	caudal vertebra	1	50-60	1
MP2	Clarias sp.	cranial roof fragment	6		
MP2	Haplotilapiini indet.	basipterygium	1	15-20	1
MP2	Haplotilapiini indet.	caudal vertebra	1	15-20	1
MP2	Haplotilapiini indet.	cleithrum	1		
MP2	Haplotilapiini indet.	fin spine	1		
MP2	Haplotilapiini indet.	dorsal or anal pterygophore	1	15-20	1
MP2	Haplotilapiini indet.	supracleithrum	1	15-20	1
MP2	Clarias sp.	cranial roof fragment	2		
MP2	Clarias sp.	articular	1	30-40	1
MP2	Clarias sp.	basioccipital	1	70-80	1
MP2	Clarias sp.	quadrate	1	40-50	1
MP2	Clarias sp.	caudal vertebra	2	60-70	2
MP2	Clarias sp.	cranial roof fragment	8	40-50	1
MP2	Haplotilapiini indet.	caudal vertebra	1		

cultural phase	taxon	skeletal element	NISP	SL in cm	number
MP2	Haplotilapiini indet.	fin spine	1		
MP2	Haplotilapiini indet.	skull roof fragment	1		
MP2	Haplotilapiini indet.	opercular	1	20-25	1
MP2	Haplotilapiini indet.	precaudal vertebra	1	20-25	1
MP2	Haplotilapiini indet.	preopercular	1	20-25	1
MP2	Haplotilapiini indet.	dorsal or anal pterygophore	1	20-25	1
MP2	Haplotilapiini indet.	dorsal or anal pterygophore	1		
MP2	Haplotilapiini indet.	cleithrum	7		
MP2	Clarias sp.	cranial roof fragment	2		
MP2	Haplotilapiini indet.	1st precaudal vertebra	1	20-25	1
MP2	Haplotilapiini indet.	1st precaudal vertebra	1	15-20	1
MP2	O. niloticus	1st precaudal vertebra	1	20-25	1
LP1	Clarias sp.	cranial roof fragment	1		
LP1	Haplotilapiini indet.	precaudal vertebra	1	15-20	1
LP1	Haplotilapiini indet.	opercular	1	20-25	1
LP1	Clarias sp.	cranial roof fragment	1		
LP1	Clarias sp.	cranial roof fragment	1		
LP1	Clarias sp.	articular	1	70-80	1
LP1	O. niloticus	hyomandibula	1	25-30	1
LP1	Clarias sp.	cranial roof fragment	2		
LP1	Haplotilapiini indet.	opercular	1		
LP1	Clarias sp.	articular	1	50-60	1
LP1	Clarias sp.	articular	1	40-50	1
LP1	Clarias sp.	articular	1	60-70	1
LP1	Clarias sp.	articular	1	60-70	1
LP1	Clarias sp.	articular	1	50-60	1
LP1	Clarias sp.	articular	1	70-80	1
LP1	Clarias sp.	basioccipital	1	40-50	1
LP1	Clarias sp.	caudal vertebra	1	60-70	1
LP1	Clarias sp.	caudal vertebra	1	50-60	1
LP1	Clarias sp.	caudal vertebra	1	50-60	1
LP1	Clarias sp.	caudal vertebra	1	60-70	1
LP1	Clarias sp.	caudal vertebra	1		
LP1	Clarias sp.	ceratohyale	1	70-80	1
LP1	Clarias sp.	ceratohyale	1	30-40	1
LP1	Clarias sp.	ceratohyale	1	40-50	1
LP1	Clarias sp.	ceratohyale	1	30-40	1
LP1	Clarias sp.	ceratohyale	1	60-70	1
LP1	Clarias sp.	ceratohyale	1	40-50	1
LP1	Clarias sp.	cleithrum	1	50-60	1
LP1	Clarias sp.	cleithrum	1		
LP1	Clarias sp.	cleithrum	1	50-60	1
LP1	Clarias sp.	cleithrum	1	30-40	1
LP1	Clarias sp.	cleithrum	1		
LP1	Clarias sp.	coracoid	1		
LP1	Clarias sp.	coracoid	1		
LP1	Clarias sp.	coracoid	1		
LP1	Clarias sp.	dentary	1	40-50	1
LP1	Clarias sp.	mesethmoid	1	40-50	1
LP1	Clarias sp.	cranial roof fragment	1		
LP1	Clarias sp.	cranial roof fragment	1		
LP1	Clarias sp.	cranial roof fragment	1		
LP1	Clarias sp.	cranial roof fragment	1		
LP1	Clarias sp.	cranial roof fragment	1		
LP1	Clarias sp.	cranial roof fragment	1		
LP1	Clarias sp.	cranial roof fragment	1		
LP1	Clarias sp.	cranial roof fragment	1		
LP1	Clarias sp.	cranial roof fragment	1		

cultural phase	taxon	skeletal element	NISP	SL in cm	number
LP1	Clarias sp.	precaudal vertebra	1	60-70	1
LP1	Clarias sp.	precaudal vertebra	1	60-70	1
LP1	Clarias sp.	precaudal vertebra	1	40-50	1
LP1	Clarias sp.	precaudal vertebra	1	50-60	1
LP1	Clarias sp.	precaudal vertebra	1	60-70	1
LP1	Clarias sp.	pectoral spine	1	30-40	1
LP1	Clarias sp.	pectoral spine	1	40-50	1
LP1	Clarias sp.	pectoral spine	1	30-40	1
LP1	Clarias sp.	pectoral spine	1	30-40	1
LP1	Clarias sp.	pectoral spine	1	40-50	1
LP1	Clarias sp.	vomerine toothplate	1	50-60	1
LP1	Clarias sp.	caudal vertebra	2		
LP1	Clarias sp.	cleithrum	2	40-50	2
LP1	Clarias sp.	cranial roof fragment	2		
LP1	Clarias sp.	cranial roof fragment	2		
LP1	Clarias sp.	cranial roof fragment	2		
LP1	Clarias sp.	cranial roof fragment	2		
LP1	Clarias sp.	cranial roof fragment	2		
LP1	Clarias sp.	cranial roof fragment	3		
LP1	Clarias sp.	cranial roof fragment	3	60-70	1
LP1	Clarias sp.	cranial roof fragment	3		
LP1	Clarias sp.	cranial roof fragment	4		
LP1	Clarias sp.	cranial roof fragment	4		
LP1	Clarias sp.	cranial roof fragment	5		
LP1	Clarias sp.	cranial roof fragment	6		
LP1	Clarias sp.	cranial roof fragment	6		
LP1	Clarias sp.	cranial roof fragment	6		
LP1	Clarias sp.	cranial roof fragment	7		
LP1	Clarias sp.	cranial roof fragment	8		
LP1	Clarias sp.	cranial roof fragment	9		
LP1	Haplotilapiini indet.	basipterygium	1	15-20	1
LP1	Haplotilapiini indet.	basipterygium	1	20-25	1
LP1	Haplotilapiini indet.	caudal vertebra	1		
LP1	Haplotilapiini indet.	caudal vertebra	1	15-20	1
LP1	Haplotilapiini indet.	cleithrum	1		
LP1	Haplotilapiini indet.	cleithrum	1		
LP1	Haplotilapiini indet.	cleithrum	1		
LP1	Haplotilapiini indet.	fin spine	1		
LP1	Haplotilapiini indet.	fin spine	1		
LP1	Haplotilapiini indet.	skull roof fragment	1		
LP1	Haplotilapiini indet.	skull roof fragment	1		
LP1	Haplotilapiini indet.	opercular	1		
LP1	Haplotilapiini indet.	opercular	1		
LP1	Haplotilapiini indet.	opercular	1		
LP1	Haplotilapiini indet.	opercular	1		
LP1	Haplotilapiini indet.	opercular	1		
LP1	O. niloticus	opercular	1	15-20	1
LP1	Haplotilapiini indet.	parasphenoid	1		
LP1	Haplotilapiini indet.	precaudal vertebra	1	15-20	1
LP1	Haplotilapiini indet.	precaudal vertebra	1	15-20	1
LP1	Haplotilapiini indet.	precaudal vertebra	1	15-20	1
LP1	Haplotilapiini indet.	preopercular	1	20-25	1
LP1	Haplotilapiini indet.	preopercular	1		
LP1	Haplotilapiini indet.	postcleithrum	1	20-25	1
LP1	Haplotilapiini indet.	dorsal or anal pterygophore	1		
LP1	Haplotilapiini indet.	dorsal or anal pterygophore	1	20-25	1
LP1	O. niloticus	cleithrum	3		
LP1	Clarias sp.	cranial roof fragment	2	50-60	1

cultural phase	taxon	skeletal element	NISP	SL in cm	number
LP1	Haplotilapiini indet.	basipterygium	1	15-20	1
LP1	Clarias sp.	cranial roof fragment	1		
LP1	Clarias sp.	precaudal vertebra	1	40-50	1
LP1	Haplotilapiini indet.	caudal vertebra	1	15-20	1
LP1	Haplotilapiini indet.	fin spine	1		
LP1	Clarias sp.	mesethmoid	1	50-60	1
LP1	Clarias sp.	cleithrum	1	40-50	1
LP1	Clarias sp.	cranial roof fragment	1		
LP1	Clarias sp.	articular	1	50-60	1
LP1	Clarias sp.	articular	1	50-60	1
LP1	Clarias sp.	articular	1	40-50	1
LP1	Clarias sp.	basioccipital	1	60-70	1
LP1	Clarias sp.	ceratohyale	1	50-60	1
LP1	Clarias sp.	dentary	1	50-60	1
LP1	Clarias sp.	dentary	1	40-50	1
LP1	Clarias sp.	dentary	1		
LP1	Clarias sp.	dentary	1	70-80	1
LP1	Clarias sp.	cranial roof fragment	1	70-80	1
LP1	Clarias sp.	pectoral spine	1	70-80	1
LP1	Clarias sp.	quadrate	1	50-60	1
LP1	Clarias sp.	cleithrum	2		
LP1	Clarias sp.	coracoid	2		
LP1	Clarias sp.	cranial roof fragment	2	50-60	2
LP1	Clarias sp.	precaudal vertebra	3	60-70	3
LP1	Clarias sp.	cranial roof fragment	31		
LP1	Clarias sp.	caudal vertebra	8		
LP1	Haplotilapiini indet.	skull roof fragment	1		
LP1	Haplotilapiini indet.	dorsal or anal pterygophore	1	15-20	1
LP1	Haplotilapiini indet.	cleithrum	2	20-25	2
LP1	Haplotilapiini indet.	cleithrum	3	15-20	3
LP1	Clarias sp.	caudal vertebra	1	50-60	1
LP1	Clarias sp.	ceratohyale	1	50-60	1
LP1	Clarias sp.	unidentified	1		
LP1	Clarias sp.	quadrate	1	60-70	1
LP1	Clarias sp.	cranial roof fragment	8	70-80	2
LP1	Haplotilapiini indet.	opercular	1	15-20	1
LP1	Haplotilapiini indet.	parasphenoid	1	20-25	1
LP1	Haplotilapiini indet.	suborbital	1		
LP1	Clarias sp.	cranial roof fragment	1		
LP1	Clarias sp.	cranial roof fragment	3		
LP1	Clarias sp.	pectoral spine	1	50-60	1
LP1	Clarias sp.	cranial roof fragment	2		
LP1	Haplotilapiini indet.	unidentified	1		
LP1	Clarias sp.	cleithrum	1	30-40	1
LP1	Clarias sp.	cranial roof fragment	1		
LP1	Clarias sp.	caudal vertebra	1	40-50	1
LP1	Clarias sp.	cleithrum	1	30-40	1
LP1	Clarias sp.	cleithrum	1	50-60	1
LP1	Clarias sp.	hyomandibula	1		
LP1	Clarias sp.	precaudal vertebra	1	40-50	1
LP1	Clarias sp.	cranial roof fragment	2	60-70	1
LP1	Clarias sp.	caudal vertebra	1		
LP1	Clarias sp.	dentary	1	40-50	1
LP1	Clarias sp.	cranial roof fragment	1	60-70	1
LP1	Clarias sp.	cranial roof fragment	2		
LP1	Clarias sp.	cranial roof fragment	1		
LP1	Haplotilapiini indet.	basipterygium	1		
LP1	Haplotilapiini indet.	skull roof fragment	1		

cultural phase	taxon	skeletal element	NISP	SL in cm	number
LP1	Haplotilapiini indet.	caudal vertebra	3	15-20	3
LP1	Haplotilapiini indet.	precaudal vertebra	3	15-20	3
LP1	Haplotilapiini indet.	opercular	1		
LP1	Haplotilapiini indet.	precaudal vertebra	1	25-30	1
LP1	Haplotilapiini indet.	dorsal or anal pterygophore	1	15-20	1
LP1	Clarias sp.	cranial roof fragment	1		
LP1	Clarias sp.	articular	1	50-60	1
LP1	Clarias sp.	cranial roof fragment	1		
disturbed	Clarias sp.	articular	1	40-50	1
disturbed	Clarias sp.	coracoid	1	40-50	1
disturbed	Clarias sp.	mesethmoid	1	40-50	1
disturbed	Clarias sp.	precaudal vertebra	1	40-50	1
disturbed	Clarias sp.	precaudal vertebra	1	60-70	1
disturbed	Clarias sp.	pectoral spine	1	50-60	1
disturbed	Clarias sp.	pectoral spine	1	50-60	1
disturbed	Clarias sp.	cranial roof fragment	12		
disturbed	Clarias sp.	caudal vertebra	2	60-70	2
disturbed	Clarias sp.	cranial roof fragment	3	50-60	3
disturbed	Clarias sp.	cleithrum	1	40-50	1
disturbed	Clarias sp.	coracoid	1		
disturbed	Haplotilapiini indet.	cleithrum	1		
disturbed	Haplotilapiini indet.	unidentified	1		
disturbed	Haplotilapiini indet.	subopercular	1	20-25	1
disturbed	Clarias sp.	mesethmoid	1	70-80	1
disturbed	Clarias sp.	cranial roof fragment	3		
disturbed	Clarias sp.	cleithrum	1	20-25	1
disturbed	Clarias sp.	precaudal vertebra	1	50-60	1
disturbed	Clarias sp.	pectoral spine	1	60-70	1
disturbed	Clarias sp.	cranial roof fragment	6		
disturbed	Clarias sp.	cleithrum	1	40-50	1
disturbed	Clarias sp.	cleithrum	1	60-70	1
disturbed	Clarias sp.	mesethmoid	1	40-50	1
disturbed	Clarias sp.	cranial roof fragment	3		
NA	Clarias sp.	basioccipital	1		
NA	Clarias sp.	articular	1		
NA	Clarias sp.	basioccipital	1		
NA	Clarias sp.	basioccipital	1		
NA	Clarias sp.	caudal vertebra	1		
NA	Clarias sp.	caudal vertebra	1		
NA	Clarias sp.	cleithrum	1		
NA	Clarias sp.	cleithrum	1		
NA	Clarias sp.	epihyale	1		
NA	Clarias sp.	palatinum	1		
NA	Clarias sp.	parasphenoid	1		
NA	Clarias sp.	precaudal vertebra	1		
NA	Clarias sp.	precaudal vertebra	1		
NA	Clarias sp.	precaudal vertebra	1		
NA	Clarias sp.	precaudal vertebra	1		
NA	Clarias sp.	pectoral spine	1		
NA	Clarias sp.	quadrate	1		
NA	Clarias sp.	vomerine toothplate	1		
NA	Clarias sp.	vomerine toothplate	1		
NA	Clarias sp.	cranial roof fragment	15		
NA	Clarias sp.	caudal vertebra	2		
NA	Clarias sp.	caudal vertebra	3		
NA	Clarias sp.	cranial roof fragment	3		
NA	Clarias sp.	cranial roof fragment	5		
NA	Clarias sp.	cranial roof fragment	9		

cultural phase	taxon	skeletal element	NISP	SL in cm	number
NA	Haplotilapiini indet.	caudal vertebra	1		
NA	Haplotilapiini indet.	caudal vertebra	1		
NA	Haplotilapiini indet.	fin spine	1		
NA	Haplotilapiini indet.	fin spine	1		
NA	Haplotilapiini indet.	2nd precaudal vertebra	1		
NA	Haplotilapiini indet.	3rd precaudal vertebra	1		
NA	Haplotilapiini indet.	precaudal vertebra	1		
NA	Haplotilapiini indet.	preopercular	1		
NA	Haplotilapiini indet.	dorsal or anal pterygophore	1		
NA	Haplotilapiini indet.	dorsal or anal pterygophore	1		
NA	Haplotilapiini indet.	dorsal or anal pterygophore	1		
NA	Haplotilapiini indet.	supracleithrum	1		
NA	Haplotilapiini indet.	skull roof fragment	2		
NA	Haplotilapiini indet.	caudal vertebra	3		
NA	Haplotilapiini indet.	cleithrum	4		
NA	Clarias sp.	unidentified	1		
NA	Clarias sp.	pectoral spine	1		
NA	Clarias sp.	caudal vertebra	2		
NA	Clarias sp.	cranial roof fragment	2		
NA	Clarias sp.	cranial roof fragment	3		
NA	Clarias sp.	cranial roof fragment	5		
NA	Haplotilapiini indet.	skull roof fragment	1		
NA	Haplotilapiini indet.	opercular	1		
NA	Clarias sp.	ceratohyale	1		
NA	Clarias sp.	ceratohyale	1		
NA	Clarias sp.	ceratohyale	1		
NA	Clarias sp.	cranial roof fragment	1		
NA	Clarias sp.	hyomandibula	1		
NA	Clarias sp.	opercular	1		
NA	Clarias sp.	pectoral spine	1		
NA	Clarias sp.	cranial roof fragment	2		
NA	Clarias sp.	hyomandibula	2		
NA	Clarias sp.	cranial roof fragment	3		
NA	Haplotilapiini indet.	maxilla	1		
NA	Haplotilapiini indet.	postcleithrum	1		
NA	Haplotilapiini indet.	1st precaudal vertebra	1		
NA	Haplotilapiini indet.	quadrate	1		
NA	O. niloticus	urohyale	1		
NA	Haplotilapiini indet.	preopercular	2		
NA	Clarias sp.	caudal vertebra	1		
NA	Clarias sp.	caudal vertebra	1		
NA	Clarias sp.	cleithrum	1		
NA	Clarias sp.	cleithrum	1		
NA	Clarias sp.	coracoid	1		
NA	Clarias sp.	cranial roof fragment	1		
NA	Clarias sp.	opercular	1		
NA	Clarias sp.	opercular	1		
NA	Clarias sp.	quadrate	1		
NA	Clarias sp.	vomerine toothplate	1		
NA	Clarias sp.	vomerine toothplate	1		
NA	Clarias sp.	coracoid	2		
NA	Clarias sp.	caudal vertebra	5		
NA	Clarias sp.	cranial roof fragment	8		
NA	Clarias sp.	articular	1		
NA	Clarias sp.	articular	1		
NA	Clarias sp.	articular	1		
NA	Clarias sp.	ceratohyale	1		
NA	Clarias sp.	ceratohyale	1		

cultural phase	taxon	skeletal element	NISP	SL in cm	number
NA	Clarias sp.	ceratohyale	1		
NA	Clarias sp.	ceratohyale	1		
NA	Clarias sp.	cleithrum	3		
NA	Clarias sp.	basioccipital	1		
NA	Clarias sp.	epihyale	1		
NA	Clarias sp.	cranial roof fragment	1		
NA	Clarias sp.	hyomandibula	1		
NA	Clarias sp.	palatinum	1		
NA	Clarias sp.	pectoral spine	1		
NA	Clarias sp.	quadrate	1		
NA	Clarias sp.	quadrate	1		
NA	Clarias sp.	quadrate	1		
NA	Clarias sp.	cranial roof fragment	11		
NA	Clarias sp.	cranial roof fragment	3		
NA	Clarias sp.	precaudal vertebra	5		
NA	Clarias sp.	caudal vertebra	8		
NA	Haplotilapiini indet.	cleithrum	1		
NA	Clarias sp.	cranial roof fragment	3		
NA	Clarias sp.	cranial roof fragment	2		
NA	Haplotilapiini indet.	caudal vertebra	1		
NA	Haplotilapiini indet.	caudal vertebra	1		
NA	Haplotilapiini indet.	precaudal vertebra	1		
NA	Clarias sp.	articular	1		
NA	Clarias sp.	articular & dentary	1		
NA	Clarias sp.	hyomandibula	1		
NA	Clarias sp.	mesethmoid	1		
NA	Clarias sp.	cranial roof fragment	2		
NA	Clarias sp.	hypohyale	1		
NA	Clarias sp.	cranial roof fragment	1		
NA	Haplotilapiini indet.	cleithrum	1		
NA	Haplotilapiini indet.	precaudal vertebra	1		
NA	Clarias sp.	cranial roof fragment	1		
NA	Clarias sp.	cleithrum	1		
NA	Clarias sp.	cranial roof fragment	1		
NA	Clarias sp.	cranial roof fragment	1		
NA	Clarias sp.	cleithrum	1		
NA	Clarias sp.	cranial roof fragment	1		
NA	Clarias sp.	cranial roof fragment	1		
NA	Clarias sp.	precaudal vertebra	1		
NA	Clarias sp.	caudal vertebra	3		
NA	Clarias sp.	articular	1		
NA	Clarias sp.	cranial roof fragment	1		
NA	Clarias sp.	cranial roof fragment	6		
NA	Clarias sp.	coracoid	1		
NA	Clarias sp.	cranial roof fragment	3		
NA	Clarias sp.	cleithrum	1		
NA	Clarias sp.	articular	1		
NA	Clarias sp.	cranial roof fragment	1		
NA	Clarias sp.	pectoral spine	1		
NA	Clarias sp.	ceratohyale	1		
NA	Clarias sp.	cleithrum	1		
NA	Clarias sp.	mesethmoid	1		
NA	Clarias sp.	cleithrum	1		
NA	Clarias sp.	cranial roof fragment	7		
NA	Haplotilapiini indet.	cleithrum	1		
NA	Haplotilapiini indet.	urohyale	1		
NA	Clarias sp.	cranial roof fragment	2		
NA	Clarias sp.	cranial roof fragment	2		

cultural phase	taxon	skeletal element	NISP	SL in cm	number
NA	Haplotilapiini indet.	fin spine	1		
NA	Haplotilapiini indet.	skull roof fragment	1	15-20	1
NA	Haplotilapiini indet.	opercular	1	15-20	1
NA	Clarias sp.	articular	1		
NA	Clarias sp.	cleithrum	1		
NA	Clarias sp.	coracoid	1		
NA	Clarias sp.	dentary	1		
NA	Clarias sp.	dentary	1		
NA	Clarias sp.	cranial roof fragment	1		
NA	Clarias sp.	cranial roof fragment	1		
NA	Clarias sp.	precaudal vertebra	1		
NA	Clarias sp.	cranial roof fragment	6		
NA	Clarias sp.	caudal vertebra	1		
NA	Clarias sp.	cleithrum	1		
NA	Clarias sp.	pectoral spine	1		
NA	Clarias sp.	cranial roof fragment	2		
NA	Haplotilapiini indet.	skull roof fragment	1		
NA	Haplotilapiini indet.	skull roof fragment	1		
NA	Haplotilapiini indet.	cleithrum	2		
NA	Haplotilapiini indet.	precaudal vertebra	2		
NA	Clarias sp.	cleithrum	1		
NA	Clarias sp.	coracoid	1		
NA	Clarias sp.	precaudal vertebra	1		
NA	Clarias sp.	cranial roof fragment	10		
NA	Haplotilapiini indet.	cleithrum	1		
NA	Clarias sp.	caudal vertebra	1		
NA	Clarias sp.	dentary	1		
NA	Clarias sp.	caudal vertebra	2		
NA	Clarias sp.	cranial roof fragment	3		
NA	Clarias sp.	ceratohyale	1		
NA	Clarias sp.	unidentified	1		
NA	Clarias sp.	cranial roof fragment	1		
NA	Haplotilapiini indet.	caudal vertebra	1		
NA	Haplotilapiini indet.	1st precaudal vertebra	1		
NA	O. niloticus	hyomandibula	1		
NA	Haplotilapiini indet.	dorsal or anal pterygophore	1		
NA	Haplotilapiini indet.	cleithrum	2		
NA	Clarias sp.	cranial roof fragment	1		
NA	Clarias sp.	cranial roof fragment	2		
NA	Haplotilapiini indet.	skull roof fragment	1		
NA	Clarias sp.	cranial roof fragment	1		
NA	Clarias sp.	pectoral spine	1		
NA	Haplotilapiini indet.	preopercular	1		
NA	Haplotilapiini indet.	caudal vertebra	3		
NA	Haplotilapiini indet.	precaudal vertebra	3		
NA	Clarias sp.	cleithrum	1		
NA	Clarias sp.	cranial roof fragment	3		
NA	Clarias sp.	cranial roof fragment	2		
NA	Clarias sp.	opercular	1		
NA	Clarias sp.	precaudal vertebra	1		
NA	Clarias sp.	cleithrum	4		
NA	Clarias sp.	cranial roof fragment	5		
NA	Haplotilapiini indet.	cleithrum	1		
NA	Haplotilapiini indet.	dorsal or anal pterygophore	1		
NA	Clarias sp.	cranial roof fragment	1		
NA	Clarias sp.	cleithrum	1		
NA	Clarias sp.	cleithrum	1		
NA	Clarias sp.	basioccipital	1		

cultural phase	taxon	skeletal element	NISP	SL in cm	number
NA	Clarias sp.	caudal vertebra	1		
NA	Clarias sp.	coracoid	1		
NA	Clarias sp.	cranial roof fragment	11		
NA	Haplotilapiini indet.	cleithrum	1		
